# Global, neuronal or β cell-specific deletion of inceptor improves glucose homeostasis in male mice with diet-induced obesity

**DOI:** 10.1038/s42255-024-00991-3

**Published:** 2024-02-28

**Authors:** Gerald Grandl, Gustav Collden, Jin Feng, Sreya Bhattacharya, Felix Klingelhuber, Leopold Schomann, Sara Bilekova, Ansarullah, Weiwei Xu, Fataneh Fathi Far, Monica Tost, Tim Gruber, Aimée Bastidas-Ponce, Qian Zhang, Aaron Novikoff, Arkadiusz Liskiewicz, Daniela Liskiewicz, Cristina Garcia-Caceres, Annette Feuchtinger, Matthias H. Tschöp, Natalie Krahmer, Heiko Lickert, Timo D. Müller

**Affiliations:** 1 Institute of Diabetes and Obesity, Helmholtz Center Munich, Neuherberg, Germany; 2 German Center for Diabetes Research, Neuherberg, Germany; 3 Institute of Diabetes and Regeneration Research, Helmholtz Center Munich, Neuherberg, Germany; 4 School of Medicine, Technische Universität München, Munich, Germany; 5 Core Facility Pathology & Tissue Analytics, Helmholtz Center Munich, Munich, Germany; 6 Medizinische Klinik und Poliklinik IV, Klinikum der Universität, Ludwig-Maximilians-Universität München, Munich, Germany; 7 Helmholtz Zentrum München, Neuherberg, Germany; 8 Walther-Straub Institute of Pharmacology and Toxicology, LMU Munich, Munich, Germany

**Keywords:** Type 2 diabetes, Obesity, Metabolism, Endocrine system and metabolic diseases

## Abstract

Insulin resistance is an early complication of diet-induced obesity (DIO)^1^, potentially leading to hyperglycaemia and hyperinsulinaemia, accompanied by adaptive β cell hypertrophy and development of type 2 diabetes^2^. Insulin not only signals via the insulin receptor (INSR), but also promotes β cell survival, growth and function via the insulin-like growth factor 1 receptor (IGF1R)^3–6^. We recently identified the insulin inhibitory receptor (inceptor) as the key mediator of IGF1R and INSR desensitization^7^. But, although β cell-specific loss of inceptor improves β cell function in lean mice^7^, it warrants clarification whether inceptor signal inhibition also improves glycaemia under conditions of obesity. We assessed the glucometabolic effects of targeted inceptor deletion in either the brain or the pancreatic β cells under conditions of DIO in male mice. In the present study, we show that global and neuronal deletion of inceptor, as well as its adult-onset deletion in the β cells, improves glucose homeostasis by enhancing β cell health and function. Moreover, we demonstrate that inceptor-mediated improvement in glucose control does not depend on inceptor function in agouti-related protein-expressing or pro-opiomelanocortin neurons. Our data demonstrate that inceptor inhibition improves glucose homeostasis in mice with DIO, hence corroborating that inceptor is a crucial regulator of INSR and IGF1R signalling.

## Abstract (web summary)

In male mice with diet-induced obesity, deletion of insulin inhibitory receptor (inceptor) in the whole body, in the brain and in pancreatic β cells improves glucose homeostasis, underlining a role of inceptor in regulating glucose homeostasis in the brain and pancreas.

## Main

Insulin resistance (IR) is one of the greatest healthcare challenges of our time^1^. An acute hallmark of IR is β cell hypertrophy, accompanied by progressive hyperinsulinaemia and hyperglycaemia, which may ultimately result in the development of type 2 diabetes (T2D)^2^. IR is often a consequence of prolonged abdominal obesity and is thought to be closely linked to overconsumption of a fat- and carbohydrate-rich western diet^8^. Understanding the multifaceted aetiology of IR is of utmost importance^9^, because it can cause overt T2D characterized by progressive β cell failure and dependency on insulin replacement therapy^2^. Albeit best known for its glucose-lowering effect, insulin promotes its biological action not only via the INSR, but also via the IGF1R to jointly regulate β cell survival, growth and function^3–6^. Consistent with this, although *Insr* knockout (KO) causes only mild diabetic symptoms with impaired insulin secretory function and total IR in β cells, *Insr/Igf1r* double KO causes overt diabetes associated with reduced β cell mass, increased apoptosis and severely compromised β cell function^10–12^. To ensure adequate islet INSR/IGF1R signalling, insulin-induced receptor activation has to be terminated at some point and the sensitivity of the INSR and IGF1R to get activated by its respective ligand has to be restored. It was only recently that we discovered a key mediator underlying this process in the pancreas, which we named inceptor (encoded by the gene *Iir*)^7^. Consistent with the ability of insulin to signal via INSR and IGF1R, inducible β cell-specific ablation of inceptor increases signalling via both INSR and IGF1R, leading to increased β cell mass and improved glucose tolerance in lean, chow-fed, normoglycaemic mice^7^. But a key open question that remains is whether inceptor inhibition also improves glycaemia under conditions of DIO and glucose intolerance. Besides the pancreas, expression of inceptor is highest in the brain and the pituitary^7^, which further raises the question of whether inceptor also regulates glucose metabolism via central mechanisms. Based on the ability of inceptor to improve islet glucose metabolism via enhanced ligand-induced INSR and IGF1R clathrin-mediated endocytosis and desensitization^7^, modulation of inceptor activity may carry pharmacological potential for the treatment of IR and/or T2D. To corroborate the pharmacological potential of the inceptor–INSR/IGF1R axis, we determined in the present study the spatial and cellular localization of inceptor in the brain, and explored whether whole-body inceptor deficiency, adult-onset β cell-specific loss of inceptor or its targeted Cre-mediated deletion in the central nervous system (CNS), or specifically in pro-opiomelanocortin (POMC) or agouti-related protein (AgRP)-expressing neurons, affects energy and/or glucose metabolism under conditions of DIO in male mice.

**Fig. 1:**
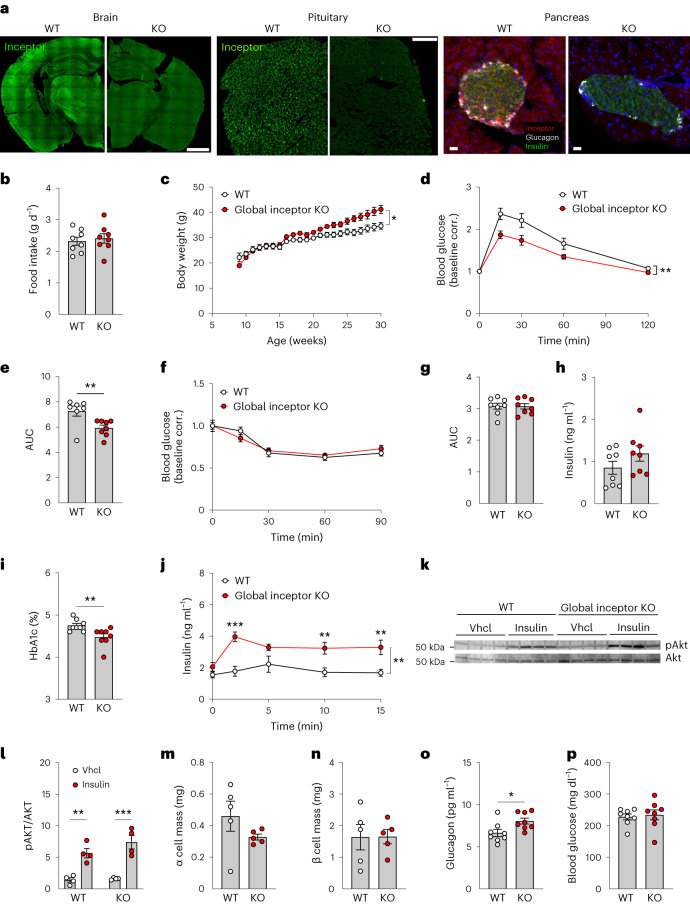
Whole-body inceptor deletion improves glucose metabolism in male mice with DIO. **a**, Inceptor immunoreactivity in whole brain, pituitary and pancreas of 10-week-old male C57BL/6J inceptor *Iir*^*+/+*^ (WT) and *Iir*^−*/*−^ (KO) mice. Scale bars, 1 mm for brain, 200 µm for pituitary and 20 µm for pancreas. **b**,**c**, Food intake (**b**) in 28-week-old and body weight (**c**) in 10- to 30-week-old male C57BL/6J WT and KO mice (*n* = 8 for each genotype). **d**,**e**, Intraperitoneal glucose tolerance (**d**) and area under the curve (AUC) (**e**) in 19-week-old male C57BL/6J WT and KO mice (*n* = 7 WT, *n* = 8 KO). **f**,**g**, Intraperitoneal insulin tolerance (**f**) and AUC (**g**) in 23-week-old male C57BL/6J WT and KO mice (*n* = 8 for each genotype). **h**,**i**, Fasting levels of plasma insulin (*n* = 8 for each genotype) in 19-week-old male C57BL/6J WT and KO mice (*n* = 8 for each genotype) (**h**) and levels of HbA1c in 27-week-old male C57BL/6J WT and KO mice (*n* = 8 for each genotype) (**i**). **j**, Glucose-stimulated insulin secretion in 27-week-old male C57BL/6J WT and KO mice (*n* = 8 for each genotype). **k**,**l**, Western blots of liver protein of 30-week-old male C57BL/6J WT and KO mice treated with a single dose of saline or 1 mU per kg of insulin (*n* = 4 for each group) (**k**) and densitometric quantification (**l**). **m**,**n**, Pancreatic α cell (**m**) and β cell (**n**) mass in 30-week-old male C57BL/6J WT and KO mice (*n* = 5 for each genotype). **o**,**p**, Fasting plasma levels of glucagon (**o**) and blood glucose (**p**) in 19-week-old male C57BL/6J WT and KO mice (*n* = 8 for each genotype). ^*^P < 0.05, ^**^P < 0.01, ^***^P < 0.001. Data are presented as mean ± s.e.m. Data in **b**, **e**, **g**–**i** and **l**–**p** were analysed by two-sided, two-tailed Student’s *t*-test. Data in **c**, **d**, **f** and **j** were analysed by two-way ANOVA with Bonferroni’s post hoc, multiple-comparison test for the different time points. *P* values for group differences are: *P* = 0.0049 (**c**), *P* = 0.0082 (**d**), *P* = 0.010 (**e**), *P* = 0.0098 (**i**), *P* = 0.0021 (**j**), *P* = 0.002 (WT) and *P* = 0.0016 (KO) (**l**) and *P* = 0.0232 (**o**). *P* values for the multiple-comparison tests are provided in Source data. Corr., corrected; vhcl, vehicle. Source data

**Extended Data Fig. 1:**
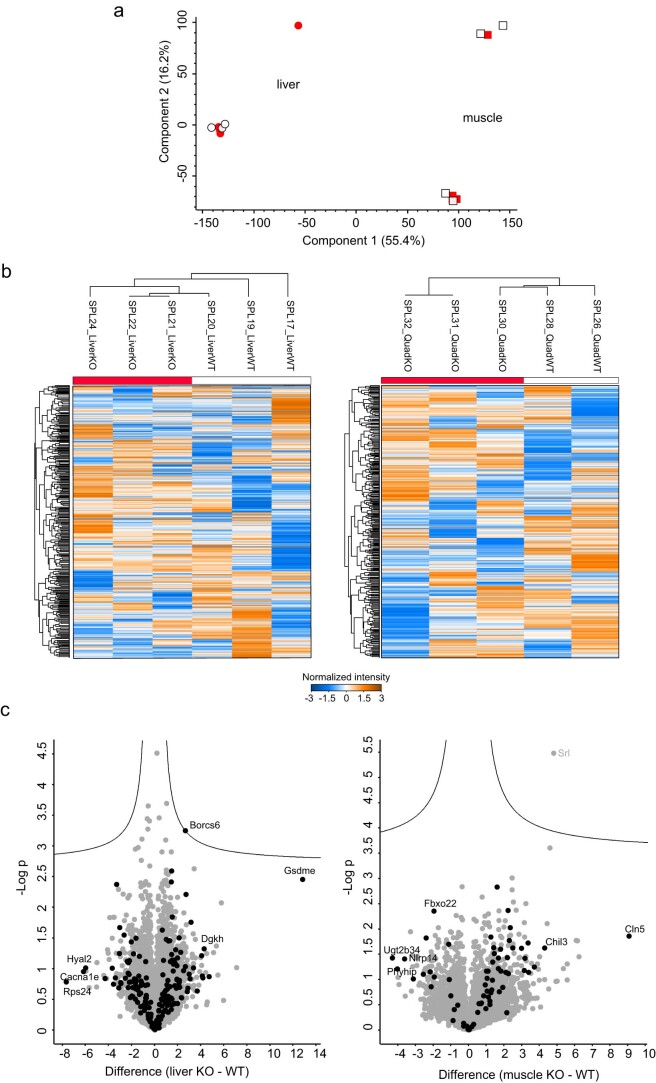
Proteome analysis of liver and muscle of whole body inceptor KO vs. WT mice. Principle component analysis (PCA) of all tissue samples (**a**). Clustering of quadriceps muscle (left), liver (middle) and hypothalamus (right) samples as well as z-scored protein intensities. Bar beneath the clustering indicates genotype (WT: white, KO: red) (n = 3 each genotype for liver, n = 3 WT, n = 2 KO for muscle) (**b**). Volcano plots of the same organs as in (**b**) ProteinGroups only found in one condition are indicated in black (**c**) (n = 3 each genotype for liver, n = 3 WT, n = 2 KO for muscle). Source data

To assess whether inceptor inhibition also improves glucose control under conditions of DIO, we generated global inceptor KO mice by crossing *Iir*^*flx/flx*^ mice with mice that express Cre recombinase under the control of the Rosa26 promoter^13^. Confirming successful target deletion, global inceptor KO mice show largely diminished inceptor immunoreactivity in the brain, pituitary and pancreas relative to wild-type (*Iir*^*wt/wt*^) (WT) controls (Fig. 1a). When fed with a high-fat diet (HFD), global inceptor KO mice show normal food intake (Fig. 1b), but slightly increased body weight relative to WT controls (Fig. 1c). Impressively, despite showing greater body weight, DIO global inceptor KO mice show improved glucose tolerance relative to WT controls with DIO (Fig. 1d,e), without changes in insulin sensitivity (Fig. 1f,g) or baseline levels of plasma insulin (Fig. 1h). But, consistent with the improved glucose tolerance (Fig. 1d,e), DIO global inceptor KO mice show decreased levels of glycated haemoglobin (HbA1c) (Fig. 1i) and enhanced glucose-stimulated insulin secretion relative to WT controls with DIO (Fig. 1j). Collectively, these data suggest that improved glucose metabolism in global inceptor KO mice originates from enhanced insulin secretion under non-basal conditions, without changes in insulin sensitivity. Consistent with the observation that insulin sensitivity is not changed in the obese inceptor KO mice, we found, after bolus insulin administration, no changes in phosphorylation of the protein kinase AKT in the liver between DIO global inceptor KO mice and their WT controls (Fig. 1k,l). Mass spectrometry (MS)-based proteomic analysis in insulin-sensitive tissues, namely muscle and liver, likewise revealed no overt changes in the proteome signature between DIO global inceptor KO mice and WT controls (Extended Data Fig. 1a–c). No differences are further observed in α cell or β cell mass (Fig. 1m,n), but plasma levels of glucagon are increased in inceptor KO mice, without changes in fasting levels of blood glucose (Fig. 1o,p).

**Fig. 2:**
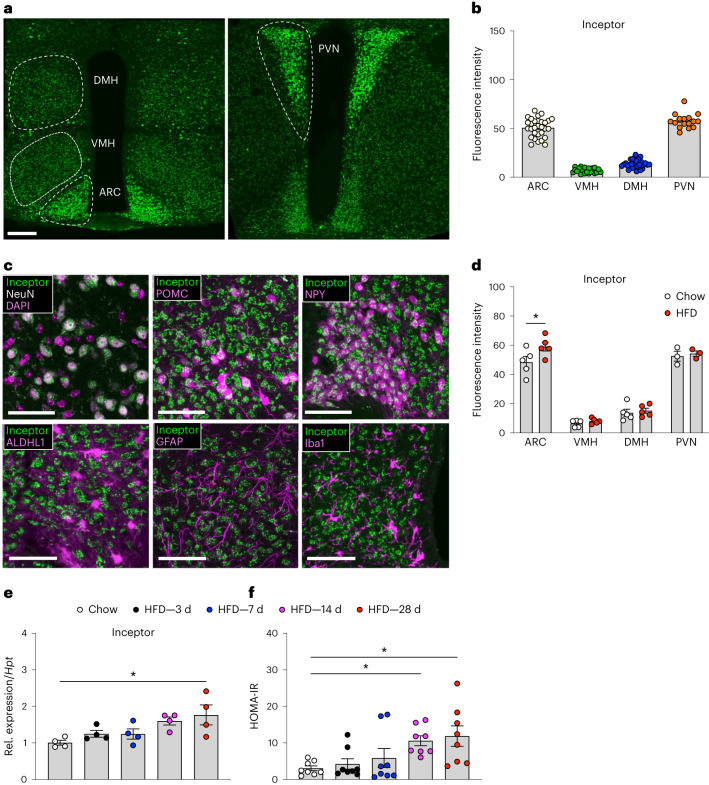
Central inceptor immunoreactivity is restricted to neurons, including those regulating energy and glucose metabolism. **a**,**b**, Representative image (**a**) and quantification (**b**) of inceptor immunoreactivity in hypothalamic nuclei of 18- to 24-week-old male C57BL/6J WT mice (*n* = 30 mice for ARC and DMH, *n* = 28 mice for VMH, *n* = 19 mice for PVN). Scale bar, 200 μm. **c**, Inceptor colocalization with *NeuN*, *Pomc*–green fluorescent protein (GFP), *Npy*–GFP, *Aldhl1*–GFP, *Gfap* and *Iba1* in the hypothalamus of 28-week-old male C57BL/6J WT mice. Scale bars, 50 μm. **d**, Inceptor immunoreactivity in 28-week-old chow- versus HFD-fed male C57BL/6J WT mice (*n* = 5 for all regions except PVN: *n* = 3). **e**, Hypothalamic inceptor messenger RNA expression in 10-week-old male C57BL/6J WT mice fed with either chow or HFD (*n* = 4 per group). **f**, HOMA-IR in 14-week-old C57BL/6J WT mice fed with either chow or HFD (*n* = 8). ^*^P < 0.05, Data are presented as mean ± s.e.m. Data in **b** and **e** were analysed by one-way ANOVA, data in **d** were analysed by two-way ANOVA and data in **f** were analysed using the Kruskal–Wallace test. *P* values for group differences are: *P* < 0.0001 (ARC versus VMH), *P* < 0.0001 (ARC versus DMH), *P* = 0.0046 (ARC versus PVN), *P* = 0.0014 (VMH versus DMH), *P* < 0.0001 (VMH versus PNV), *P* < 0.0001 (DMH versus PVN) (**b**), *P* = 0.0156 (**d**), *P* = 00137 (**e**), *P* = 0.0217 (chow versus HFD 14 d) and *P* = 0.0232 (chow versus HFD 28 d) (**f**). A more detailed statistical report is provided in Source data. Rel., relative. Source data

Based on the predominant expression of inceptor in the CNS, pituitary and pancreas, we next investigated the spatiotemporal expression pattern of inceptor in the CNS more closely. Consistent with previous reports^7^, we found broad inceptor immunoreactivity in the adult mouse brain (Fig. 1a). In the hypothalamus, inceptor immunoreactivity was high in the arcuate and paraventricular nuclei (ARC and PVN, respectively) and low in the dorsomedial hypothalamic and ventromedial hypothalamic nuclei (DMH and VMH, respectively) (Fig. 2a,b). In the hypothalamus, inceptor immunoreactivity colocalized with the neuronal marker neuronal nuclear protein (*NeuN*, also known as *Fox3*), but not with markers indicative of astrocytes (aldehyde dehydrogenase 1 family member L1, *Aldh1l1*), microglia (glial fibrillary acylated protein, *Gfap*) or neuroglia (ionized calcium-binding adaptor molecule 1, *Iba1*) (Fig. 2c), hence indicating that inceptor was primarily located in CNS neurons. In the hypothalamic ARC, we found inceptor colocalized with neurons that express *Pomc* and neuropeptide Y (*Npy*) (Fig. 2c). It is interesting that hypothalamic inceptor immunoreactivity is increased in the ARC of obese relative to lean mice, with no difference in the PVN, DMH or VMH (Fig. 2d). Hypothalamic inceptor expression further gradually increases during the progression of HFD exposure, and this is paralleled by increased IR, as estimated by homeostatic model assessment for IR (HOMA-IR) (Fig. 2e,f).

**Fig. 3:**
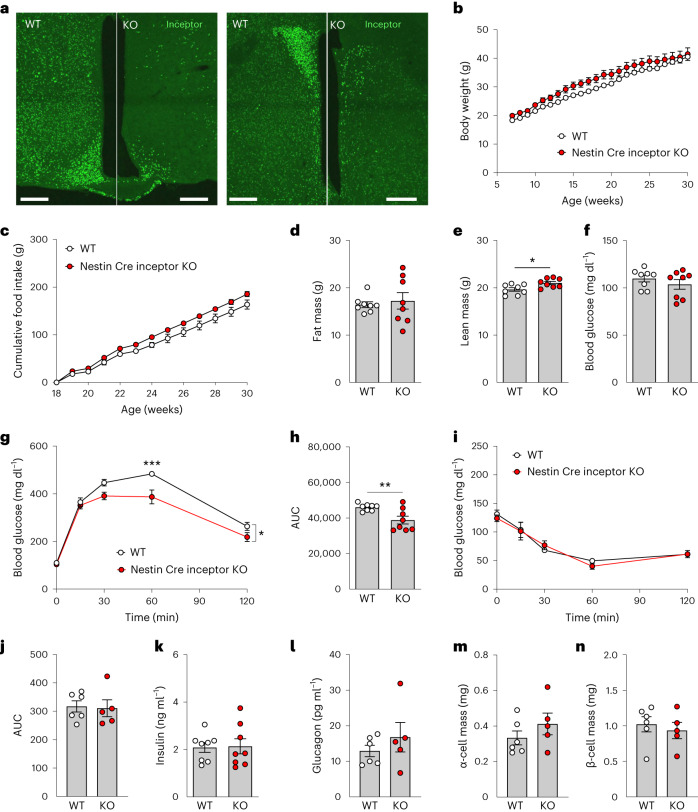
Neuronal loss of inceptor improves glucose metabolism without affecting body weight in male mice with DIO. **a**, Inceptor immunoreactivity in the hypothalamus of 11-week-old male C57BL/6J *Nestin Cre*^*+/*−^*Iir*^*wt/wt*^ (WT) and *Nestin Cre*^*+/*−^*Iir*^*flx/flx*^ (KO) mice. Scale bars, 200 μm. **b**–**f**, Body weight (**b**), cumulative food intake (**c**), as well as fat mass (**d**) and lean tissue mass (**e**) and fasting plasma levels of blood glucose (**f**), in 26-week-old male C57BL/6J WT and KO mice (*n* = 8 for each genotype) on an HFD. **g**,**h**, Intraperitoneal glucose tolerance (**g**) and AUC (**h**) in 27-week-old male C57BL/6J WT and KO mice (*n* = 8 for each genotype). **i**,**j**, Intraperitoneal insulin tolerance (**i**) and AUC (**j**) in 28-week-old male C57BL/6J mice on an HFD (*n* = 6 WT, *n* = 5 KO). **k**,**l**, Fasting levels of insulin in 27-week-old and 32-week-old C57BL/6J WT and KO mice (*n* = 8 for each group) (**k**) and fasting levels of glucagon in 27-week-old and 32-week-old C57BL/6J WT (*n* = 6) and KO mice (*n* = 5) (**l**). **m**,**n**, Pancreatic α cell (**m**) and β cell (**n**) mass in 36-week-old C57BL/6J WT and KO mice (*n* = 6 WT, *n* = 5 KO). ^*^P < 0.05, ^**^P < 0.01, ^***^P < 0.001. Data are presented as mean ± s.e.m. Data in **d**, **e**, **h**, **j**, **k**, **l**, **m** and **n** were analysed by two-sided, two-tailed Student’s *t*-test, data in **f** were analysed using the Mann–Whitney *U*-test and data in **b**, **c**, **g** and **i** were analysed by two-way ANOVA with Bonferroni’s post hoc comparison for individual time points. *P* values for group differences are *P* < 0.0169 (**e**), *P* = 0.0123 (**g**) and *P* = 0.0074 (**h**). A more detailed statistical report and the *P* values for the multiple-comparison test (**g**) are provided in Source data. Source data

**Extended Data Fig. 2:**
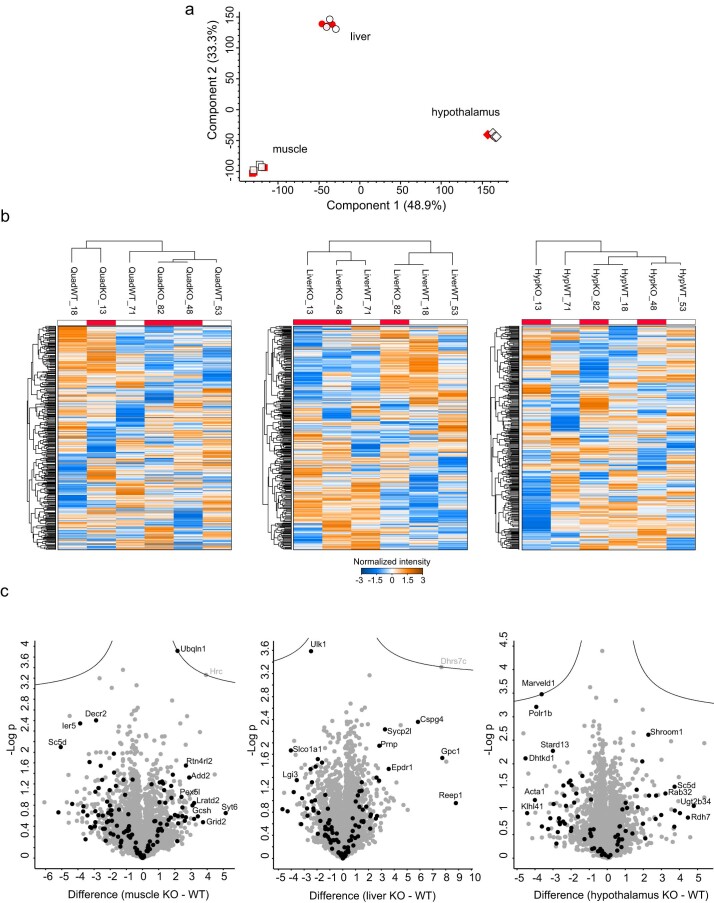
Proteome analysis of liver, muscle, and hypothalamus of neuronal inceptor KO vs. WT mice. Principle component analysis (PCA) of all tissue samples (**a**). Clustering of quadriceps muscle (left) and liver (right) samples as well as z-scored protein intensities. Bar beneath the clustering indicates genotype (WT: white, KO: red) (n = 3 each genotype) (**b**). Volcano plots of the same organs as in (**b**) ProteinGroups only found in one condition are indicated in black (**c**) (n = 3 each genotype. Source data

Based on the high expression of inceptor in the hypothalamus (Fig. 2a,b) and the well-established role of CNS INSR signalling in the control of systemic glucose homeostasis^14–16^, we next assessed whether targeted neuronal loss of inceptor affects systemic glucose metabolism in mice with DIO. Neuron-specific, inceptor-deficient mice were generated by crossing *Iir*^*flx/flx*^ mice with mice that express Cre recombinase under control of the *Nestin* promoter. Relative to WT controls (*NestinCre*^*+*^*Iir*^*wt/wt*^), neuron-specific inceptor KO mice (*NestinCre*^*+*^*Iir*^*flx/flx*^) show largely blunted inceptor immunoreactivity in the brain, including the hypothalamus (Fig. 3a). When chronically fed an HFD, Nestin Cre inceptor KO mice showed no difference in body weight (Fig. 3b), food intake (Fig. 3c) or fat mass (Fig. 3d) relative to WT controls, but slightly elevated lean tissue mass (Fig. 3e). No differences were observed in fasting levels of blood glucose (Fig. 3f), but, similar to whole-body inceptor KO mice, glucose tolerance was increased in obese Nestin Cre inceptor KO mice relative to WT controls (Fig. 3g,h). Similar to mice with global inceptor deficiency, neuronal loss of inceptor did not lead to changes in insulin sensitivity (Fig. 3i,j). MS-based analysis in muscle, liver and hypothalamus showed, likewise, no overt changes in the proteome signature between Nestin Cre inceptor KO mice and their WT controls (Extended Data Fig. 2a–c). No differences were further observed in baseline levels of plasma insulin or glucagon (Fig. 3k,l) or in α cell and β cell mass (Fig. 3m,n).

**Extended Data Fig. 3:**
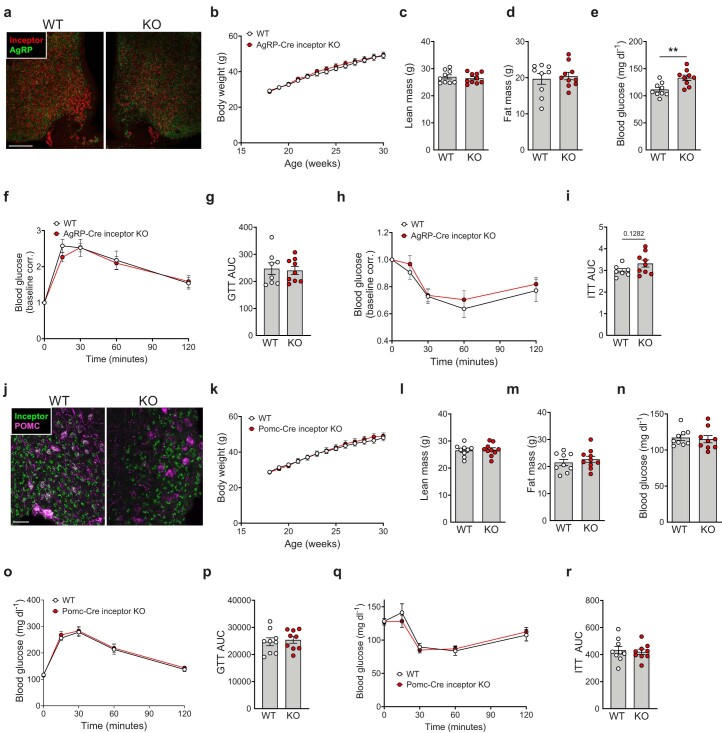
AgRP or POMC neurons do not mediate neuronal effects of inceptor on glucose homeostasis. Inceptor immunoreactivity in the hypothalamus of 20 week old C57BL/6J *Agrp-Cre*^*+/*−^ *Iir*^*wt/wt*^ (WT) and *Agrp-Cre*^*+/*−^ *Iir*^*flx/flx*^ mice (KO). Scale bar is 100 µm (**a**). **b**–**e**, Body weight (**b**), as well as lean (**c**) and fat (**d**) tissue mass and fasting glucose (**e**) in 30 week old C57BL/6J WT and KO mice (n = 9 WT, n = 10 KO). **f**,**g**, Intraperitoneal glucose tolerance (**f**) and area under the curve (AUC) (**g**) in 30 week old male C57BL/6J WT and KO mice on a HFD (n = 8 WT, n = 9 KO). **h**,**i**, Intraperitoneal insulin tolerance (**h**) and AUC (**i**) in 31 week old male mice on a HFD (n = 8 WT, n = 9 KO). **j**, Inceptor immunoreactivity in the hypothalamus of 20 week old C57BL/6J *Pomc-Cre*^*+/*−^ *Iir*^*wt/wt*^ (WT) and *Pomc-Cre*^*+/*−^ *Iir*^*flx/flx*^ (KO) mice. Scale bar is 50 µm. **k**–**n**, Body weight (**k**), as well as lean (**l**) and fat (**m**) tissue mass and fasting glucose (**n**) in 30 week old male C57BL/6J WT and KO mice on a HFD (n = 9 each genotype). **o**,**p**, Intraperitoneal glucose tolerance (**o**) and area under the curve (AUC) (**p**) in 30 week old male C57BL/6J WT and KO mice on a HFD (n = 9 each genotype). **q**,**r**, Intraperitoneal insulin tolerance (**q**) and area under the curve (AUC) (**r**) in 31 week old C57BL/6J WT and KO mice (n = 9 each genotype). Asterisks indicate ** p < 0.01. Data present mean ± SEM. Data in panel C, E, G, I, L, M, N, P, R were analyzed by students two-sided, two-tailed test. Data in panel D were analyzed using the Mann-Whitney Test. Data in panel B, F, H, K, O, Q were analyzed by two-way ANOVA with Bonferroni post-hoc comparison for individual time points. P-value for group differences is p < 0.00377 **(E)**. Corr., corrected. Source data

As a result of the demonstrated colocalization of inceptor with POMC and AgRP (Fig. 2c), both of which are implicated in regulating systemic energy metabolism^17^, we next assessed whether deletion of inceptor specifically in these neurons affects whole-body energy and glucose metabolism. Mice with deletion of inceptor in either AgRP or POMC neurons were generated by crossing *Iir*^*flx/flx*^ mice with mice that express Cre recombinase under control of either the *Agrp* or *Pomc* promotor^18,19^. Compared with *Agrp-Cre*^*+/*−^*Iir*^*wt/wt*^ (WT) controls, HFD-fed mice with *Agrp*-specific loss of inceptor (*Agrp-Cre*^*+/*−^*Iir*^*flx/flx*^) show reduced inceptor immunoreactivity in the ARC (Extended Data Fig. 3a), but without difference in body weight or body composition relative to WT controls (Extended Data Fig. 3b–d). *Agrp*-specific inceptor KO mice show slightly elevated levels of fasting blood glucose (Extended Data Fig. 3e), without differences in glucose tolerance or insulin sensitivity relative to WT controls (Extended Data Fig. 3f–i).

Deletion of inceptor in POMC neurons was verified using immunohistochemistry (IHC) by demonstrating that inceptor colocalizes with POMC in WT but not POMC inceptor KO mice (Extended Data Fig. 3j). When fed with an HFD, POMC inceptor KO mice show no differences in body weight, body composition or blood glucose relative to WT controls (Extended Data Fig. 3k–n). No changes are further observed in glucose tolerance or insulin sensitivity (Extended Data Fig. 3o–r). Collectively, these data indicate that the improved glucose metabolism that is observed in the global and neuronal inceptor KO mice is not mediated by impaired inceptor function in either POMC or AgRP neurons.

**Fig. 4:**
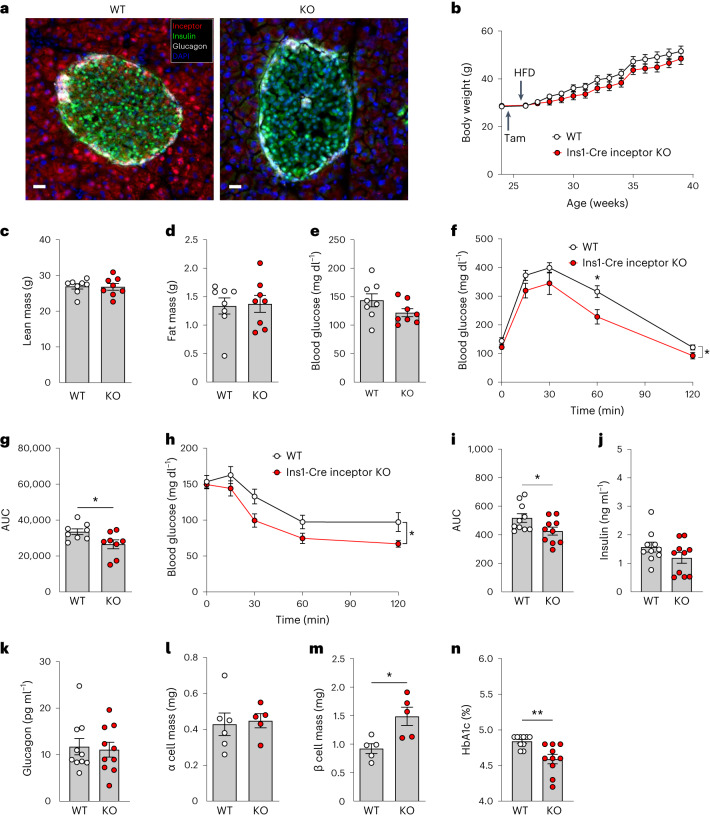
DIO male mice with adult-onset, β cell-specific inceptor deletion show improved glucose metabolism. **a**, Inceptor immunoreactivity in the pancreas of male 26-week-old *Ins1Cre*^*ERT*−*/*−^*Iir*^*flx/flx*^ (WT) and *Ins1Cre*^*ERT+/*−^*Iir*^*flx/flx*^ (KO) C57BL/6J mice. Scale bars, 20 μm. **b**–**e**, Body weight (**b**), as well as lean (**c**) and fat (**d**) tissue mass and fasting glucose (**e**) in 30-week-old male C57BL/6J WT and KO mice (*n* = 8 for each genotype). Tam, Tamoxifen. **f**,**g**, Intraperitoneal glucose tolerance (**f**) and AUC (**g**) in 18-week-old male C57BL/6J WT and KO mice on an HFD (*n* = 8 for each genotype). **h**,**i**, Intraperitoneal insulin tolerance (**h**) and AUC (**i**) in 18-week-old male C57BL/6J WT and KO mice on an HFD (*n* = 10 for each genotype). **j**,**k**, Fasting levels of plasma insulin (*n* = 10 for each genotype) (**j**) and glucagon (**k**) (*n* = 10 for each genotype) in 22-week-old male C57BL/6J WT and KO mice. **l**,**m**, Pancreatic α cell mass (**l**; n = 5 each genotype) and β cell mass (**m**; *n* = 6 WT, *n* = 5 KO) in 26-week-old male C57BL/6J WT and KO mice. **n**, Whole-blood HbA1c in 18-week-old male C57BL/6J WT and KO mice (*n* = 10 mice per genotype). ^*^P < 0.05, ^**^P < 0.01. Data are presented as mean ± s.e.m. Data in **c**, **d**, **e**, **g**, **l**, **j**, **k**, **l**, **m** and **n** were analysed by two-sided, two-tailed Student’s *t*-test, data in **b**, **f** and **h** were analysed by two-way ANOVA with Bonferroni’s post hoc comparison for individual time points and data in **n** were analysed using Mann–Whitney *U*-test. *P* values for group differences are *P* < 0.0426 (**f**), *P* = 0.0322 (**g**), *P* = 0.0382 (**h**), *P* = 0.0379 (**i**), *P* = 0.0142 (**m**) and *P* = 0.002 (**n**). A more detailed statistical report and the *P* value for the multiple-comparison test (**f**) are provided in Source data. Source data

**Extended Data Fig. 4:**
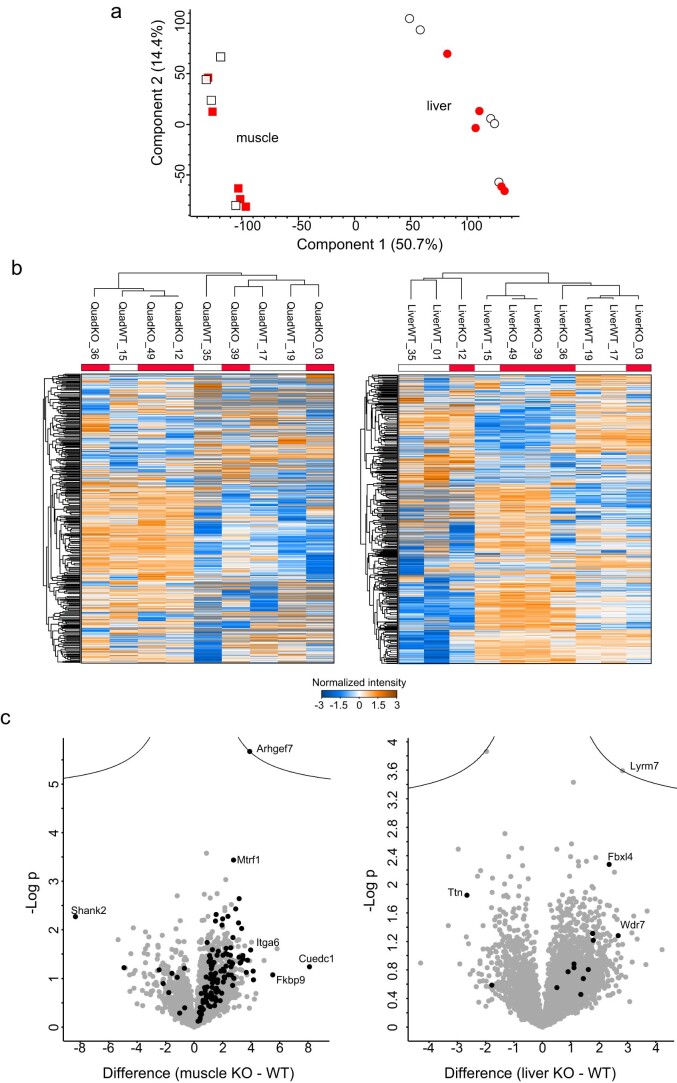
Proteome analysis of liver and muscle of adult-onset *β*-cell-specific inceptor KO vs. WT mice. Principle component analysis (PCA) of all tissue samples (**a**). Clustering of quadriceps muscle (left) and liver (right) samples as well as z-scored protein intensities. Bar beneath the clustering indicates genotype (WT: white, KO: red) (n = 4 KO, n = 5 WT for muscle, n = 5 each genotype for liver) (**b**). Volcano plots of the same organs as in (**b**) ProteinGroups only found in one condition are indicated in black (**c**) (n = 5 each genotype). Source data

Based on the demonstrated role of inceptor to regulate islet glucose metabolism in lean mice^7^, we next assessed whether targeted deletion of inceptor in adult pancreatic β cells improves glycaemic control also under conditions of DIO. Adult-onset, β cell, inceptor-deficient mice were generated by crossing *Iir*^*flx/flx*^ mice to mice that express Cre recombinase in a tamoxifen-inducible manner under control of the *Ins1* promoter. Tamoxifen induction of β cell inceptor KO was initiated at the age of 26 weeks and was verified by IHC (Fig. 4a). When chronically fed with an HFD, adult-onset β cell inceptor KO mice (*Ins1Cre*^*ERT+/*−^*Iir*^*flx/flx*^) show no differences in body weight (Fig. 4b), body composition (Fig. 4c,d) or blood glucose (Fig. 4e) relative to WT controls (*Ins1Cre*^*ERT*−*/*−^ *Iir*^*flx/flx*^), but display improved glucose tolerance (Fig. 4f,g) and enhanced insulin sensitivity (Fig. 4h,i). Furthermore, mice with adult-onset β cell inceptor deletion show no significant difference in fasted plasma levels of insulin (Fig. 4j) and no changes in plasma glucagon or α cell mass (Fig. 4k,l), but slightly increased β cell mass (Fig. 4m), and a significant improvement in HbA1c levels (Fig. 4n). No overt changes were observed on the proteome of either the liver or the muscle (Extended Data Fig. 4a–c).

In summary, our data show that mice with DIO and perturbed inceptor function in either the whole body or specifically in CNS neurons or the pancreatic β cells are protected from diet-induced glucose intolerance. Preservation of glucose tolerance in obese global inceptor-deficient mice is not related to changes in body weight, food intake or insulin sensitivity, but is associated with enhanced glucose-stimulated insulin secretion. Furthermore, it was shown that inceptor immunoreactivity is high in hypothalamic areas regulating energy and glucose metabolism and that inceptor colocalizes with neuronal markers (including POMC and AgRP), but not with markers indicative of astrocytes, neuroglia or microglia. In striking contrast to mice with neuronal loss of inceptor, we have shown that targeted deletion of inceptor in either POMC or AgRP neurons does not protect mice with DIO from glucose intolerance. Collectively, our data show that, under conditions of DIO, inceptor functions in both the CNS and pancreatic β cells regulate whole-body glucose metabolism, without major effects on body weight or body composition. Furthermore, we identified CNS neurons as the primary source of inceptor expression in the brain, and exclude a role of inceptor function in POMC or AgRP neurons in the regulation of glucose metabolism. Our data are consistent with our previous report showing that inceptor plays a key role in regulating islet insulin action in lean normoglycaemic mice^7^ and supports the hypothesis that inhibition of inceptor to sensitize INSR/IGF1R action may be a promising pharmacological target in the context of diet-induced impairments of glucose homeostasis, in particular with regard to β cell mass and health, with the potential to avoid β cell failure and slow diabetes progression. Notably, similar to the whole-body inceptor KO, we show that pan-neuronal loss of inceptor improves glucose metabolism in mice with DIO without affecting body weight or body composition, suggesting that central loss of inceptor does not fully restore central insulin sensitivity under conditions of DIO, but acts on glucose homeostasis by a different mechanism^14,17,20^. The observation that CNS loss of inceptor improves systemic glucose tolerance in mice with DIO is consistent with previous reports that link central insulin action to control of peripheral glucose metabolism^20,21^, as well as a recently published paper, showing that inceptor is co-expressed with INSR and IGF1R in neurons and that knockdown of inceptor increases insulin sensitivity in ex vivo neuron cultures^22^. However, we show that these central effects are not mediated via inceptor action in AgRP or POMC neurons. Nevertheless, underlining the potential relevance of inceptor inhibition for the treatment of diabetes, we have shown in the present study that targeted adult-onset loss of inceptor in pancreatic β cells renders mice with DIO less susceptible to diet-induced impairment of glucose metabolism, primarily by improving β cell health and function. These data are consistent with reports indicating the necessity of early interventions to maintain adequate β cell glucose sensitivity^21^. In line with this notion, early intensive insulin therapy has been demonstrated to recover and maintain β cell maintenance and function, and to slow down progression of T2D^23^, but with the potential side effects of inducing hypoglycaemia and body weight gain^24–28^. The beneficial effects of early insulin are thought to be primarily through reduced glucotoxicity, which undermines β cell function. We have recently shown, in streptozotocin-induced diabetic mice, that long-term hyperglycaemia and β cell dysfunction/dedifferentiation can be counteracted by intense prolonged insulin therapy^6^. It is interesting that single-cell RNA sequencing data of islet cells revealed that INSR signalling by exogenous insulin supplementation triggered β cell redifferentiation and regeneration for diabetes remission, again confirming that increased insulin action has direct beneficial effects on β cell health and function^6^. Hence, in contrast to intense, early onset insulin therapy, inceptor-mediated improvement of β cell function may offer the potential to counteract the detrimental glucometabolic effects of DIO without the risk of causing hypoglycaemia or unwanted body weight gain.

## Methods

### Animals and housing conditions

Animal experiments were performed in accordance with the Animal Protection Law of the European Union and with the permission of the government of upper Bavaria (Regierung von Oberbayern), Germany. Only male mice were used in the studies, because female mice are largely resistant to DIO and alterations in glucose metabolism when chronically fed with an HFD^29^. Mice were fed a 58% high-fat, high-sucrose diet (Research Diets, catalogue no. D12331) and were group housed on a 12:12 h light:dark cycle at 22 ± 1 °C, 45–55% humidity and with free access to food and water unless indicated otherwise. C57BL/6J mice were provided by Janvier Labs. Transgenic mice were generated on a C57BL/6J background as described. *Nestin-Cre* mice (catalogue no. 003771)^30^, *Ins1-Cre*^*ERT*^ mice (MGI: catalogue no. 4410453)^31^, *Agrp*-Cre mice (catalogue no. 012899)^19^ and *Pomc*-Cre mice (catalogue no. 005965)^18^ were purchased from the Jackson Laboratories.

### Animal metabolic studies

Food consumption was measured per cage in double-housed, or temporally single-housed, mice. Body composition (fat and lean mass) was measured using quantitative nuclear magnetic resonance technology (EchoMRI). For analysis of glucose tolerance or insulin tolerance, mice were fasted for 6 h, followed by intraperitoneal administration of 1.5–2 g kg^−1^ of glucose or 0.5–1 U kg−1 of insulin (Humalog, Eli Lilly). Plasma levels of insulin and glucagon were measured by ELISA (Crystal Chem, catalogue nos. 90082, 8151890050) following the manufacturer’s instructions.

### IHC

For brain images, mice were perfused with phosphate-buffered saline followed by 4% paraformaldehyde (Thermo Fisher Scientific), postfixed for 1 d, equilibrated in 30% sucrose for 1 d and sectioned on a cryostat (Leica Biosystems) at 30–40 μm. Staining was performed in 0.1 M tris-buffered solution with 0.25% gelatine (0.25%) and 0.5% Triton X-100. For the pancreas, dissected pancreatic cryo-samples were cryosectioned at 12 µm and briefly fixed in formalin (formalin 10% neutral buffered, Sigma-Aldrich, catalogue no. HT501128). Pancreatic islets were analysed by triple staining for insulin, glucagon and inceptor. A complete list of primary and secondary antibodies and dilutions is provided in Supplementary Information. Nuclei were identified with Hoechst 33342. Stained slides were digitized with an AxioScan 7 digital slide scanner (Zeiss, ZEN Blue v.3.5) equipped with a ×20 magnification objective.

### IHC for α cell and β cell volume and islet size

Dissected pancreata were fixed in formalin (formalin 10% neutral buffered) for 24 h at room temperature and processed for paraffin embedding (Tissue Tec VIP.6, Sakura Europe). Paraffinized pancreata were cross-sectioned into three to four parallel, equidistant slices per case. Maintaining their orientation, the tissue slices were vertically embedded in paraffin. After costaining for insulin and glucagon, nuclei were labelled with Hoechst 33342 (Thermo Fisher Scientific, catalogue no. H13997, 5 µg ml^−1^). A complete list of primary and secondary antibodies and dilutions is provided in Supplementary Information. The stained tissue sections were scanned with an AxioScan 7 digital slide scanner (Zeiss, ZEN Blue v.3.5) equipped with a ×20 magnification objective. Quantification of insulin or glucagon expression cells was performed on the entire tissue sections by using image analysis software Visiopharm. The insulin- or glucagon-expressing cells were classified automatically using the fluorescence intensity of each hormone. The β cell volume (mg) was calculated by multiplying the detected relative insulin-positive cell area by total pancreatic weight. The α cell volume (mg) was similarly calculated based on the detected glucagon-positive cell area. The area of the pancreatic islet was calculated based on the insulin- and glucagon-positive area.

### Proteomics sample preparation

Tissues were disrupted using a tissuelyser (QIAGEN), heated for 5 min at 95 °C and 1,000 r.p.m. in 2% sodium deoxycholate (SDC) buffer (2% SDC, 100 mM Tris-HCl, pH 8.5) and sonicated (Diagenode Bioruptor, 15 × 30 s at high intensity); for liver and muscle, each step was done twice. After centrifugation, the protein concentration of the supernatant was determined using the BCA Protein Assay (Thermo Fisher Scientific, catalogue no. 23225). Protein, 25 µg per sample, was reduced, alkylated with 10 mM tris(2-carboxyethyl)phosphine and 40 mM chloroacetamide at 40 °C in the dark for 10 min and then digested overnight (37 °C, 1,000 r.p.m.) with a 1:50 ratio (protein:enzyme) of trypsin (Sigma-Aldrich, catalogue no. t6567) and LysC (Wako, catalogue no. 129-02541). On the next day, peptides were acidified and loaded on to activated triple layer styrene divinylbenzene-reversed-phase-sulfonated STAGE tips (SDB-RPS; 3M Empore). Peptides were washed with 100 µl of ethylacetate, 1% trifluoroacetic acid (TFA), 100 µl of 30% methanol, 1% TFA and 150 µl of 0.2% TFA and eluted with 60 µl of elution buffer (80% acetonitrile (ACN), 5% NH_4_OH). Peptides were lyophilized and dissolved in 10 µl of MS-loading buffer (2% ACN, 0.1% TFA).

### LC–MS/MS analysis

Liquid chromatography–tandem MS (LC–MS/MS) analysis of 500 ng of peptides was performed on an Orbitrap Exploris 480 (Thermo Fisher Scientific) equipped with a nano-electrospray ion source and FAIMS (CV50), coupled to an EASY-nLC 1200 high-performance LC (HPLC) (all Thermo Fisher Scientific). The LC was equipped with a 50-cm column packed in-house with ReproSil-Pur C18-AQ 1.9-μm resin (Dr. Maisch HPLC GmbH). The peptides were separated at 60 °C over 1 h by reversed-phase chromatography using a binary buffer system consisting of buffer A (0.1 formic acid) and buffer B (80% ACN, 0.1% formic acid). Starting with 5% buffer B, this fraction was increased stepwise to 45% over 45 min, followed by a wash-out at 95%, all at a constant flow rate of 300 nl min^−1^. After using electrospray ionization to transfer the peptides to the mass spectrometer, a data-independent method was used for measurement. For this, one ms1 scan (300–1,650 *m*/*z*, maximum ion fill time of 45 ms, normalized automatic gain control (AGC) target = 300%, *R* = 120.000 at 200 *m*/*z*) was followed by 66-ms^2^ fragment scans of unequally spaced windows (fill time = 22 ms, normalized AGC target = 1,000%, normalized higher-energy collision dissociation collision energy = 30%, *R* = 15.000)

### Data analysis for proteomics

DIA raw files were demultiplexed with Spectronaut HTRMS converter and analysed with Spectronaut (v.18.1.230626.50606). Analysis of the resulting protein file was performed in Perseus (v.1.6.15.0) using standard parameters if not stated otherwise. Proteomic samples that showed a considerably lower protein group count than others in the same tissue were excluded. The log_2_(transformed) values with <4 s.d. of the average distribution were considered missing values. Thereafter protein groups were filtered for a minimum of three values in at least one sample group (tissue + genotype) and missing values imputed with a normal distribution (downshift 1.8 stdvs, width 0.3).

### Western blotting

Protein samples were prepared as described for proteomics. Protein, 20 μg, was run on a sodium dodecylsulfate–polyacrylamide gel electrophoresis gradient gel (4–20%) in a Protean System (BioRad) in 25 mM Tris, 192 mM glycine, pH 8.3 buffer. Samples were transferred to poly(vinylidene fluoride) membranes using a BioRad Turboblot system, stained with primary anti-Akt (Cell Signaling, catalogue no. 2920, 1:1,000) and p-AKT S473 (Cell Signaling, catalogue no. 4060, 1:1,000) antibodies, and secondary antibodies (BioRad, Starbright 700, anti-rabbit Alexa Fluor-790 anti-mouse (Abcam, catalogue no. ab175781), both 1:6,000) and detected on a BioRad ChemiDoc system with a fluorescence detection module.

### Image analysis

Images were obtained as serial *z*-stacks using a Leica SP5 or LSM 880 Airyscan microscope (Zeiss, ZEN Black v.2.3) as tile scans (10% overlap). Final images were analysed and processed with ImageJ (v.2.14; Java 1.8). Inceptor colocalization was performed with manual blind counting. The mean fluorescence intensity in hypothalamic target regions was measured on unaltered images and corrected for background fluorescence using Fiji 1.0 (ImageJ). When possible, quantifications were performed on several sections spanning the medial ARC nucleus and PVN and averaged.

### Gene expression analysis

RNA was extracted using RNeasy Mini Kits (QIAGEN). Complementary DNA was generated with QuantiTech reverse transcription kit (QIAGEN). Quantitative PCR was performed with a ViiA 7 PCR System (Applied Biosystems) using the TaqMan probes *Hprt* (Mm01545399_m1) and *inceptor* (Mm00478295_m1) from Thermo Fisher Scientific. Target gene expression was normalized to reference gene *Hprt*, by ΔΔ*C*^*T*^.

### Statistics

For animal studies, sample sizes were calculated based on a power analysis assuming that a ≥5-g difference in body weight between genotypes can be assessed with a power of ≥75% when using a two-sided statistical test under the assumption of an s.d. of 3.5 and an α level of 0.05. Statistical analyses were performed two sided, using the statistical tools implemented in GraphPad Prism. Before statistical analysis, data were tested for normal distribution using the Kolmogorow–Smirnow test with Lillifors correction. In the case of non-normal distribution, data were analysed using either the Mann–Whitney *U*-test or the Kruskal–Wallace test. In the case of normal distribution, data were analysed using an unpaired (two-sided), two-tailed Student’s *t*-test, one-way analysis of variance (ANOVA) or two-way ANOVA, followed by an appropriate post hoc multiple-comparison test as indicated in the figure legends. *P* ≤ 0.05 was considered statistically significant. Animals were either randomly assigned into treatment groups or grouped based on their genotype (WT or KO). At the study’s start, only age-matched mice were included in the studies. No other covariates were controlled for. Analyses of glucose and insulin tolerance were performed by experienced research assistants who did not know prior treatment conditions. Ex vivo studies were performed in ID-coded vials without statement of treatment on the vials and with most, but not all, investigators being blinded to the underlying genotypes and treatment conditions. No data were excluded from the analysis unless scientific (for example, outlier identified by the Grubbs test for outlier) or animal welfare reasons (for example, injury due to fighting) demanded exclusion. Outliers are stated in Source data.

### Reporting summary

Further information on research design is available in the Nature Portfolio Reporting Summary linked to this article.

### Supplementary information

Supplementary Information List of used antibodies and dilutions.

Reporting Summary

### Source data

Source Data Fig. 1 Statistical source data for Fig. 1.

Source Data Fig. 2 Statistical source data for Fig. 2.

Source Data Fig. 3 Statistical source data for Fig. 3.

Source Data Fig. 4 Statistical source data for Fig. 4.

Source Data Extended Data Fig. 1 Statistical source data for Extended Data Fig. 1.

Source Data Extended Data Fig. 2 Statistical source data for Extended Data Fig. 2.

Source Data Extended Data Fig. 3 Statistical source data for Extended Data Fig. 3.

Source Data Extended Data Fig. 4 Statistical source data for Extended Data Fig. 4.

Source Data Fig. 1 Original data for Fig. 1a.

Source Data Fig. 2 Original data for Fig. 2a–d.

Source Data Fig. 3 Original data for Fig. 3a.

Source Data Fig. 4 Original data for Fig. 4a.

Source Data Extended Data Fig. 3 Original data for Extended Data Fig. 3a,j.

Source Data Fig. 1 Unprocessed western blots for Fig. 1k.

## Data Availability

The data used for the statistical analysis are available in Source data, along with the GraphPad Prism-derived report on the statistical analysis as appropriate. The statistical report contains the mean difference between the treatment groups, the 95% confidence intervals, the significance summary and the exact *P* values (unless *P* < 0.0001). The MS proteomics data have been deposited to the ProteomeXchange Consortium via the PRIDE^32^ partner repository with the accession no. PXD046256. Raw images are included in Source data, with the exception of the histology pictures in Figs. 1m,n, 3m,n and 4l,m, which were too large for public repositories and are available from the corresponding authors upon request. Source data are provided with this paper.

## Code availability

No customized code was used for the present study.
