# Supplementary material for: Global, neuronal or β cell-specific deletion of inceptor improves glucose homeostasis in male mice with diet-induced obesity
Source: Nat Metab. 2024 Feb 28;6(3):448–57. doi: 10.1038/s42255-024-00991-3 (PMC10963260; doi:10.1038/s42255-024-00991-3)
Supplement: Supplementary file 1 — List of used antibodies and dilutions. [file 42255_2024_991_MOESM1_ESM.pdf]

nature metabolism

Letter https://doi.org/10.1038/s42255-024-00991-3

Global, neuronal or β cell-specific deletion of inceptor improves glucose homeostasis in male mice with diet-induced obesity

In the format provided by the authors and unedited

antibody	manufacturer, catalogue #	dilution
rat monoclonal anti-Inceptor 2G6	produced in house by MAB core facility Helmholtz	1:200
rabbit anti-Pomc precursor	Phoenix Pharmaceuticals, H-029-30	1:1000
goat anti-Agrp	R&D systems, AF634	1:100
goat anti-GFAP	Sigma, SAB2500462	1:1000
mouse anti-Neun, A60	Merck, MAB377	1:500
goat anti-Iba1	Abcam, ab107519	1:1000
rabbit anti-insulin	Cell Signalling, #3014	1:1000
guinea pig anti-Glucagon	Takara Bio, #M182	1:2500, 1:3000, 1:1500
mouse anti Akt	Cell Signaling, #2920	1:1000
rabbit anti p-AKT S473	Cell Signaling, #4060	1:1000
anti-insulin	Cell Signaling, #3014	1:800, 1:100
goat Anti-Rabbit IgG StarBright Blue 700	BioRad #12004161	1:6000
donkey Anti-Mouse IgG H&L (Alexa Fluor® 790)	Abcam, ab175782	1:6000
anti-rabbit Alexa546 ; Dilution1:2,000)	Invitrogen, #A10040	1:2000
goat anti-guinea pig AF555 , Dilution 1:2000)	Invitrogen, #A21435	1:2000
AlexaFluor750-conjugated goat anti-rabbit	Invitrogen, #A21039	1:100
Donkey anti-Rat IgG (H+L) Alexa Fluor™ Plus 488	Thermo Fisher, A48269	1:800
Donkey anti-Rat IgG (H+L) Alexa Fluor™ 568	Thermo Fisher, A78946	1:800
Donkey anti-Goat IgG (H+L) Alexa Fluor™ Plus 488	Thermo Fisher, A32814	1:800
Donkey anti-Goat IgG (H+L) Alexa Fluor™ Plus 568	Thermo Fisher, A11057	1:800
Donkey anti-Goat IgG (H+L) Alexa Fluor™ 647	Thermo Fisher, A21447	1:800
Donkey anti-Rabbit IgG (H+L) Alexa Fluor™ 488	Thermo Fisher, A21206	1:800
Donkey anti-Mouse IgG (H+L) Alexa Fluor™ 647	Thermo Fisher, A31571	1:800
Donkey anti-Rat IgG (H+L) Alexa Fluor™ 488	Thermo Fisher, A21208	1:800
Donkey anti-Rat IgG (H+L) Alexa Fluor™ Plus 555	Thermo Fisher, A48270	1:800
