# Supplementary material for: Global, neuronal or β cell-specific deletion of inceptor improves glucose homeostasis in male mice with diet-induced obesity
Source: Nat Metab. 2024 Feb 28;6(3):448–57. doi: 10.1038/s42255-024-00991-3 (PMC10963260; doi:10.1038/s42255-024-00991-3)
Supplement: Supplementary file 2 — Reporting Summary [file 42255_2024_991_MOESM2_ESM.pdf]

nature portfolio

Corresponding author(s): Timo D. Müller, Heiko Lickert

Last updated by author(s): Jan 5, 2024

Reporting Summary

Nature Portfolio wishes to improve the reproducibility of the work that we publish. This form provides structure for consistency and transparency in reporting. For further information on Nature Portfolio policies, see our Editorial Policies and the Editorial Policy Checklist.

Statistics

For all statistical analyses, confirm that the following items are present in the figure legend, table legend, main text, or Methods section.

n/a	Confirmed	
☐	☒	The exact sample size (*n*) for each experimental group/condition, given as a discrete number and unit of measurement
☐	☒	A statement on whether measurements were taken from distinct samples or whether the same sample was measured repeatedly
☐	☒	The statistical test(s) used AND whether they are one- or two-sided<br>*Only common tests should be described solely by name; describe more complex techniques in the Methods section.*
☐	☒	A description of all covariates tested
☐	☒	A description of any assumptions or corrections, such as tests of normality and adjustment for multiple comparisons
☐	☒	A full description of the statistical parameters including central tendency (e.g. means) or other basic estimates (e.g. regression coefficient) AND variation (e.g. standard deviation) or associated estimates of uncertainty (e.g. confidence intervals)
☐	☒	For null hypothesis testing, the test statistic (e.g. *F*, *t*, *r*) with confidence intervals, effect sizes, degrees of freedom and *P* value noted<br>*Give P values as exact values whenever suitable.*
☒	☐	For Bayesian analysis, information on the choice of priors and Markov chain Monte Carlo settings
☒	☐	For hierarchical and complex designs, identification of the appropriate level for tests and full reporting of outcomes
☒	☐	Estimates of effect sizes (e.g. Cohen's *d*, Pearson's *r*), indicating how they were calculated

Our web collection on statistics for biologists contains articles on many of the points above.

Software and code

Policy information about availability of computer code

Data collection	Images were obtained as serial Z-stacks using a Leica SP5.<br>Stained slides were digitized with an AxioScan 7 digital slide scanner.<br>Western Blots were imaged on a Biorad Chemidoc analyser.
Data analysis	Statistical analyses were performed using GraphPad Prism version 9. Histological analysis were performed using Visiopharm (v. 2018.9).<br>Western Blot Densitometry analysis was performed using Biorad ImageLab 6.0.1

For manuscripts utilizing custom algorithms or software that are central to the research but not yet described in published literature, software must be made available to editors and reviewers. We strongly encourage code deposition in a community repository (e.g. GitHub). See the Nature Portfolio guidelines for submitting code & software for further information.

Data

Policy information about availability of data

All manuscripts must include a data availability statement. This statement should provide the following information, where applicable:

- Accession codes, unique identifiers, or web links for publicly available datasets
- A description of any restrictions on data availability
- For clinical datasets or third party data, please ensure that the statement adheres to our policy

The data used for the statistical analysis are available in the data source file, along with the GraphPad Prizm-derived report on the statistical analysis as appropriate. The statistical report contains the mean difference between the treatment groups, the 95% confidence intervals, the significance summary, and the exact P values (unless P < 0.0001). The mass spectrometry proteomics data have been deposited to the ProteomeXchange Consortium via the PRIDE31 partner repository with the

dataset identifier PXD046256. Raw images are included in the data source files, with exception of the histology pictures in Figure 1M,N, Figure 2M,N, and Figure 4L,M, which were too large for public repositories and are available from the corresponding author upon request.

Field-specific reporting

Please select the one below that is the best fit for your research. If you are not sure, read the appropriate sections before making your selection.

☒ Life sciences ☐ Behavioural & social sciences ☐ Ecological, evolutionary & environmental sciences

For a reference copy of the document with all sections, see nature.com/documents/nr-reporting-summary-flat.pdf

Life sciences study design

All studies must disclose on these points even when the disclosure is negative.

Sample size	For animal studies, sample sizes were calculated based on a power analysis assuming that a greater or equal (>/=) 5 g difference in body weight between genotypes can be assessed with a power of >/= 75% when using a 2-sided statistical test under the assumption of a standard deviation of 3.5 and an alpha level of 0.05.
Data exclusions	No data were excluded from the analysis unless scientific (e.g. significant outlier identified by the Grubbs test for outlier) or animal welfare reasons (e.g. injury due to fighting) demanded exclusion. Outliers are stated in the data source file.
Replication	In vivo and ex vivo data were obtained in independent biological replicates as indicated in the figure legends.
Randomization	Animals were either randomly assigned into treatment groups, or were grouped based on their genotype (WT or KO). At study start, only age-matched mice were included in the studies. There were no other covariats controlled.
Blinding	For in vivo studies, drugs were aliquoted by a lead scientist in number-coded vials and most, but not all, handling investigators were blinded to the treatment condition. Analyses of glucose and insulin tolerance were performed by experienced research assistants which did not know prior treatment conditions. Ex vivo studies were performed in ID coded vials without statement of treatment on the vials. Ex vivo studies were performed in ID coded vials, and with with most, but not all investigators, being blinded to the underlying genotypes.

Reporting for specific materials, systems and methods

We require information from authors about some types of materials, experimental systems and methods used in many studies. Here, indicate whether each material, system or method listed is relevant to your study. If you are not sure if a list item applies to your research, read the appropriate section before selecting a response.

Materials & experimental systems

n/a	Involved in the study	
☐	☒	Antibodies
☒	☐	Eukaryotic cell lines
☒	☐	Palaeontology and archaeology
☐	☒	Animals and other organisms
☒	☐	Human research participants
☒	☐	Clinical data
☒	☐	Dual use research of concern

Methods

n/a	Involved in the study	
☒	☐	ChIP-seq
☒	☐	Flow cytometry
☒	☐	MRI-based neuroimaging

Antibodies

Antibodies used

rat monoclonal anti-Inceptor 2G6 produced in house by MAB core facility Helmholtz 1:200
rabbit anti-Pomc precursor Phoenix Pharmaceuticals, H-029-30 1:1000
goat anti-Agrp R&D systems, AF634 1:100
goat anti-GFAP Sigma, SAB2500462 1:1000
mouse anti-Neun, A60 Merck, MAB377 1:500
goat anti-Iba1 Abcam, ab107519 1:1000
rabbit anti-insulin Cell Signalling, #3014 1:1000
guinea pig anti-Glucagon Takara Bio, #M182 1:2500, 1:3000, 1:1500
mouse anti Akt Cell Signaling, #2920 1:1000
rabbit anti p-AKT S473 Cell Signaling, #4060 1:1000
anti-insulin Cell Signaling, #3014 1:800, 1:100
goat Anti-Rabbit IgG StarBright Blue 700 BioRad #12004161 1:6000
donkey Anti-Mouse IgG H&L (Alexa Fluor® 790) Abcam, ab175782 1:6000
anti-rabbit Alexa546 ; Dilution1:2,000) Invitrogen, #A10040 1:2000
goat anti-guinea pig AF555 , Dilution 1:2000) Invitrogen, #A21435 1:2000
AlexaFluor750-conjugated goat anti-rabbit Invitrogen, #A21039 1:100

Donkey anti-Rat IgG (H+L) Alexa Fluor™ Plus 488 Thermo Fisher, A48269 1:800
Donkey anti-Rat IgG (H+L) Alexa Fluor™ 568 Thermo Fisher, A78946 1:800
Donkey anti-Goat IgG (H+L) Alexa Fluor™ Plus 488 Thermo Fisher, A32814 1:800
Donkey anti-Goat IgG (H+L) Alexa Fluor™ Plus 568 Thermo Fisher, A11057 1:800
Donkey anti-Goat IgG (H+L) Alexa Fluor™ 647 Thermo Fisher, A21447 1:800
Donkey anti-Rabbit IgG (H+L) Alexa Fluor™ 488 Thermo Fisher, A21206 1:800
Donkey anti-Mouse IgG (H+L) Alexa Fluor™ 647 Thermo Fisher, A31571 1:800
Donkey anti-Rat IgG (H+L) Alexa Fluor™ 488 Thermo Fisher, A21208 1:800
Donkey anti-Rat IgG (H+L) Alexa Fluor™ Plus 555 Thermo Fisher, A48270 1:800

Validation

Monoclonal anti-inceptor 2G6 (produced in house by MAB core facility Helmholtz, 1:200) was validated according to Ansarullah et al. 2021, against WT and KO C57Bl/6 mice on Western Blot and immunohistochemistry applications.

Rabbit anti-Pomc precursor (Phoenix Pharmaceuticals, 1:1000) is a validated polyclonal antibody used in 21 publications. Applications include Immunohistochemistry and Western Blot. The antibody reacts with POMC from mouse and rat. Based on the manufacturer, the antibody was validated on mouse and rat brain slices.

Goat anti-Agrp (R&D systems, AF634, 1:100) is a validated polyclonal antibody used in 181 publications with human samples, and 11604 publications with mouse samples. It is affinity purified, detects mouse Agrp in direct ELISAs and is recommended for immunohistochemistry.

Goat anti-GFAP (Sigma, SAB2500462, 1:1000) is a validated polyclonal antibody used in 18 publications. Goat polyclonal anti-GFAP antibody reacts with rat, canine, human, and mouse glial fibrillary acidic proteins. It is recommended for detection and quantitation of GFAP by Western blotting and immunohistochemical (IHC) techniques.

Mouse anti-Neun (Merck, MAB377, 1:500) Anti-NeuN Antibody, clone A60 detects level of NeuN and has been published and validated for use in FC, IC, IF, IH, IH(P), IP and WB. It has been used in over 5000 publications. MILLIPORE's exclusive monoclonal antibody to vertebrate neuron-specific nuclear protein called NeuN (or Neuronal Nuclei) reacts with most neuronal cell types throughout the nervous system of mice including cerebellum, cerebral cortex, hippocampus, thalamus, spinal cord and neurons in the peripheral nervous system including dorsal root ganglia, sympathetic chain ganglia and enteric ganglia. Developmentally, immunoreactivity is first observed shortly after neurons have become postmitotic, no staining has been observed in proliferative zones. The immunohistochemical staining is primarily localized in the nucleus of the neurons with lighter staining in the cytoplasm. The few cell types not reactive with MAB377 include Purkinje, mitral and photoreceptor cells. The antibody is an excellent marker for neurons in primary cultures and in retinoic acid-stimulated P19 cells. It is also useful for identifying neurons in transplants.

Goat anti-Iba1 (Abcam ab107519, 1:1000) is a validated polyclonal antibody used in 137 publications. It is affinity purified and detects rat Iba1 in liver, kidney and brain sections. It is recommended for IHC applications.

Rabbit anti Insulin (C27C9) Rabbit mAb #3014 (Cell Signaling, 1:300) is a validated monoclonal antibody used in 119 publications. Applications include Immunohistochemistry, ChIP, and Immunoprecipitation. The antibody reacts with Insulin from human, mouse and rat. Based on the manufacturer, the antibody meets all of the quality control standards defined by Cell Signaling Technology, Inc. Validations include Immunohistochemical analysis of paraffin-embedded human pancreas, showing the staining of β cells, using Insulin (C27C9) Rabbit mAb. The antibody is further reported to show very clear staining at 1:2000 with no background staining in primary human cells.

Anti-glucagon (Takara, M182) is a polyclonal guinea pig antibody, supplied by Takara Bio, cited in 24 publications. Applications used include IHC and CLARITY. Its a Guinea Pig polyclonal antibody raised against the peptide [HSQGTFTSDYSKYLDSRRAQDFVQWLMNT] of human Glucagon conjugated with KLH as an immunogen.The lyophilized antibody was dissolved in 50 µl of specifed water. The antibody dilutions were applied for ELISA assay by colorimetric detection using a microtiter plate immobilized with human Glucagon peptide. The expected antibody titration was obtained. Manufacturing Control: Purification: Guinea Pig serum IgG was purified by affinity column chromatography, dissolved in 10 mM PBS, pH 7.4, containing 1.0% bovine serum albumin, and then lyophilized.

Mouse anti Akt (Cell Signaling #2920, 1:1000) Akt (pan) (40D4) Mouse mAb #2920 is a validated monoclonal antibody with 1376 publications. It reacts with mouse, human, rat and monkey tissues. It is affinity purified and suitable for Western Blot, immunohistochemistry, IP.

Rabbit anti p-AKT S473 (Cell Signaling #4060, 1:1000), Phospho-Akt (Ser473) (D9E) is a validated monoclonal antibody used in 10015 publications. Phospho-Akt (Ser473) (D9E) XP® Rabbit mAb detects endogenous levels of Akt1 only when phosphorylated at Ser473. This antibody also recognizes Akt2 and Akt3 when phosphorylated at the corresponding residues. Species Reactivity: Human, Mouse, Rat, Hamster, Monkey, D. melanogaster, Zebrafish, Bovine. It is affinity purified and suitable for Western Blot, immunohistochemistry, IP.

Animals and other organisms

Policy information about studies involving animals; ARRIVE guidelines recommended for reporting animal research

Laboratory animals

Only male mice were used in the studies, since female mice are largely resistant to diet-induced obesity and to alterations in glucose metabolism when chronically fed with a high-fat diet.

Figure 1A: 9-10-wk old male C57BL/6J wt inceptor Iir +/+ (WT) and inceptor Iir -/- (KO) mice
Figure 1B: 28-wk old male C57BL/6J wt inceptor Iir +/+ (WT) and inceptor Iir -/- (KO) mice
Figure 1C: 10-30-wk old male C57BL/6J wt inceptor Iir +/+ (WT) and inceptor Iir -/- (KO) mice
Figure 1D and E: 19-wk old male C57BL/6J wt inceptor Iir +/+ (WT) and inceptor Iir -/- (KO) mice

Figure 1F and G: 23-wk old male C57BL/6J wt inceptor Iir +/+ (WT) and inceptor Iir -/- (KO) mice
Figure 1H, O and P: 19-wk old male C57BL/6J wt inceptor Iir +/+ (WT) and inceptor Iir -/- (KO) mice
Figure 1I and 1J: 27-wk old male C57BL/6J wt inceptor Iir +/+ (WT) and inceptor Iir -/- (KO) mice
Figure 1K-N: 30-wk old male C57BL/6J wt inceptor Iir +/+ (WT) and inceptor Iir -/- (KO) mice
Figure 2A and B: 18-24 week old male C57BL/6J WT mice
Figure 2 C and D: 28 week old male C57BL/6J WT mice
Figure 2E: 10 week old male C57BL/6J WT mice
Figure 2F: 14 week old male C57BL/6J WT mice
Figure 3A: 11 week old male C57BL/6J Nestin Cre+/- Iirwt/wt (WT) and Nestin Cre+/- Iirflx/flx (KO) mice
Figure 3B: 8-30 week old male C57BL/6J Nestin Cre+/- Iirwt/wt (WT) and Nestin Cre+/- Iirflx/flx (KO) mice
Figure 3 C-F: 26 week old male C57BL/6J Nestin Cre+/- Iirwt/wt (WT) and Nestin Cre+/- Iirflx/flx (KO) mice
Figure 3G and H: 27 week old male C57BL/6J Nestin Cre+/- Iirwt/wt (WT) and Nestin Cre+/- Iirflx/flx (KO) mice
Figure 3 I and J: 28 week old male C57BL/6J Nestin Cre+/- Iirwt/wt (WT) and Nestin Cre+/- Iirflx/flx (KO) mice
Figure 3 K: 27 week old male C57BL/6J Nestin Cre+/- Iirwt/wt (WT) and Nestin Cre+/- Iirflx/flx (KO) mice
Figure 3L: 32 week old male C57BL/6J Nestin Cre+/- Iirwt/wt (WT) and Nestin Cre+/- Iirflx/flx (KO) mice
Figure 3 M and N: 36 week old male C57BL/6J Nestin Cre+/- Iirwt/wt (WT) and Nestin Cre+/- Iirflx/flx (KO) mice
Figure 4A: 26 week old male Ins1 CreERT-/- Iirflx/flx (WT) and Ins1 CreERT+/- Iirflx/flx (KO) C57BL/6J mice
Figure 4B: 24-40 week old male Ins1 CreERT-/- Iirflx/flx (WT) and Ins1 CreERT+/- Iirflx/flx (KO) C57BL/6J mice
Figure 4C-E: 30 week old male Ins1 CreERT-/- Iirflx/flx (WT) and Ins1 CreERT+/- Iirflx/flx (KO) C57BL/6J mice
Figure 4 F and G: 18 week old male Ins1 CreERT-/- Iirflx/flx (WT) and Ins1 CreERT+/- Iirflx/flx (KO) C57BL/6J mice
Figure 4 H and I: 18 week old male Ins1 CreERT-/- Iirflx/flx (WT) and Ins1 CreERT+/- Iirflx/flx (KO) C57BL/6J mice
Figure 4 J and K: 22 week old male Ins1 CreERT-/- Iirflx/flx (WT) and Ins1 CreERT+/- Iirflx/flx (KO) C57BL/6J mice
Figure 4 L and M: 26 week old male Ins1 CreERT-/- Iirflx/flx (WT) and Ins1 CreERT+/- Iirflx/flx (KO) C57BL/6J mice
Figure 4 N: 18 week old male Ins1 CreERT-/- Iirflx/flx (WT) and Ins1 CreERT+/- Iirflx/flx (KO) C57BL/6J mice
Extended Data Figure 1 A-C: 30 week old male C57BL/6J inceptor Iir+/+ (WT) and Iir-/- (KO) mice
Extended Data Figure 2 A-C: 36 week old male C57BL/6J inceptor Nestin Cre+/- Iirwt/wt (WT) and Nestin Cre+/- Iirflx/flx (KO) mice
Extended Data Figure 3A: 20 week old male C57BL/6J Agrp-Cre+/- Iirwt/wt (WT) and Agrp-Cre+/- Iirflx/flx (KO) mice
Extended Data Figure 3B-G: 30 week old male C57BL/6J Agrp-Cre+/- Iirwt/wt (WT) and Agrp-Cre+/- Iirflx/flx (KO) mice
Extended Data Figure 3 H and I: 31 week old male C57BL/6J Agrp-Cre+/- Iirwt/wt (WT) and Agrp-Cre+/- Iirflx/flx (KO) mice
Extended Data Figure 3J: 20 week old male C57BL/6J Pomc-Cre+/- Iirwt/wt (WT) and Pomc-Cre+/- Iirflx/flx (KO) mice
Extended Data Figure 3L-P: 30 week old male C57BL/6J Pomc-Cre+/- Iirwt/wt (WT) and Pomc-Cre+/- Iirflx/flx (KO) mice
Extended Data Figure 3 Q and R: 30 week old male C57BL/6J Pomc-Cre+/- Iirwt/wt (WT) and Pomc-Cre+/- Iirflx/flx (KO) mice
Extended Data Figure 4 A-C: 26 week old male Ins1 CreERT-/- Iirflx/flx (WT) and Ins1 CreERT+/- Iirflx/flx (KO) C57BL/6J mice

Wild animals

no wild animals were used in the study

Field-collected samples

no field collected animals were used in the study

Ethics oversight

Experiments were performed in accordance with the Animal Protection Law of the European Union after permission by the Government of Upper Bavaria (Regierung von Oberbayern), Germany

Note that full information on the approval of the study protocol must also be provided in the manuscript.
