# Supplementary figures and images for: Global, neuronal or β cell-specific deletion of inceptor improves glucose homeostasis in male mice with diet-induced obesity (part 1 of 2)
Source: Nat Metab. 2024 Feb 28;6(3):448–57. doi: 10.1038/s42255-024-00991-3 (PMC10963260; doi:10.1038/s42255-024-00991-3)

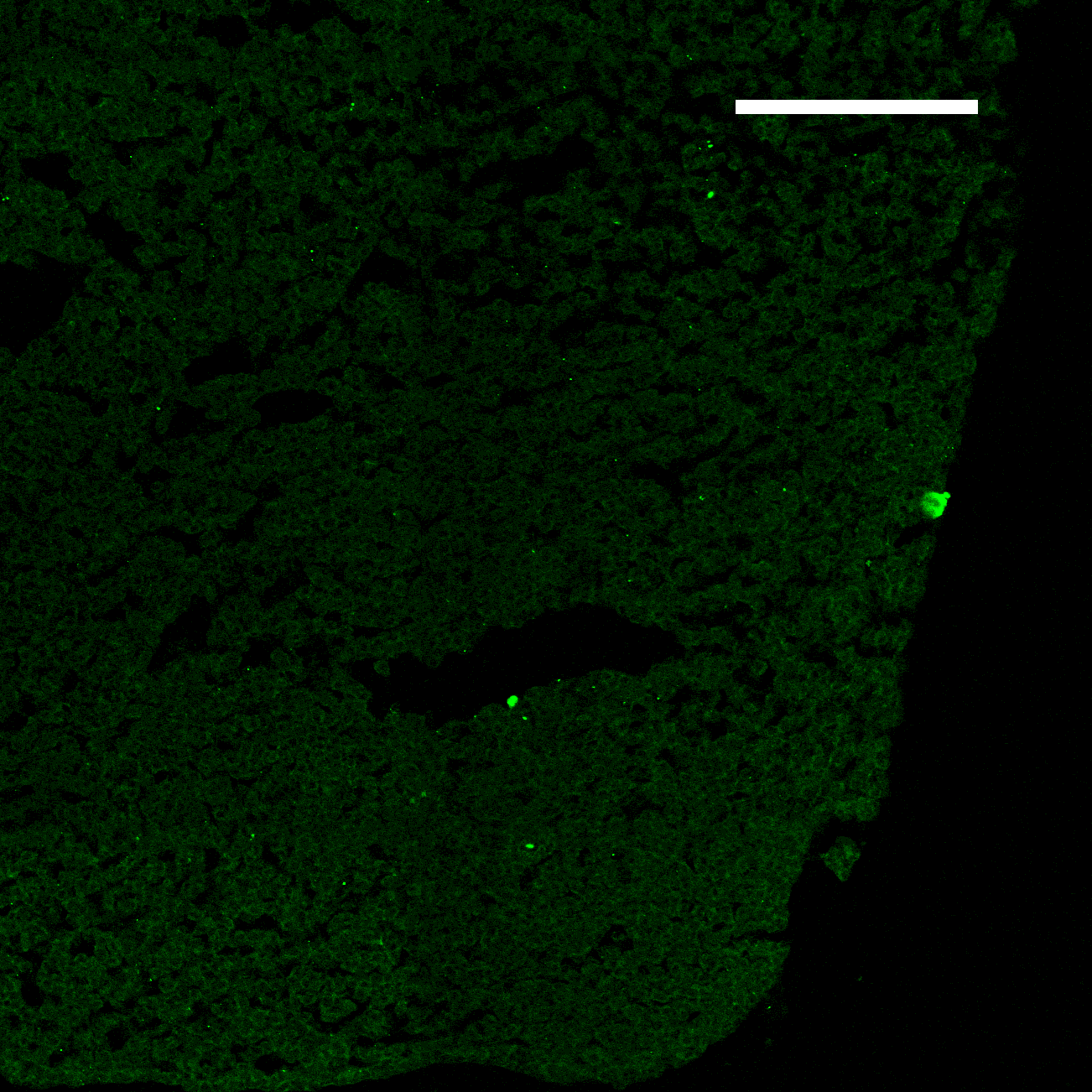

Supplement: Supplementary file 11 — Original data for Fig. 1a. [file 42255_2024_991_MOESM11_ESM.zip › Figure 1A/20230914_28_hom_Iir-555_RGB-inceptor-only_scale1.tif]

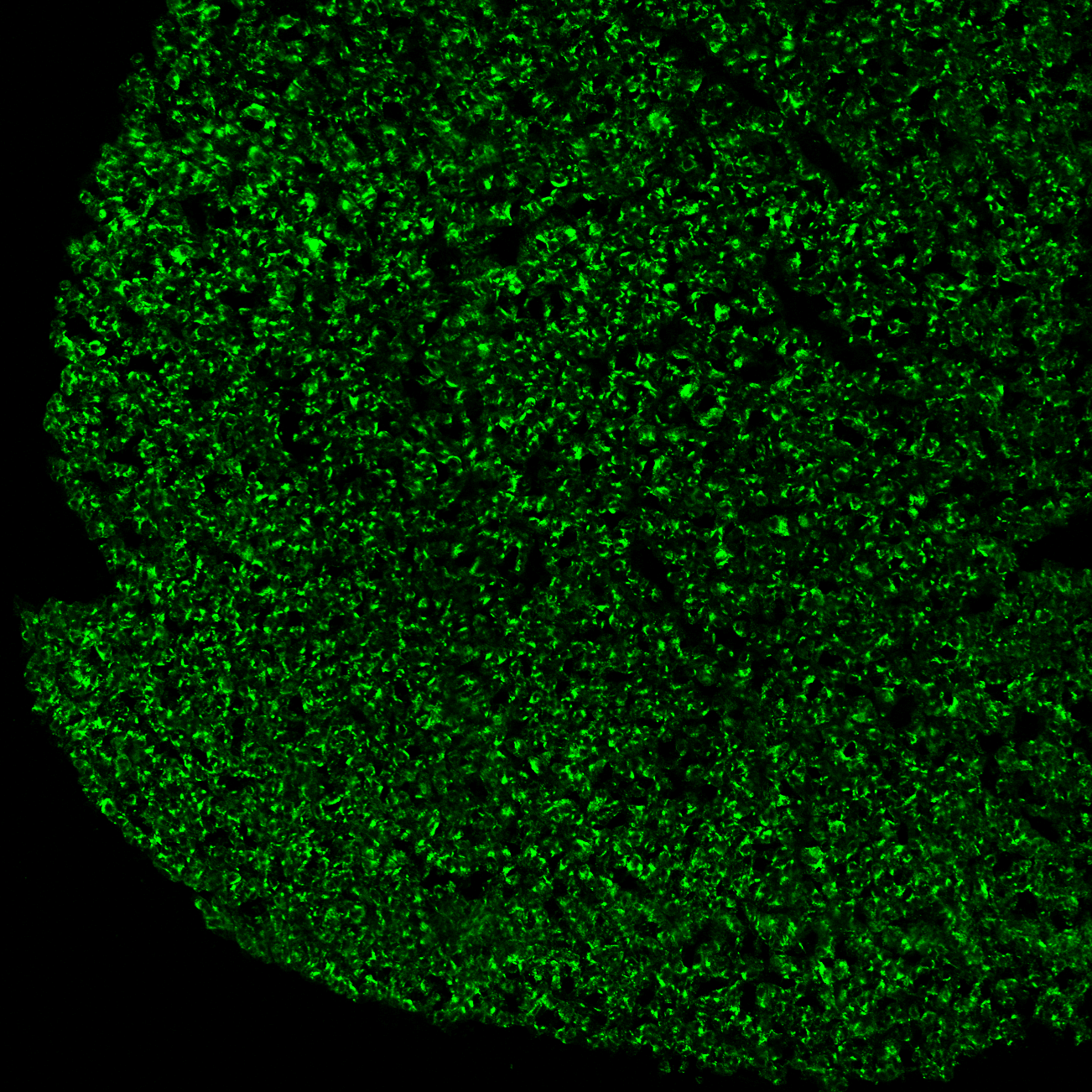

Supplement: Supplementary file 11 — Original data for Fig. 1a. [file 42255_2024_991_MOESM11_ESM.zip › Figure 1A/20230914_60_wt_Iir-555_RGB-inceptor-only_no_scale.tif]

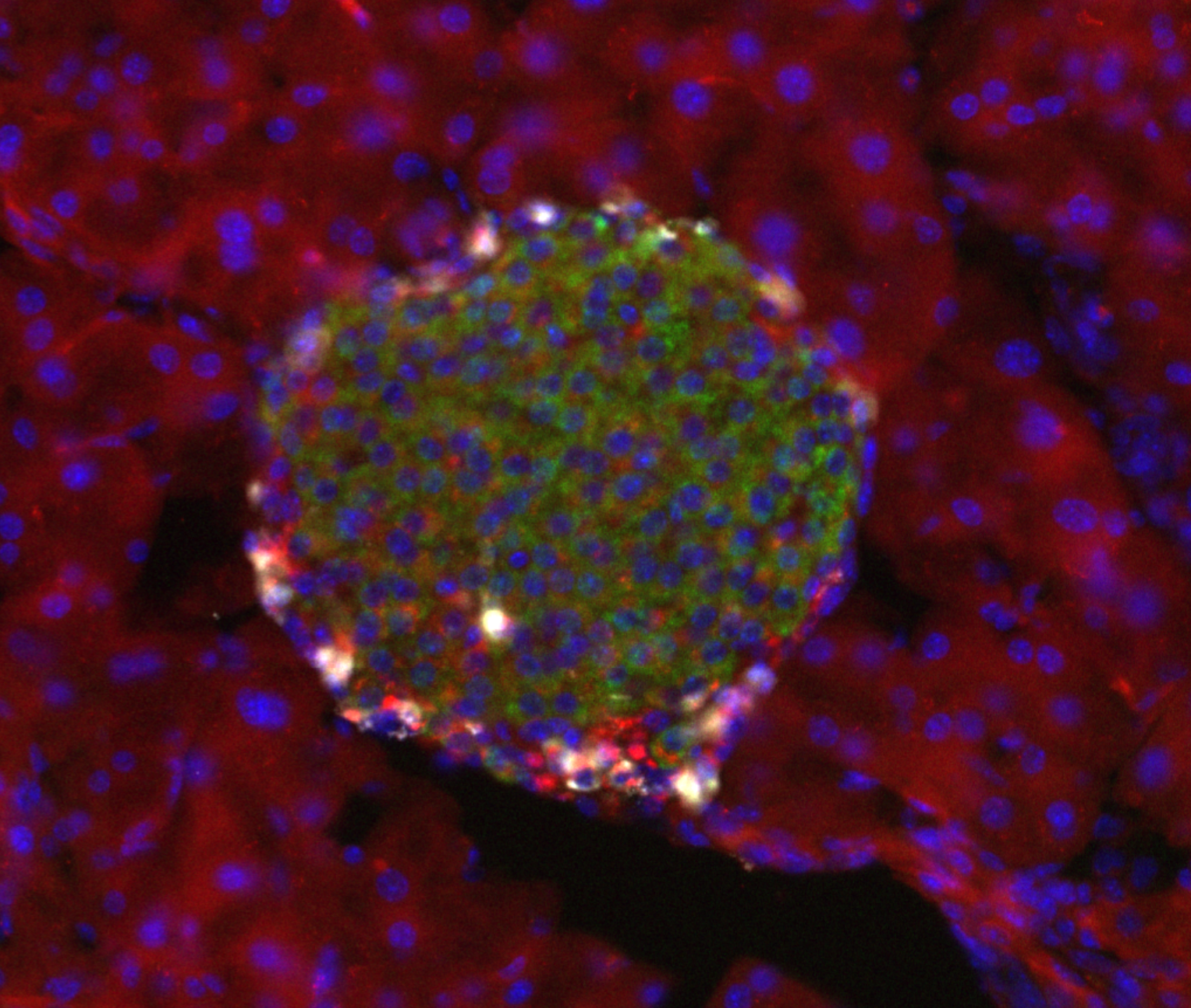

Supplement: Supplementary file 11 — Original data for Fig. 1a. [file 42255_2024_991_MOESM11_ESM.zip › Figure 1A/IDO Grandl Pancr Ins750 Gcg647 Inc555 Slide M59 WT rescan.tiff]

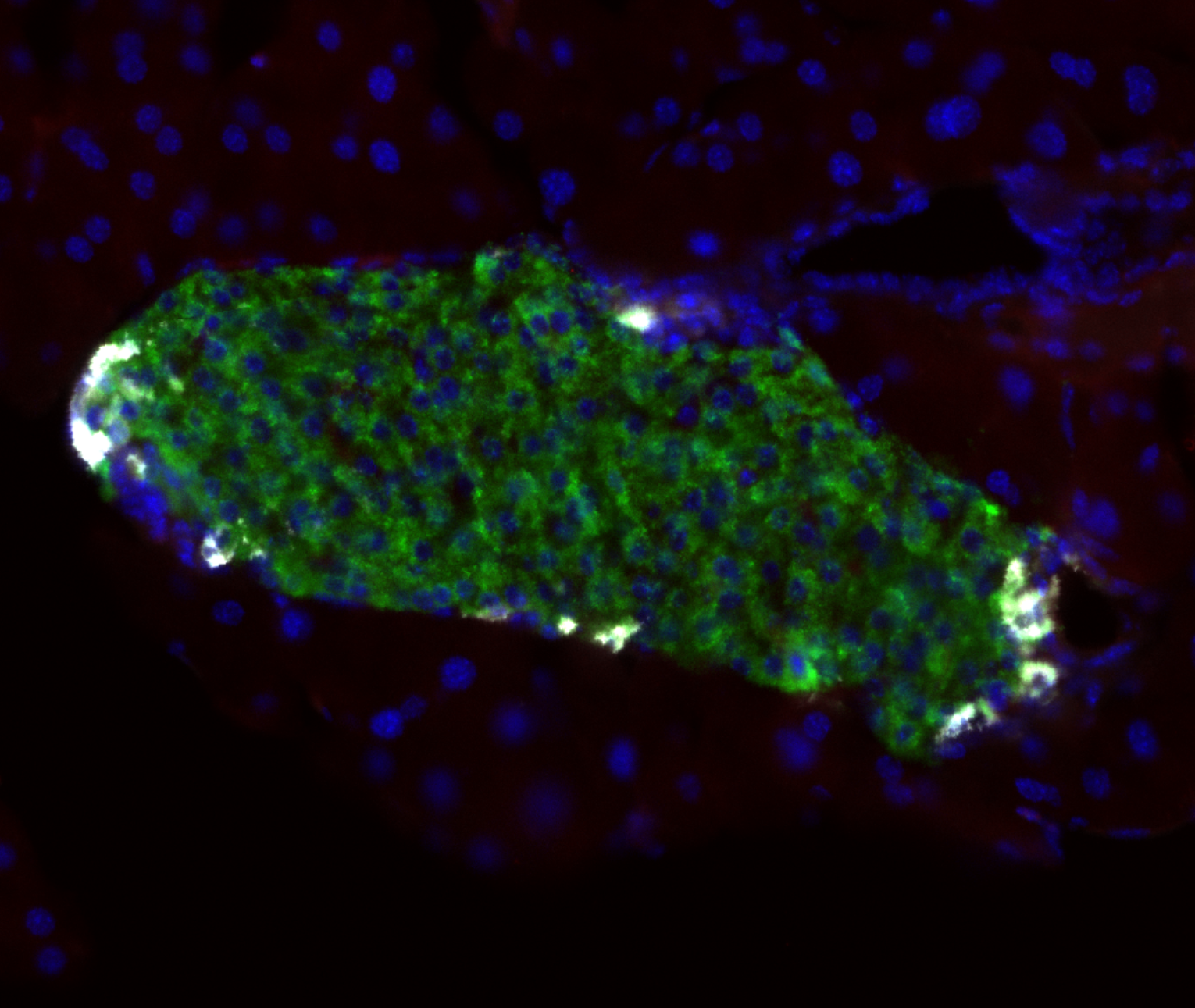

Supplement: Supplementary file 11 — Original data for Fig. 1a. [file 42255_2024_991_MOESM11_ESM.zip › Figure 1A/IDO Grandl Pancr Ins750 Gcg647 Inc555 Slide2 Probe M47.tiff]

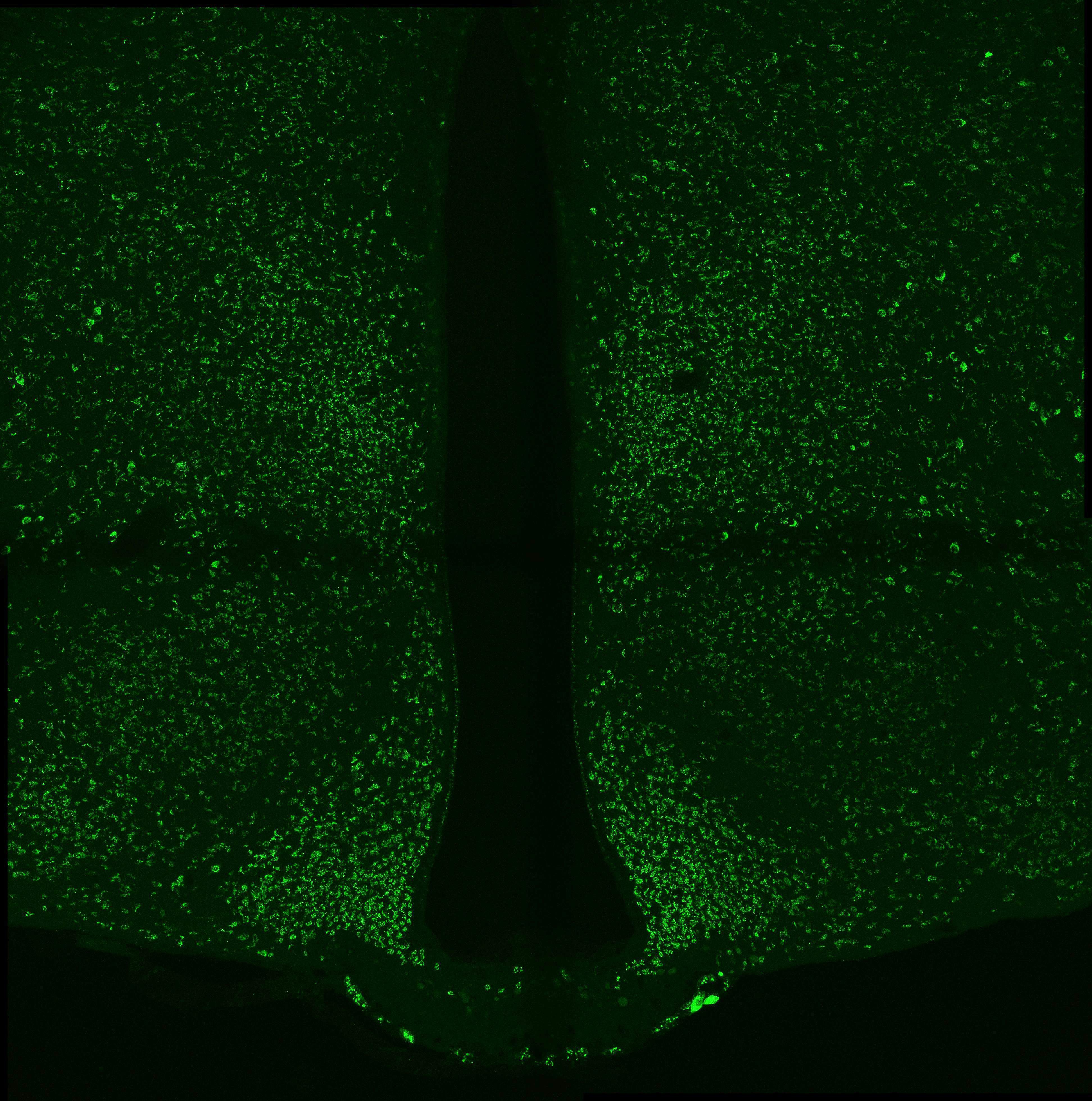

Supplement: Supplementary file 12 — Original data for Fig. 2a–d. [file 42255_2024_991_MOESM12_ESM.zip › Figure 2A/IDRr-WT2-MidARH4-20x-brightness.jpg]

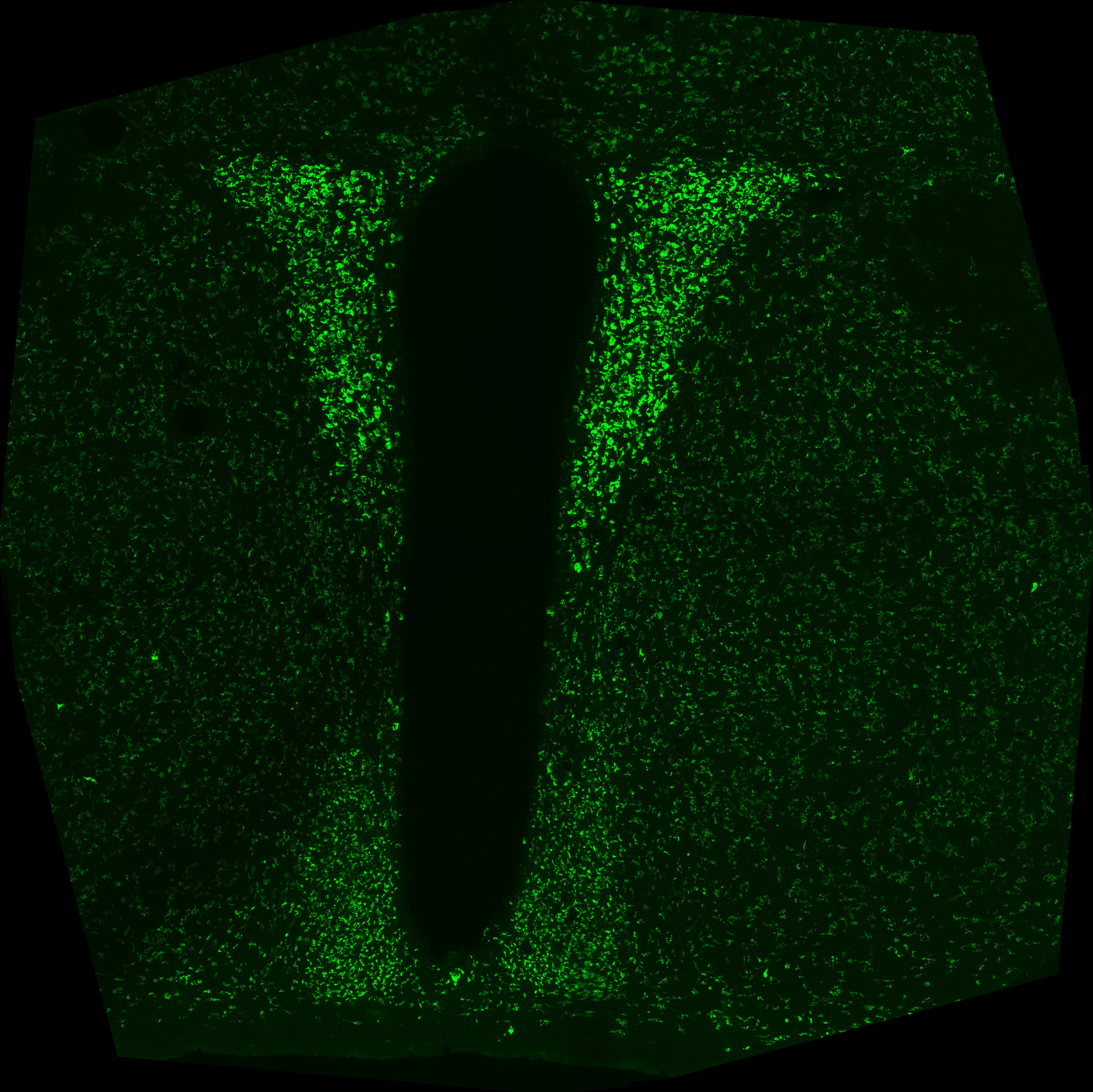

Supplement: Supplementary file 12 — Original data for Fig. 2a–d. [file 42255_2024_991_MOESM12_ESM.zip › Figure 2A/IGFRL-PVN.jpg]

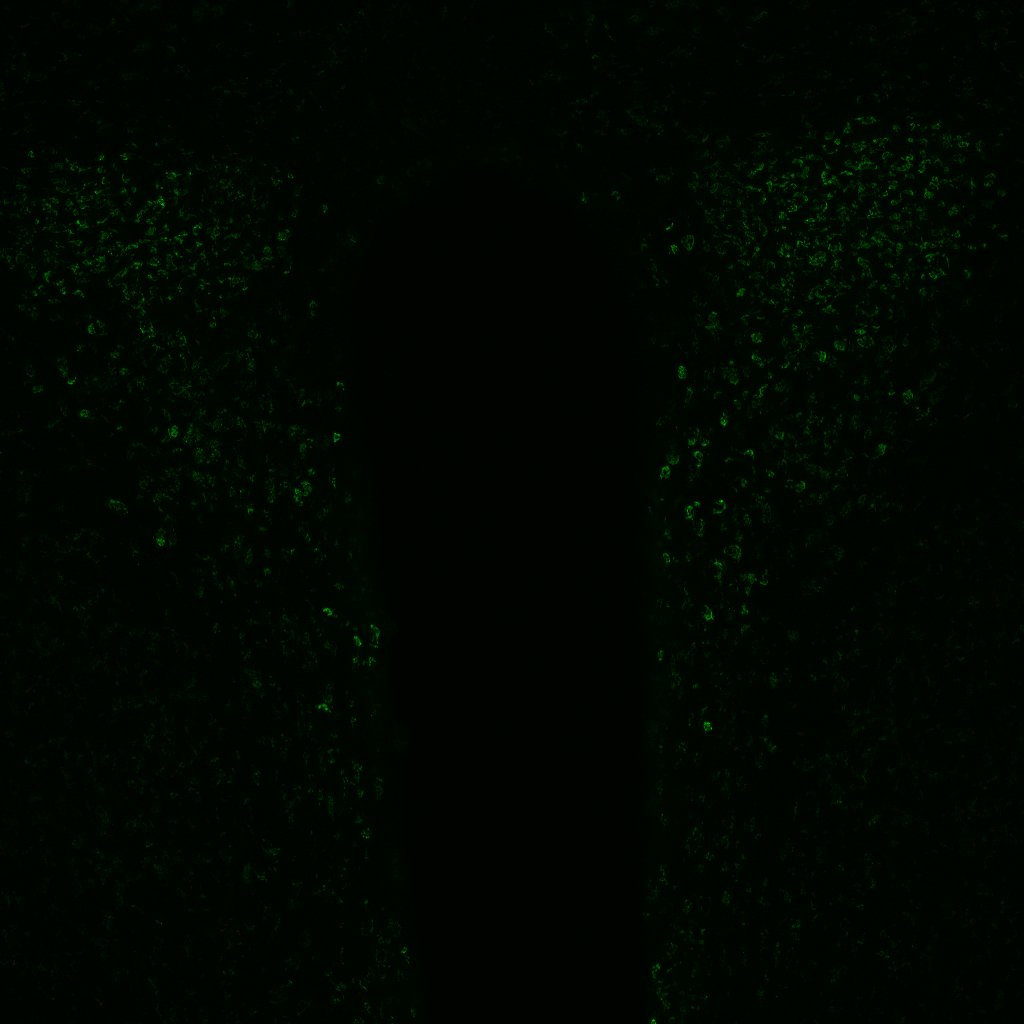

Supplement: Supplementary file 12 — Original data for Fig. 2a–d. [file 42255_2024_991_MOESM12_ESM.zip › Figure 2B/Mouse 30/1821-5 PVH.jpg]

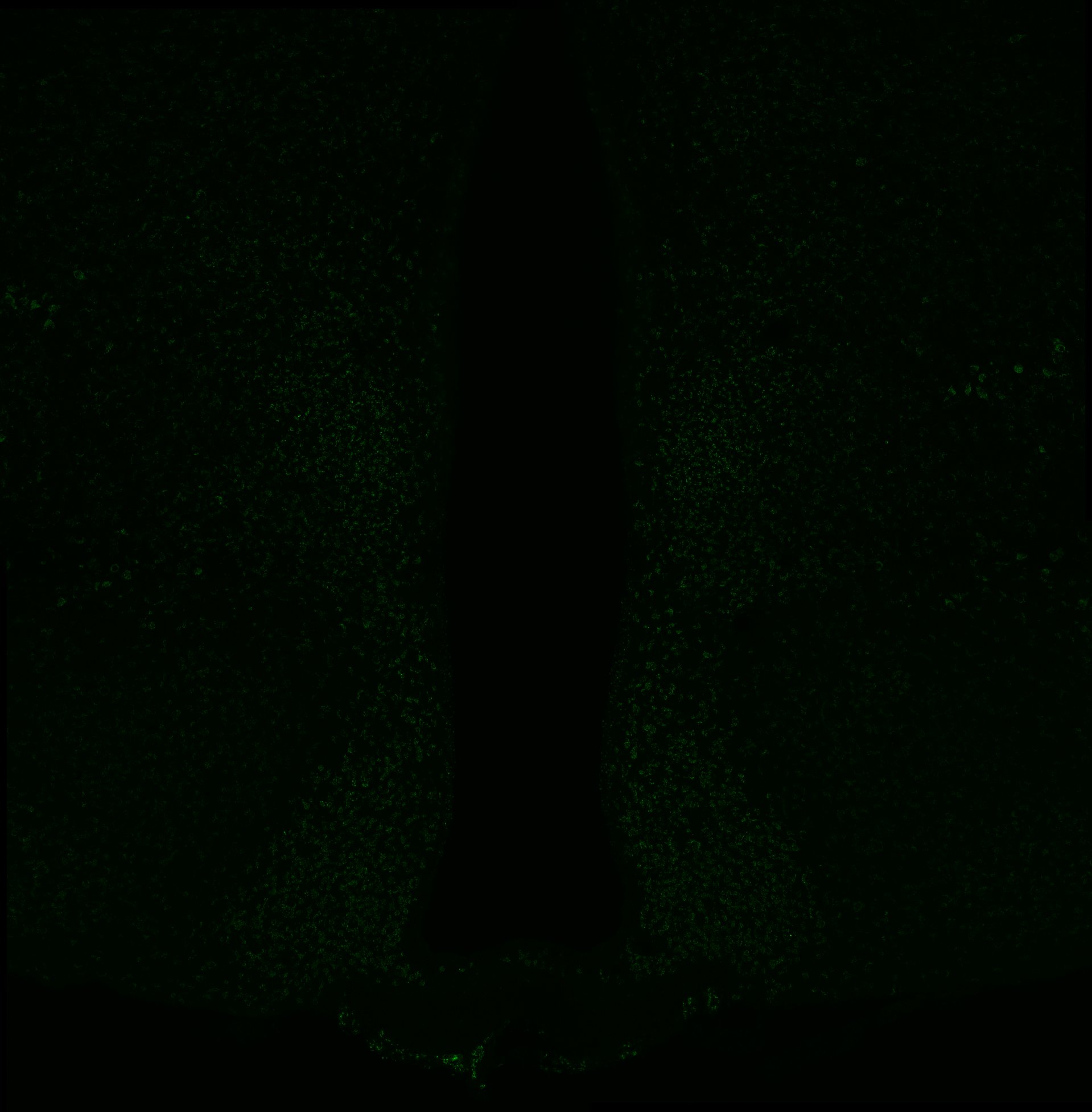

Supplement: Supplementary file 12 — Original data for Fig. 2a–d. [file 42255_2024_991_MOESM12_ESM.zip › Figure 2B/Mouse 30/1821-5 MidARH3.jpg]

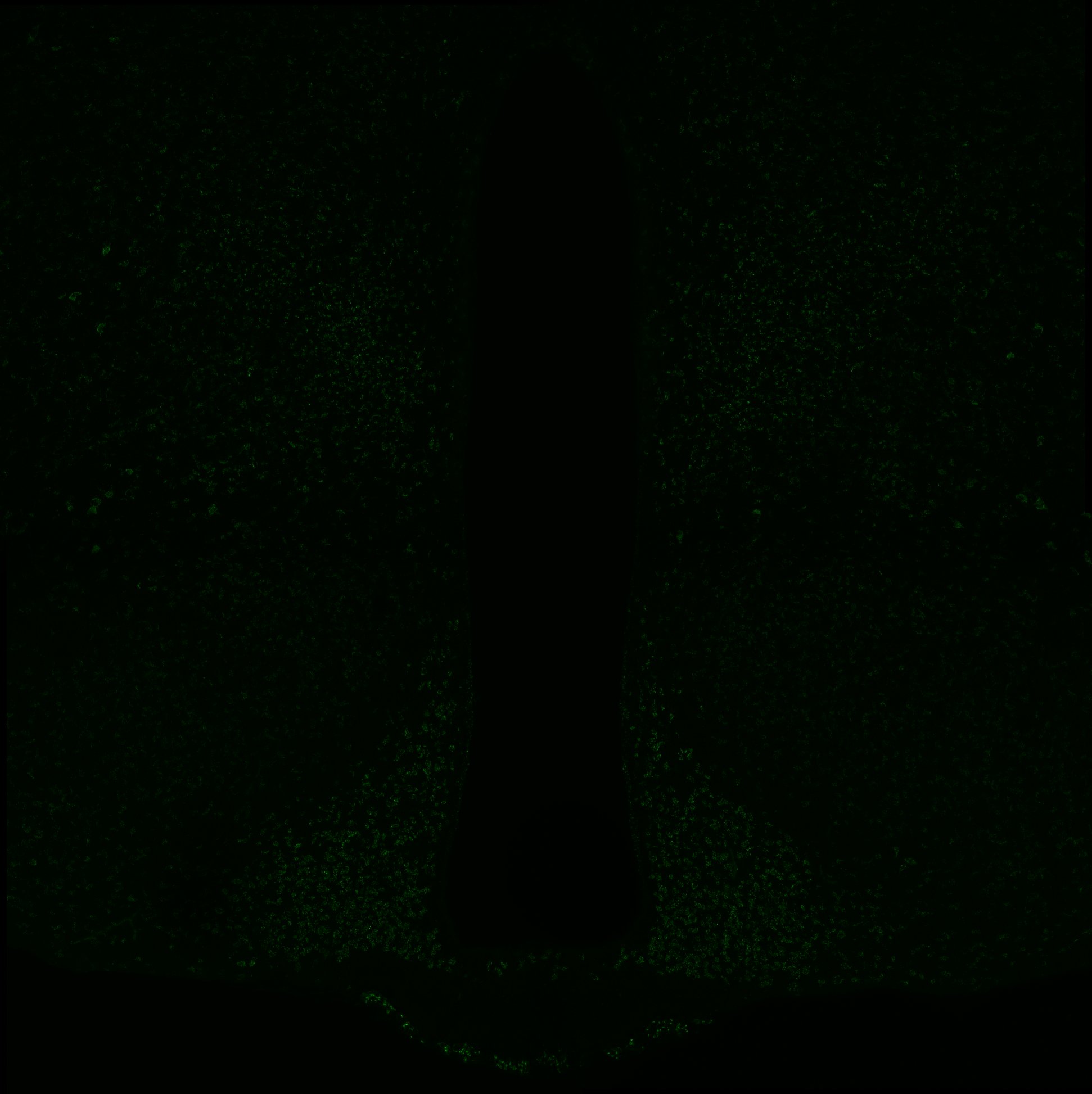

Supplement: Supplementary file 12 — Original data for Fig. 2a–d. [file 42255_2024_991_MOESM12_ESM.zip › Figure 2B/Mouse 30/1821-5 MidARH2.jpg]

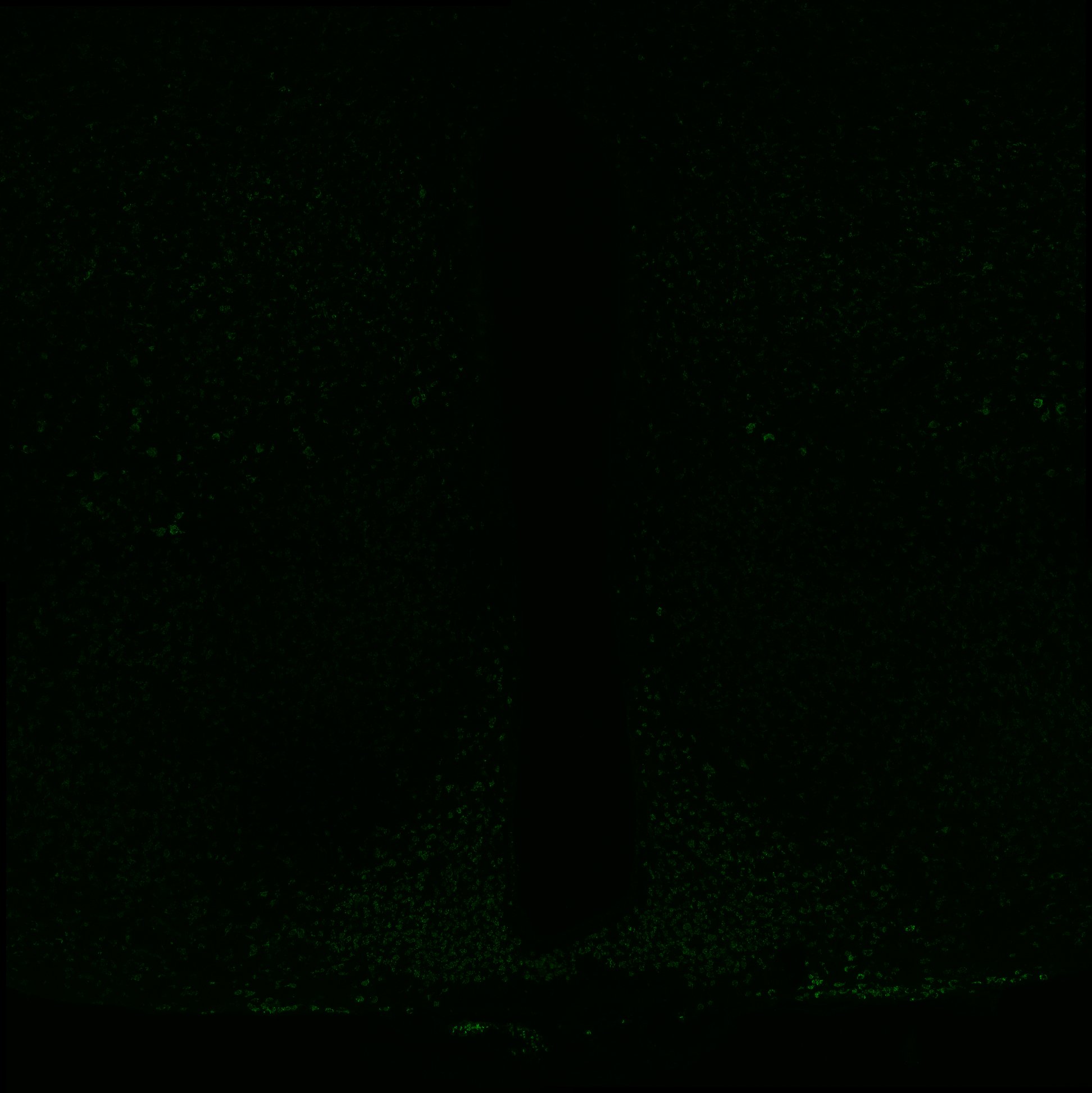

Supplement: Supplementary file 12 — Original data for Fig. 2a–d. [file 42255_2024_991_MOESM12_ESM.zip › Figure 2B/Mouse 30/1821-5 MidARH1.jpg]

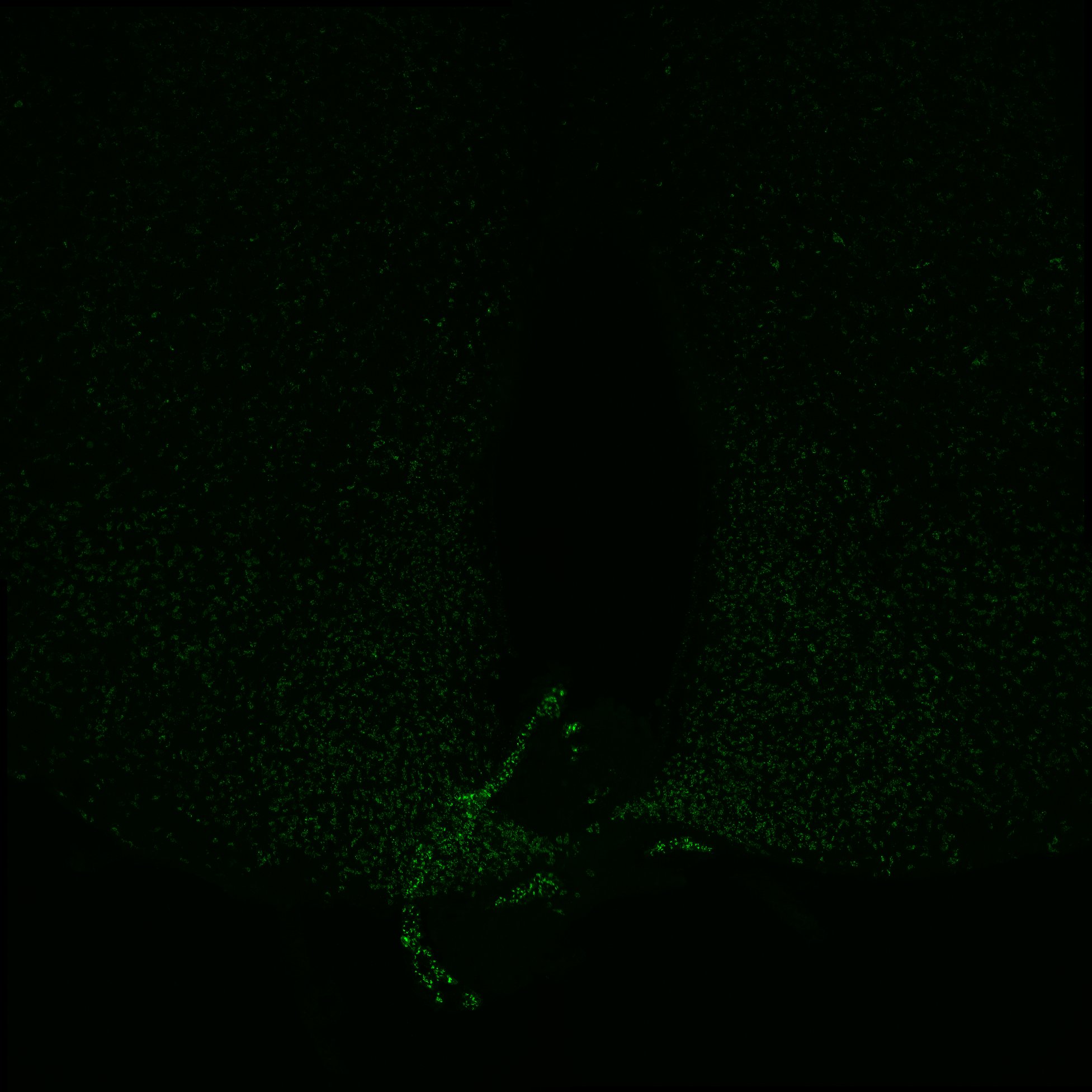

Supplement: Supplementary file 12 — Original data for Fig. 2a–d. [file 42255_2024_991_MOESM12_ESM.zip › Figure 2B/Mouse 22/1814-2 PostARH.jpg]

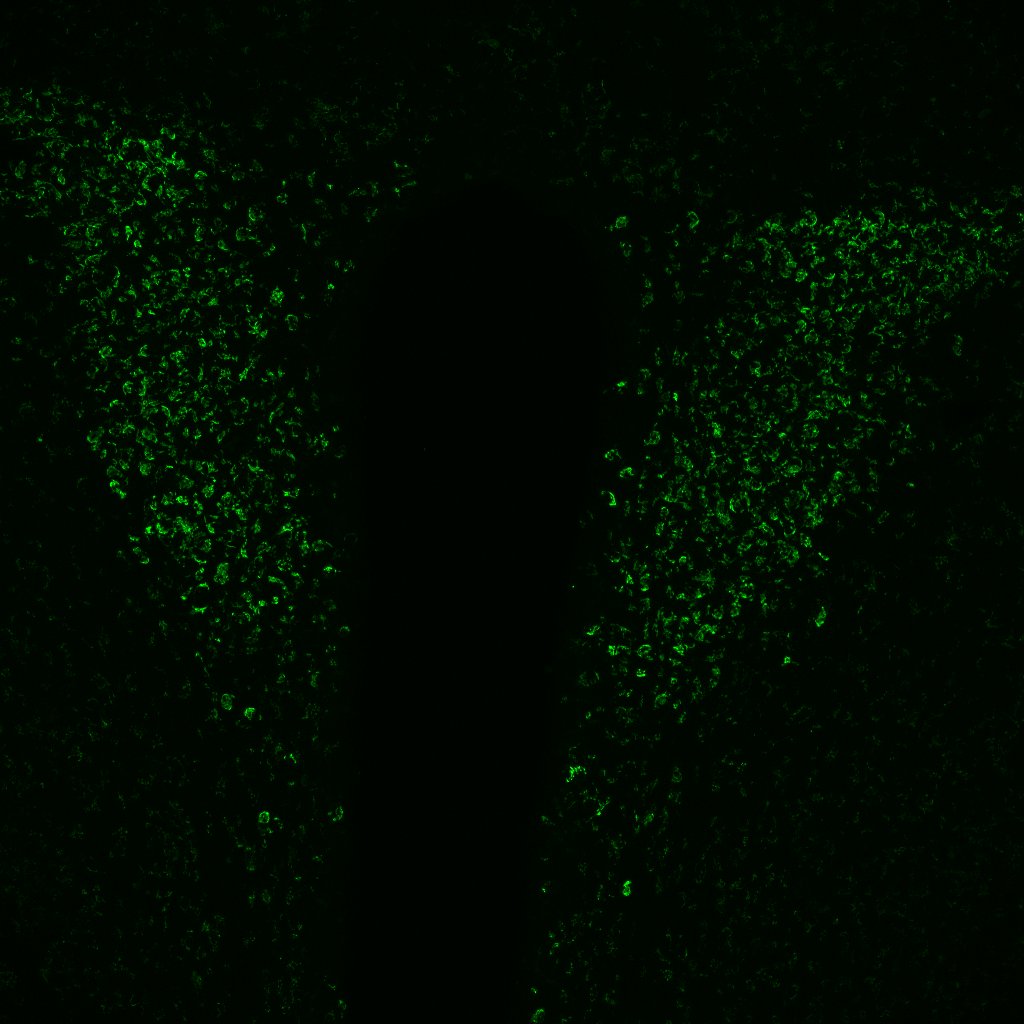

Supplement: Supplementary file 12 — Original data for Fig. 2a–d. [file 42255_2024_991_MOESM12_ESM.zip › Figure 2B/Mouse 22/1814-2 PVH.jpg]

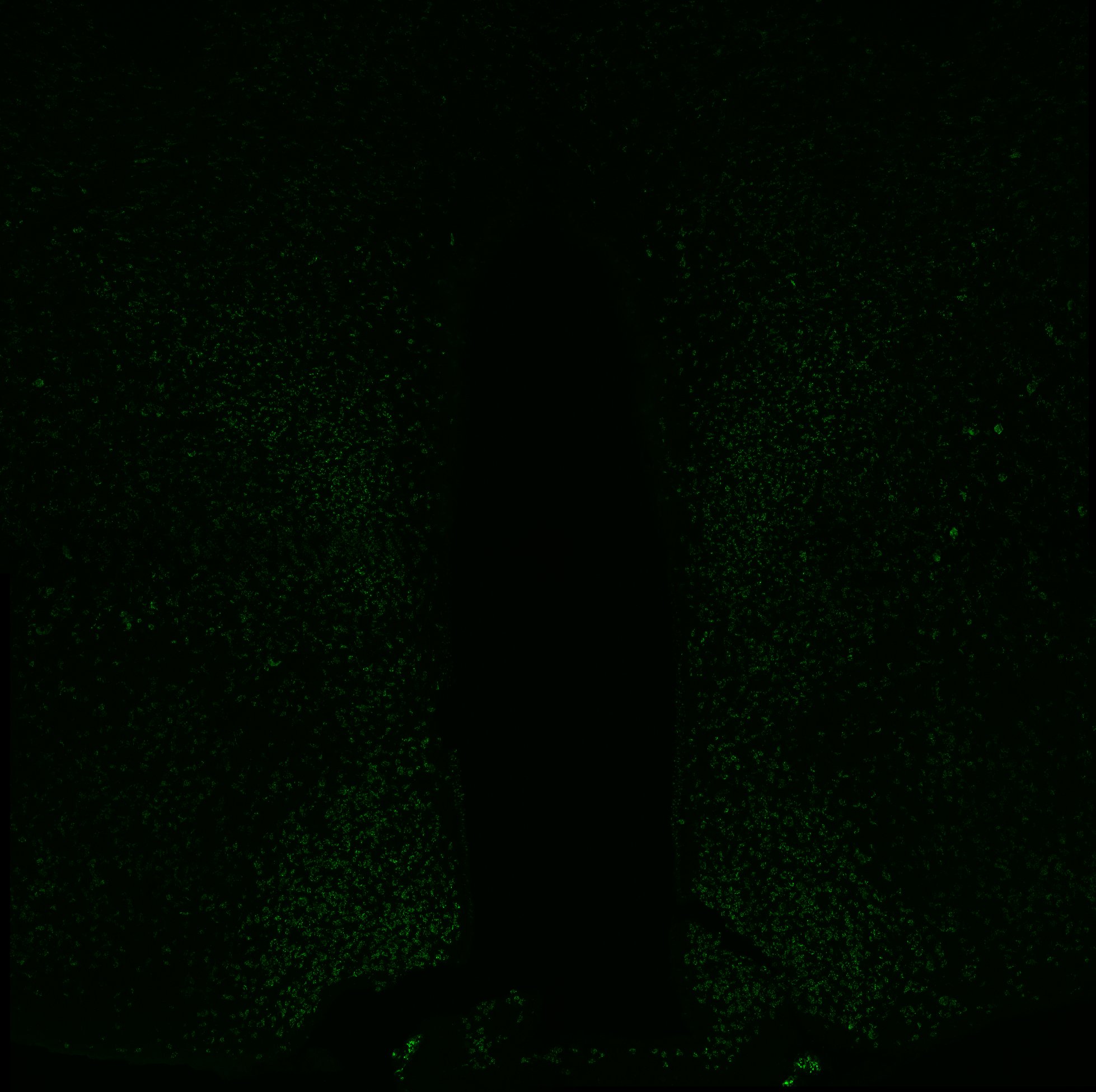

Supplement: Supplementary file 12 — Original data for Fig. 2a–d. [file 42255_2024_991_MOESM12_ESM.zip › Figure 2B/Mouse 22/1814-2 MidARH1.jpg]

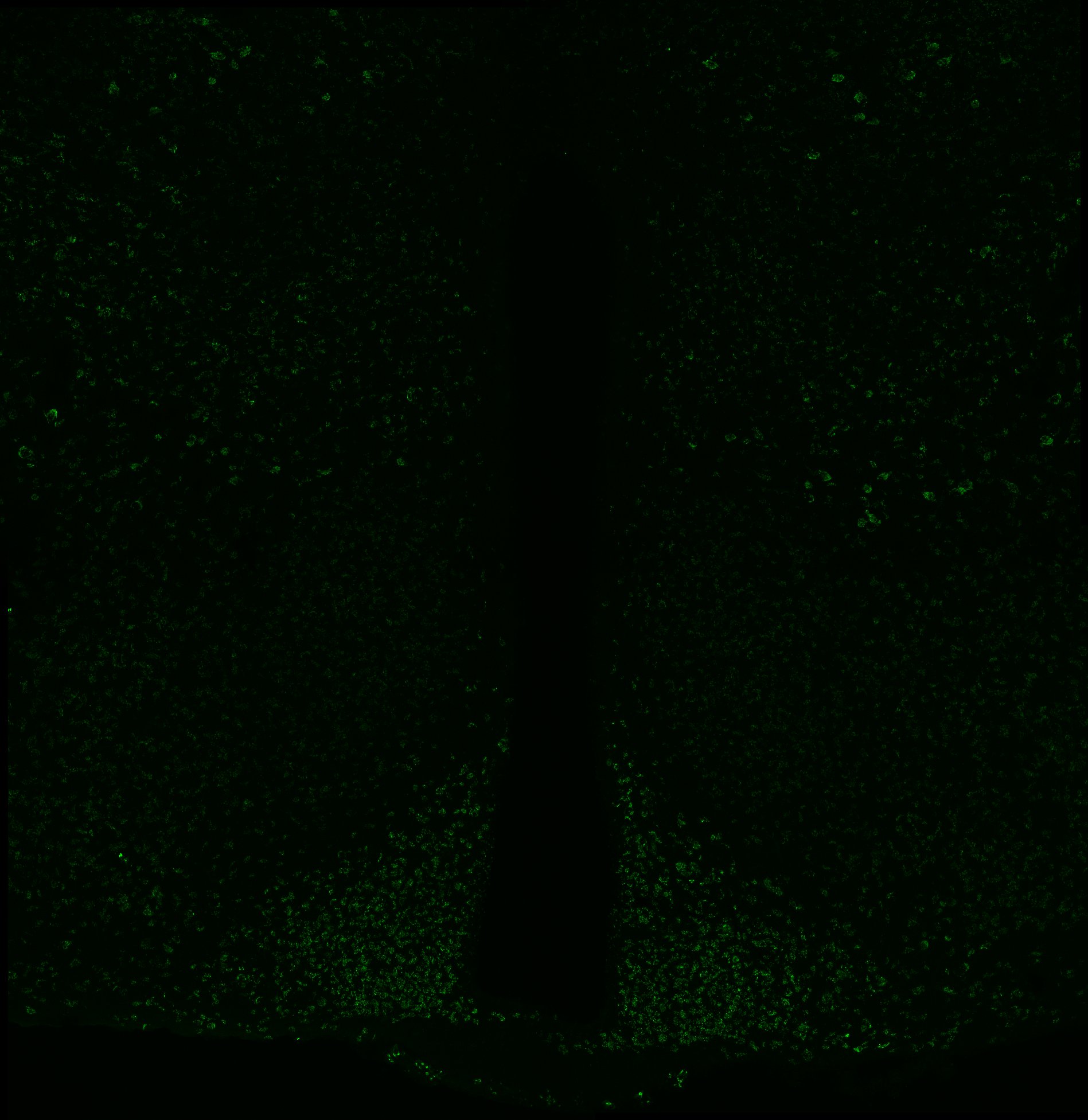

Supplement: Supplementary file 12 — Original data for Fig. 2a–d. [file 42255_2024_991_MOESM12_ESM.zip › Figure 2B/Mouse 22/1814-2 MidARH2.jpg]

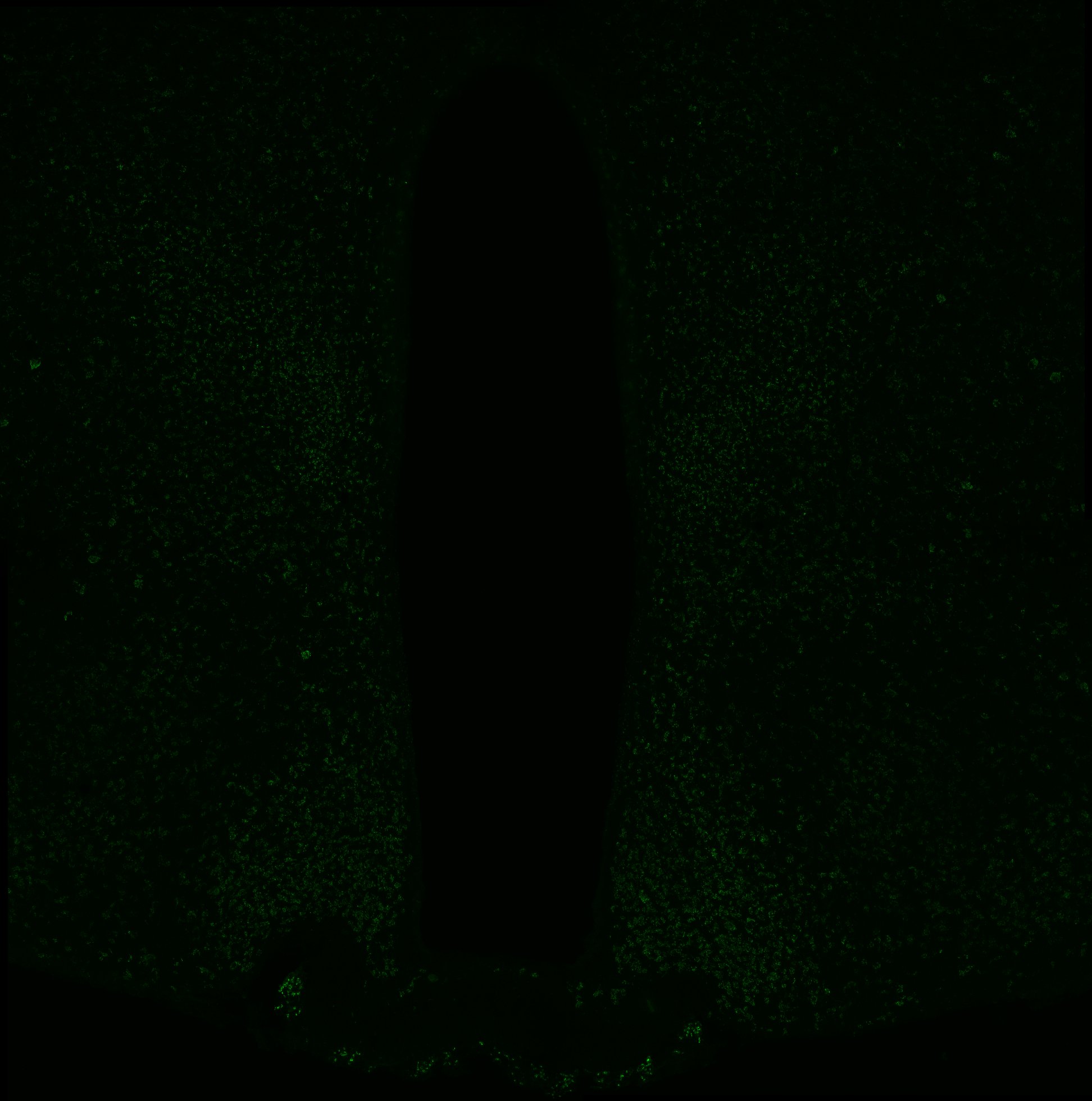

Supplement: Supplementary file 12 — Original data for Fig. 2a–d. [file 42255_2024_991_MOESM12_ESM.zip › Figure 2B/Mouse 25/1814-5 MidARH2.jpg]

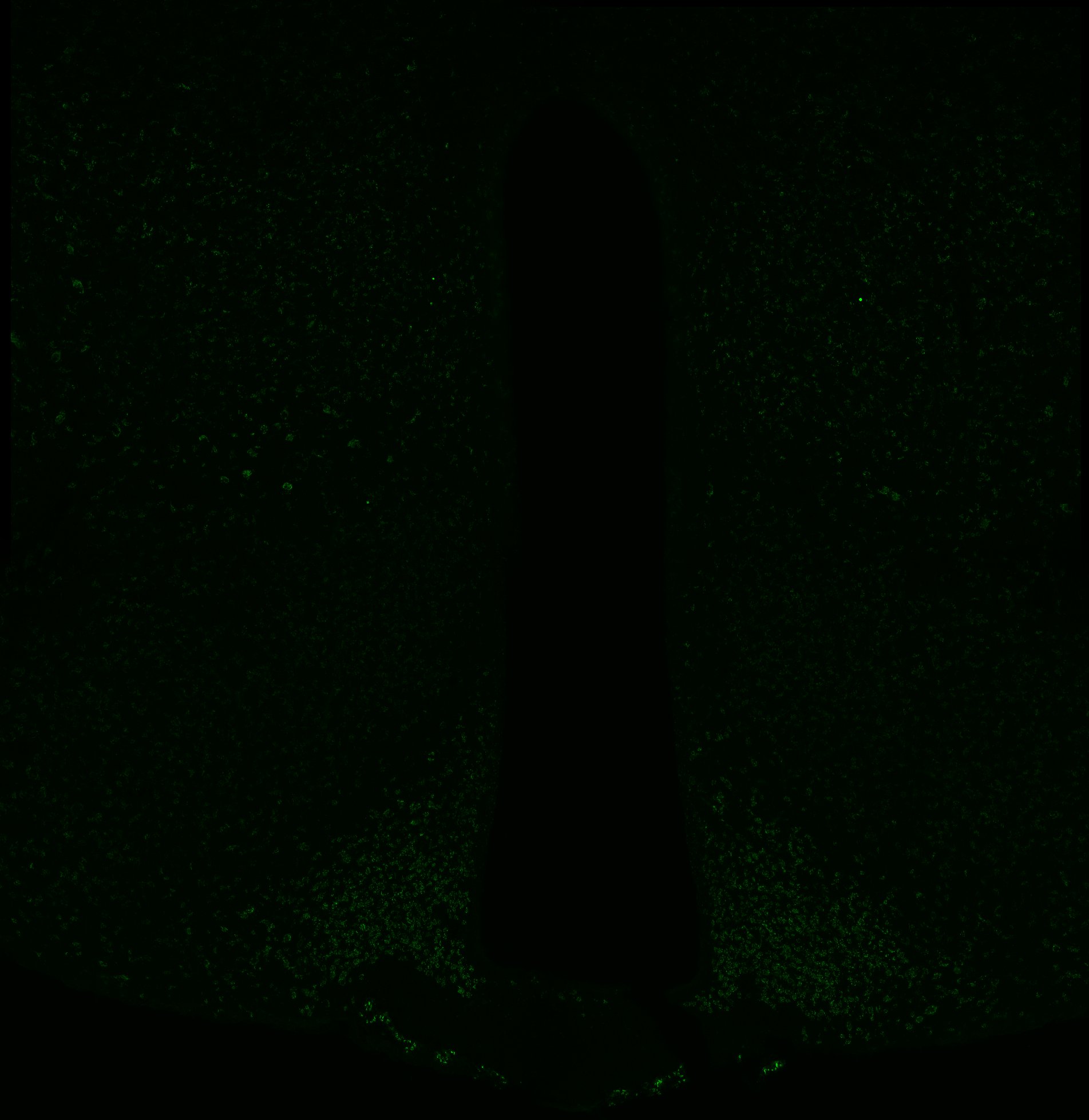

Supplement: Supplementary file 12 — Original data for Fig. 2a–d. [file 42255_2024_991_MOESM12_ESM.zip › Figure 2B/Mouse 25/1814-5 MidARH1.jpg]

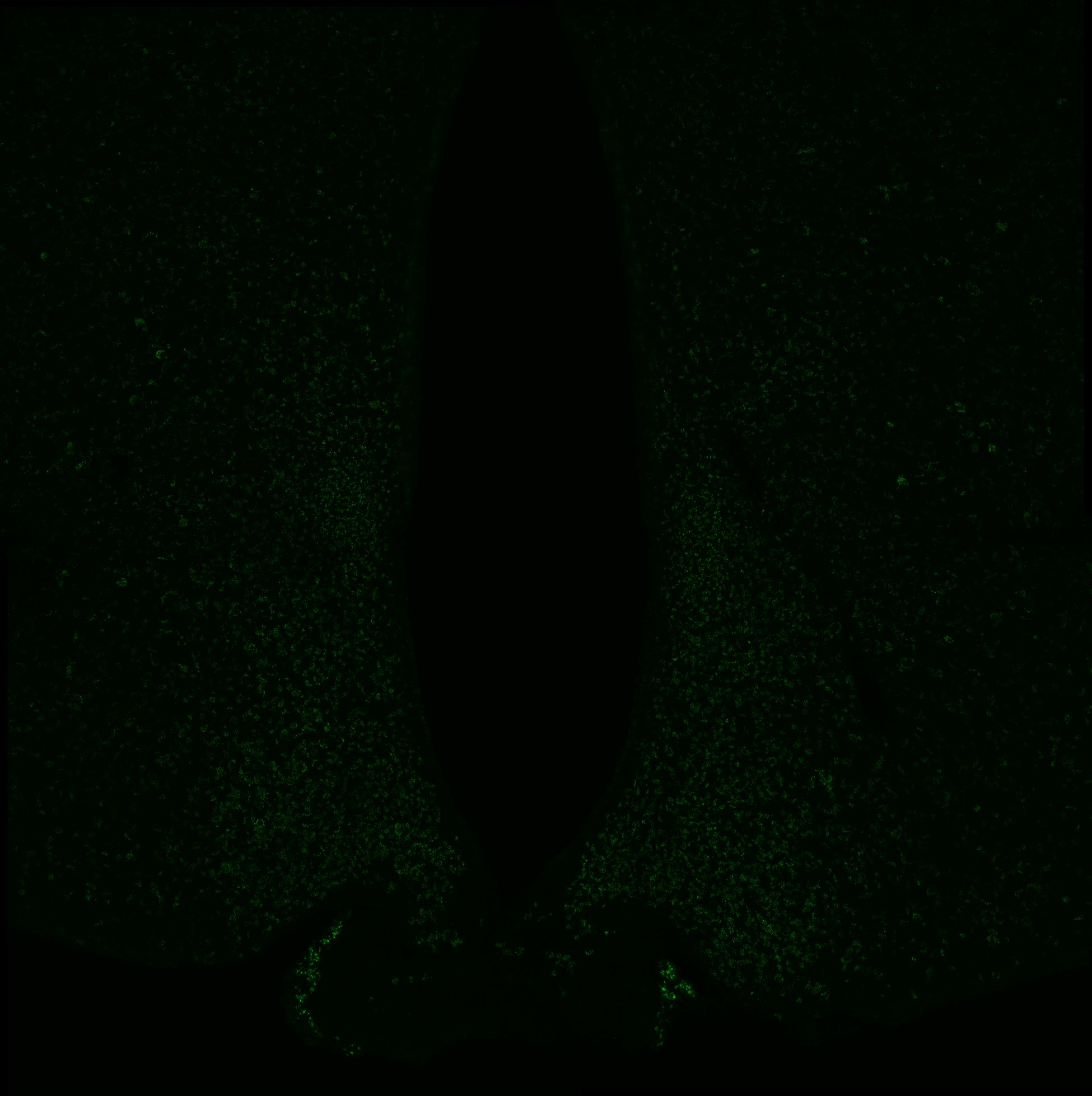

Supplement: Supplementary file 12 — Original data for Fig. 2a–d. [file 42255_2024_991_MOESM12_ESM.zip › Figure 2B/Mouse 25/1814-5 PostARH.jpg]

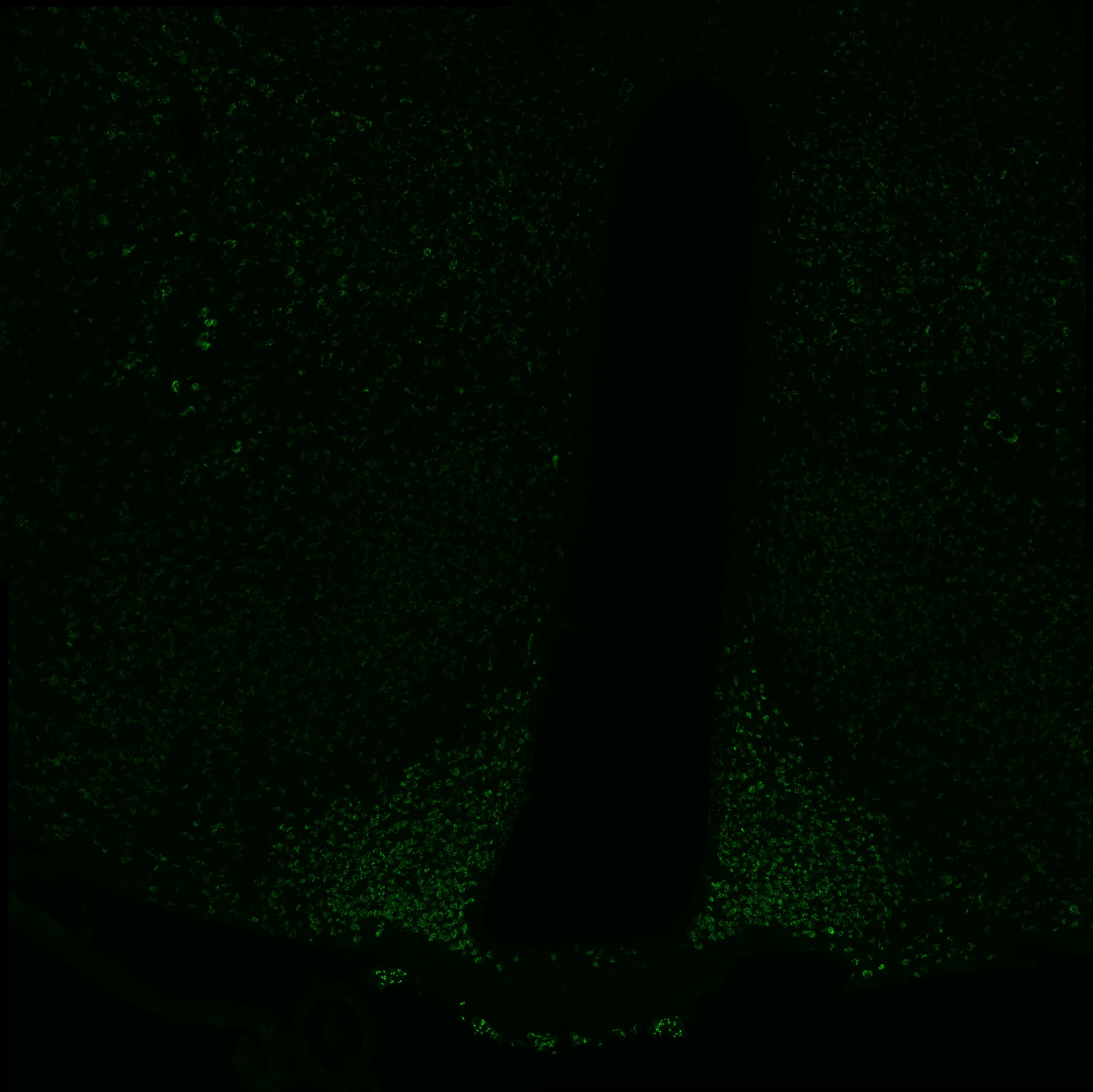

Supplement: Supplementary file 12 — Original data for Fig. 2a–d. [file 42255_2024_991_MOESM12_ESM.zip › Figure 2B/Mouse 13/1813-3 MidARH1.jpg]

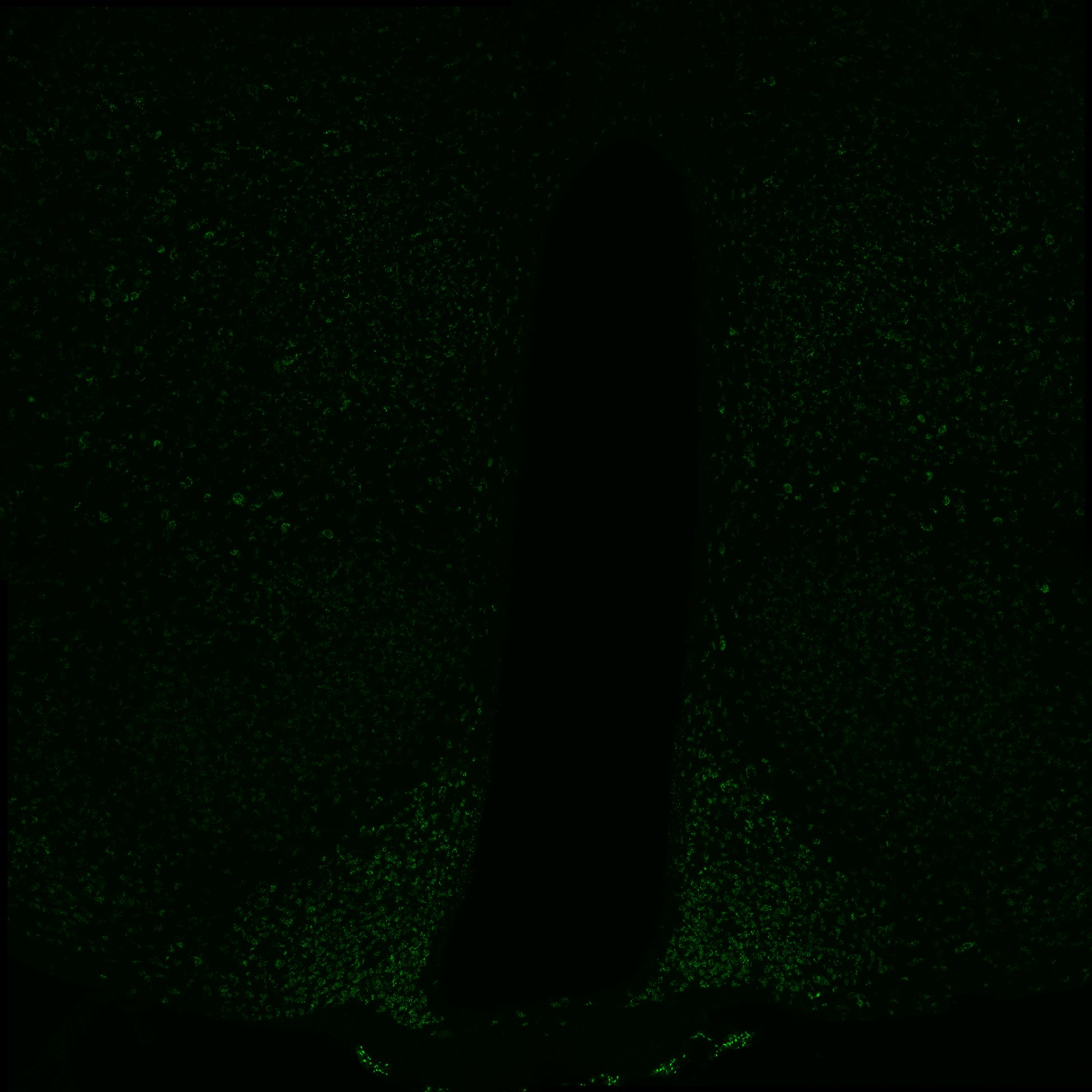

Supplement: Supplementary file 12 — Original data for Fig. 2a–d. [file 42255_2024_991_MOESM12_ESM.zip › Figure 2B/Mouse 13/1813-3 MidARH2.jpg]

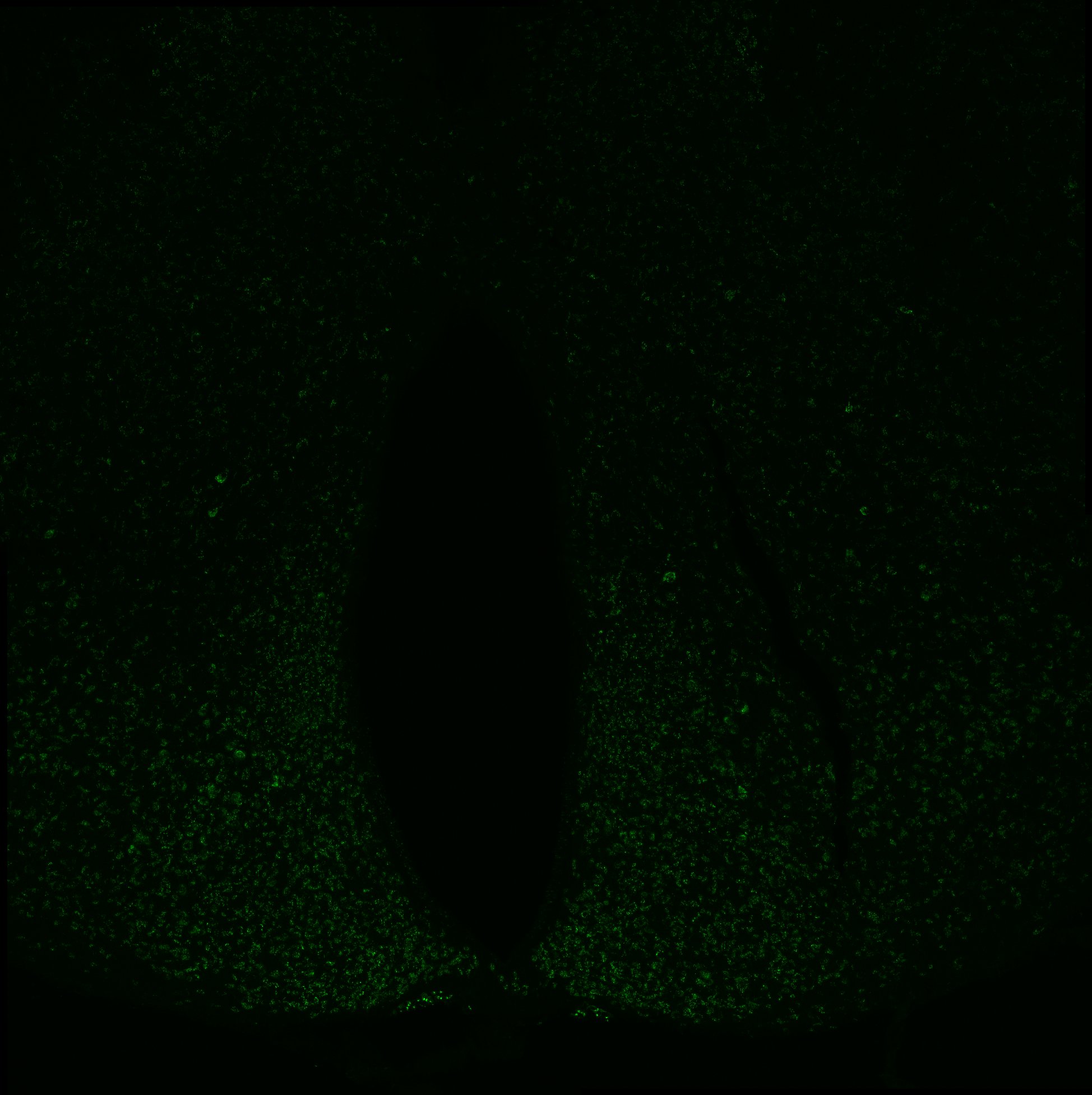

Supplement: Supplementary file 12 — Original data for Fig. 2a–d. [file 42255_2024_991_MOESM12_ESM.zip › Figure 2B/Mouse 13/1813-3 PostARH.jpg]

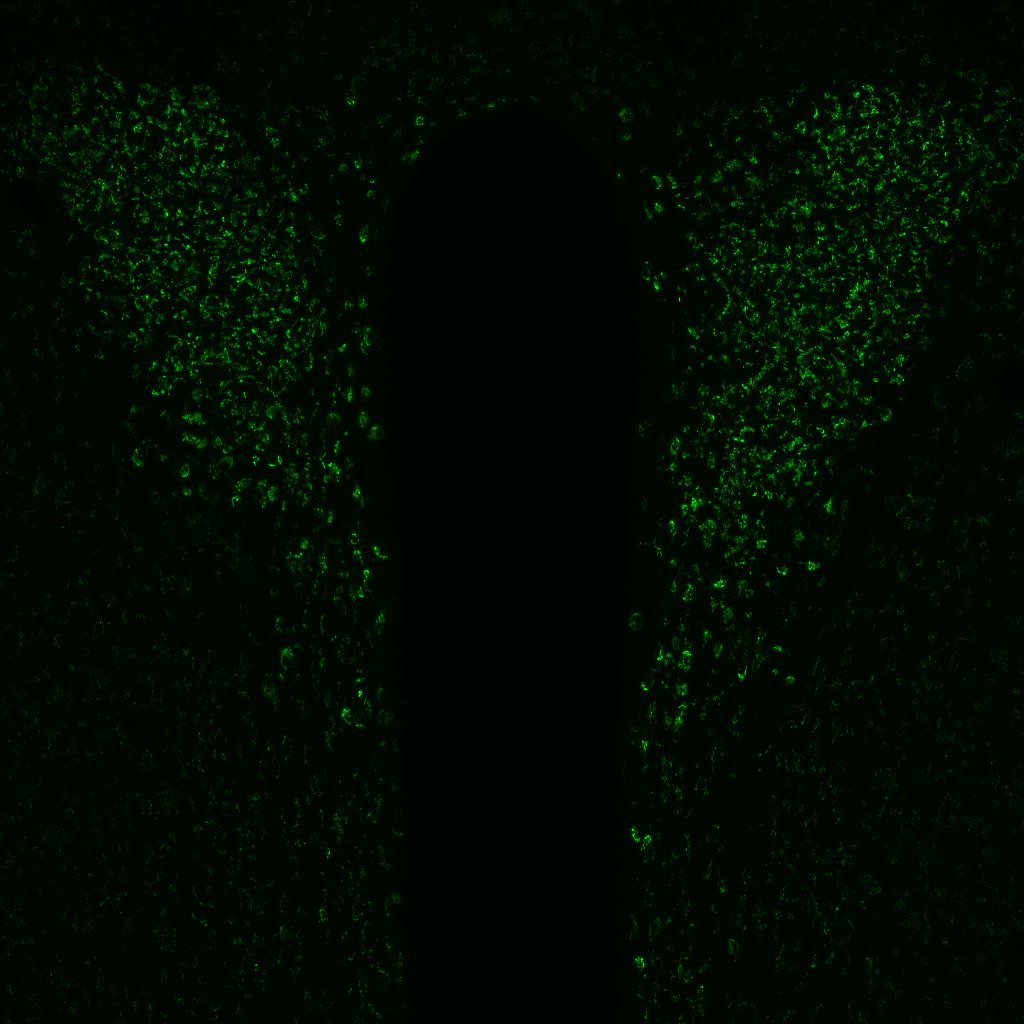

Supplement: Supplementary file 12 — Original data for Fig. 2a–d. [file 42255_2024_991_MOESM12_ESM.zip › Figure 2B/Mouse 13/1813-3 PVH.jpg]

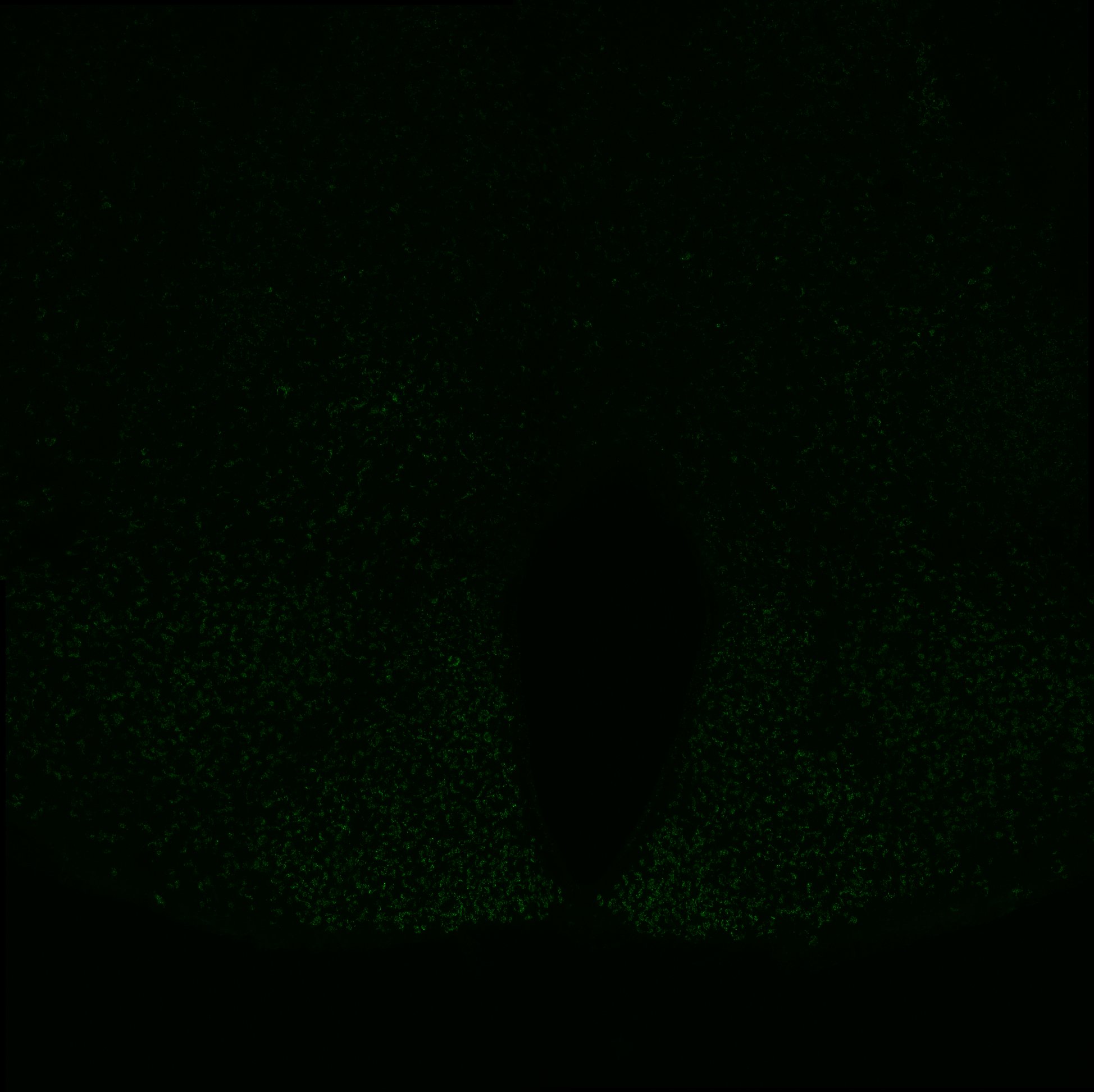

Supplement: Supplementary file 12 — Original data for Fig. 2a–d. [file 42255_2024_991_MOESM12_ESM.zip › Figure 2B/Mouse 14/1813-4 PostARH.jpg]

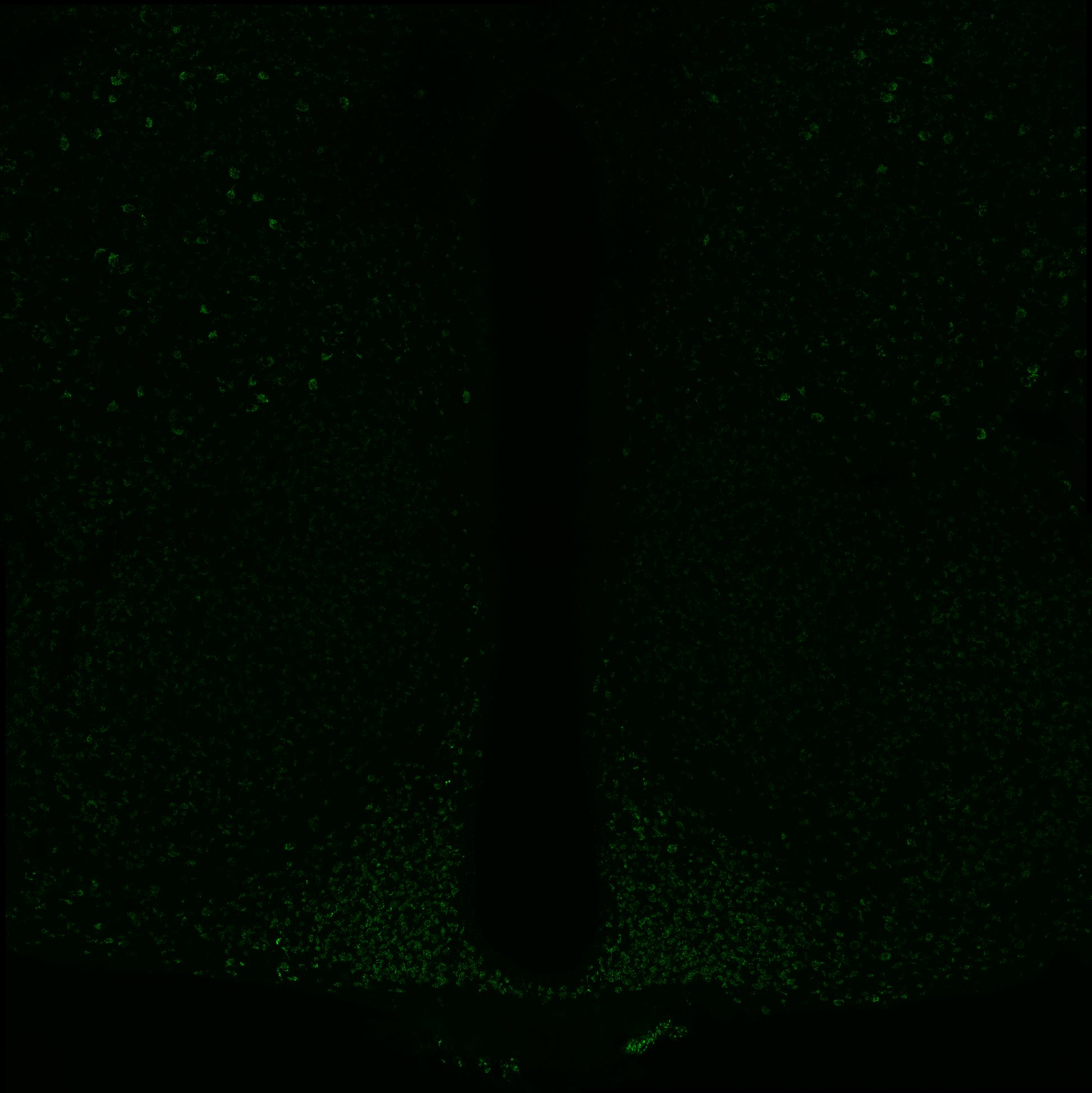

Supplement: Supplementary file 12 — Original data for Fig. 2a–d. [file 42255_2024_991_MOESM12_ESM.zip › Figure 2B/Mouse 14/1813-4 MidARH2.jpg]

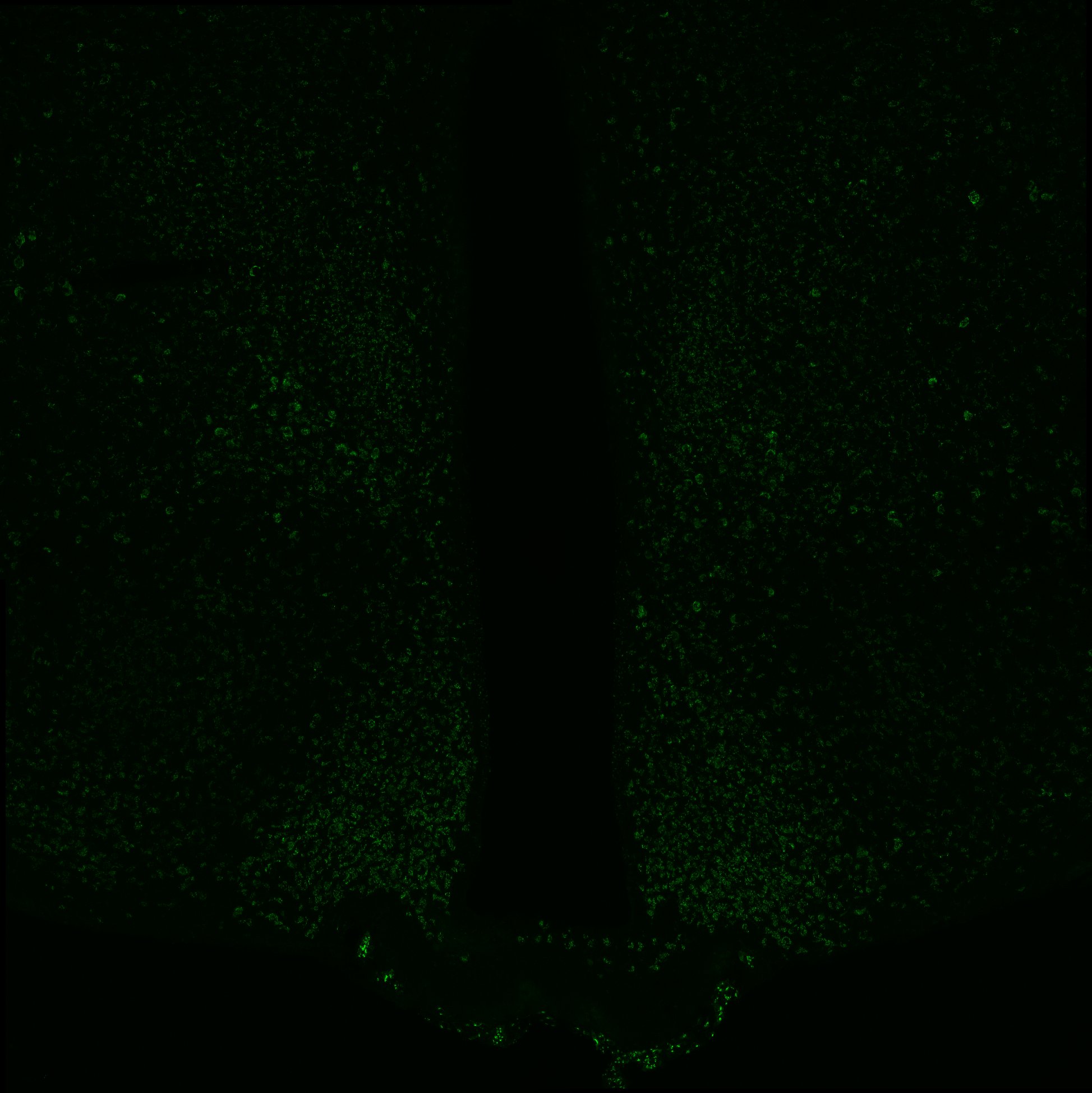

Supplement: Supplementary file 12 — Original data for Fig. 2a–d. [file 42255_2024_991_MOESM12_ESM.zip › Figure 2B/Mouse 14/1813-4 MidARH3.jpg]

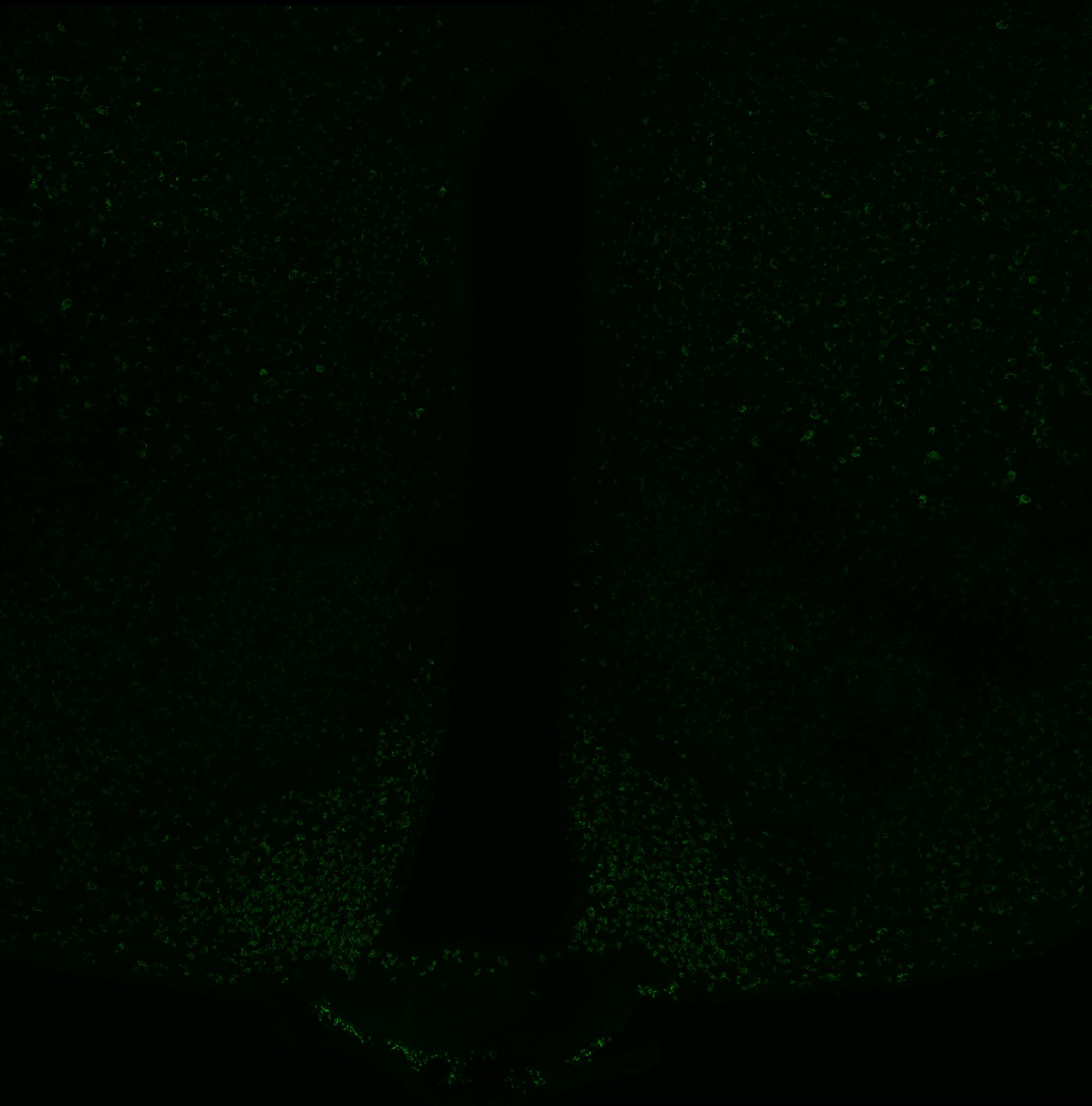

Supplement: Supplementary file 12 — Original data for Fig. 2a–d. [file 42255_2024_991_MOESM12_ESM.zip › Figure 2B/Mouse 14/1813-4 MidARH1.jpg]

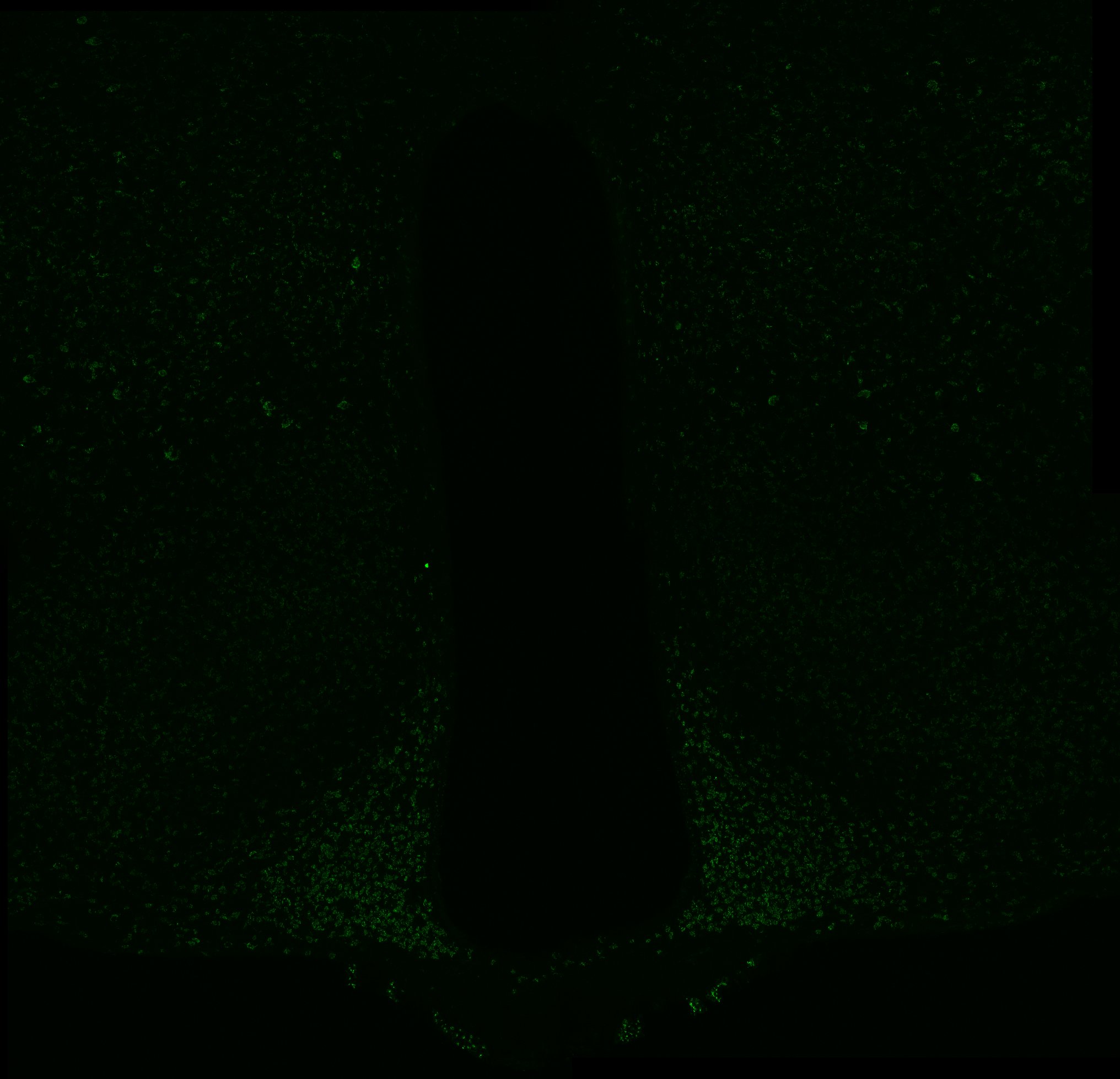

Supplement: Supplementary file 12 — Original data for Fig. 2a–d. [file 42255_2024_991_MOESM12_ESM.zip › Figure 2B/Mouse 15/1813-5 MidARH1.jpg]

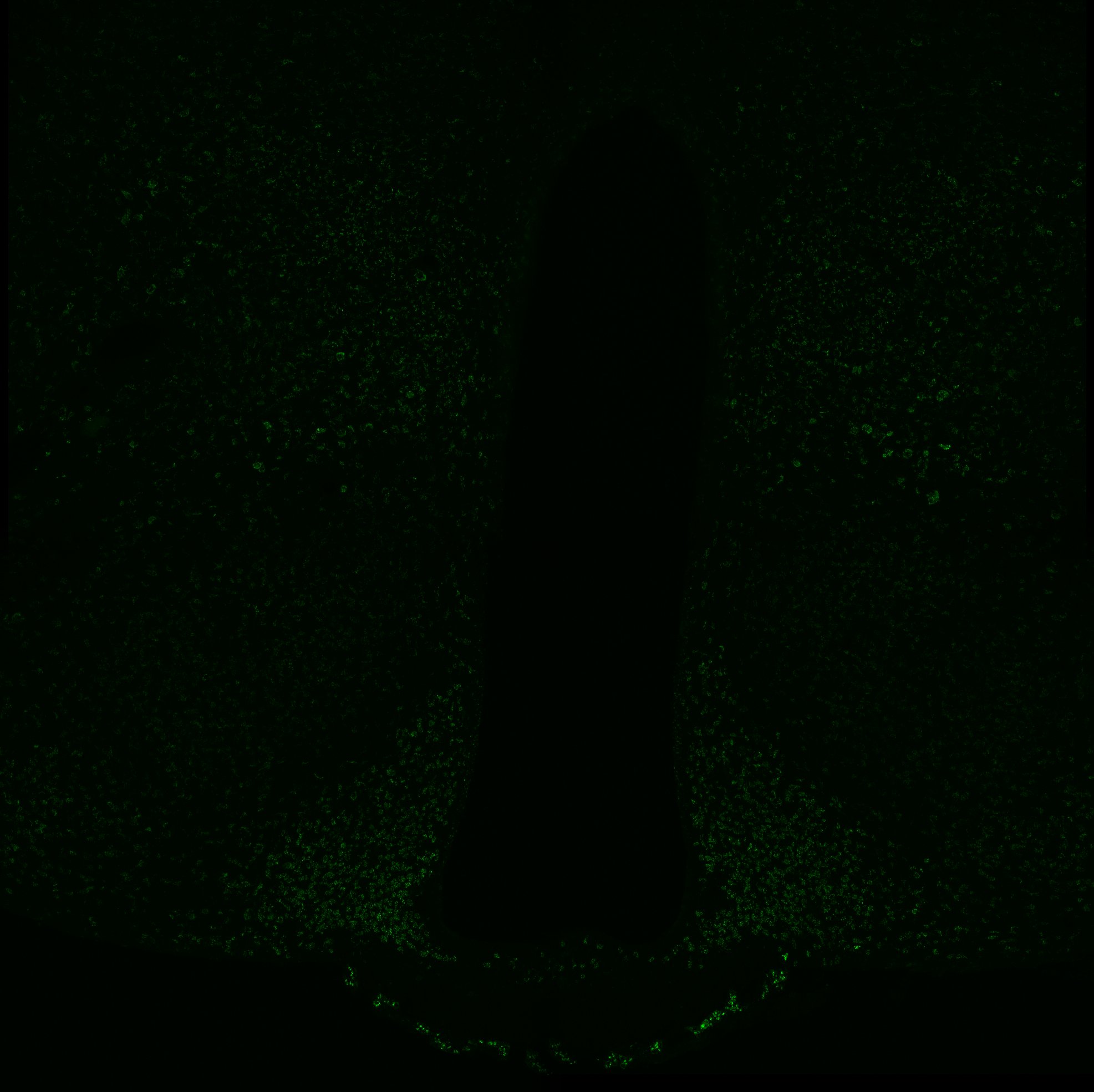

Supplement: Supplementary file 12 — Original data for Fig. 2a–d. [file 42255_2024_991_MOESM12_ESM.zip › Figure 2B/Mouse 15/1813-5 MidARH2.jpg]

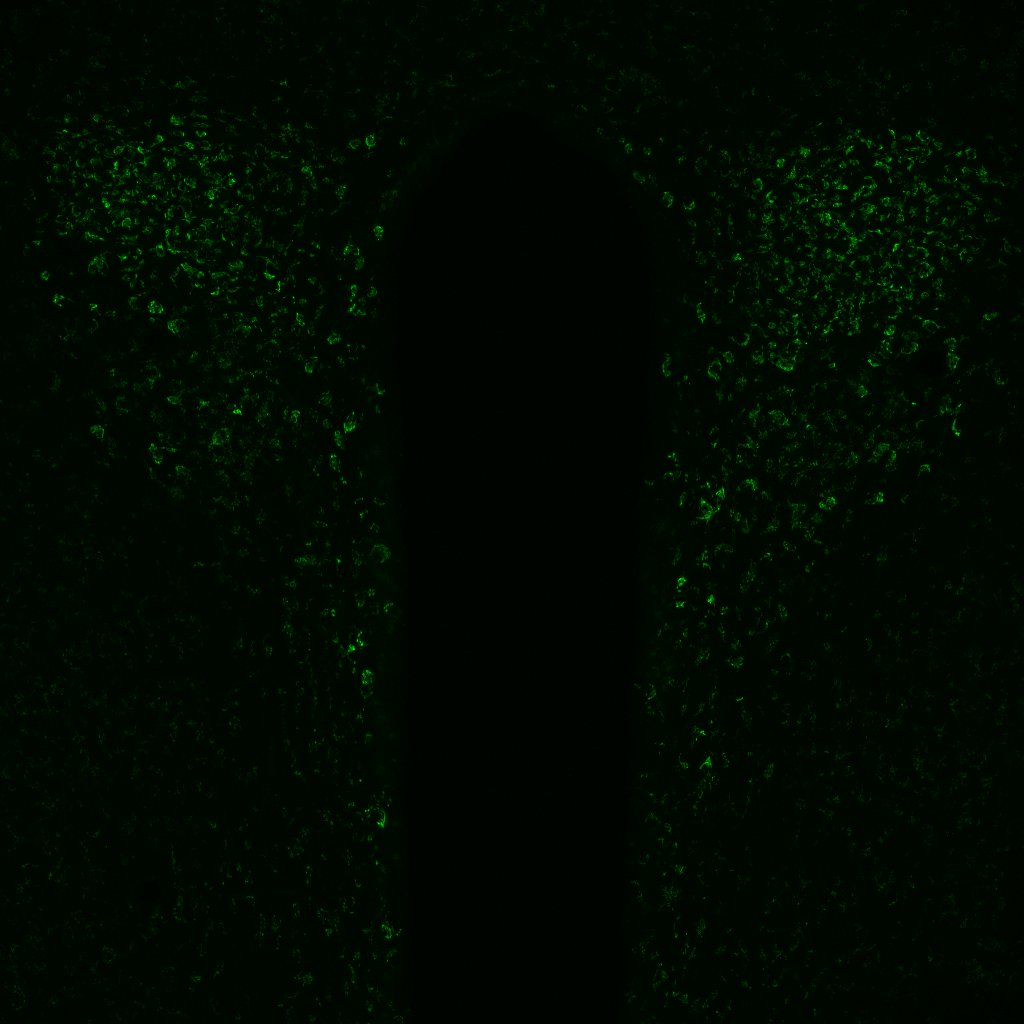

Supplement: Supplementary file 12 — Original data for Fig. 2a–d. [file 42255_2024_991_MOESM12_ESM.zip › Figure 2B/Mouse 15/1813-5 PVH.jpg]

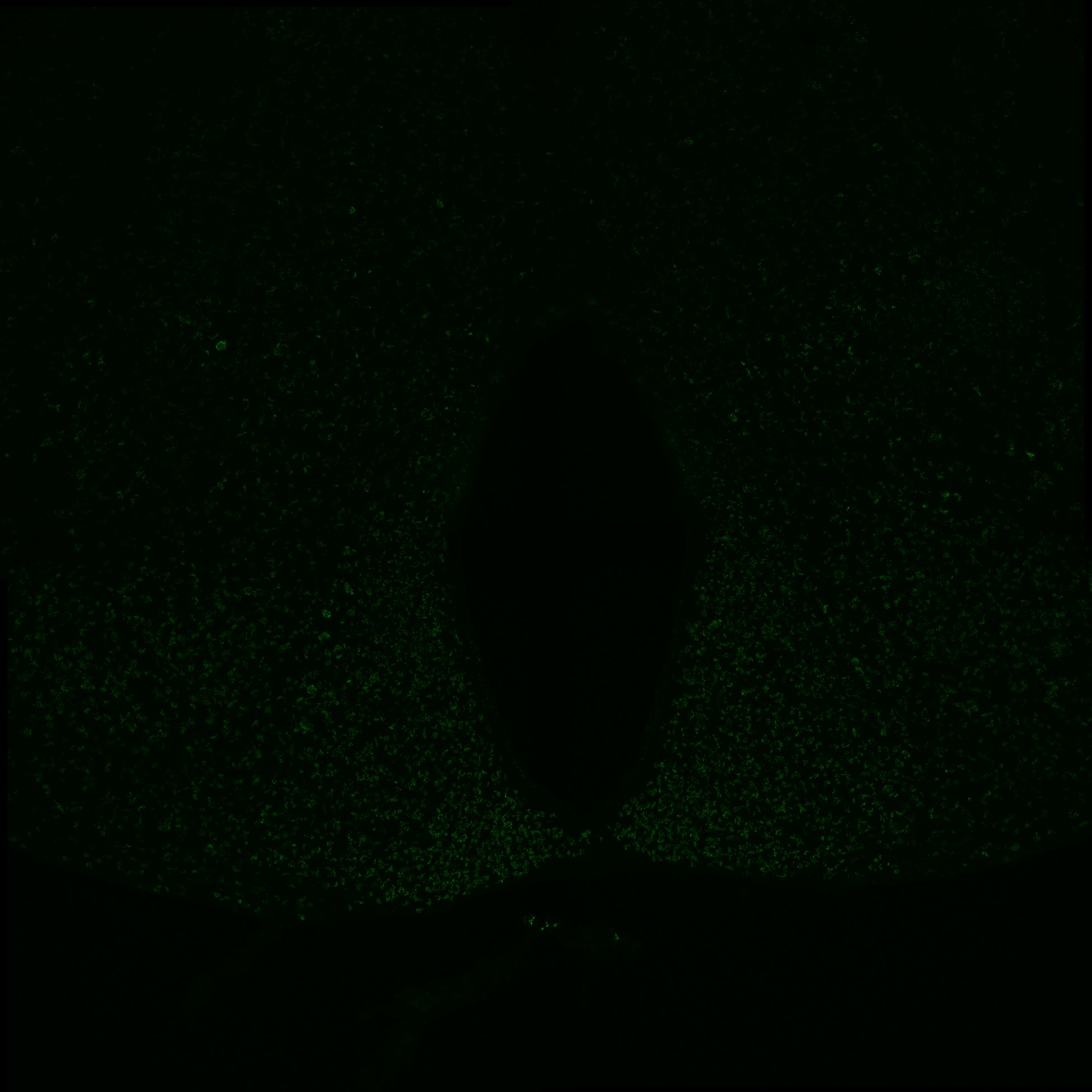

Supplement: Supplementary file 12 — Original data for Fig. 2a–d. [file 42255_2024_991_MOESM12_ESM.zip › Figure 2B/Mouse 15/1813-5 PostARH.jpg]

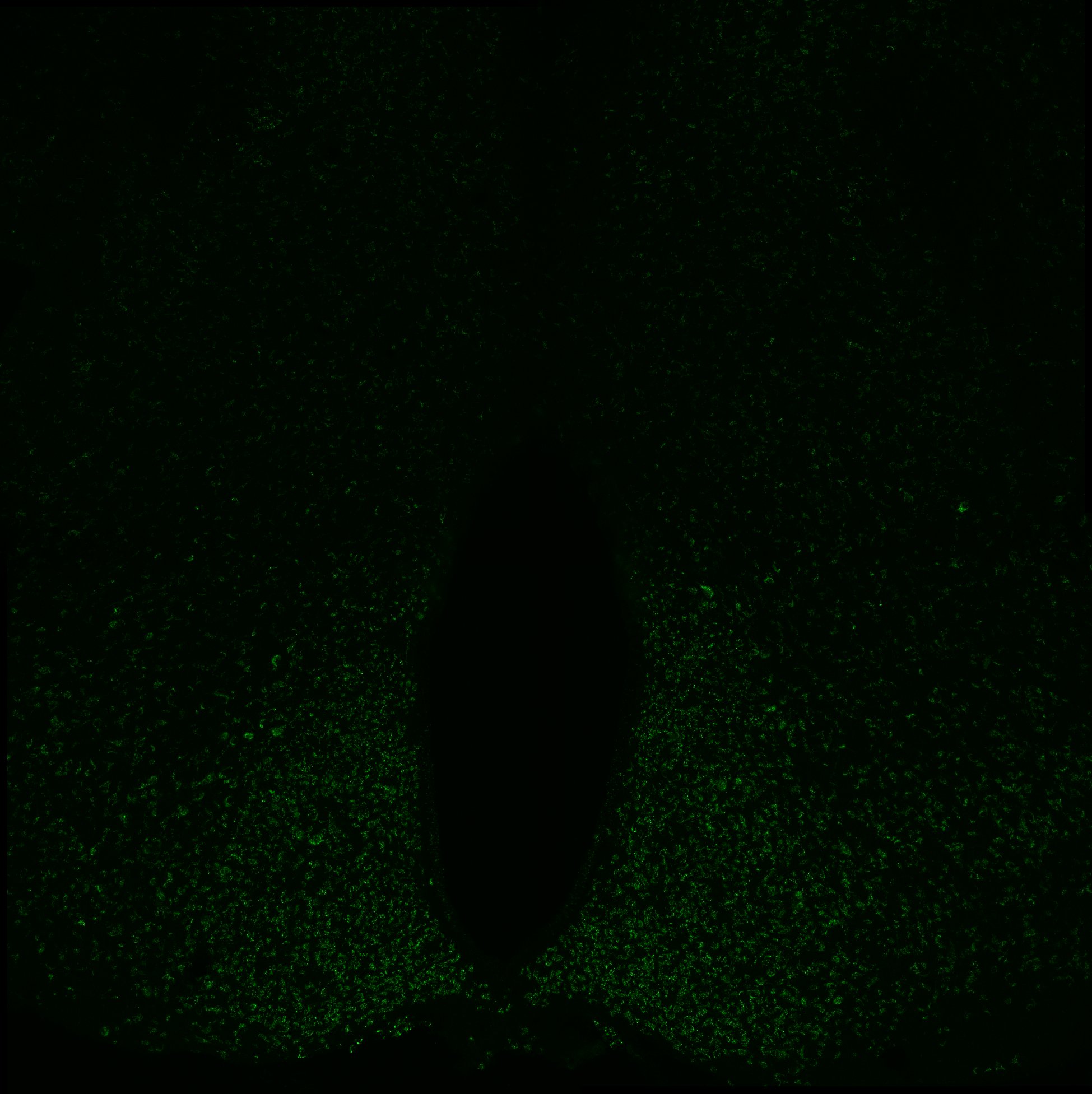

Supplement: Supplementary file 12 — Original data for Fig. 2a–d. [file 42255_2024_991_MOESM12_ESM.zip › Figure 2B/Mouse 12/1813-2 PostARH.jpg]

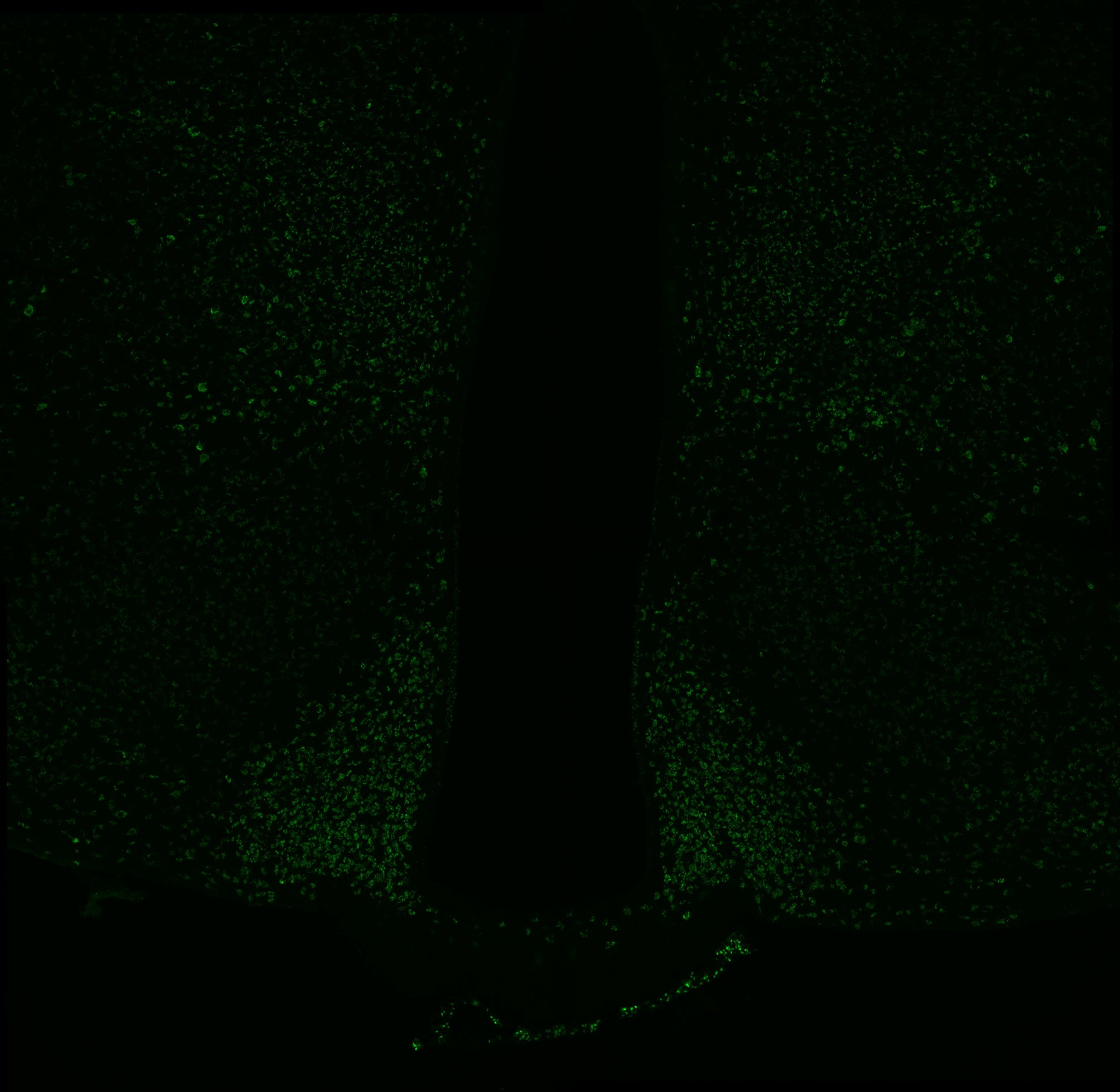

Supplement: Supplementary file 12 — Original data for Fig. 2a–d. [file 42255_2024_991_MOESM12_ESM.zip › Figure 2B/Mouse 12/1813-2 MidARH2.jpg]

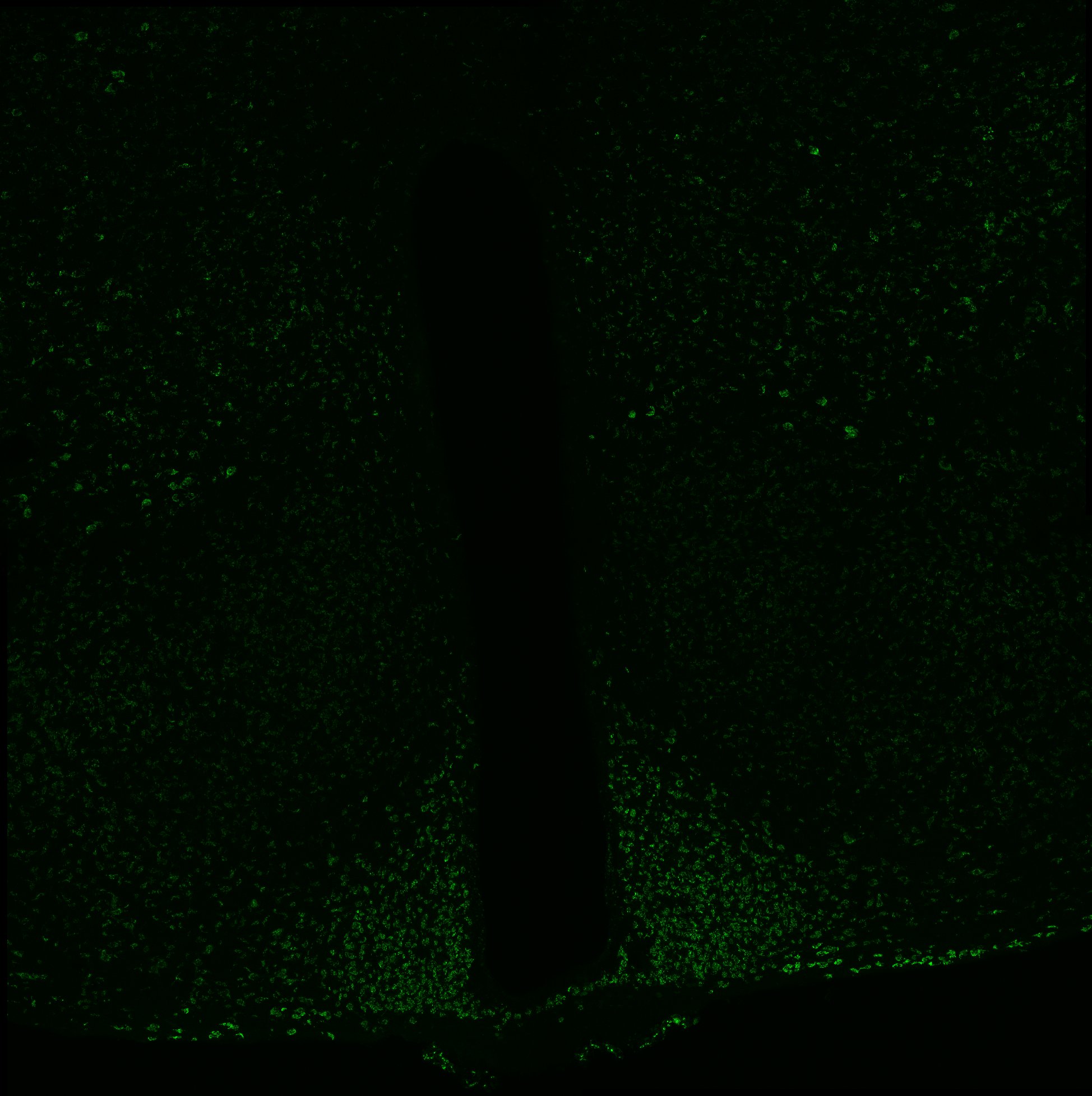

Supplement: Supplementary file 12 — Original data for Fig. 2a–d. [file 42255_2024_991_MOESM12_ESM.zip › Figure 2B/Mouse 12/1813-2 MidARH1.jpg]

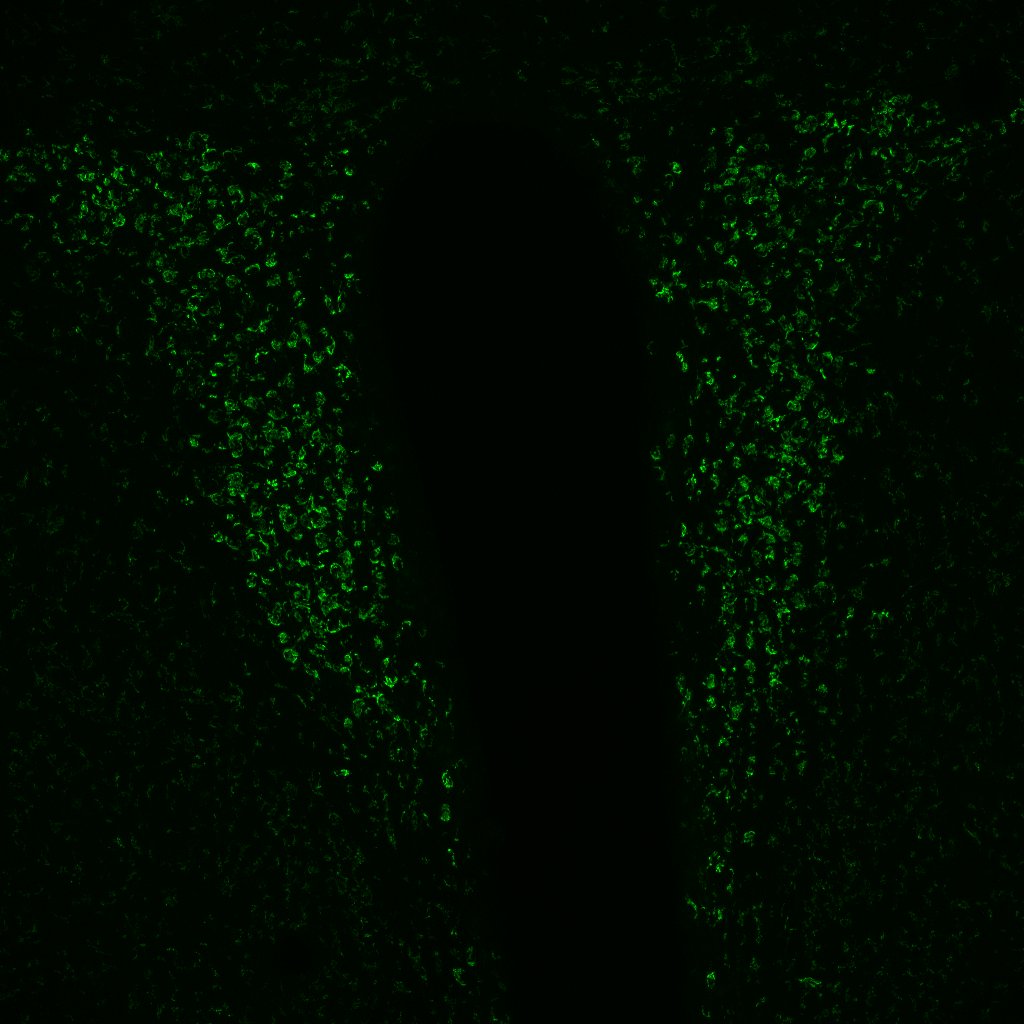

Supplement: Supplementary file 12 — Original data for Fig. 2a–d. [file 42255_2024_991_MOESM12_ESM.zip › Figure 2B/Mouse 12/1813-2 PVH.jpg]

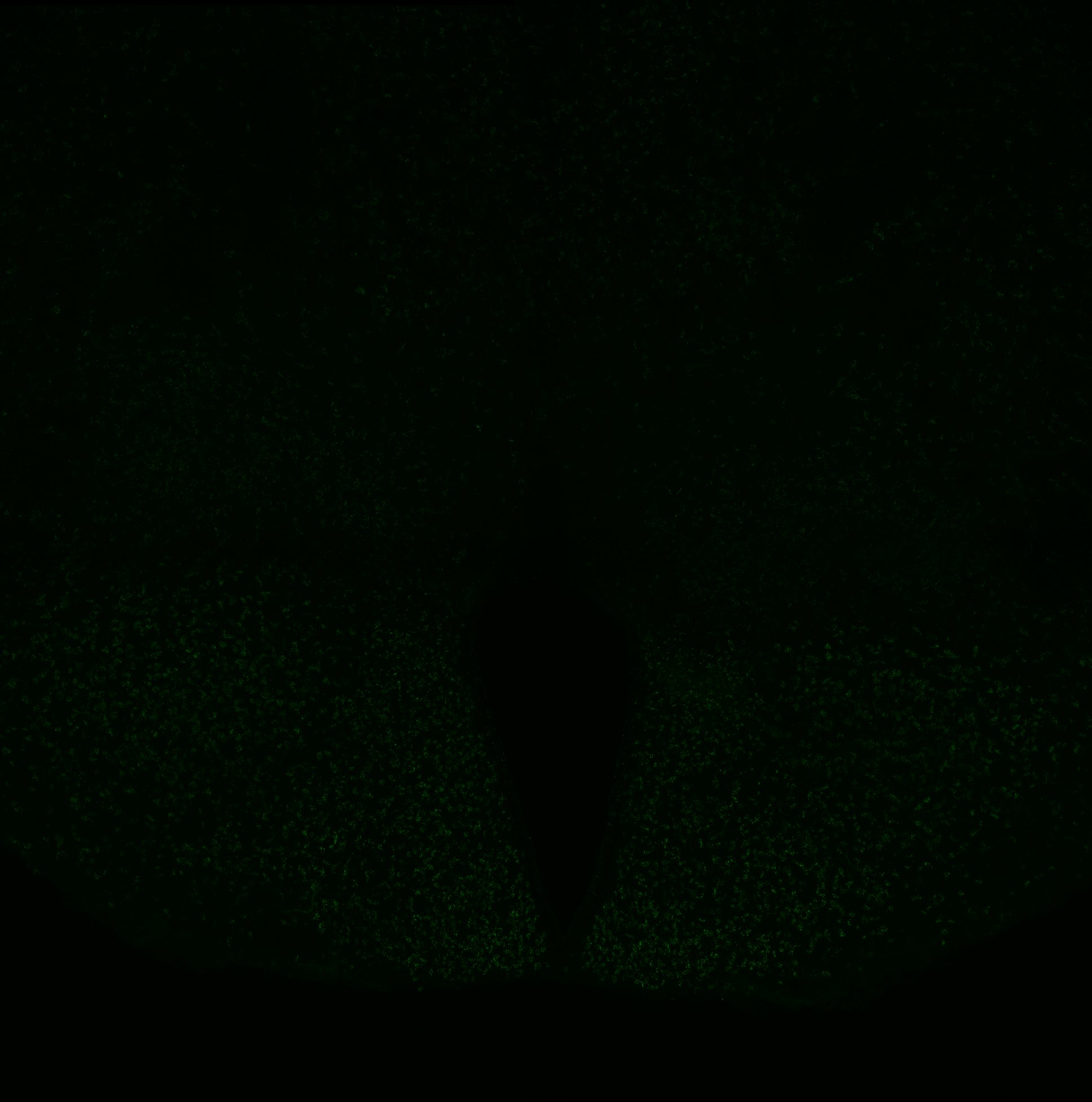

Supplement: Supplementary file 12 — Original data for Fig. 2a–d. [file 42255_2024_991_MOESM12_ESM.zip › Figure 2B/Mouse 24/1814-4 PostARH.jpg]

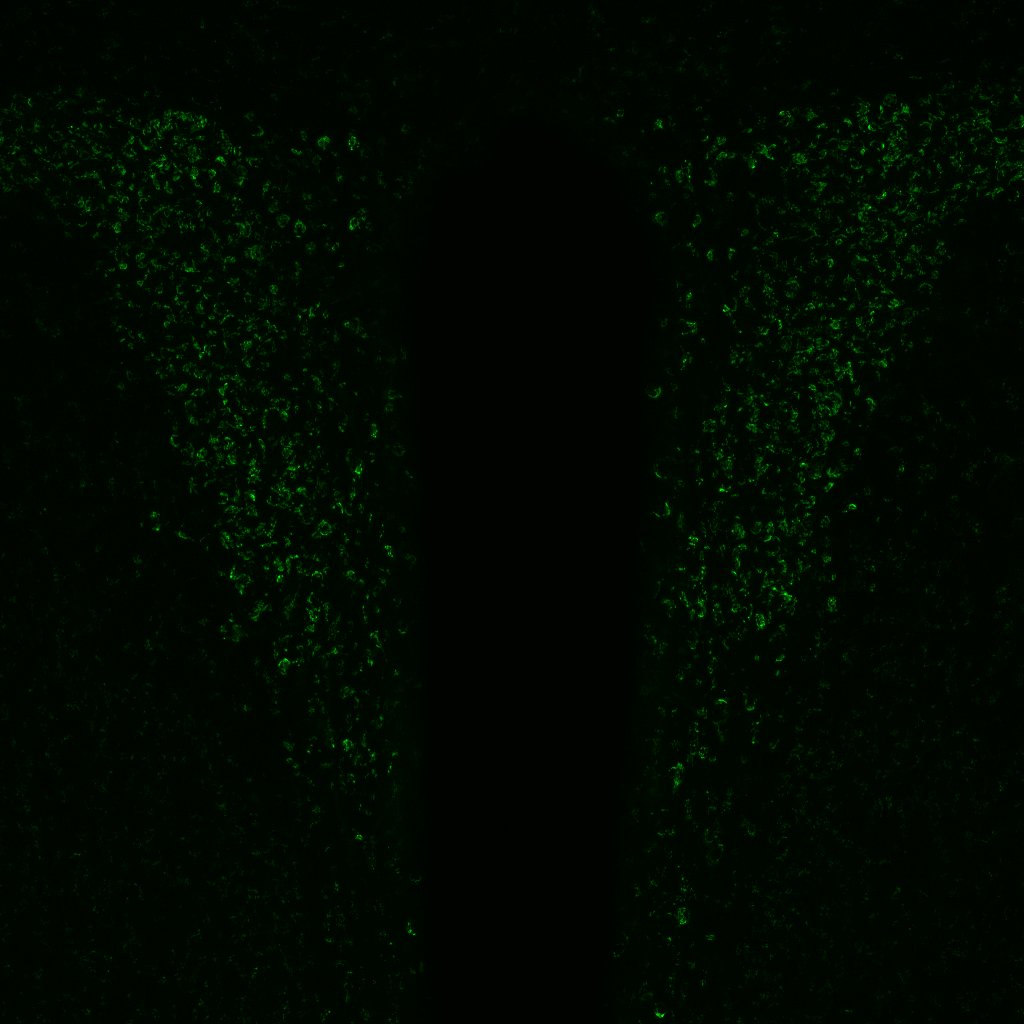

Supplement: Supplementary file 12 — Original data for Fig. 2a–d. [file 42255_2024_991_MOESM12_ESM.zip › Figure 2B/Mouse 24/1814-4 PVH.jpg]

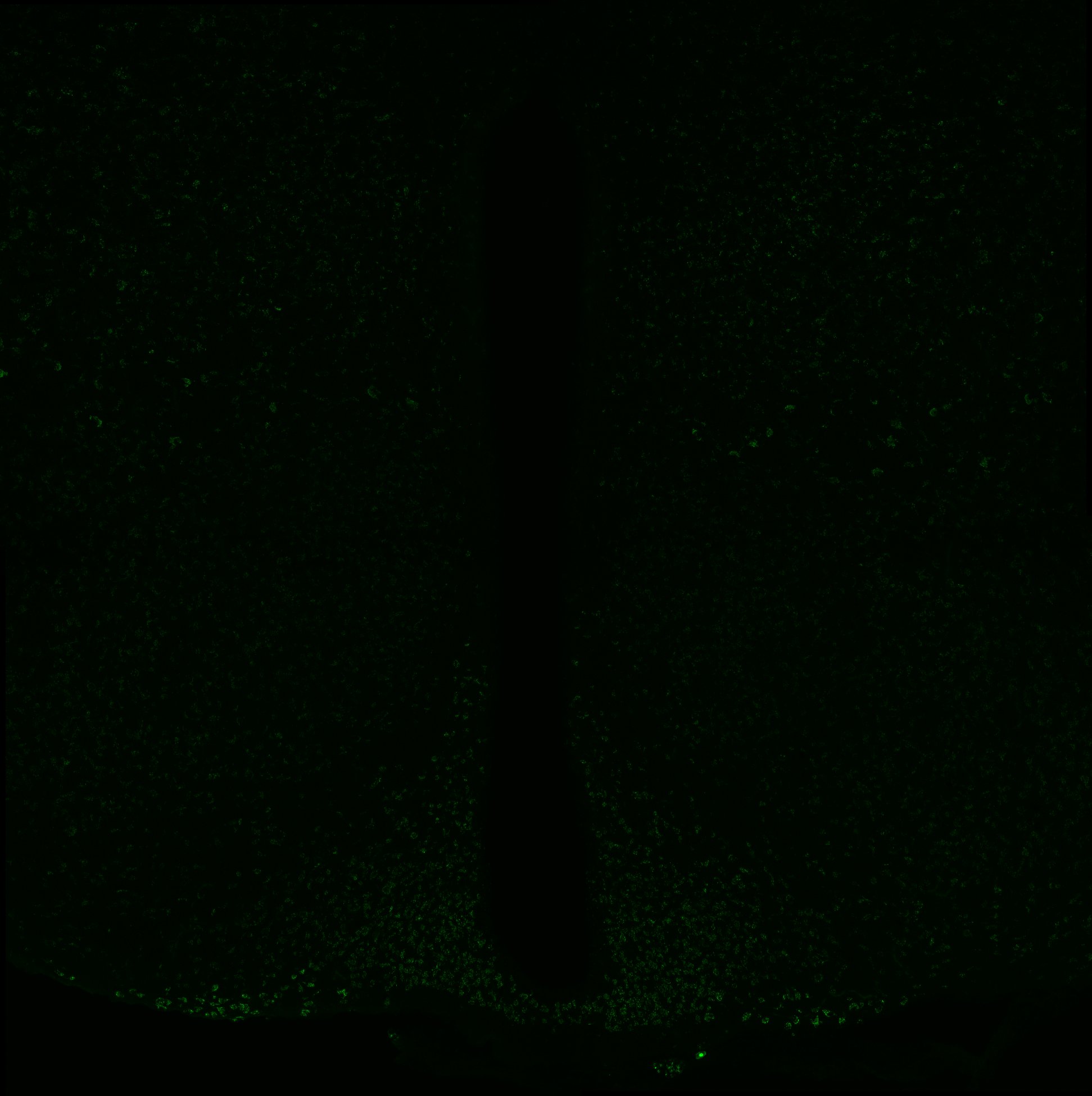

Supplement: Supplementary file 12 — Original data for Fig. 2a–d. [file 42255_2024_991_MOESM12_ESM.zip › Figure 2B/Mouse 24/1814-4 MidARH1.jpg]

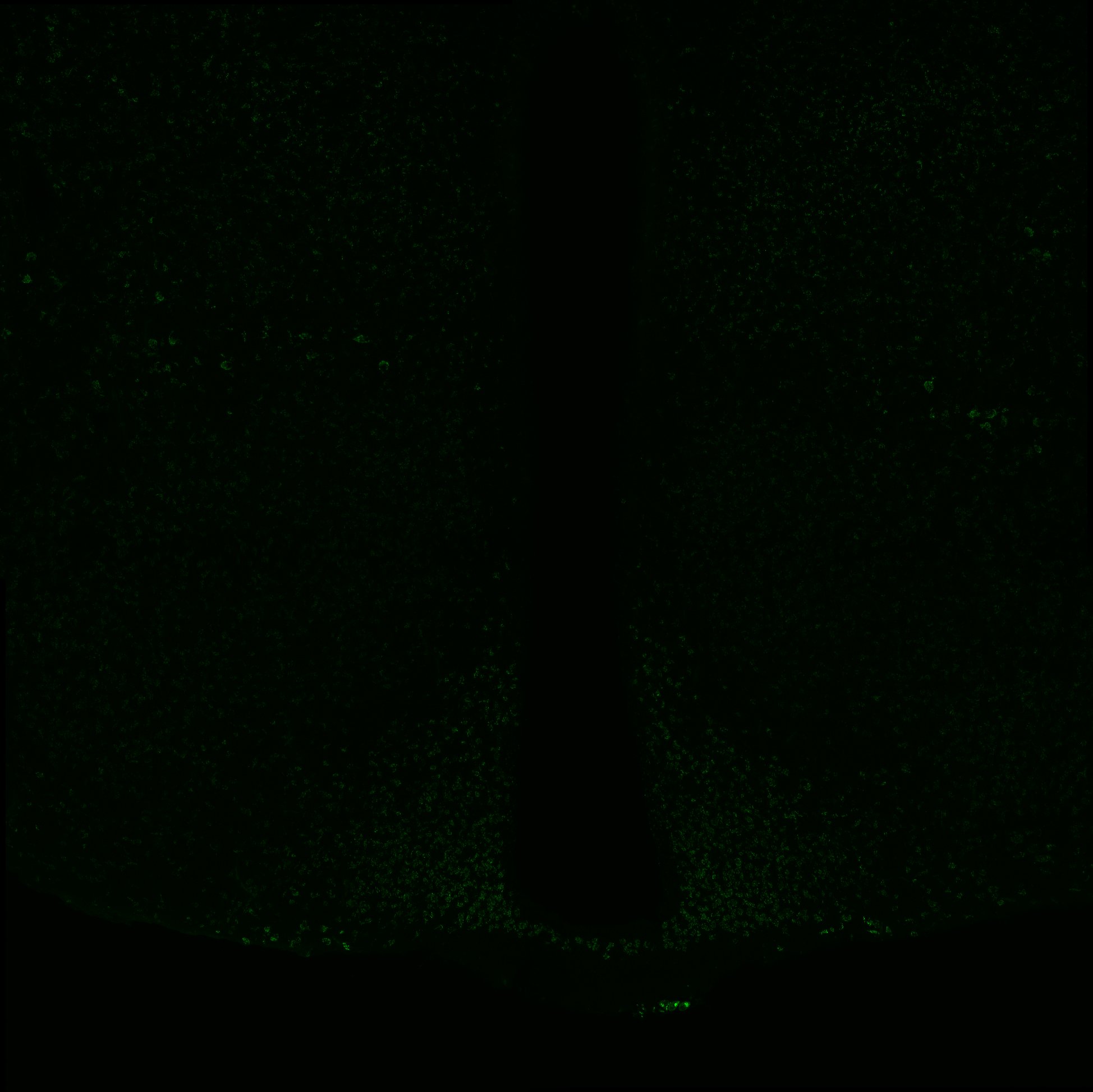

Supplement: Supplementary file 12 — Original data for Fig. 2a–d. [file 42255_2024_991_MOESM12_ESM.zip › Figure 2B/Mouse 24/1814-4 MidARH3.jpg]

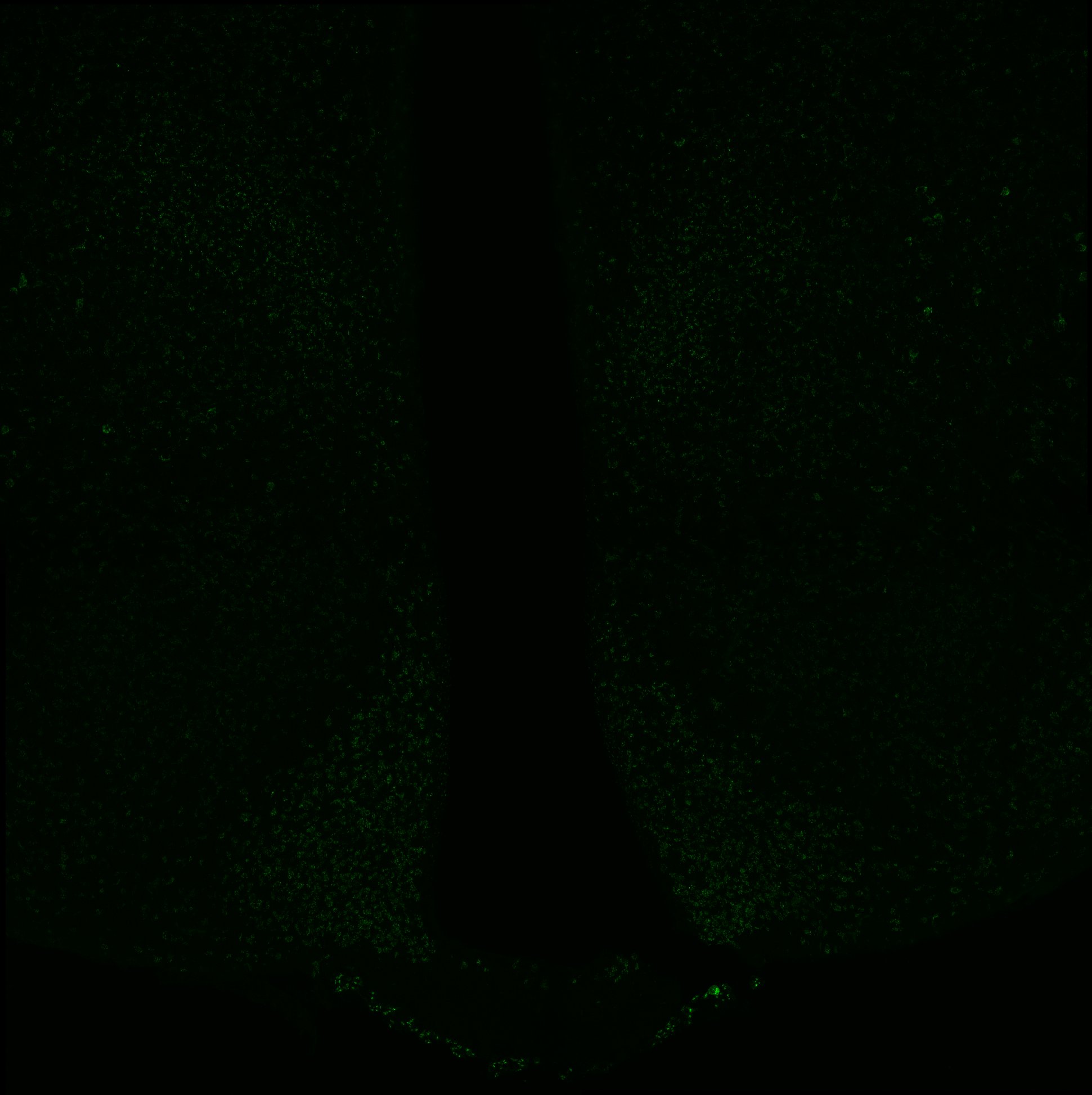

Supplement: Supplementary file 12 — Original data for Fig. 2a–d. [file 42255_2024_991_MOESM12_ESM.zip › Figure 2B/Mouse 24/1814-4 MidARH2.jpg]

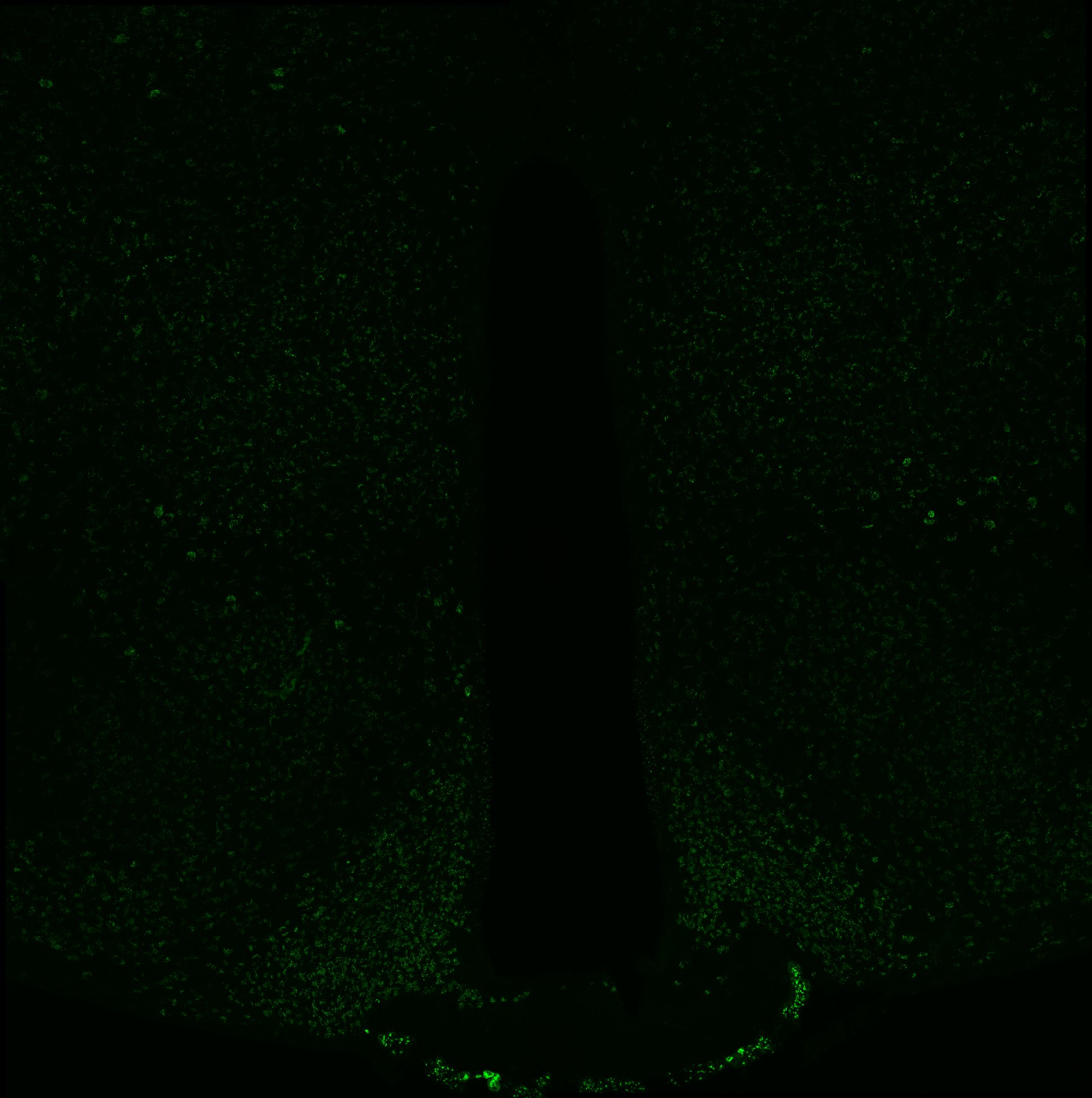

Supplement: Supplementary file 12 — Original data for Fig. 2a–d. [file 42255_2024_991_MOESM12_ESM.zip › Figure 2B/Mouse 23/1814-3 MidARH2.jpg]

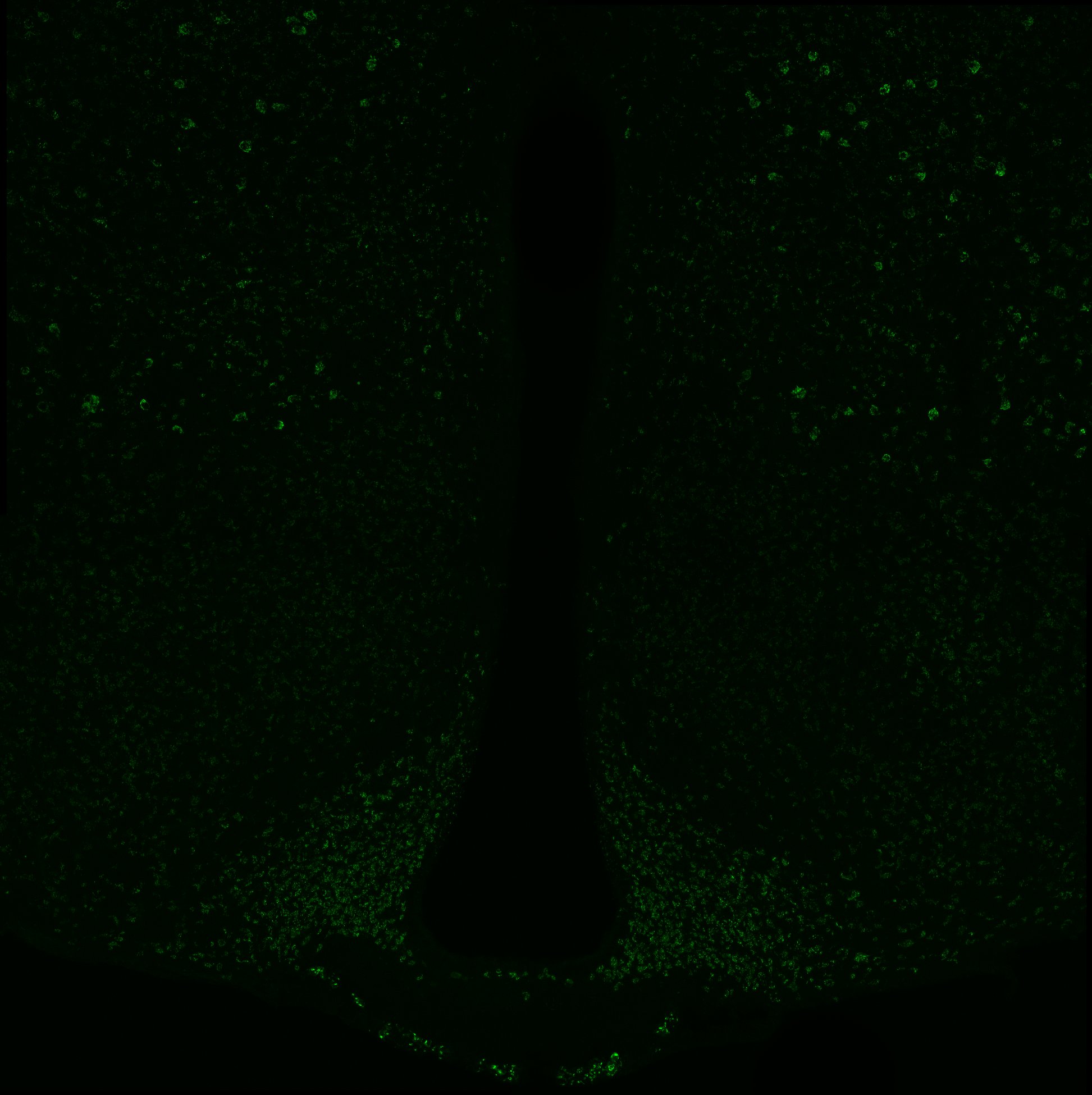

Supplement: Supplementary file 12 — Original data for Fig. 2a–d. [file 42255_2024_991_MOESM12_ESM.zip › Figure 2B/Mouse 23/1814-3 MidARH1.jpg]

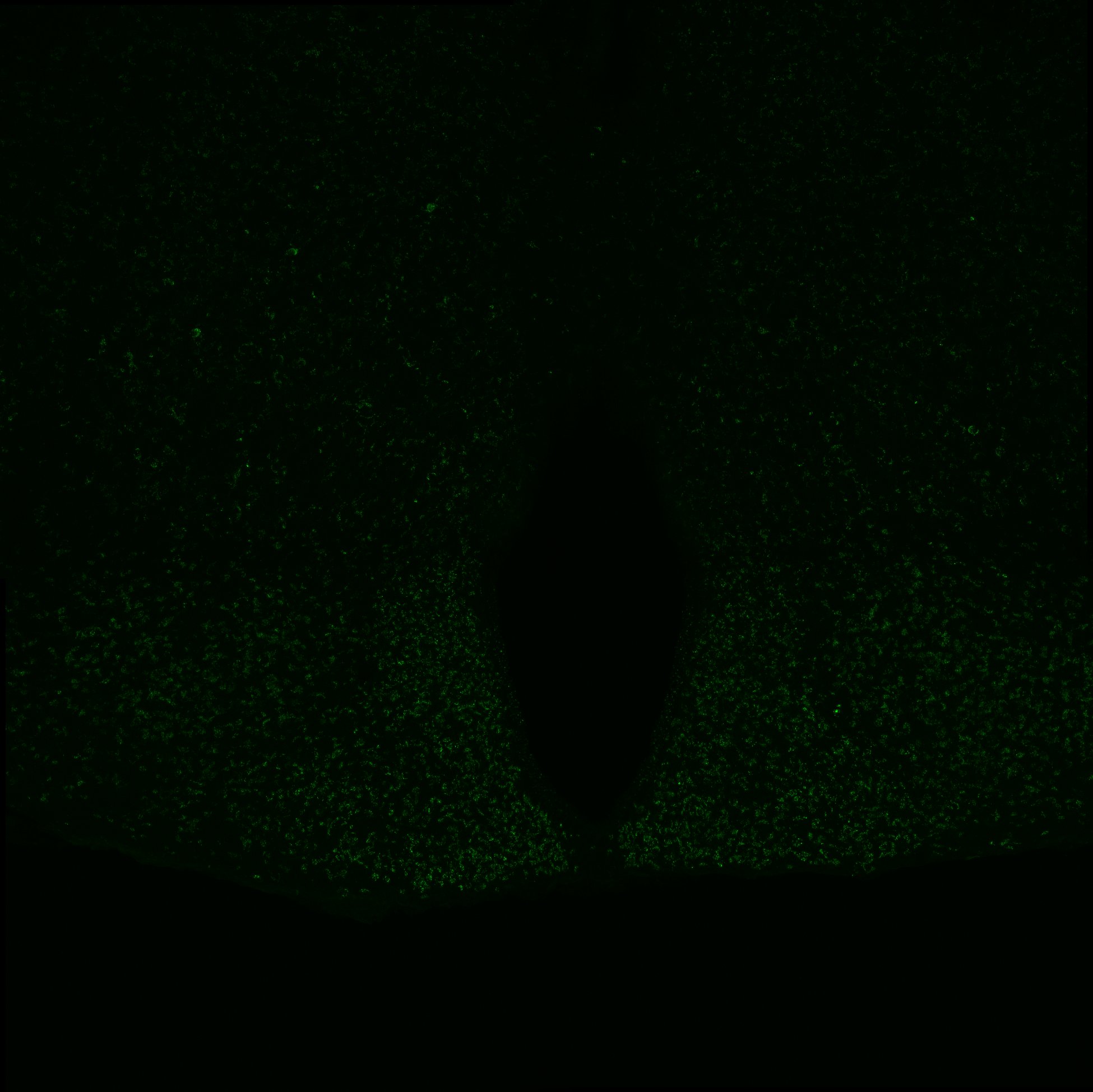

Supplement: Supplementary file 12 — Original data for Fig. 2a–d. [file 42255_2024_991_MOESM12_ESM.zip › Figure 2B/Mouse 23/1814-3 PostARH.jpg]

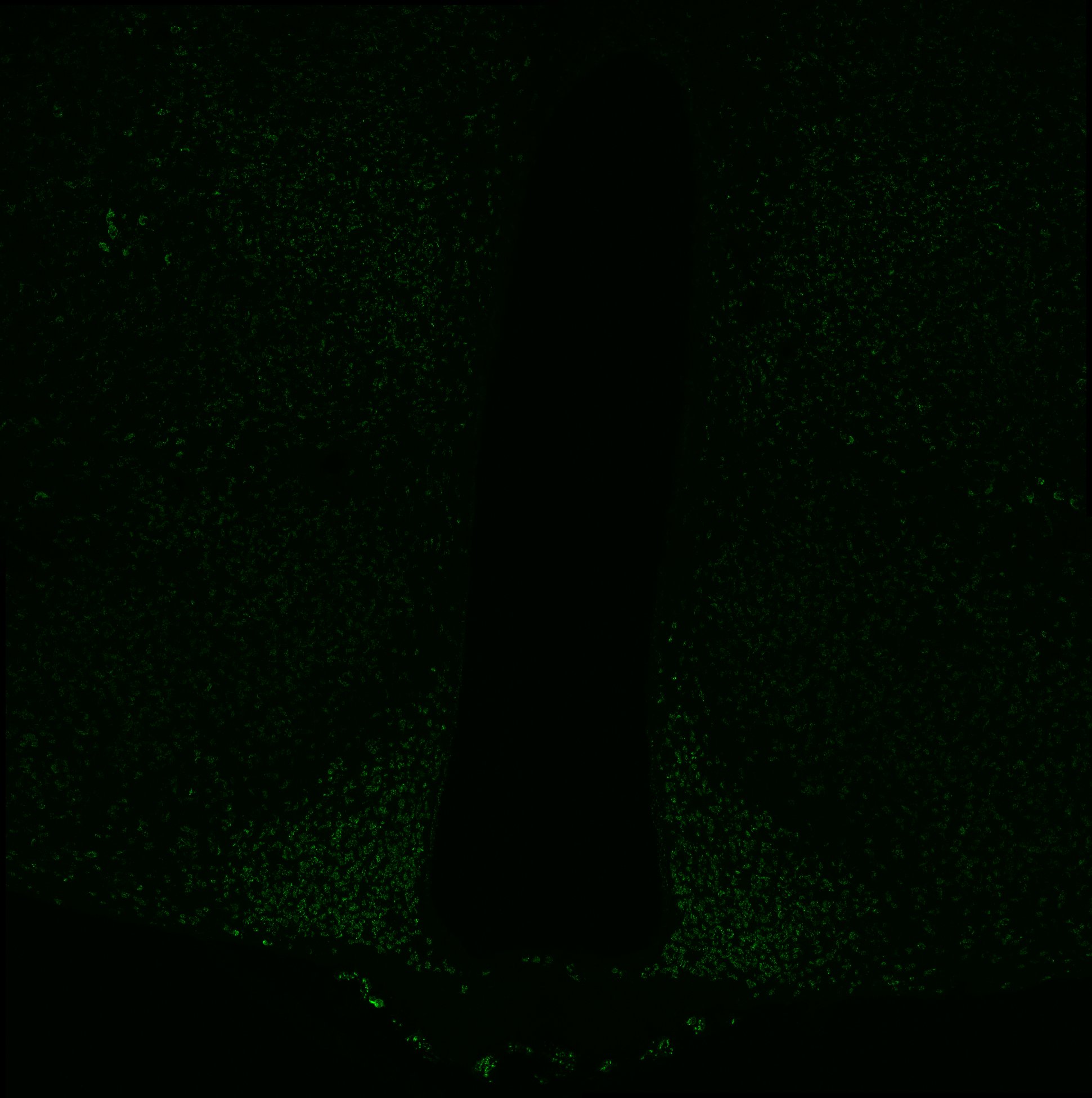

Supplement: Supplementary file 12 — Original data for Fig. 2a–d. [file 42255_2024_991_MOESM12_ESM.zip › Figure 2B/Mouse 3/1830-3 MidARH1.jpg]

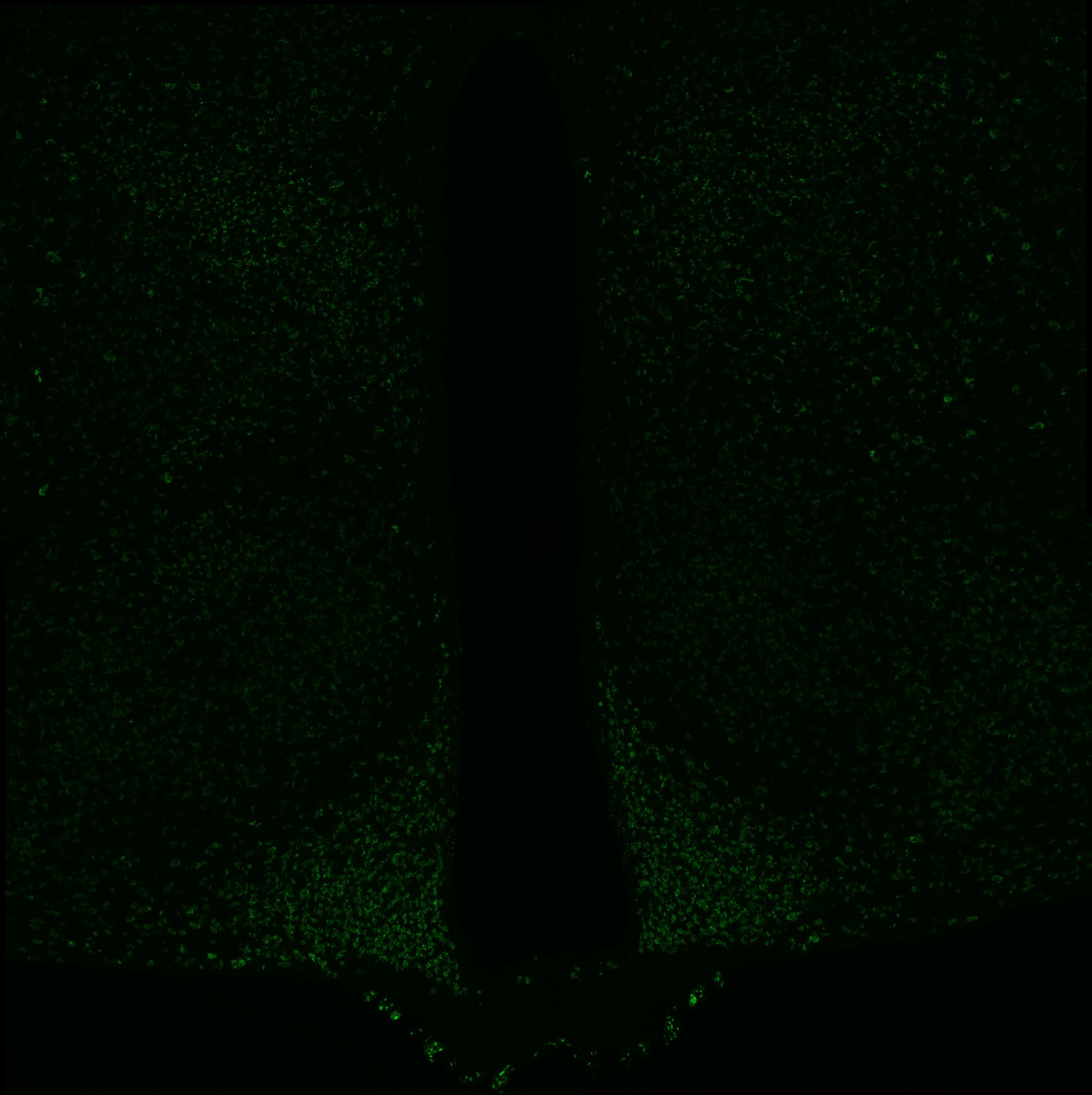

Supplement: Supplementary file 12 — Original data for Fig. 2a–d. [file 42255_2024_991_MOESM12_ESM.zip › Figure 2B/Mouse 3/1830-3 MidARH2.jpg]

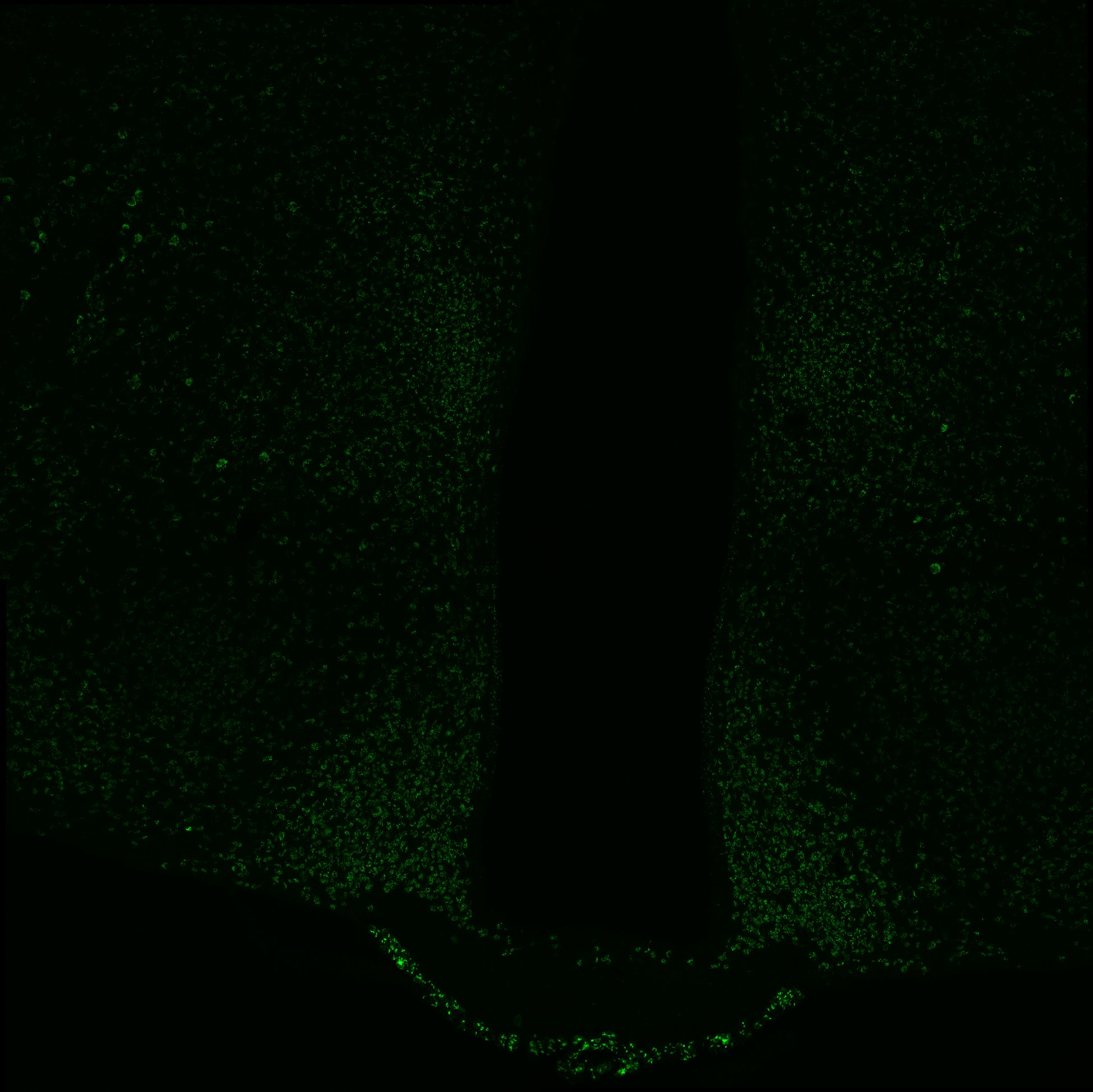

Supplement: Supplementary file 12 — Original data for Fig. 2a–d. [file 42255_2024_991_MOESM12_ESM.zip › Figure 2B/Mouse 3/1830-3 MidARH3.jpg]

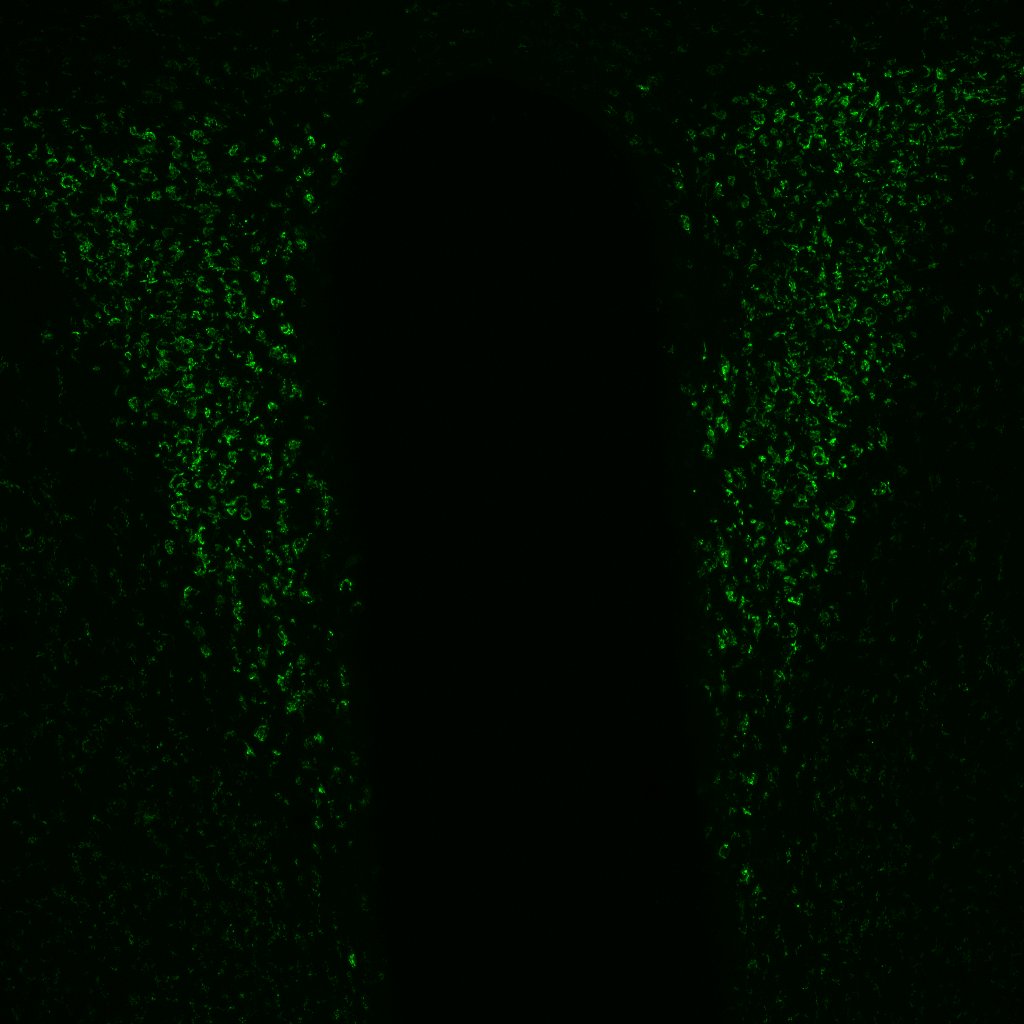

Supplement: Supplementary file 12 — Original data for Fig. 2a–d. [file 42255_2024_991_MOESM12_ESM.zip › Figure 2B/Mouse 3/1830-3 PVH.jpg]

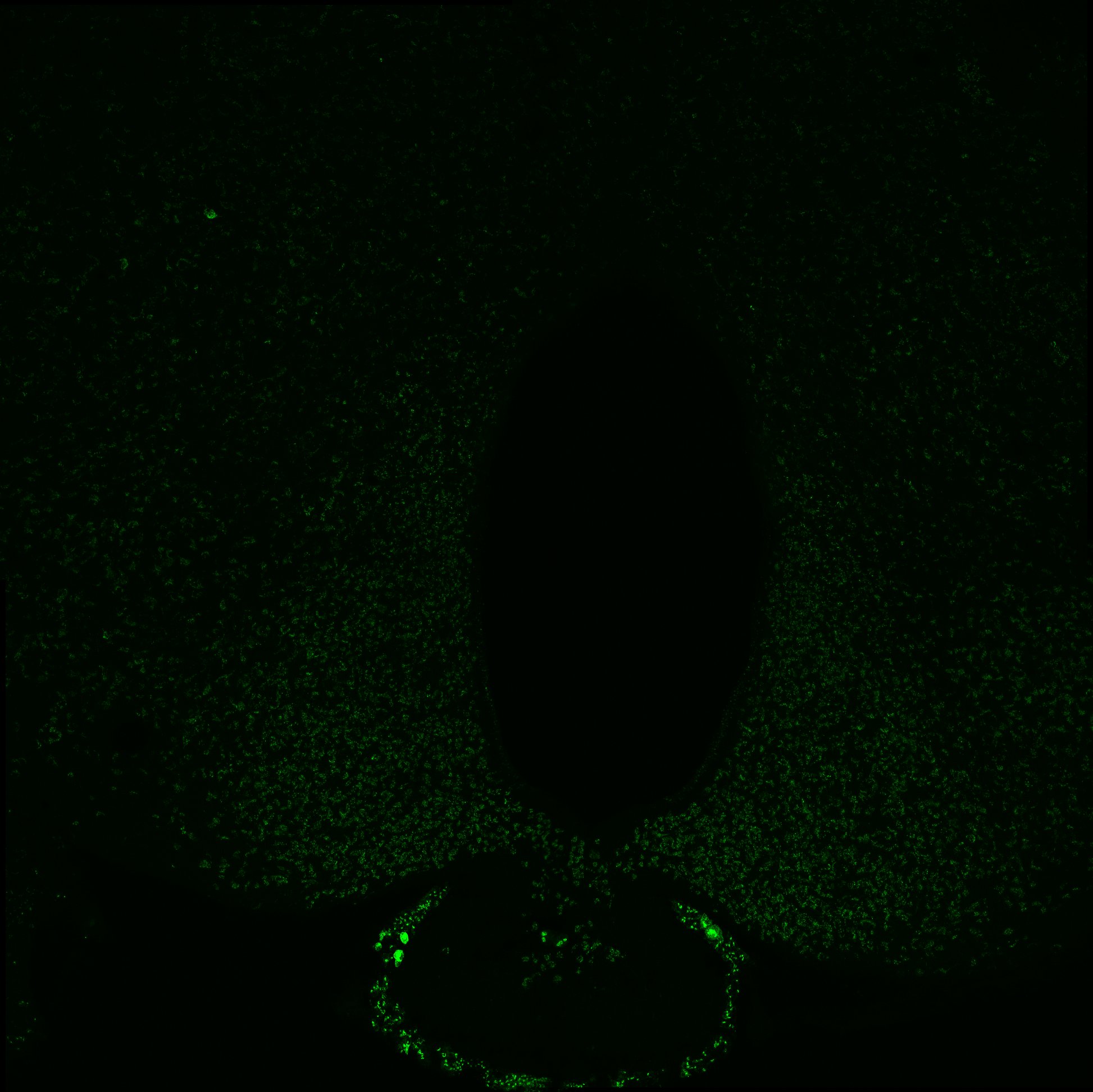

Supplement: Supplementary file 12 — Original data for Fig. 2a–d. [file 42255_2024_991_MOESM12_ESM.zip › Figure 2B/Mouse 3/1830-3 PostARH.jpg]

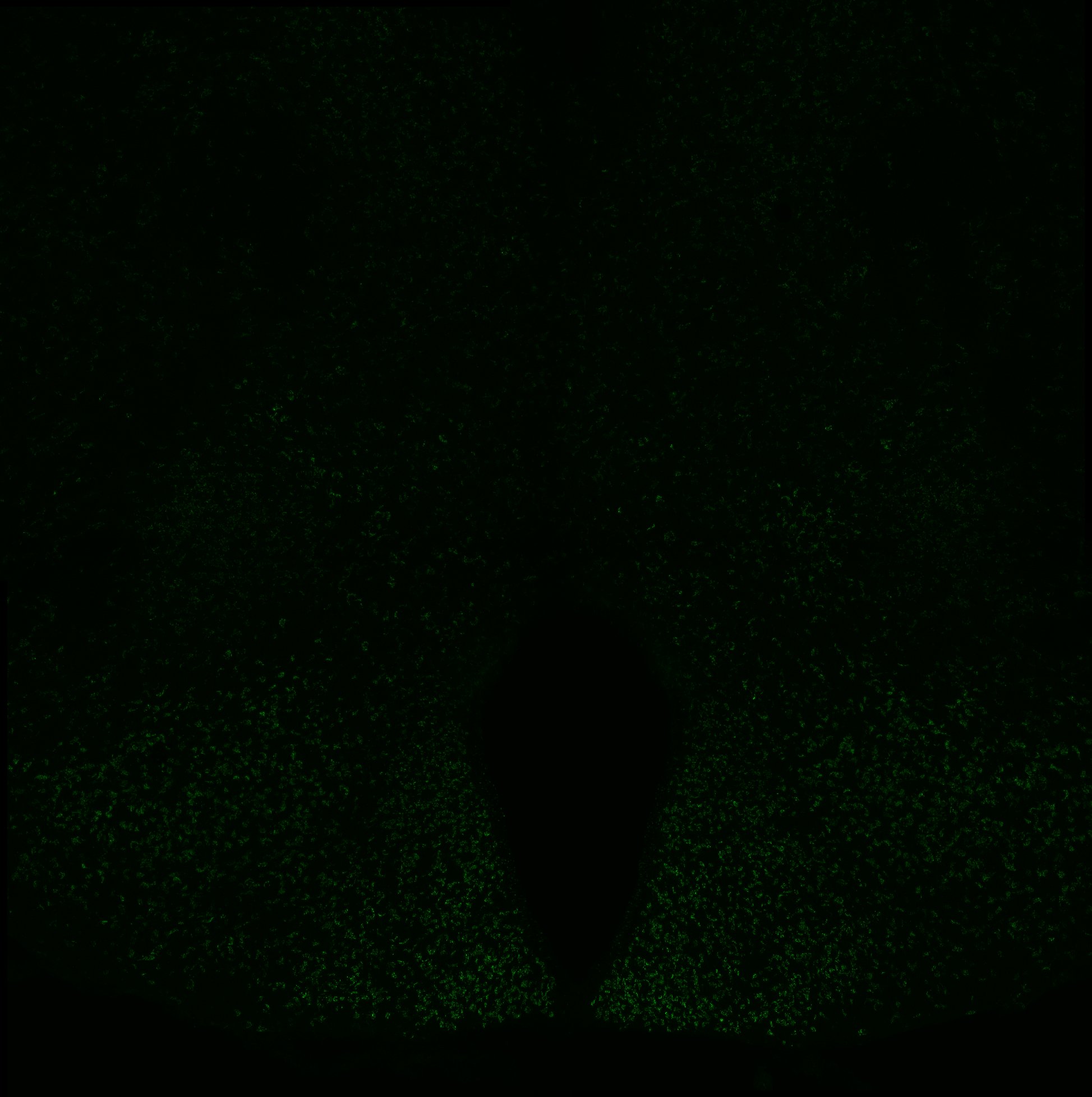

Supplement: Supplementary file 12 — Original data for Fig. 2a–d. [file 42255_2024_991_MOESM12_ESM.zip › Figure 2B/Mouse 4/1830-4 PostARH.jpg]

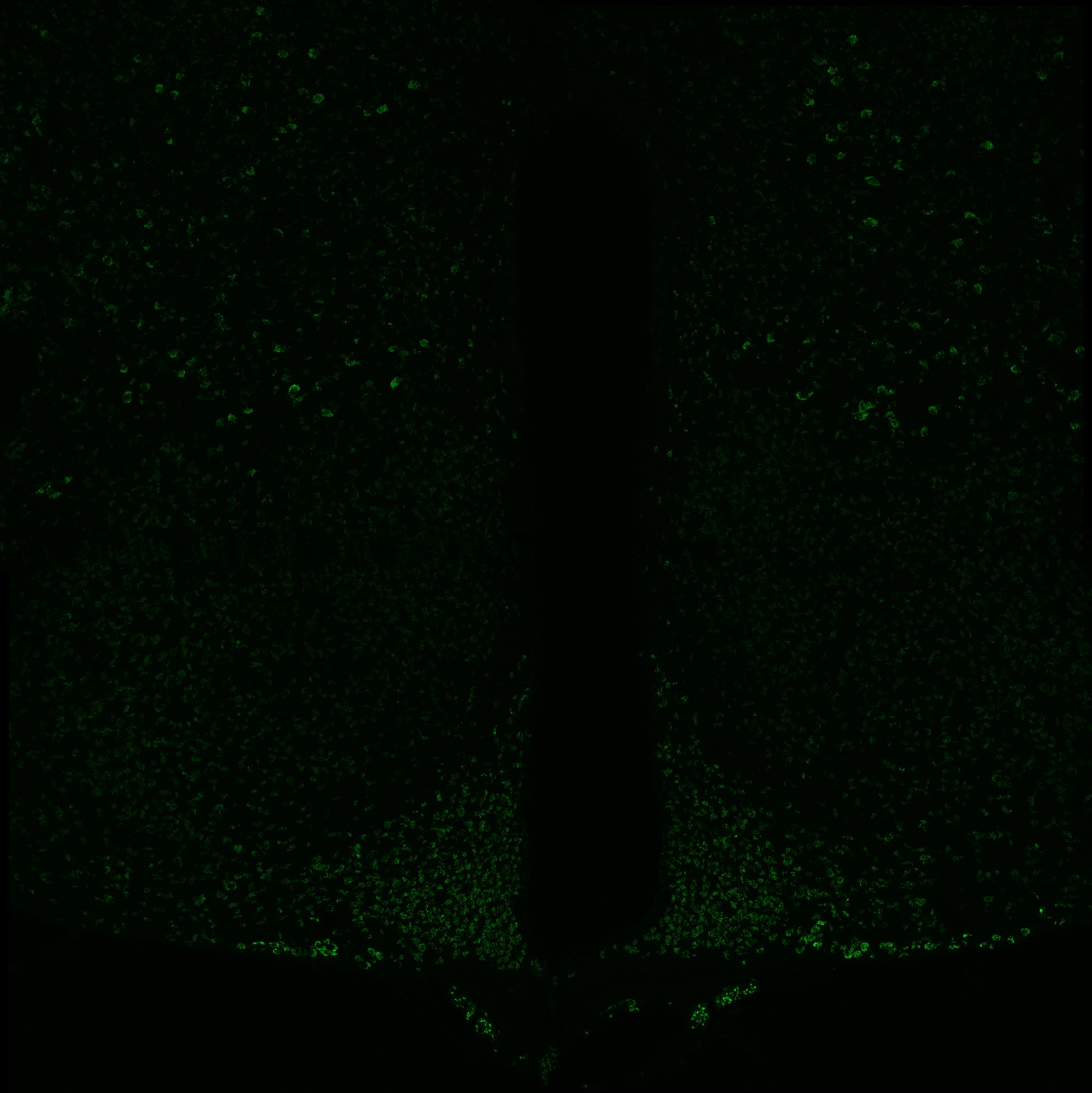

Supplement: Supplementary file 12 — Original data for Fig. 2a–d. [file 42255_2024_991_MOESM12_ESM.zip › Figure 2B/Mouse 4/1830-4 MidARH2.jpg]

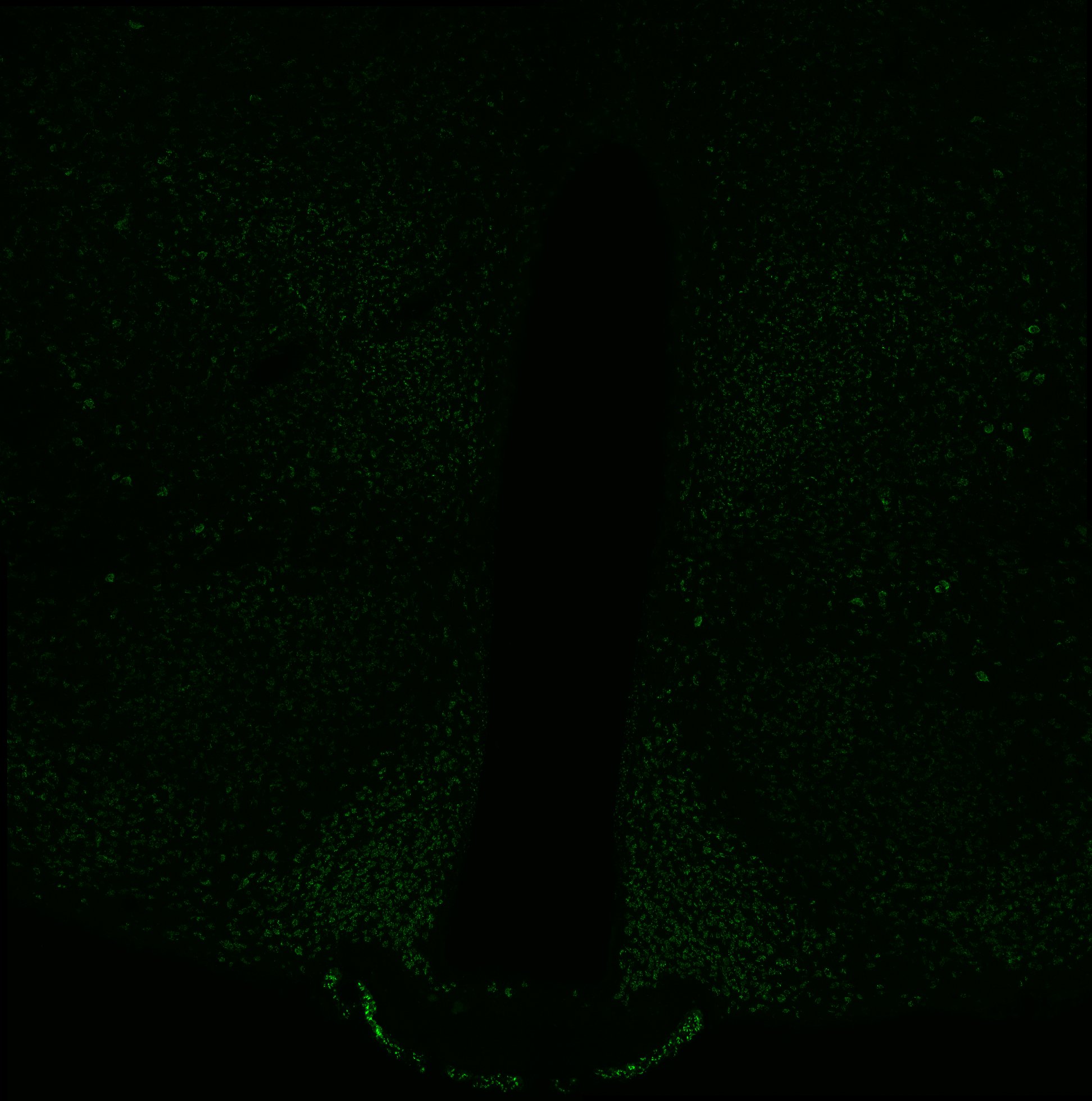

Supplement: Supplementary file 12 — Original data for Fig. 2a–d. [file 42255_2024_991_MOESM12_ESM.zip › Figure 2B/Mouse 4/1830-4 MidARH1.jpg]

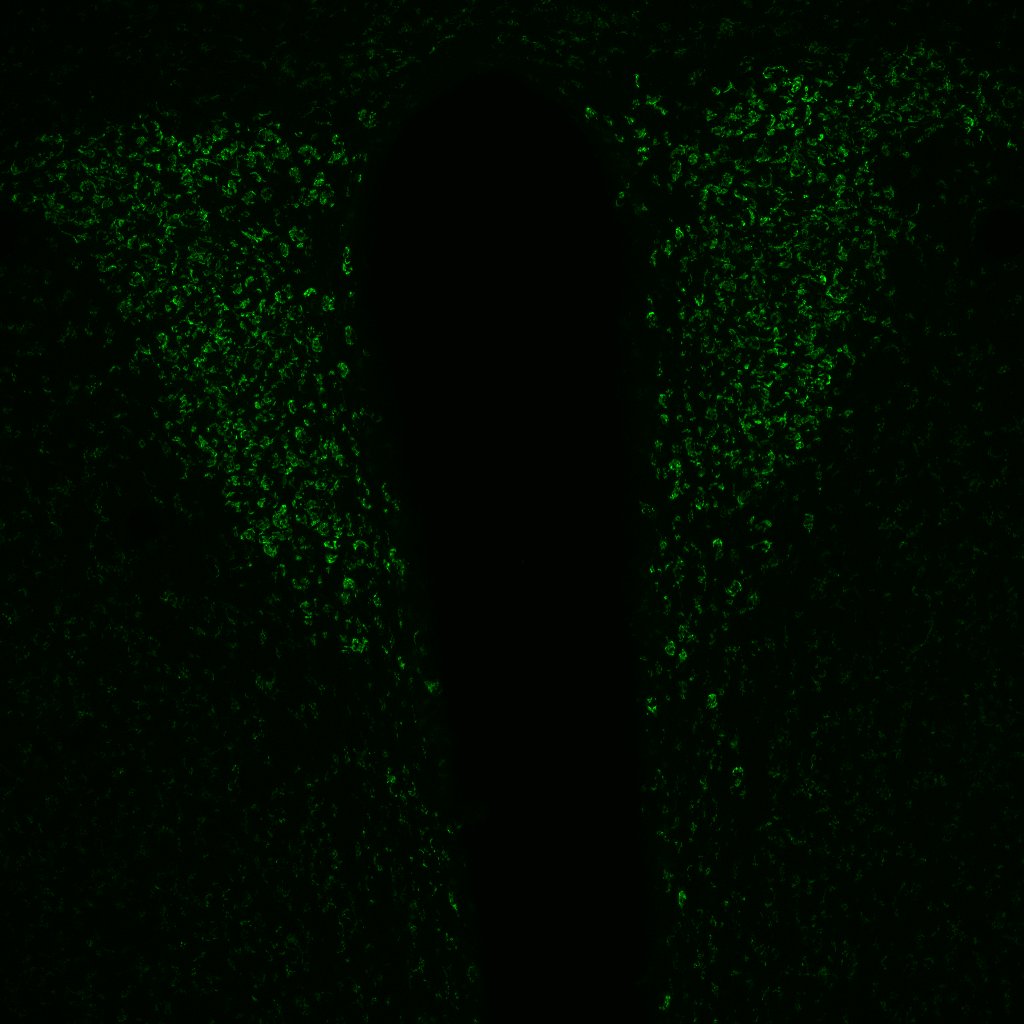

Supplement: Supplementary file 12 — Original data for Fig. 2a–d. [file 42255_2024_991_MOESM12_ESM.zip › Figure 2B/Mouse 4/1830-4 PVH.jpg]

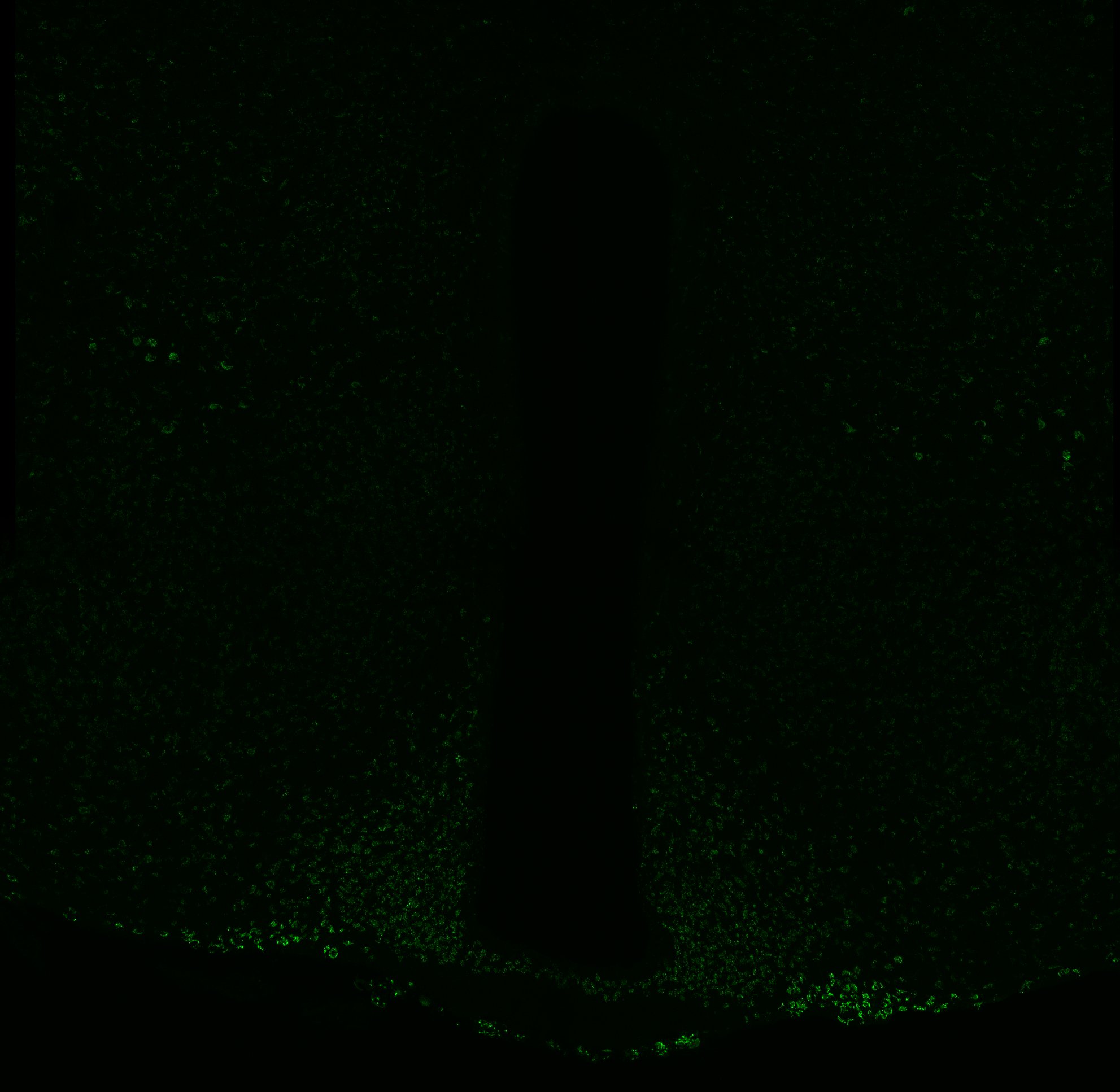

Supplement: Supplementary file 12 — Original data for Fig. 2a–d. [file 42255_2024_991_MOESM12_ESM.zip › Figure 2B/Mouse 5/1830-5 MidARH1.jpg]

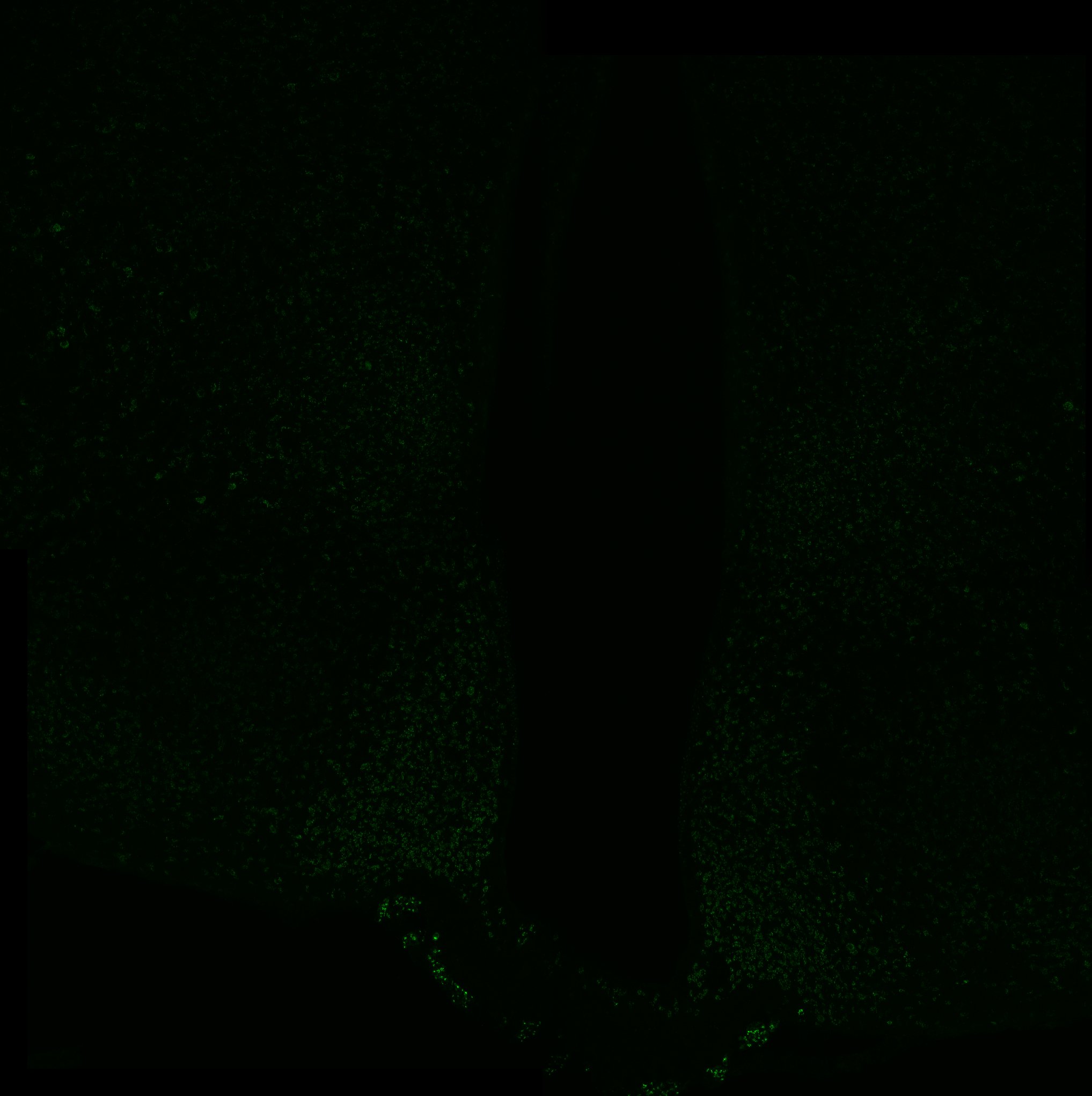

Supplement: Supplementary file 12 — Original data for Fig. 2a–d. [file 42255_2024_991_MOESM12_ESM.zip › Figure 2B/Mouse 5/1830-5 MidARH3.jpg]

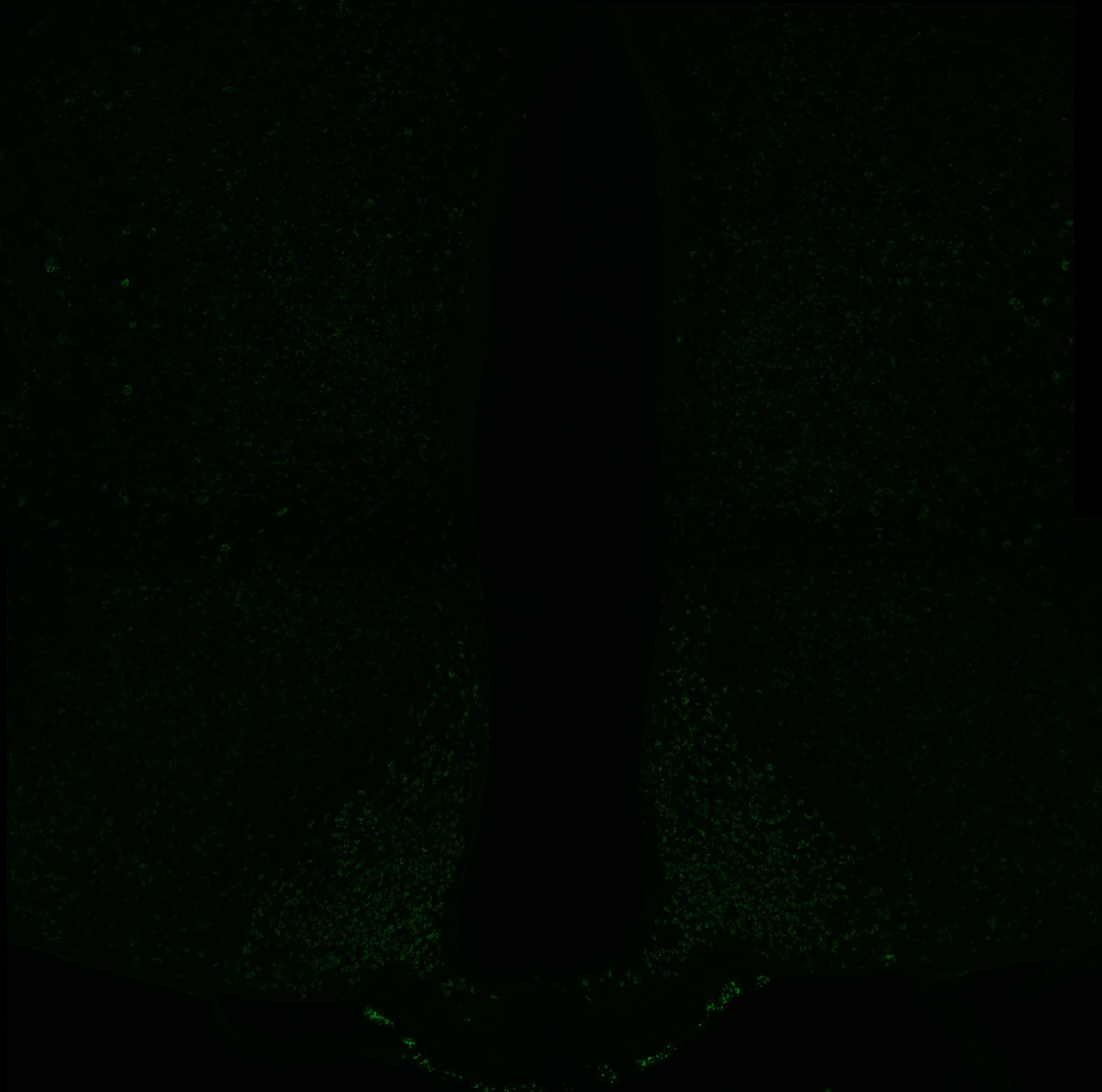

Supplement: Supplementary file 12 — Original data for Fig. 2a–d. [file 42255_2024_991_MOESM12_ESM.zip › Figure 2B/Mouse 5/1830-5 MidARH2.jpg]

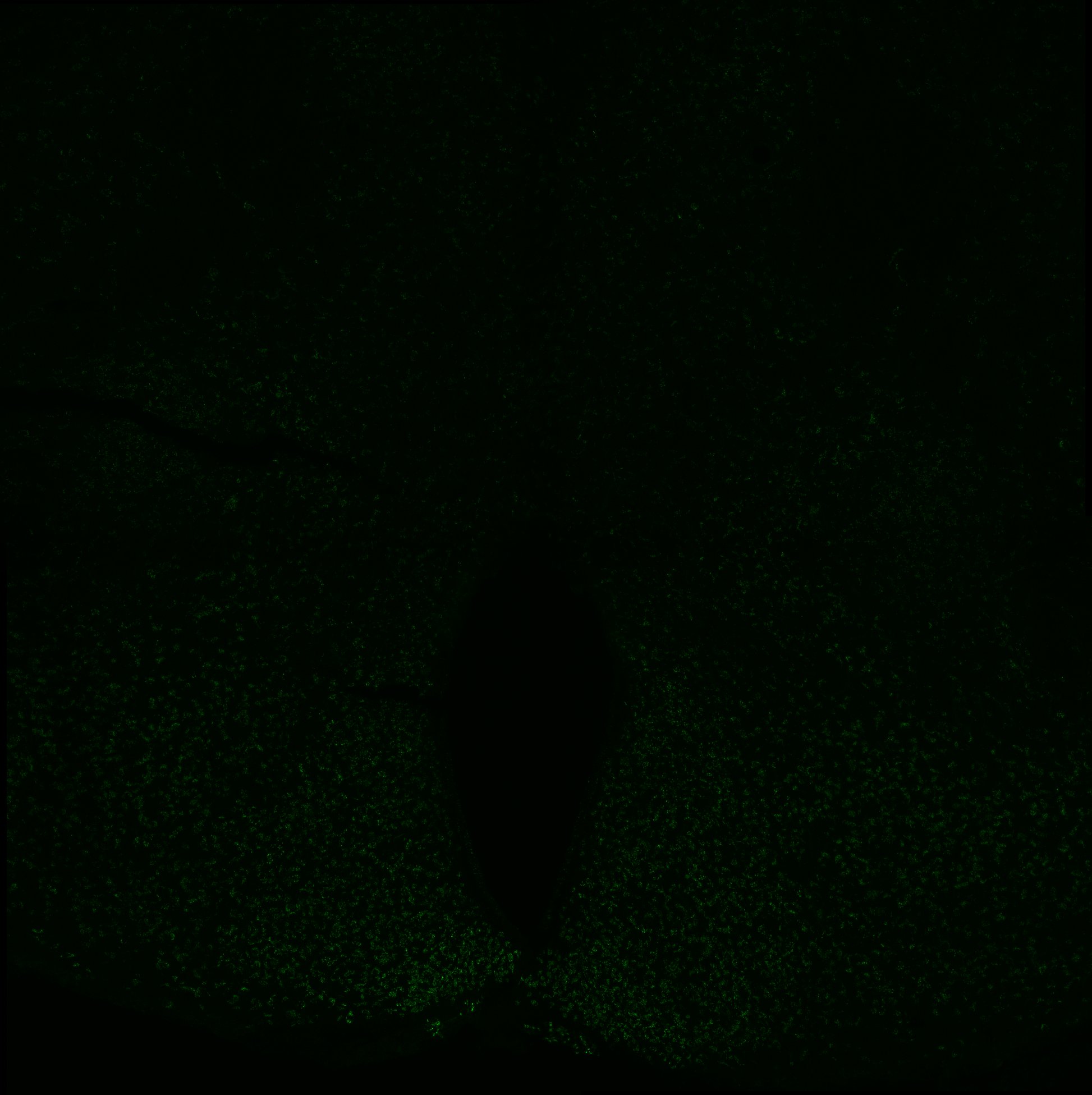

Supplement: Supplementary file 12 — Original data for Fig. 2a–d. [file 42255_2024_991_MOESM12_ESM.zip › Figure 2B/Mouse 5/1830-5 PostARH.jpg]

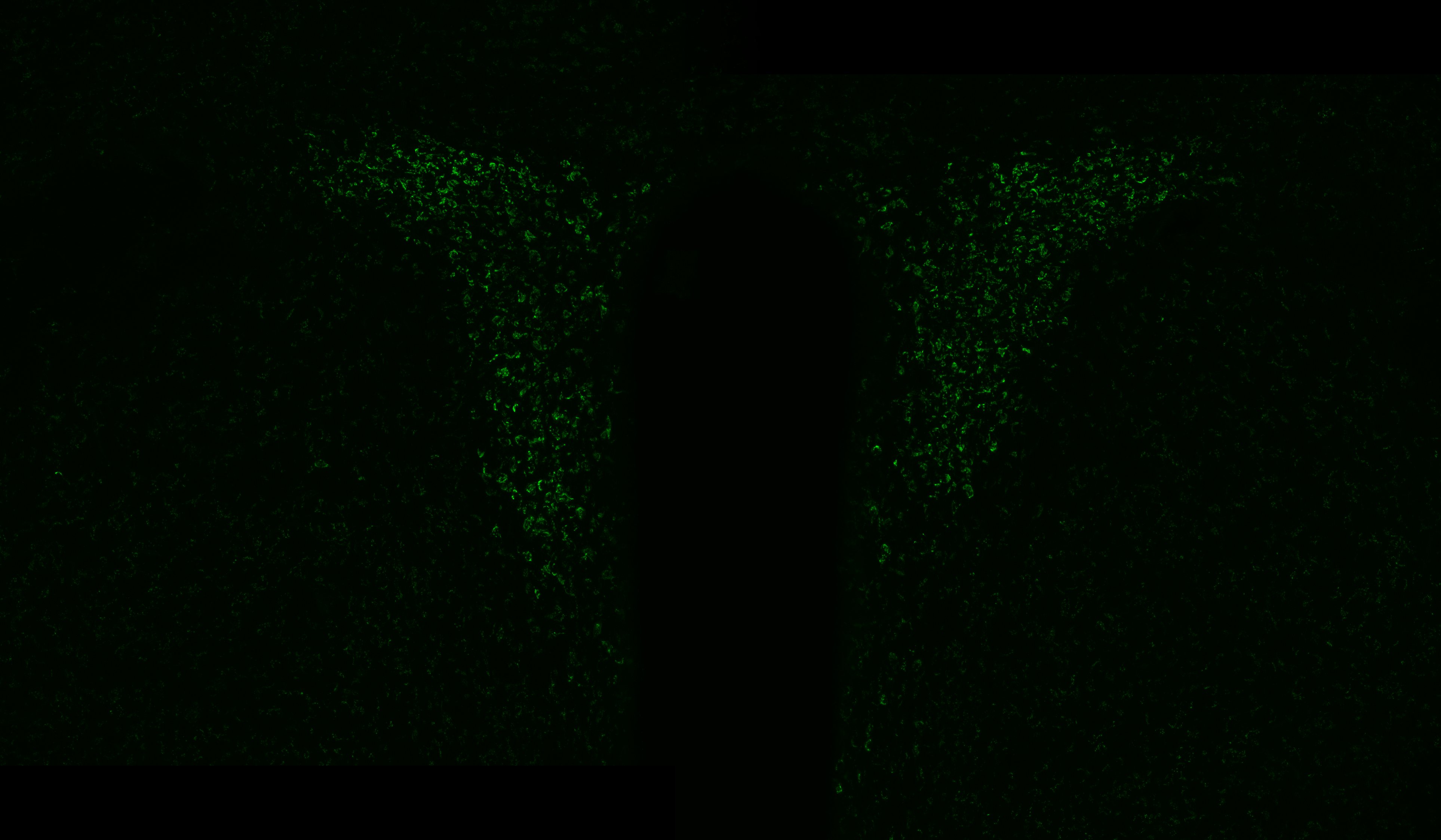

Supplement: Supplementary file 12 — Original data for Fig. 2a–d. [file 42255_2024_991_MOESM12_ESM.zip › Figure 2B/Mouse 5/1830-5 PVH.jpg]

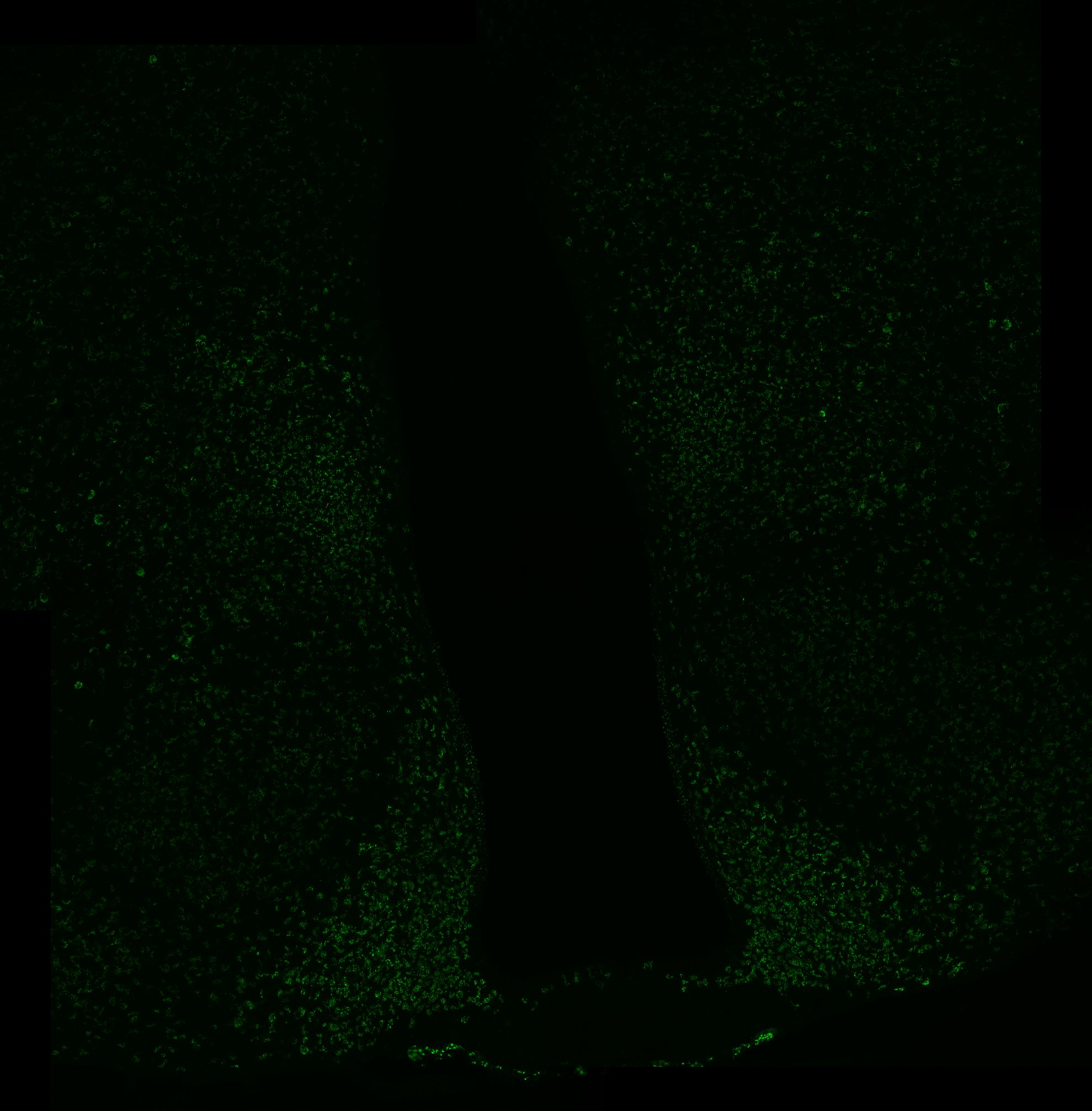

Supplement: Supplementary file 12 — Original data for Fig. 2a–d. [file 42255_2024_991_MOESM12_ESM.zip › Figure 2B/Mouse 2/1830-2 MidARH2.jpg]

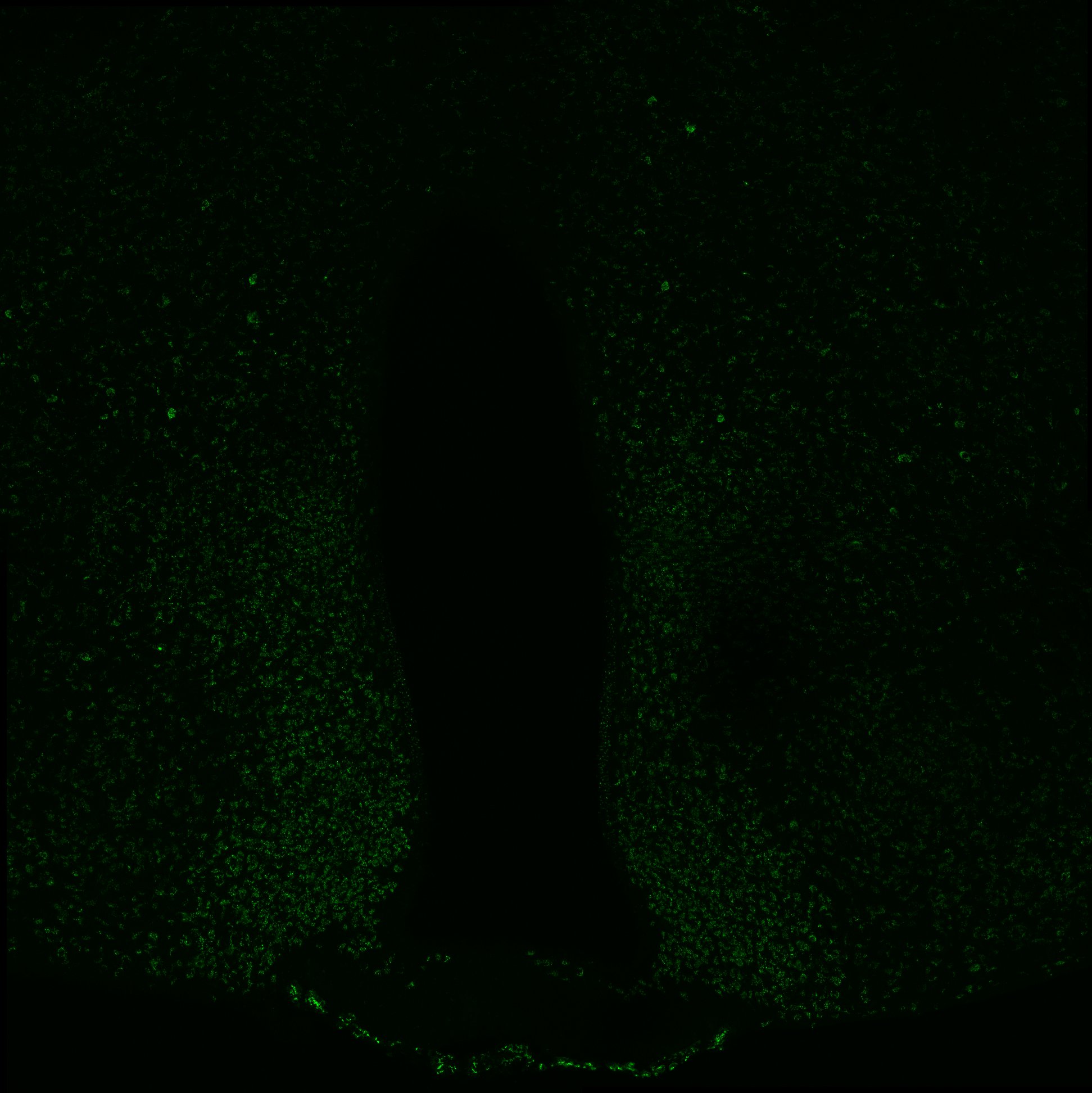

Supplement: Supplementary file 12 — Original data for Fig. 2a–d. [file 42255_2024_991_MOESM12_ESM.zip › Figure 2B/Mouse 2/1830-2 MidARH1.jpg]

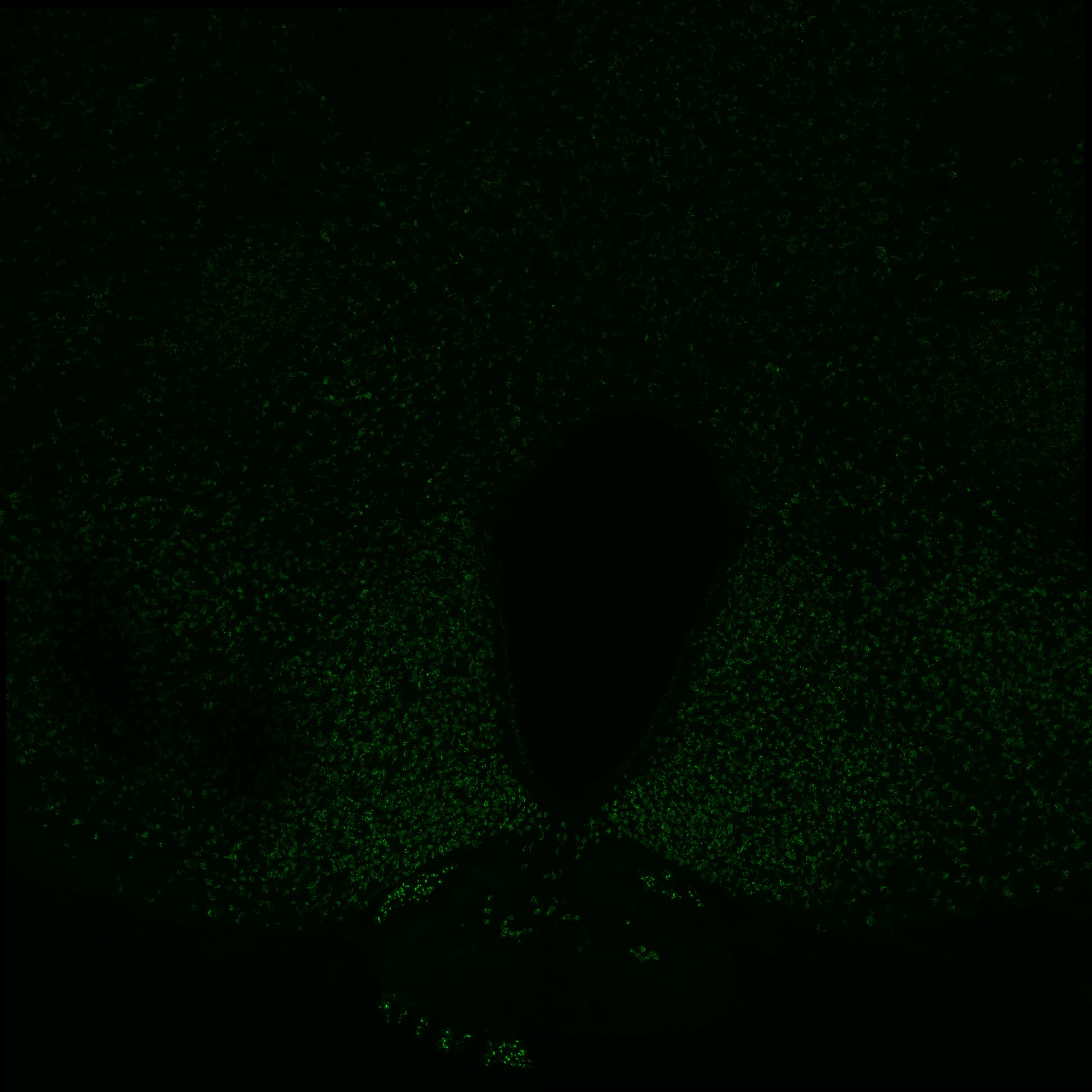

Supplement: Supplementary file 12 — Original data for Fig. 2a–d. [file 42255_2024_991_MOESM12_ESM.zip › Figure 2B/Mouse 2/1830-2 PostARH1.jpg]

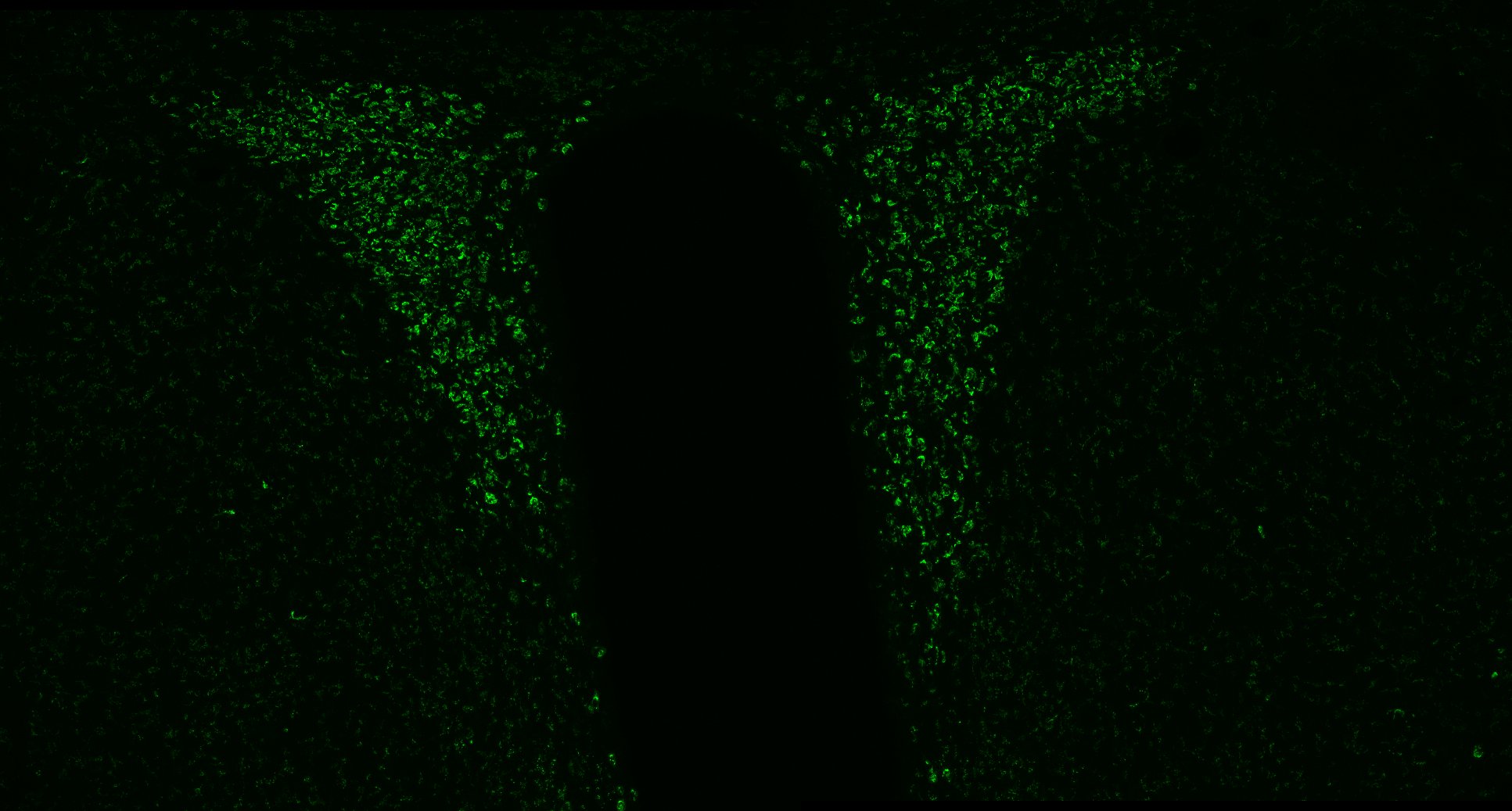

Supplement: Supplementary file 12 — Original data for Fig. 2a–d. [file 42255_2024_991_MOESM12_ESM.zip › Figure 2B/Mouse 2/1830-2 PVH1.jpg]

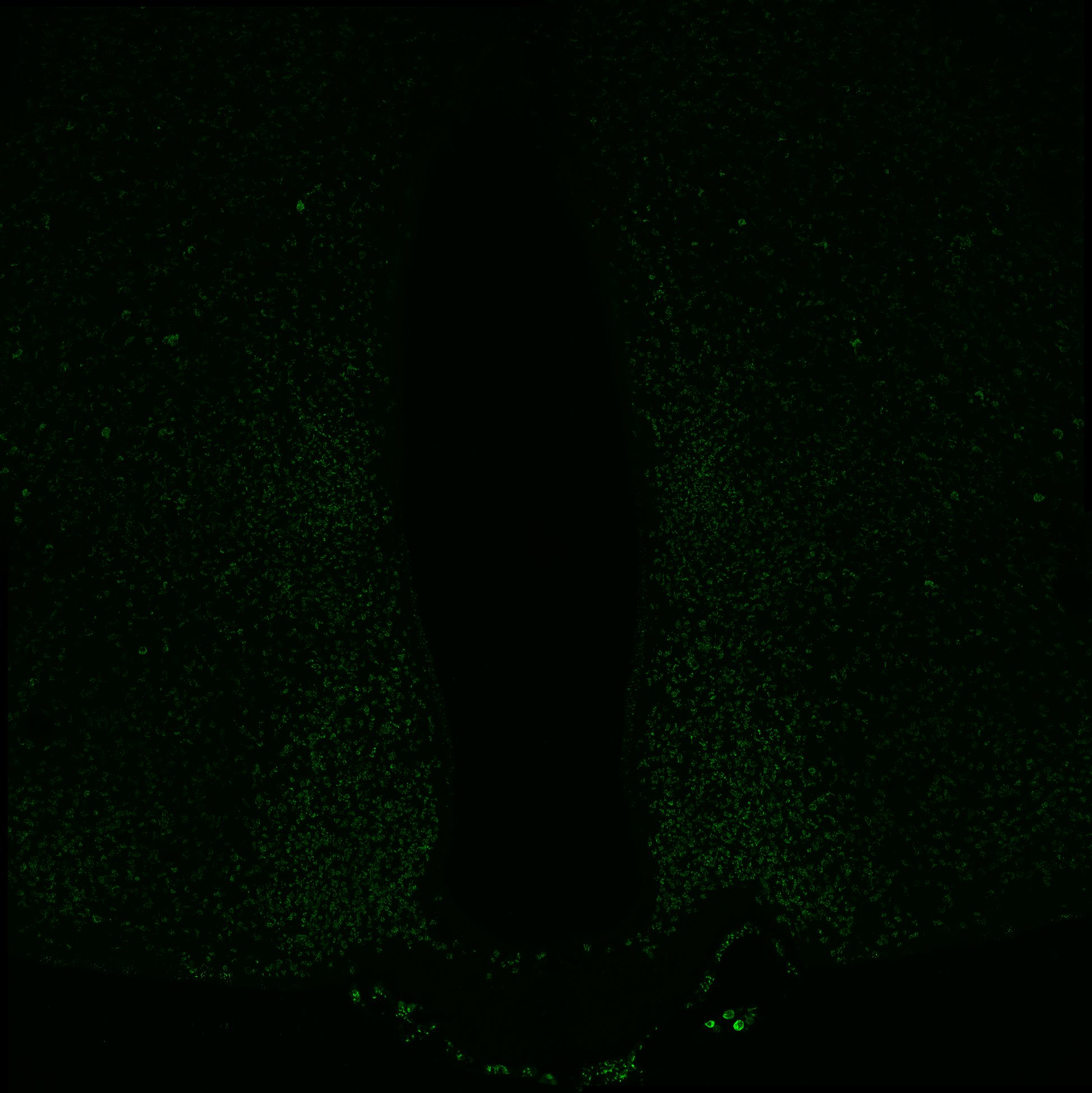

Supplement: Supplementary file 12 — Original data for Fig. 2a–d. [file 42255_2024_991_MOESM12_ESM.zip › Figure 2B/Mouse 26/1821-1 MidARH2.jpg]

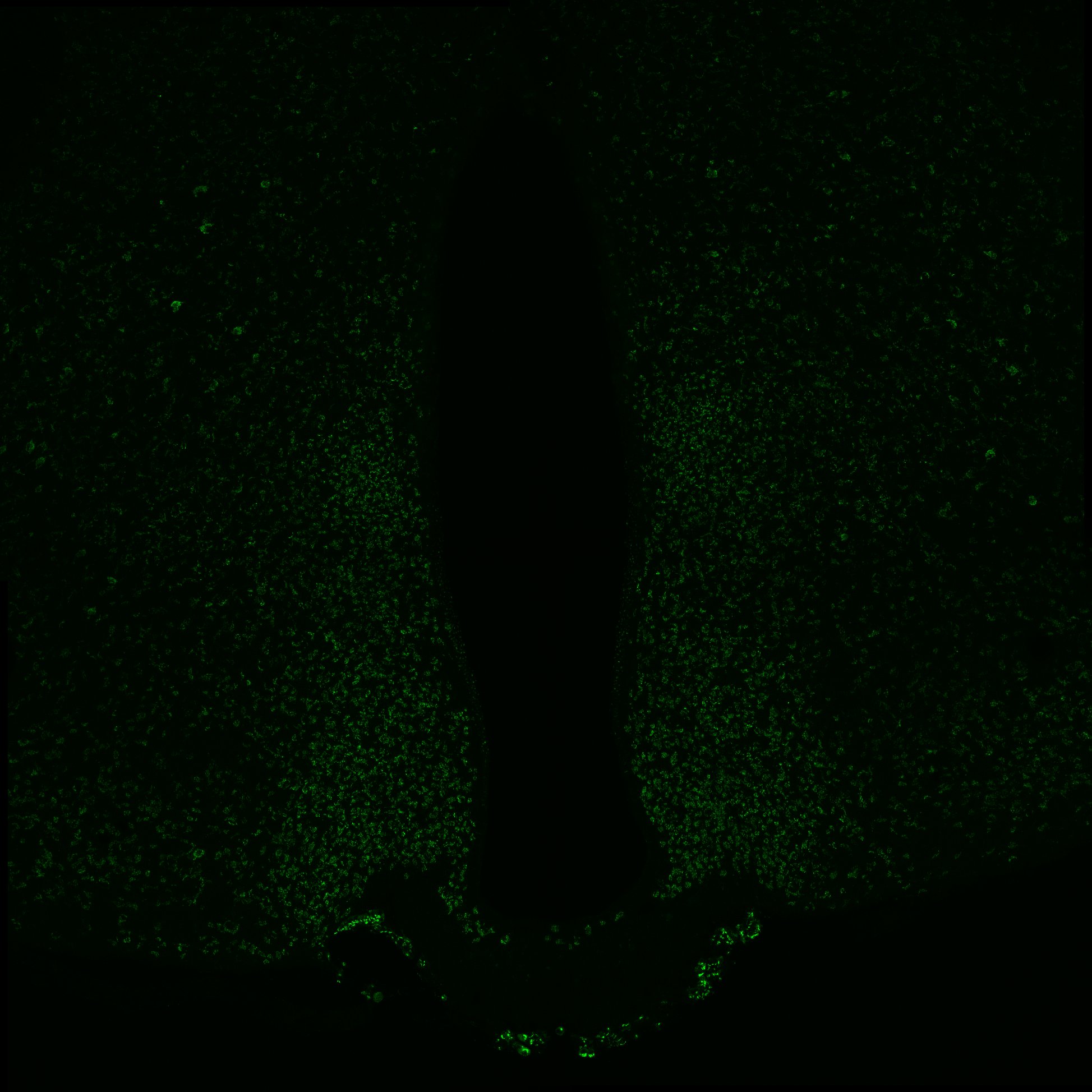

Supplement: Supplementary file 12 — Original data for Fig. 2a–d. [file 42255_2024_991_MOESM12_ESM.zip › Figure 2B/Mouse 26/1821-1 MidARH1.jpg]

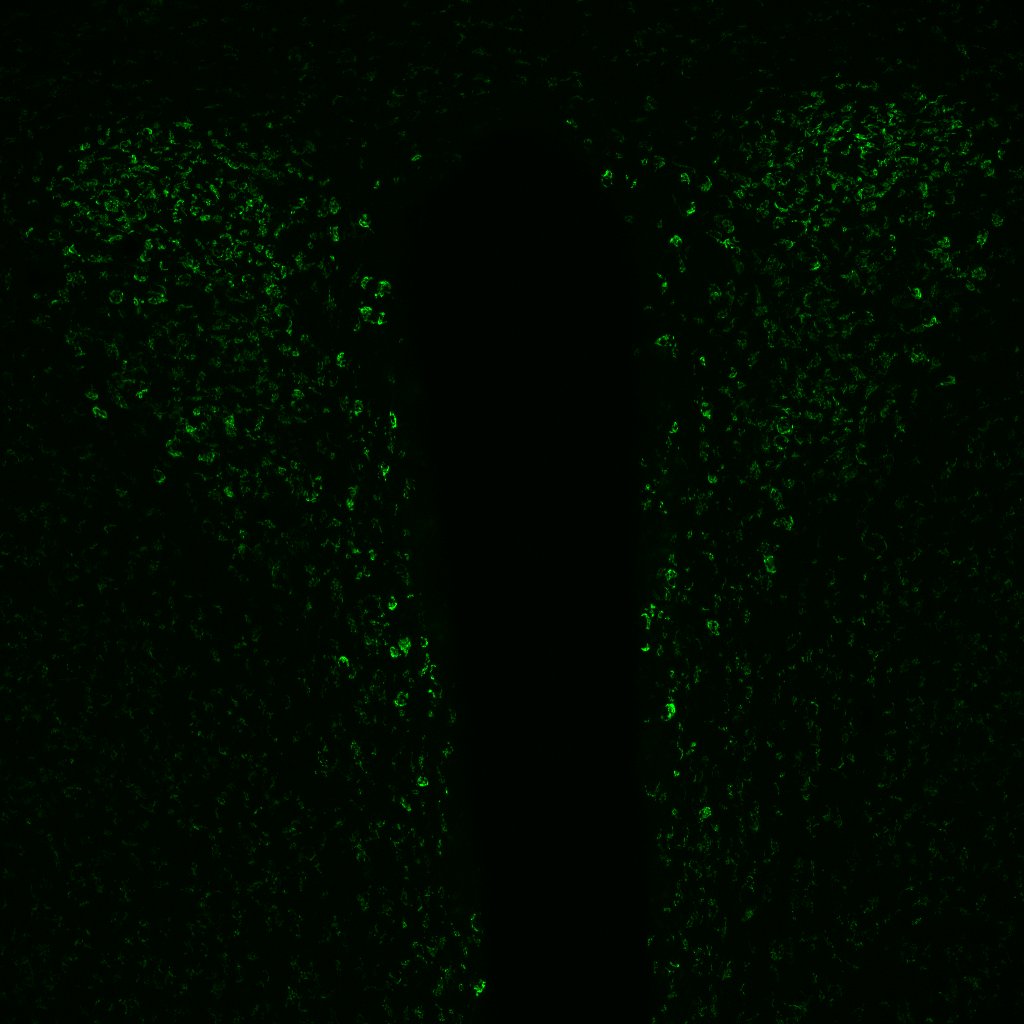

Supplement: Supplementary file 12 — Original data for Fig. 2a–d. [file 42255_2024_991_MOESM12_ESM.zip › Figure 2B/Mouse 26/1821-1 PVH1.jpg]

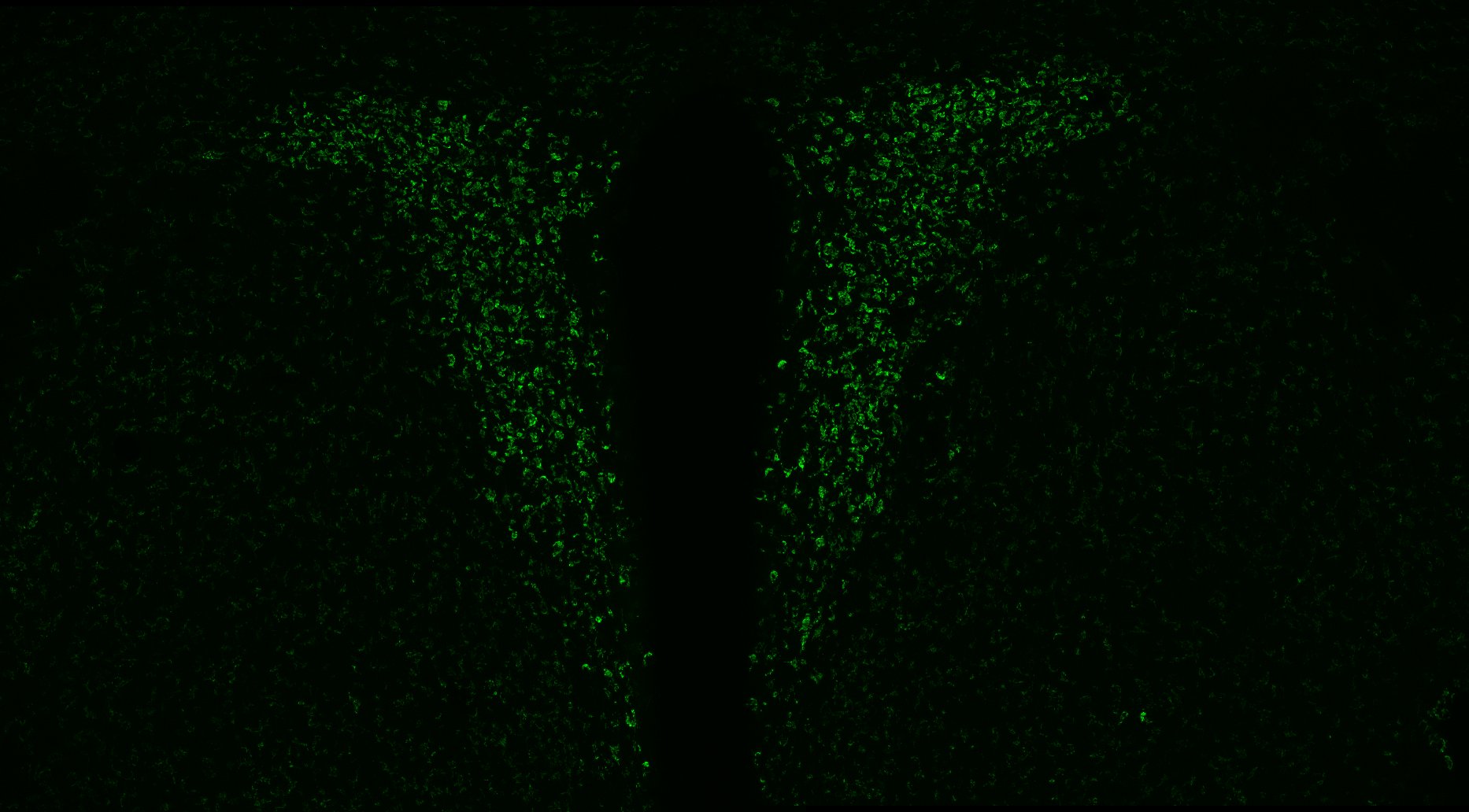

Supplement: Supplementary file 12 — Original data for Fig. 2a–d. [file 42255_2024_991_MOESM12_ESM.zip › Figure 2B/Mouse 26/1821-1 PVH2.jpg]

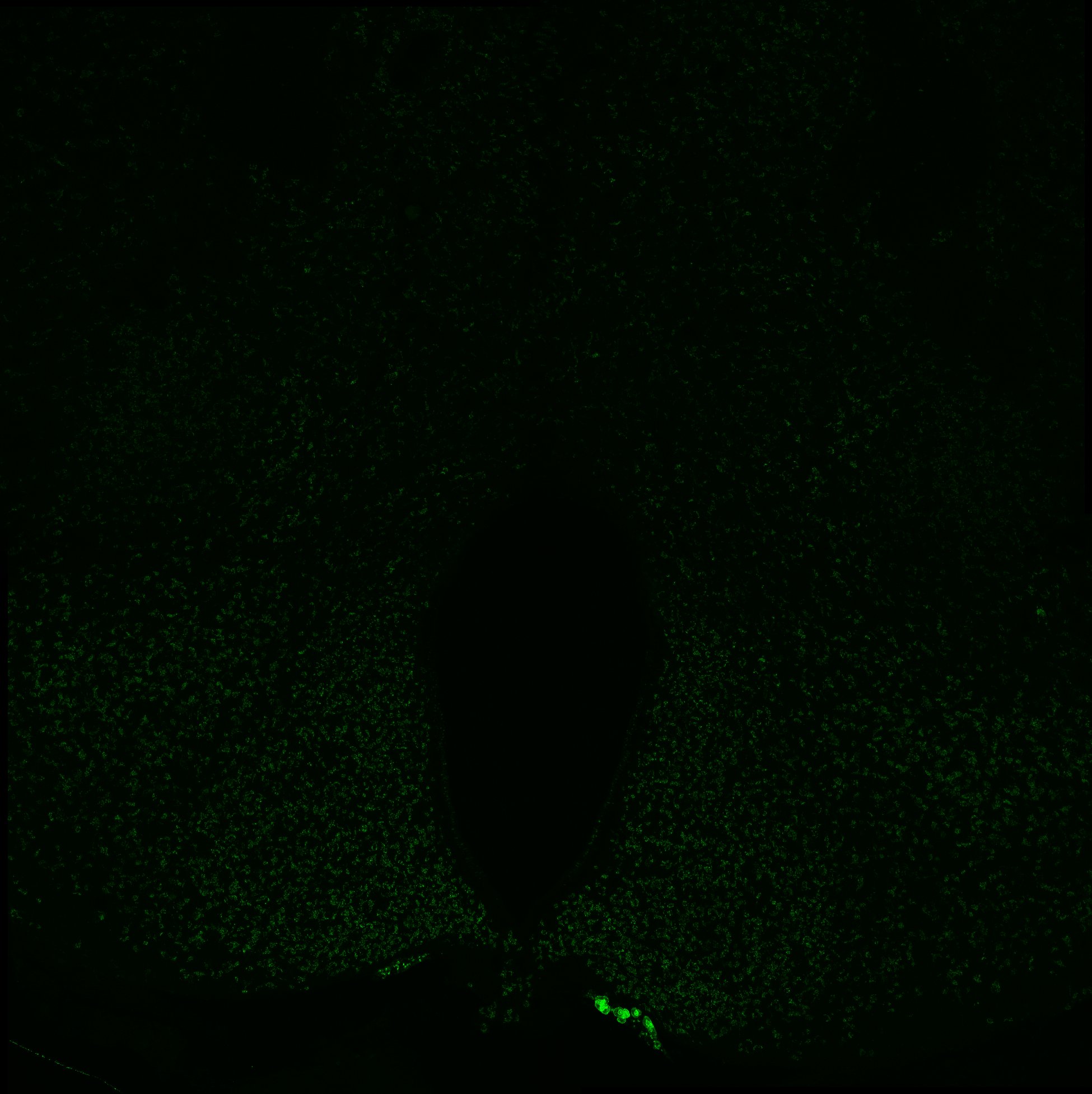

Supplement: Supplementary file 12 — Original data for Fig. 2a–d. [file 42255_2024_991_MOESM12_ESM.zip › Figure 2B/Mouse 26/1821-1 PostARH.jpg]

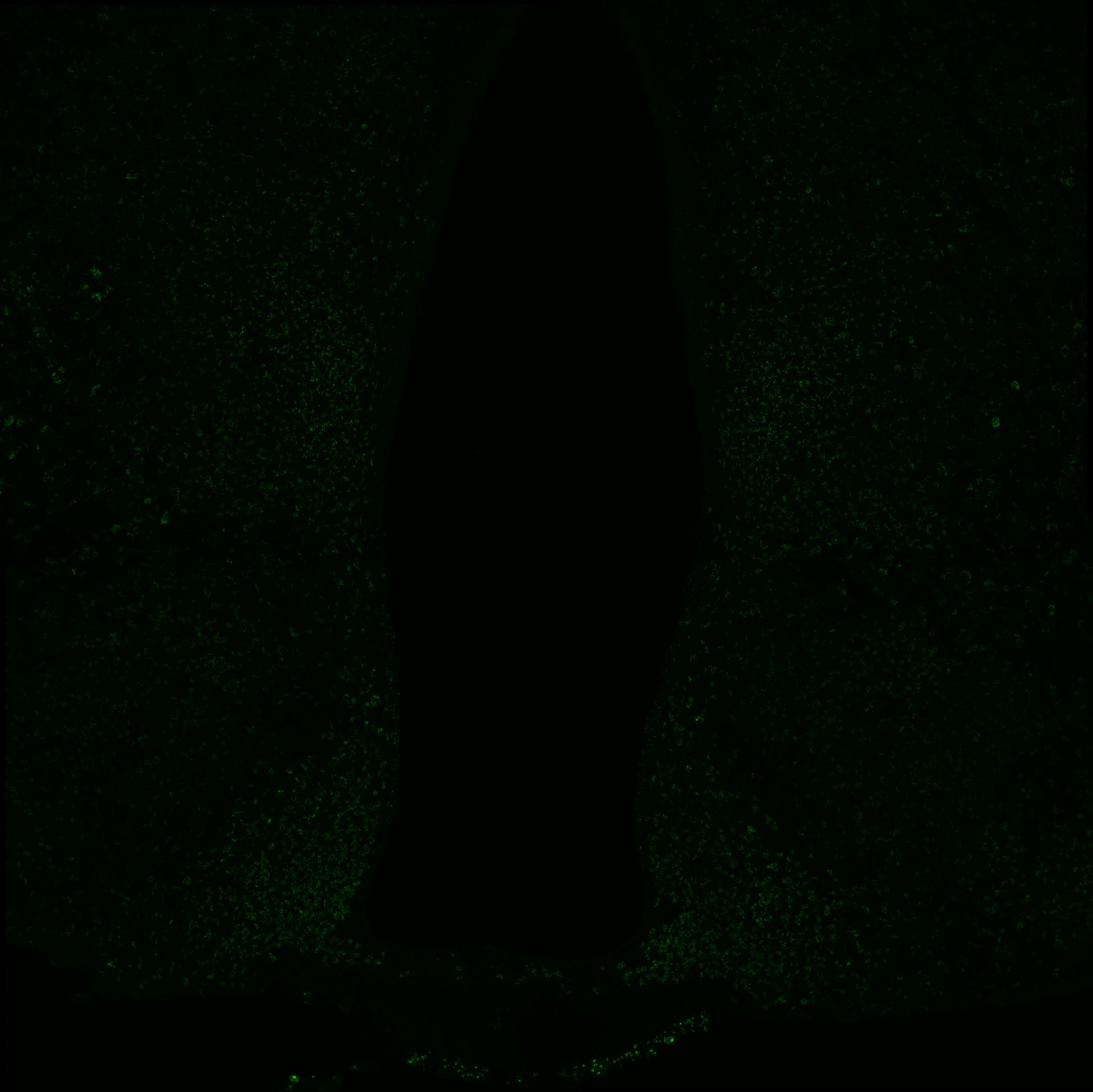

Supplement: Supplementary file 12 — Original data for Fig. 2a–d. [file 42255_2024_991_MOESM12_ESM.zip › Figure 2B/Mouse 19/1818-4 MidARH1.jpg]

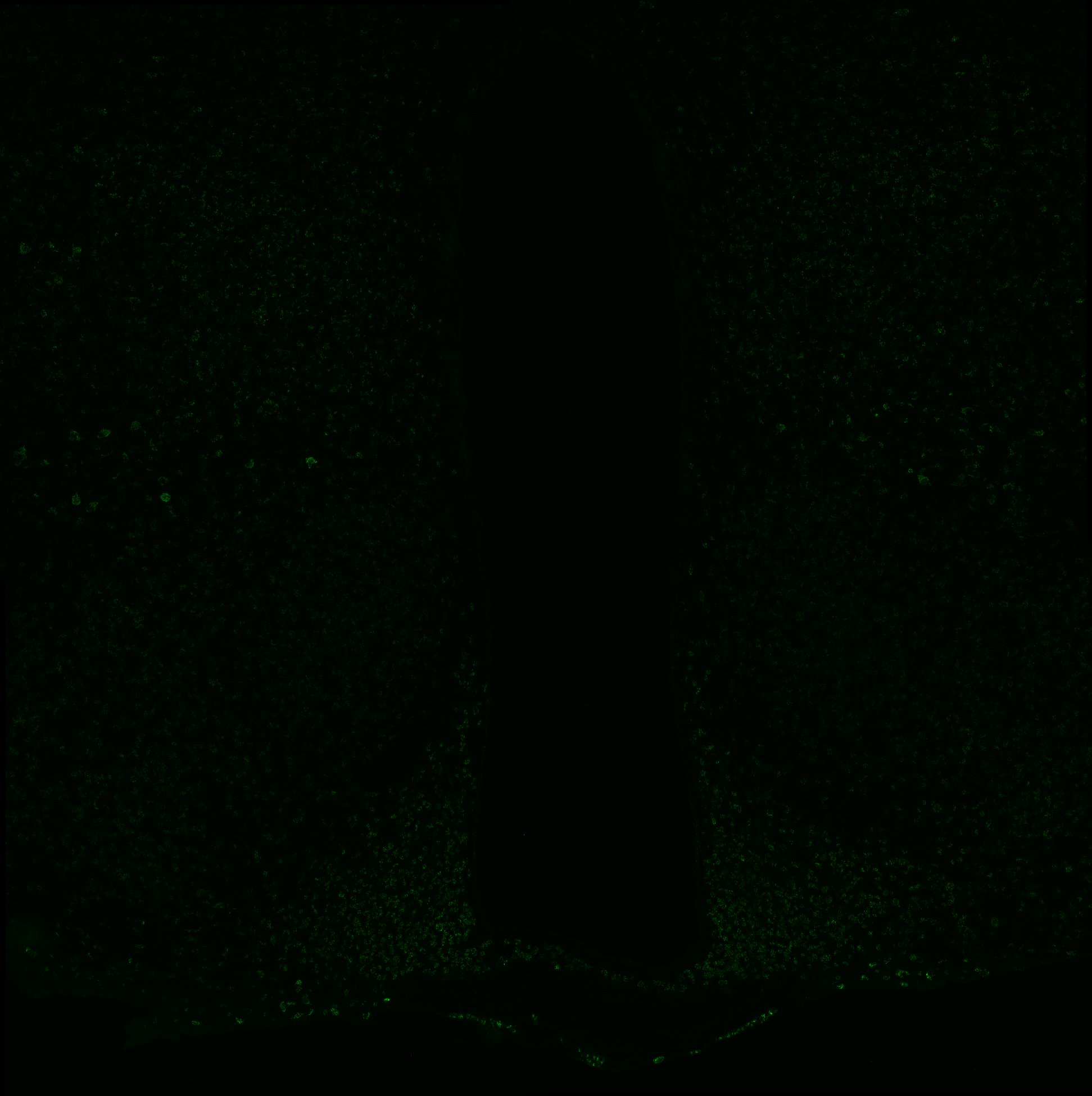

Supplement: Supplementary file 12 — Original data for Fig. 2a–d. [file 42255_2024_991_MOESM12_ESM.zip › Figure 2B/Mouse 19/1818-4 MidARH2.jpg]

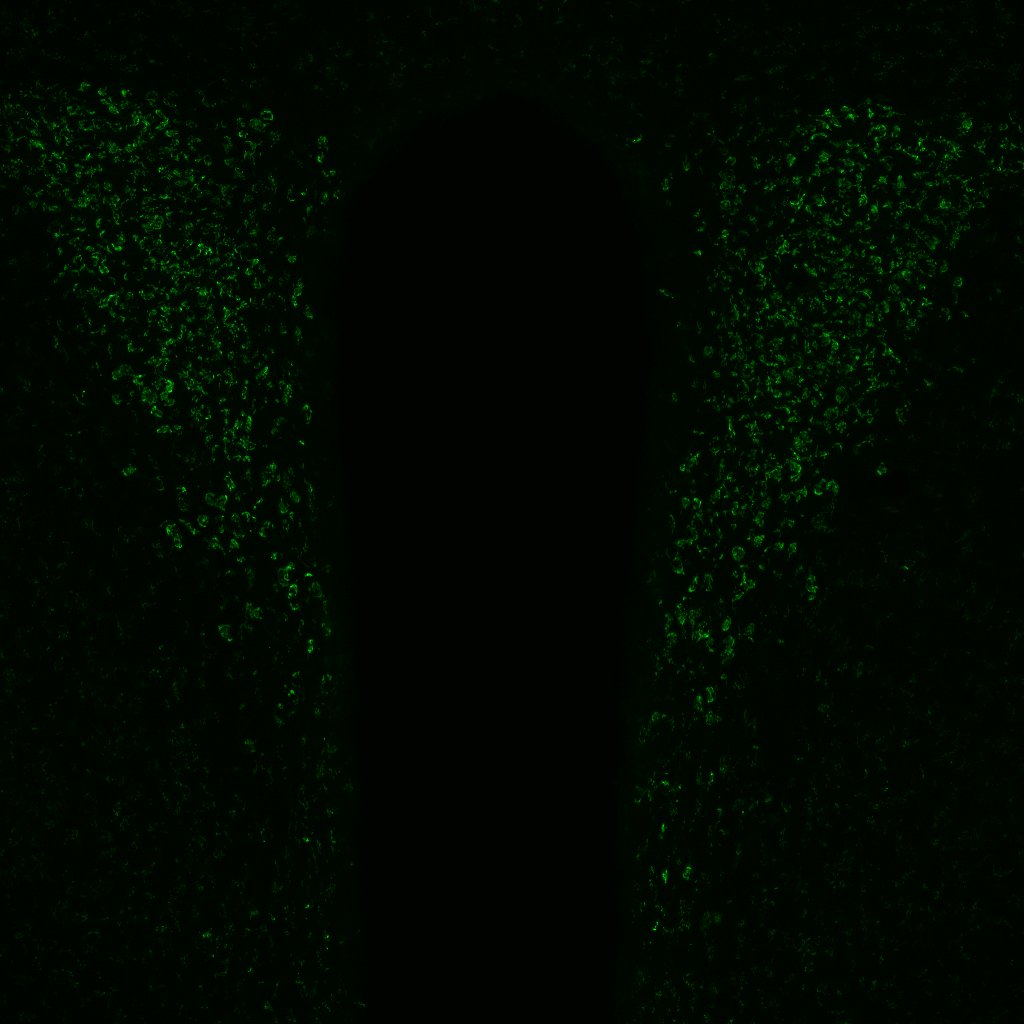

Supplement: Supplementary file 12 — Original data for Fig. 2a–d. [file 42255_2024_991_MOESM12_ESM.zip › Figure 2B/Mouse 19/1818-4 PVH.jpg]

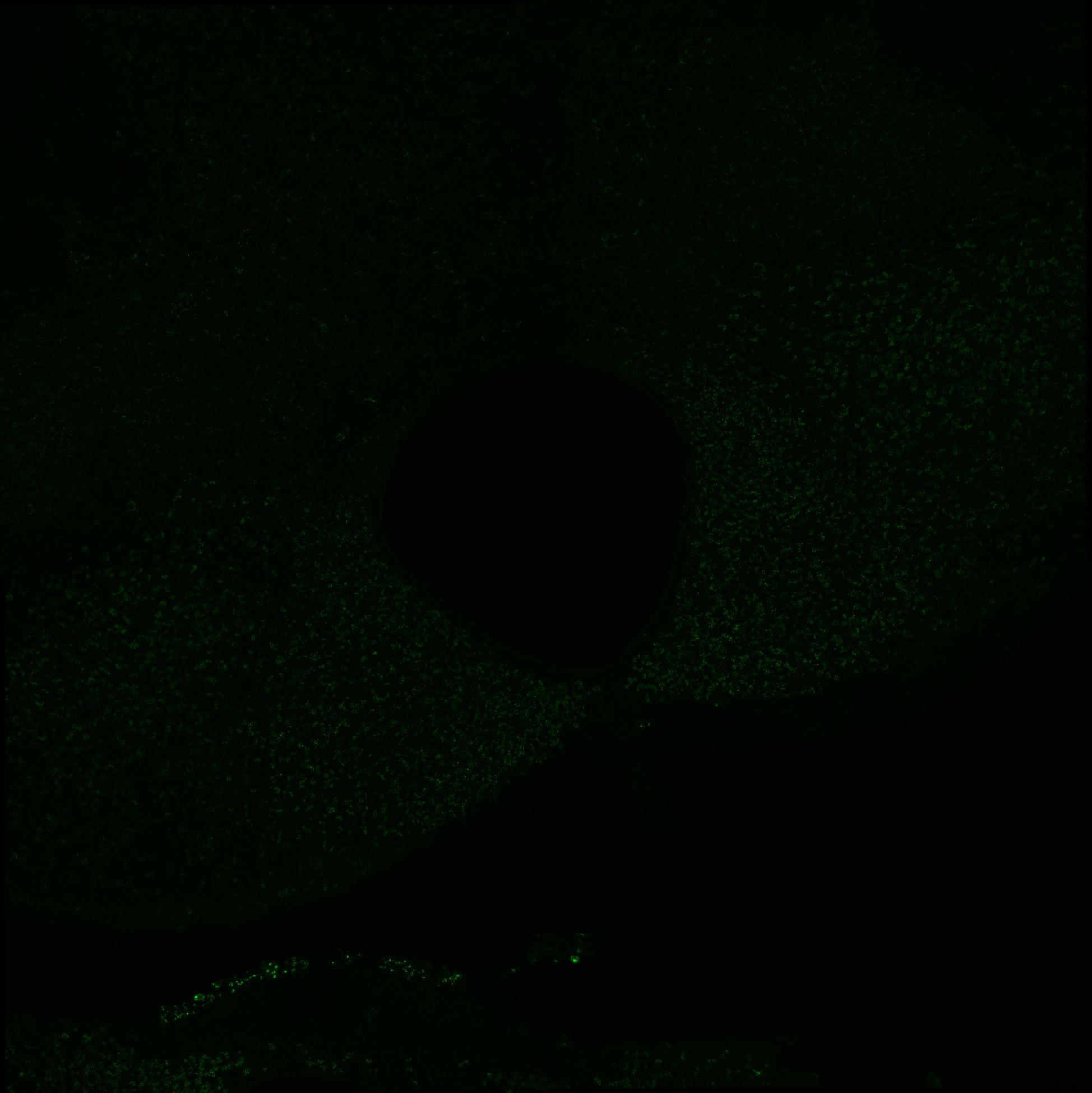

Supplement: Supplementary file 12 — Original data for Fig. 2a–d. [file 42255_2024_991_MOESM12_ESM.zip › Figure 2B/Mouse 19/1818-4 PostARH.jpg]

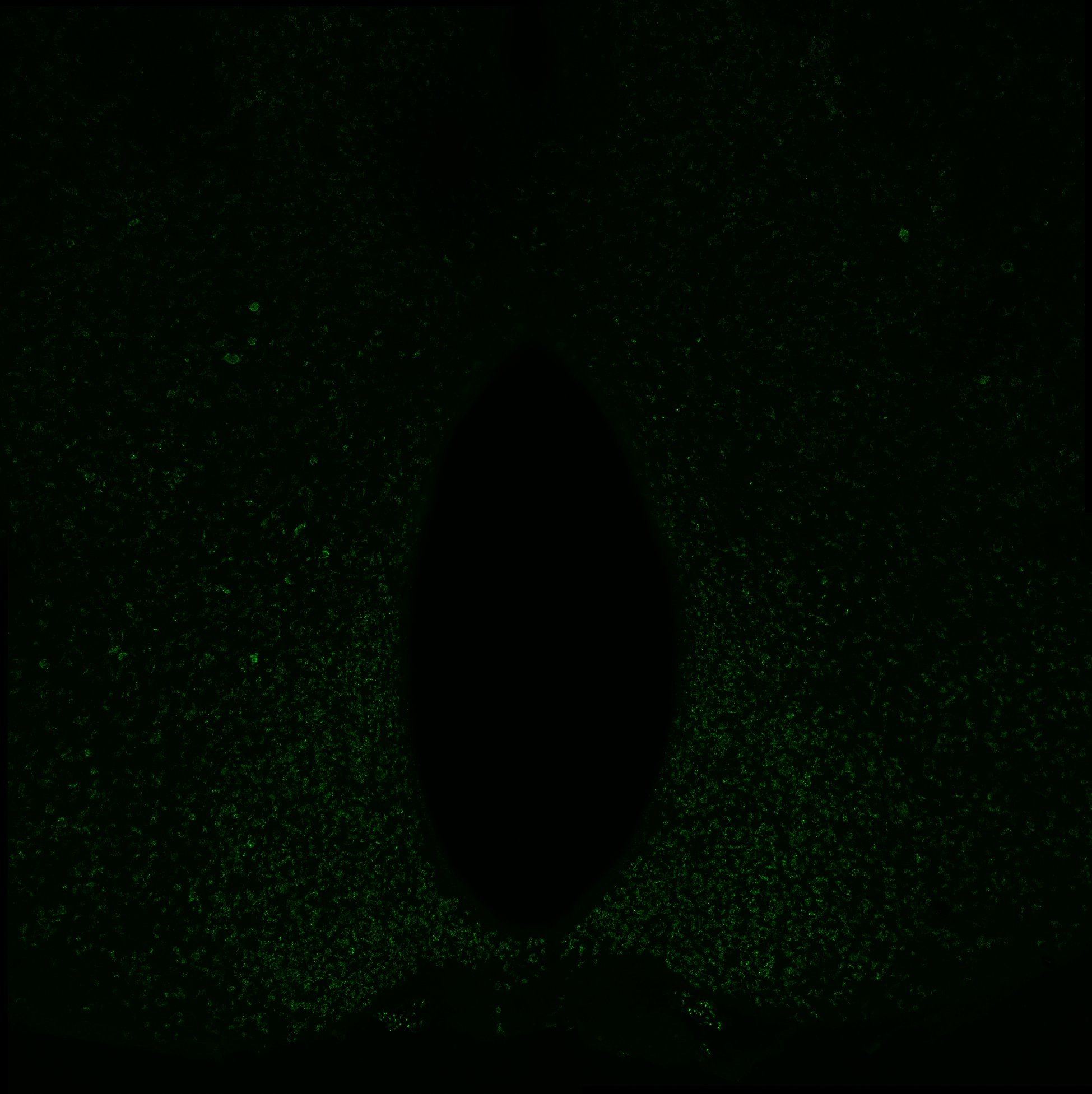

Supplement: Supplementary file 12 — Original data for Fig. 2a–d. [file 42255_2024_991_MOESM12_ESM.zip › Figure 2B/Mouse 21/1814-1 PostARH.jpg]

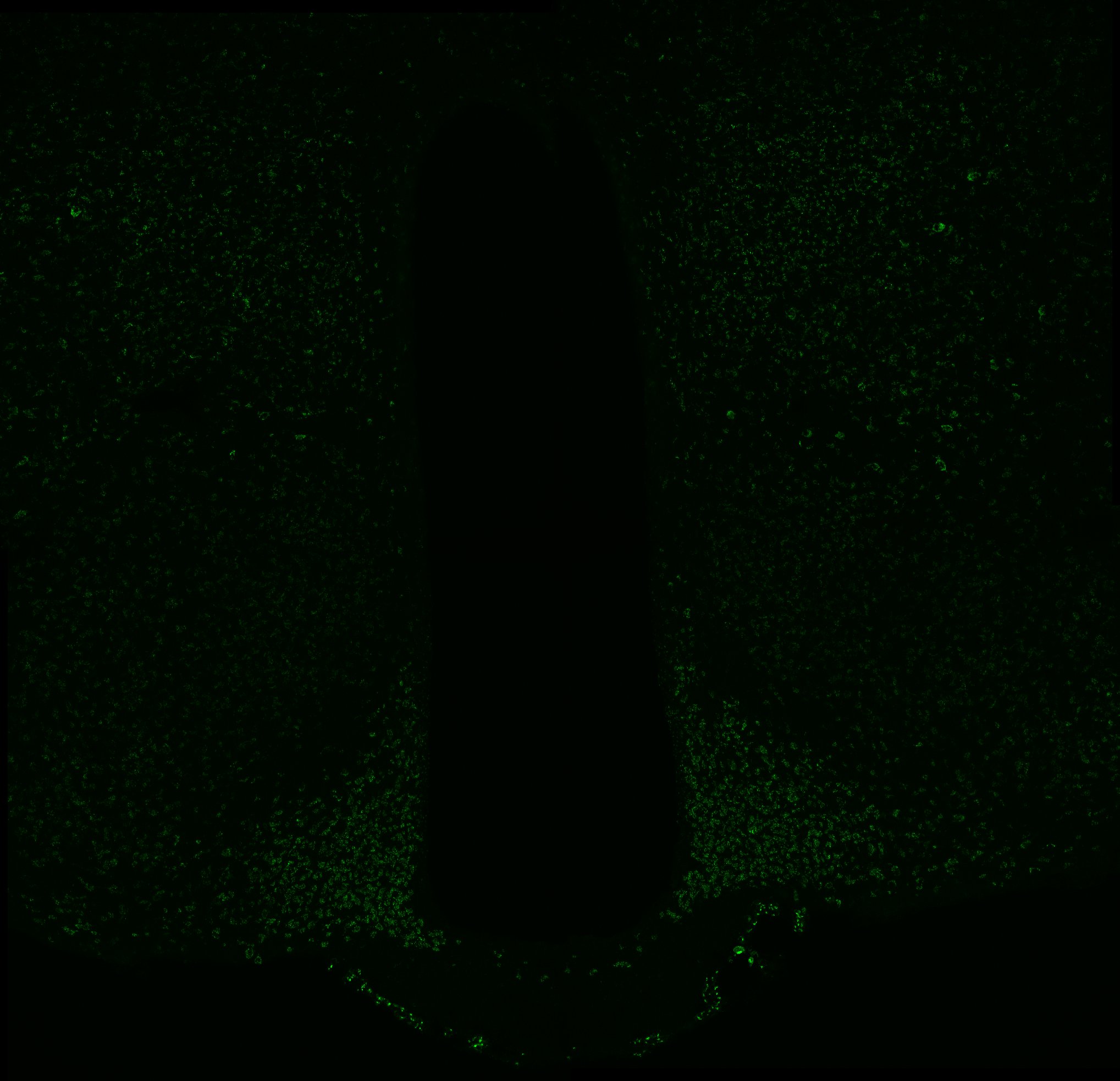

Supplement: Supplementary file 12 — Original data for Fig. 2a–d. [file 42255_2024_991_MOESM12_ESM.zip › Figure 2B/Mouse 21/1814-1 MidARH2.jpg]

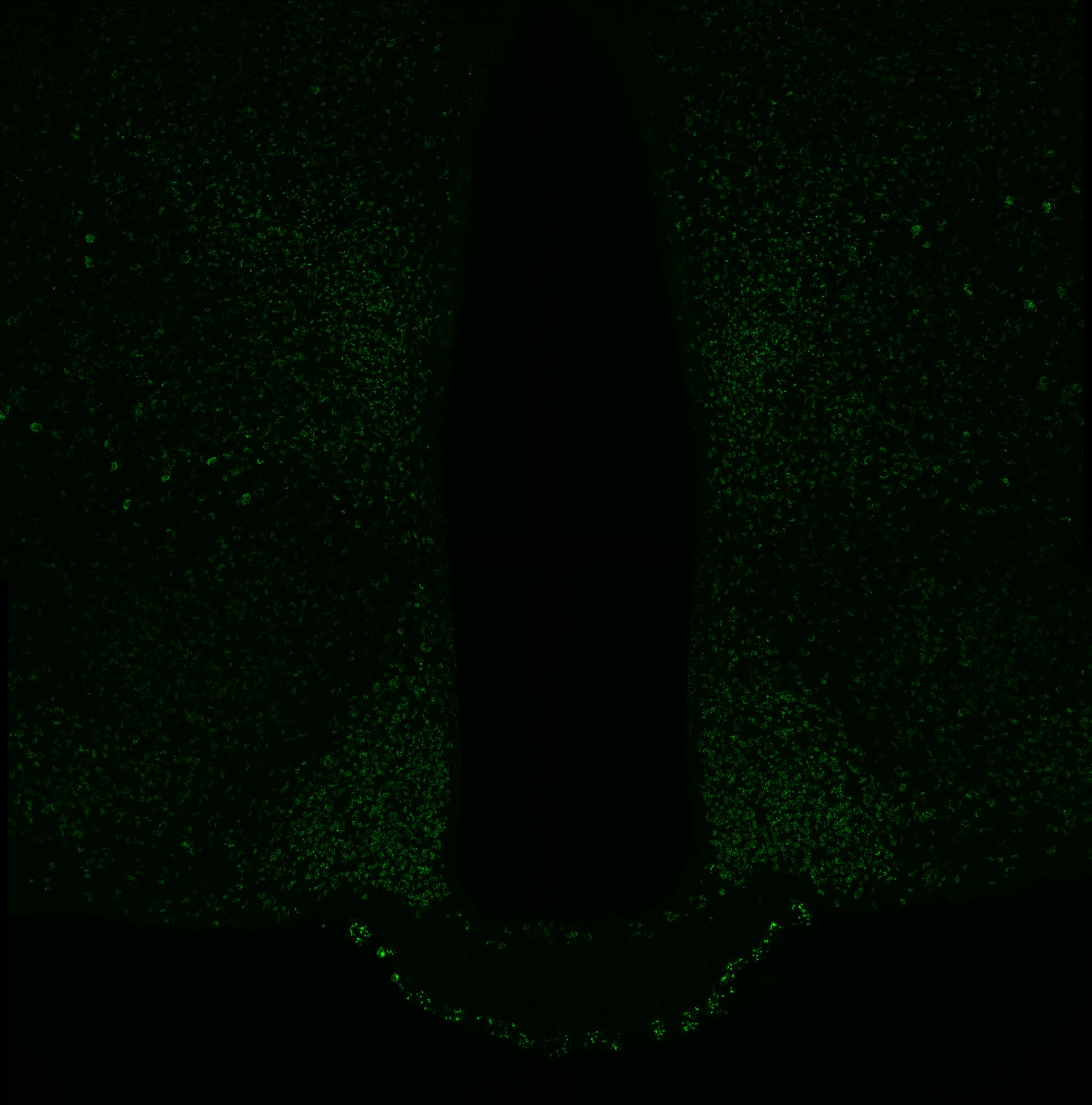

Supplement: Supplementary file 12 — Original data for Fig. 2a–d. [file 42255_2024_991_MOESM12_ESM.zip › Figure 2B/Mouse 21/1814-1 MidARH1.jpg]

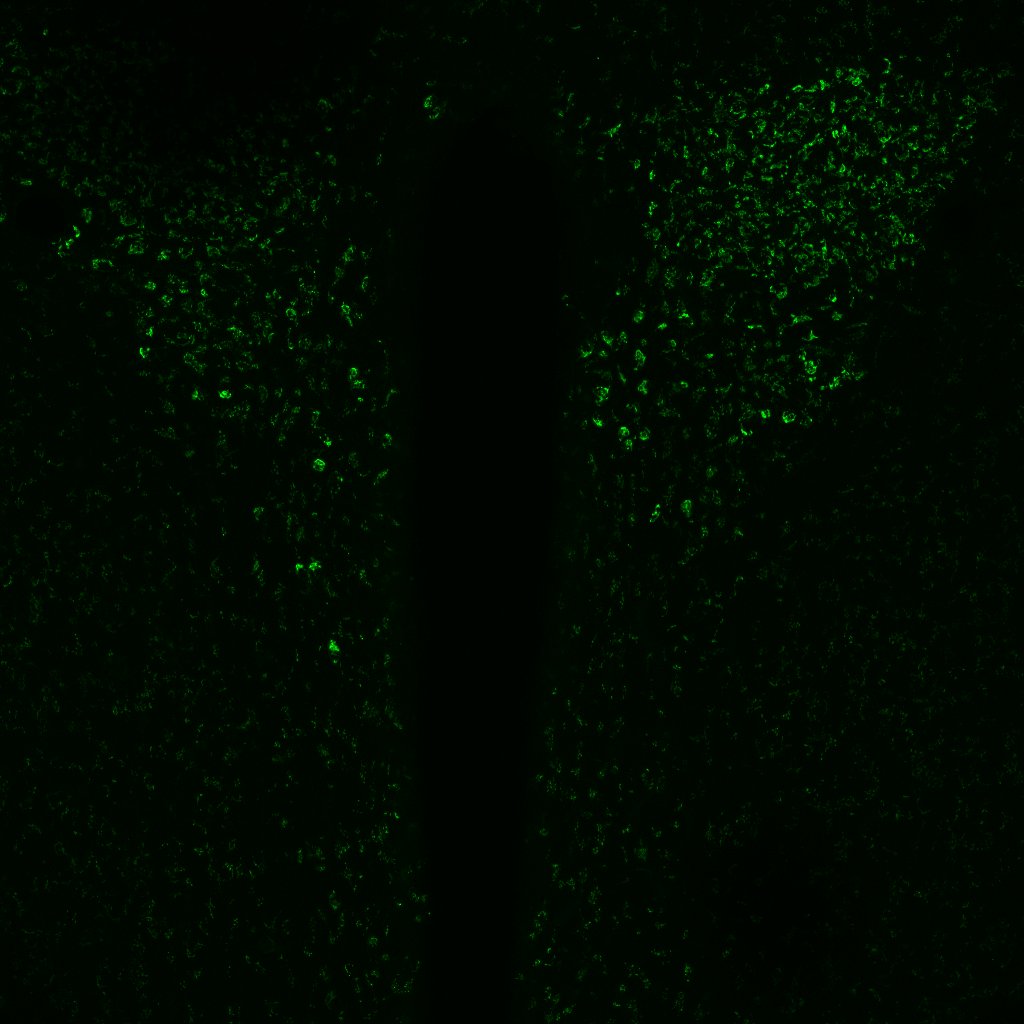

Supplement: Supplementary file 12 — Original data for Fig. 2a–d. [file 42255_2024_991_MOESM12_ESM.zip › Figure 2B/Mouse 17/1818-2 PVH1.jpg]

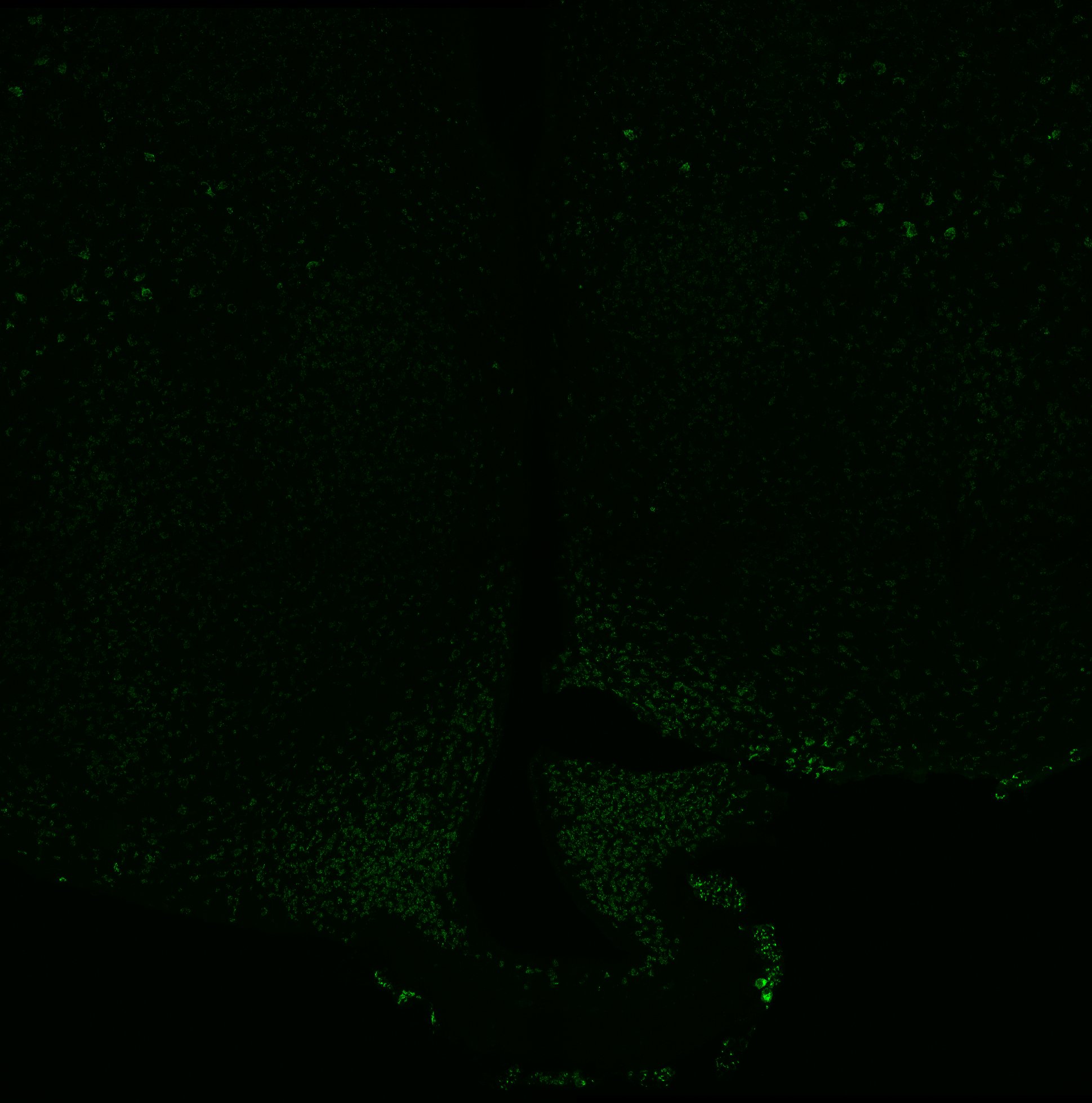

Supplement: Supplementary file 12 — Original data for Fig. 2a–d. [file 42255_2024_991_MOESM12_ESM.zip › Figure 2B/Mouse 17/1818-2 MidARH1.jpg]

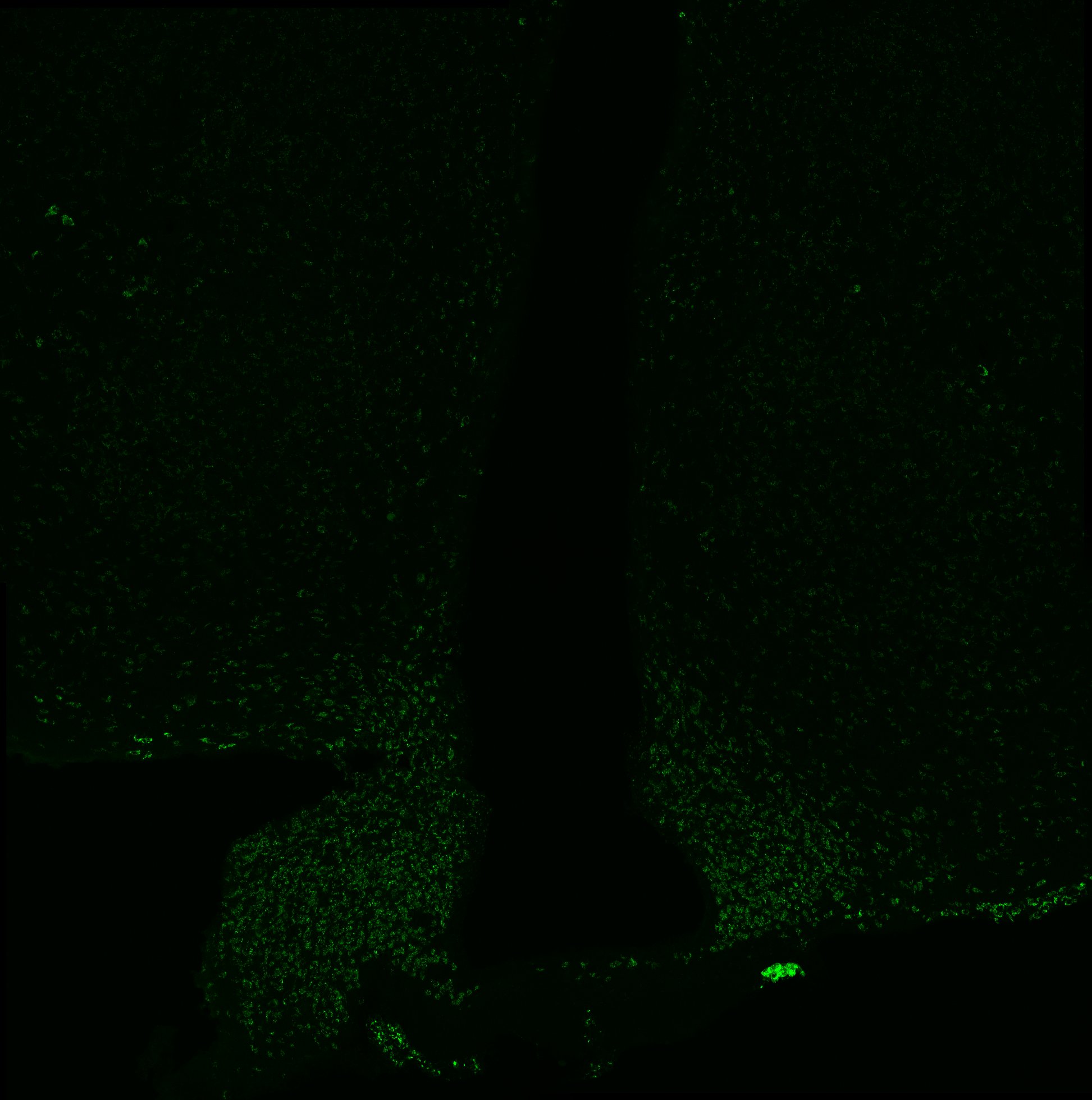

Supplement: Supplementary file 12 — Original data for Fig. 2a–d. [file 42255_2024_991_MOESM12_ESM.zip › Figure 2B/Mouse 17/1818-2 MidARH3.jpg]

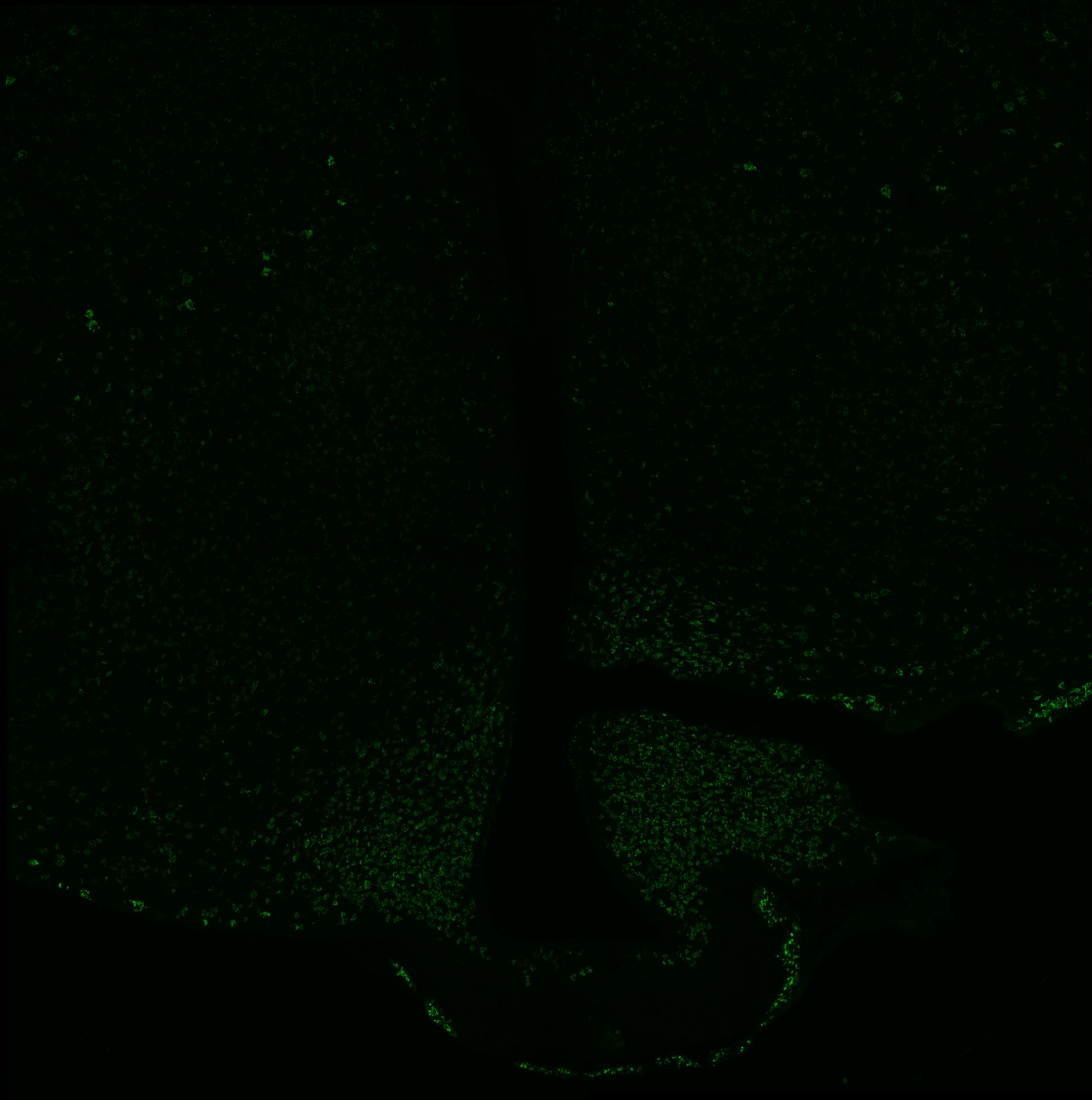

Supplement: Supplementary file 12 — Original data for Fig. 2a–d. [file 42255_2024_991_MOESM12_ESM.zip › Figure 2B/Mouse 17/1818-2 MidARH2.jpg]

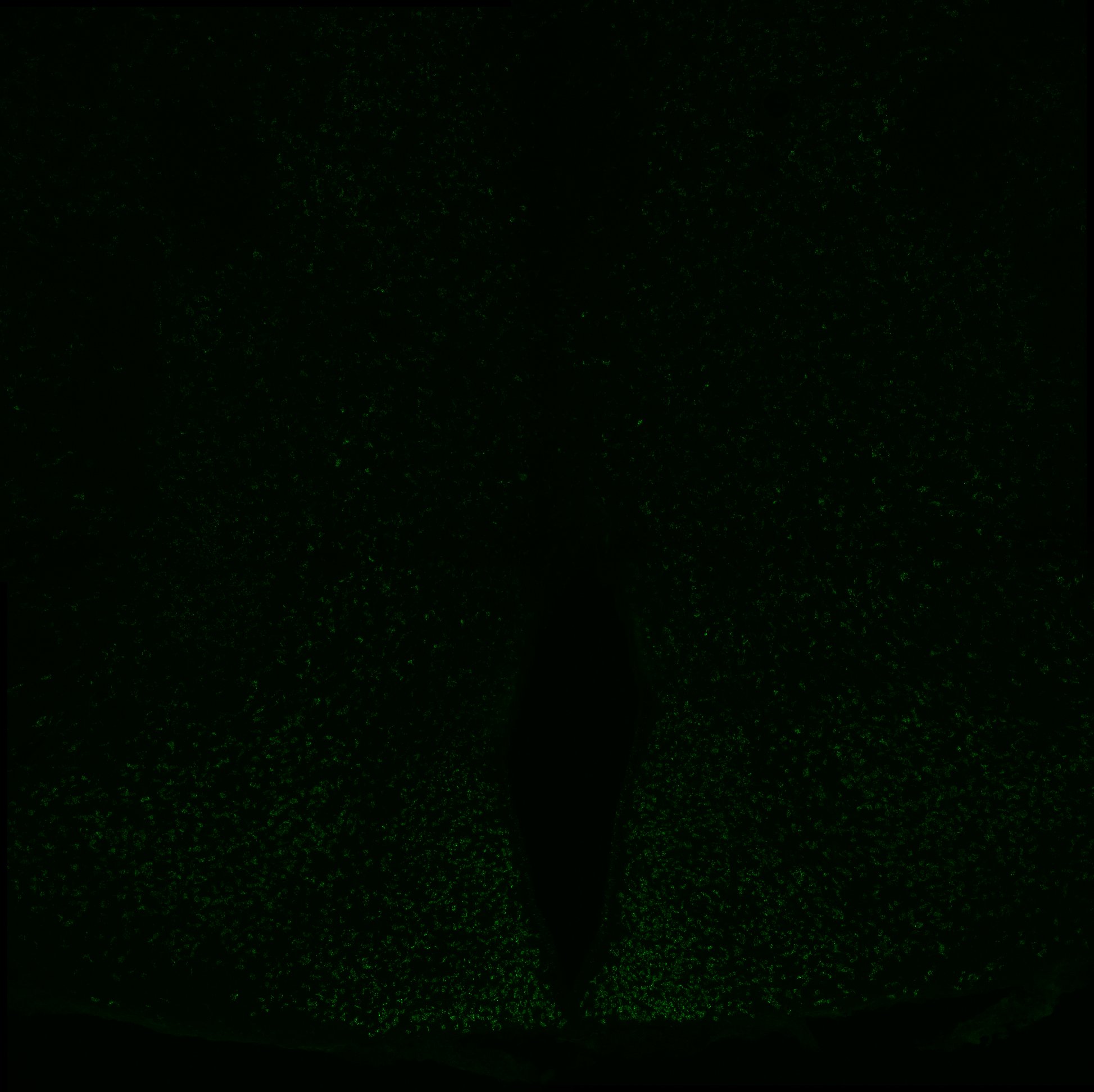

Supplement: Supplementary file 12 — Original data for Fig. 2a–d. [file 42255_2024_991_MOESM12_ESM.zip › Figure 2B/Mouse 17/1818-2 PostARH.jpg]

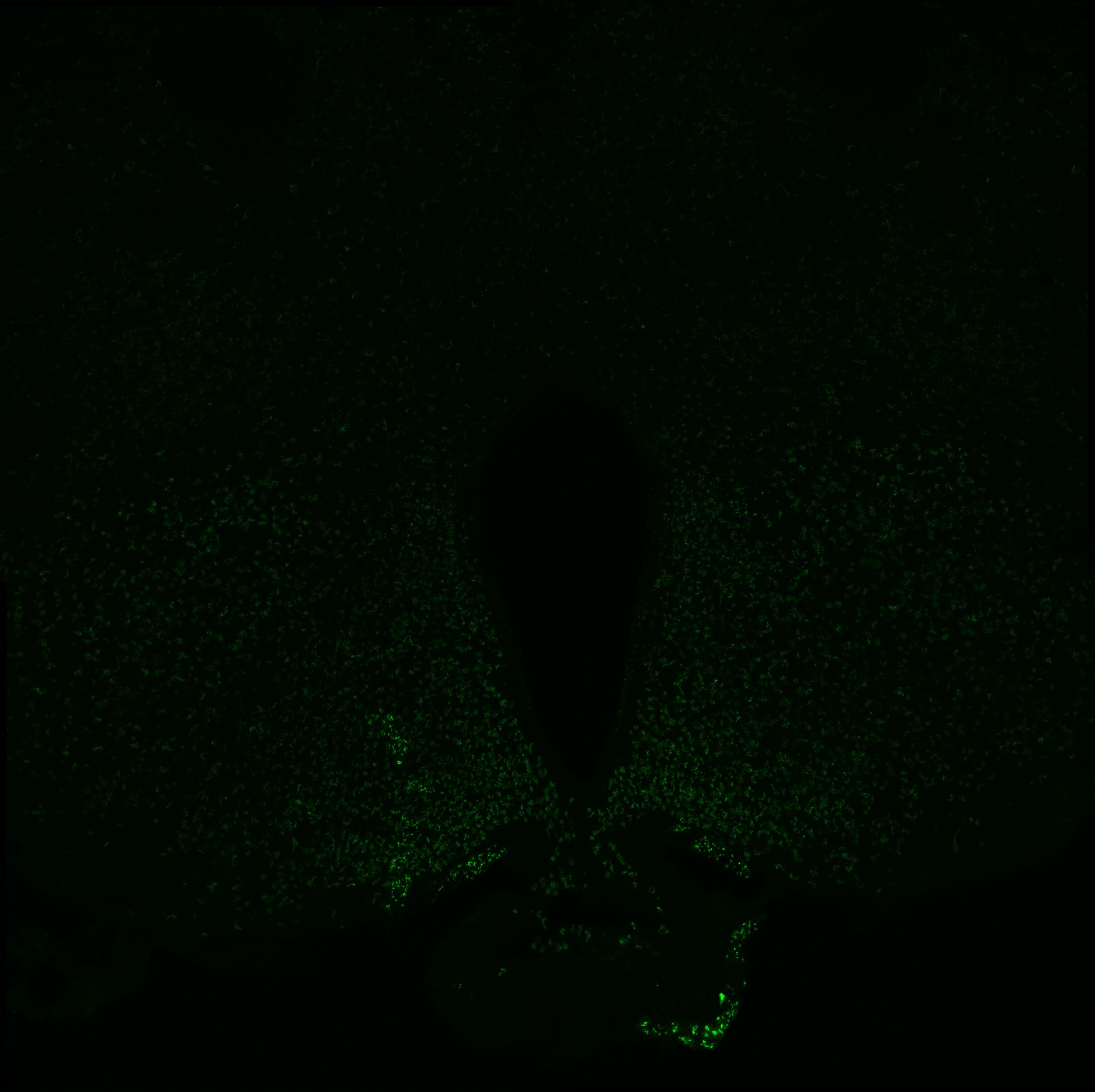

Supplement: Supplementary file 12 — Original data for Fig. 2a–d. [file 42255_2024_991_MOESM12_ESM.zip › Figure 2B/Mouse 28/1821-3 PostARH.jpg]

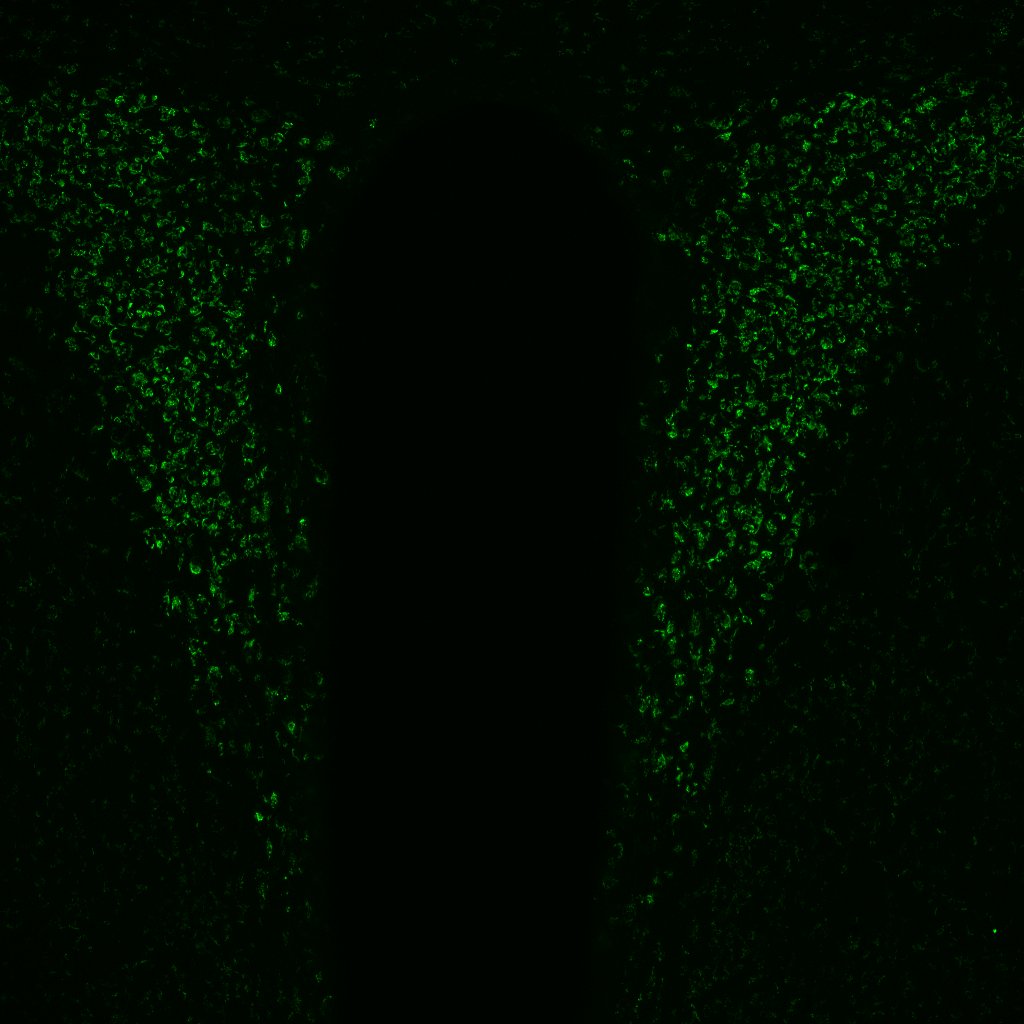

Supplement: Supplementary file 12 — Original data for Fig. 2a–d. [file 42255_2024_991_MOESM12_ESM.zip › Figure 2B/Mouse 28/1821-3 PVH.jpg]

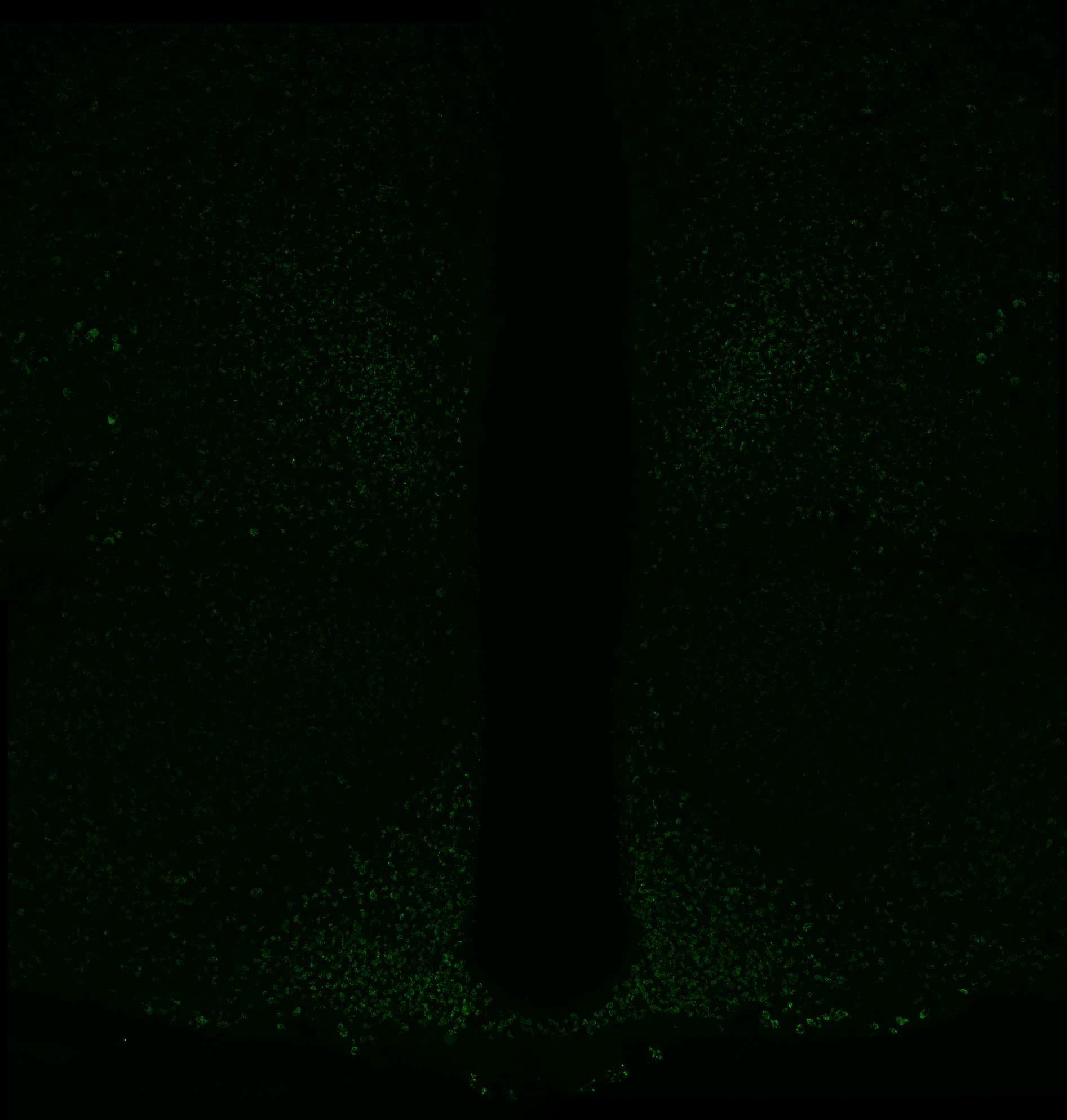

Supplement: Supplementary file 12 — Original data for Fig. 2a–d. [file 42255_2024_991_MOESM12_ESM.zip › Figure 2B/Mouse 28/1821-3 MidARH2.jpg]

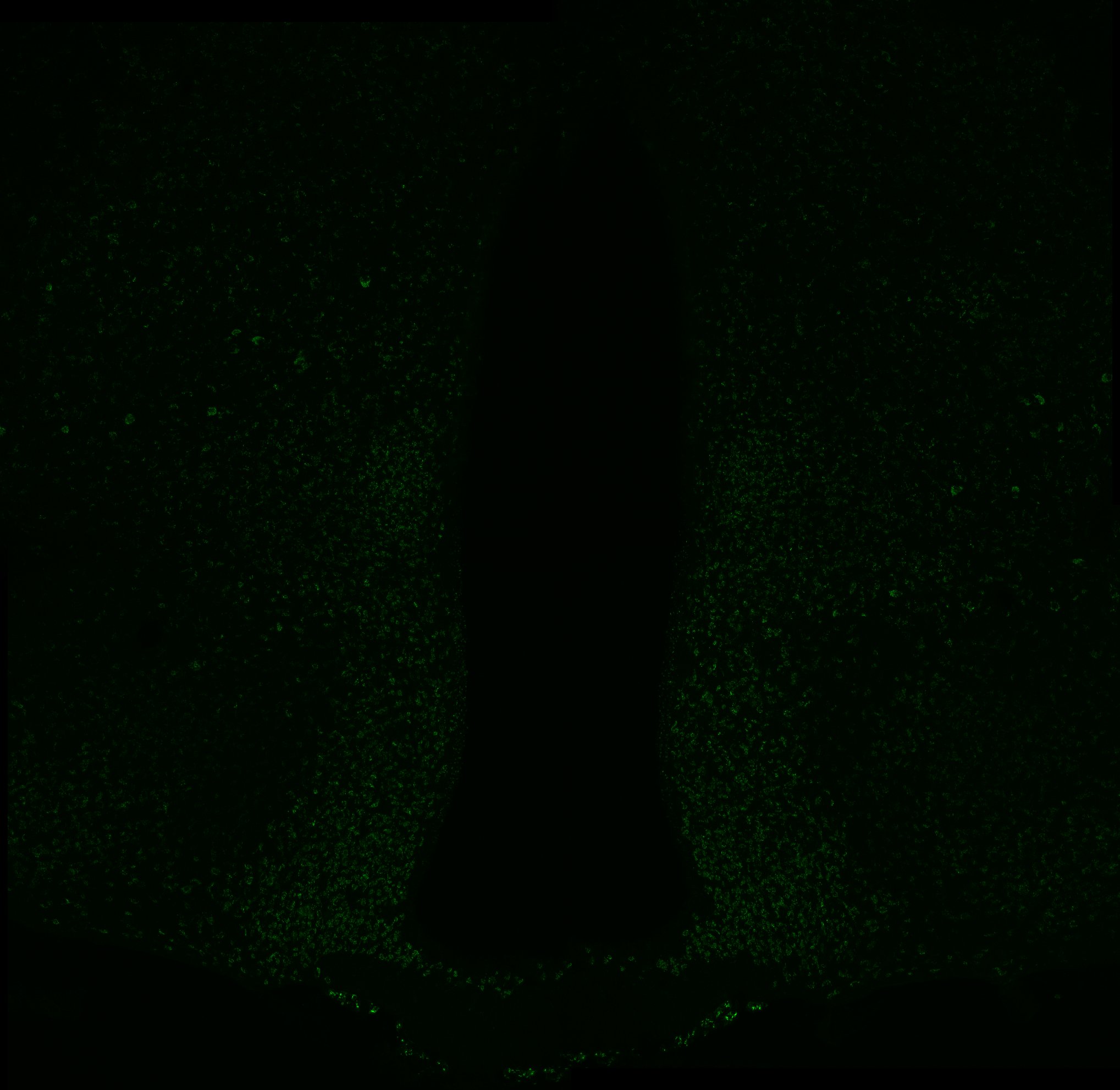

Supplement: Supplementary file 12 — Original data for Fig. 2a–d. [file 42255_2024_991_MOESM12_ESM.zip › Figure 2B/Mouse 28/1821-3 MidARH3.jpg]

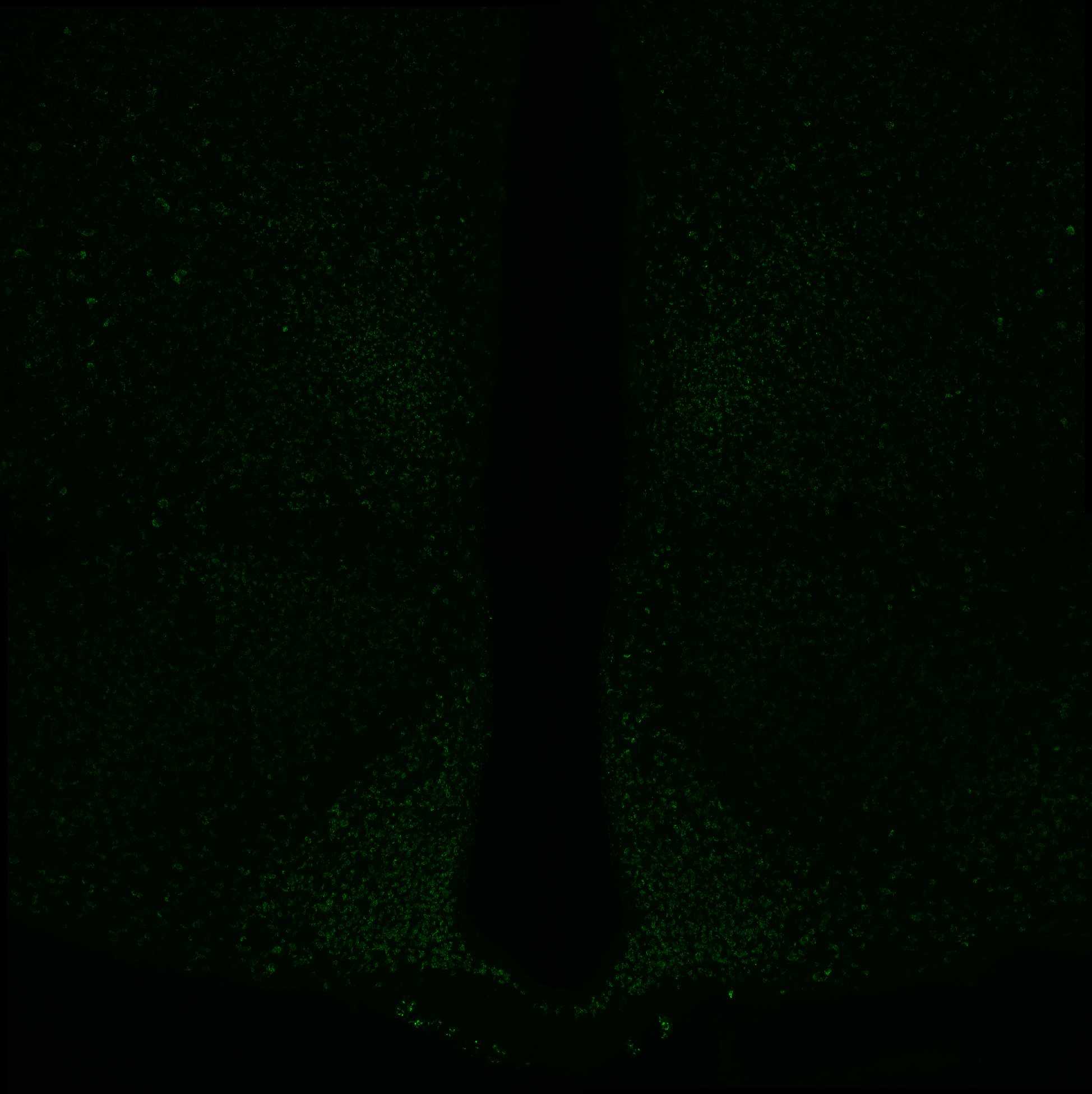

Supplement: Supplementary file 12 — Original data for Fig. 2a–d. [file 42255_2024_991_MOESM12_ESM.zip › Figure 2B/Mouse 28/1821-3 MidARH1.jpg]

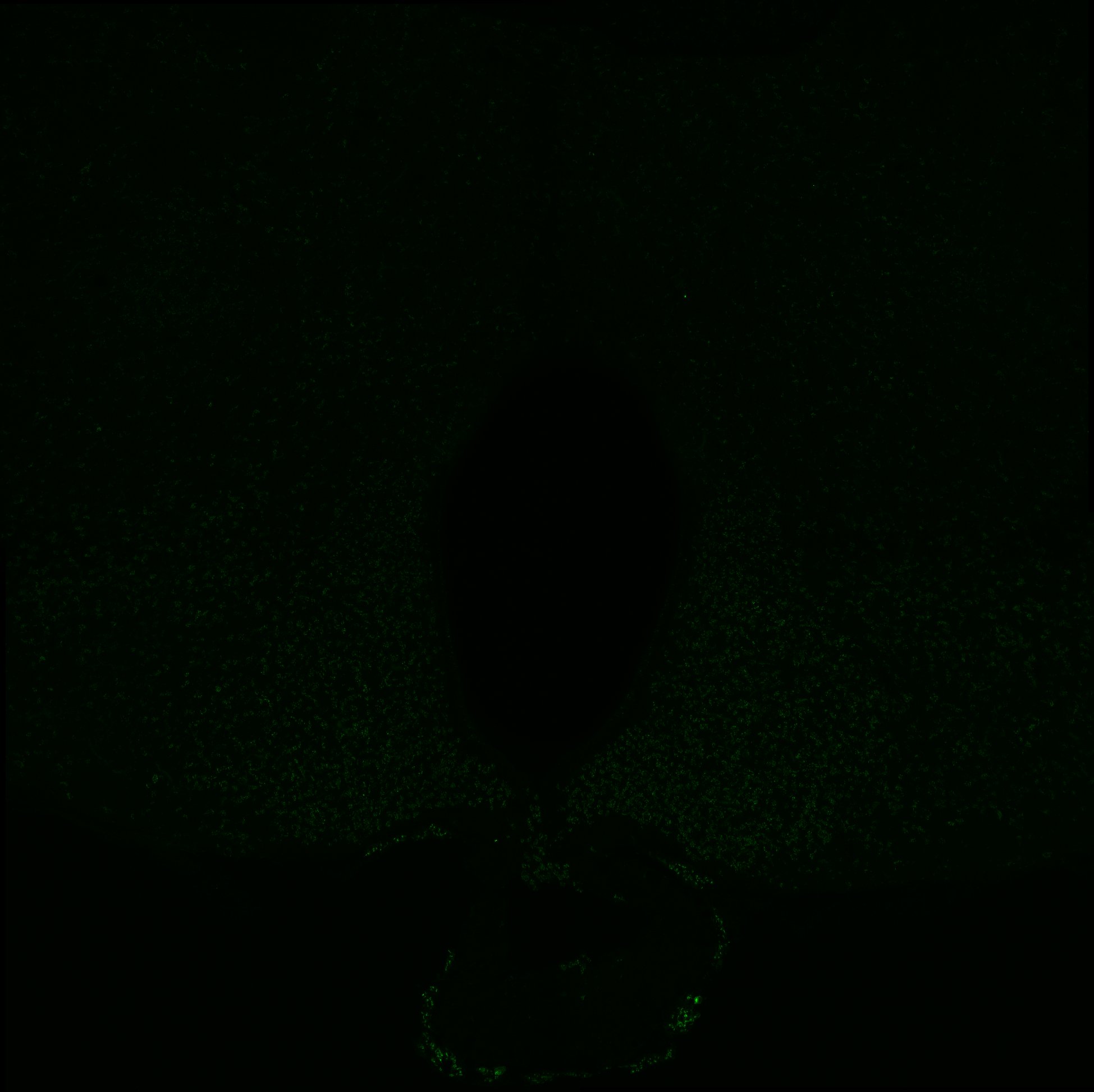

Supplement: Supplementary file 12 — Original data for Fig. 2a–d. [file 42255_2024_991_MOESM12_ESM.zip › Figure 2B/Mouse 10/1845-5 PostARH.jpg]

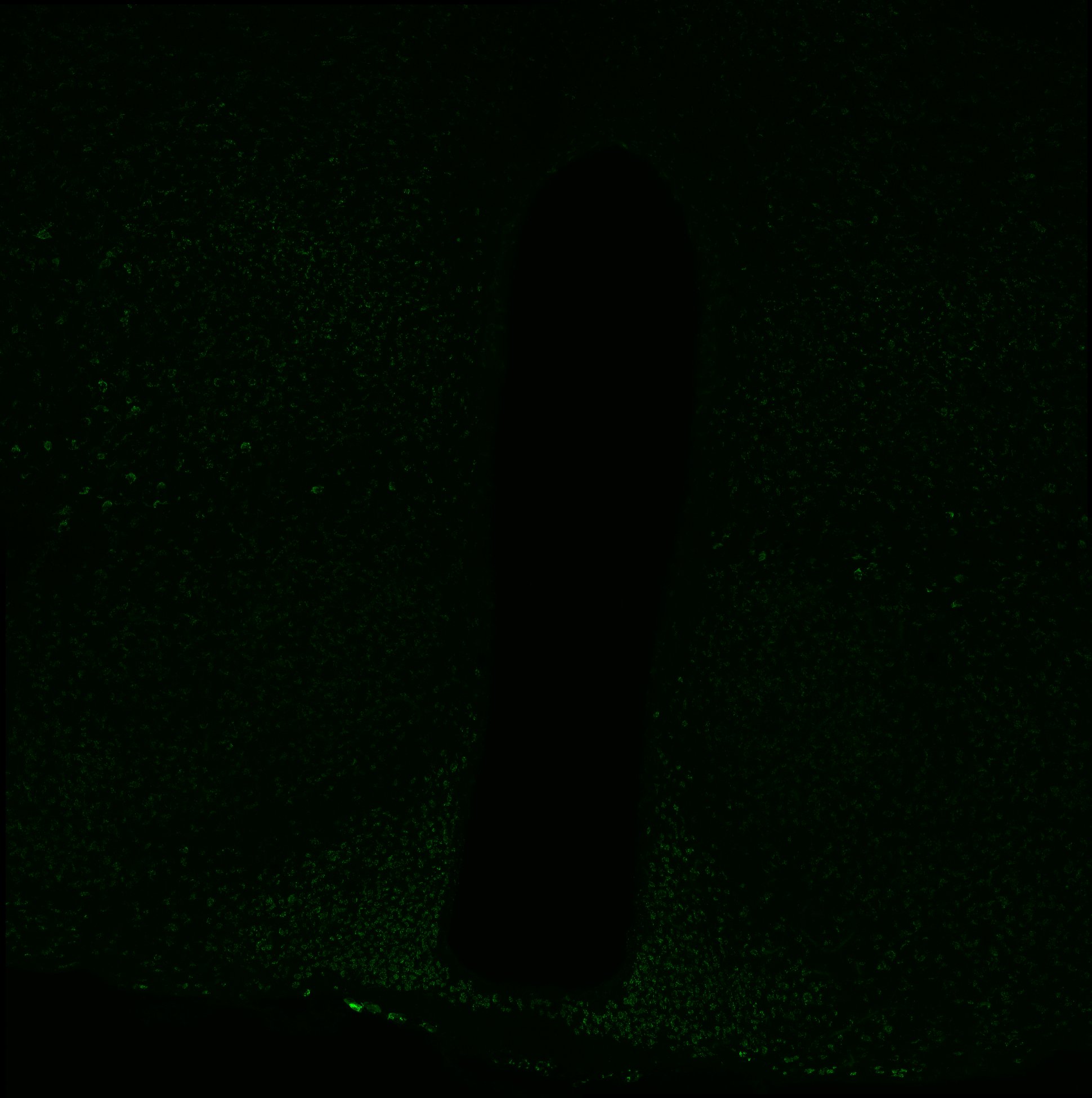

Supplement: Supplementary file 12 — Original data for Fig. 2a–d. [file 42255_2024_991_MOESM12_ESM.zip › Figure 2B/Mouse 10/1845-5 MidARH2.jpg]

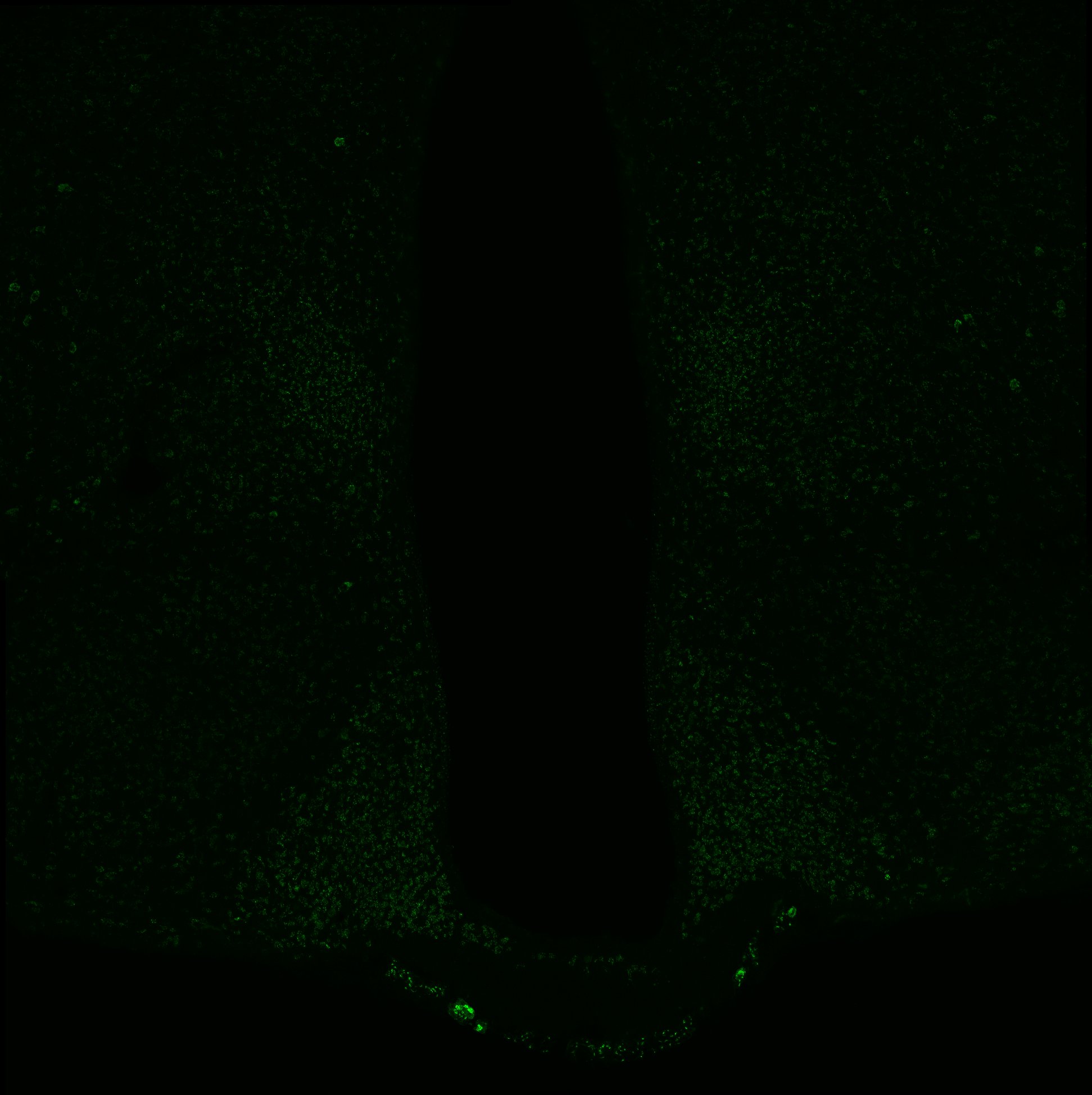

Supplement: Supplementary file 12 — Original data for Fig. 2a–d. [file 42255_2024_991_MOESM12_ESM.zip › Figure 2B/Mouse 10/1845-5 MidARH1.jpg]

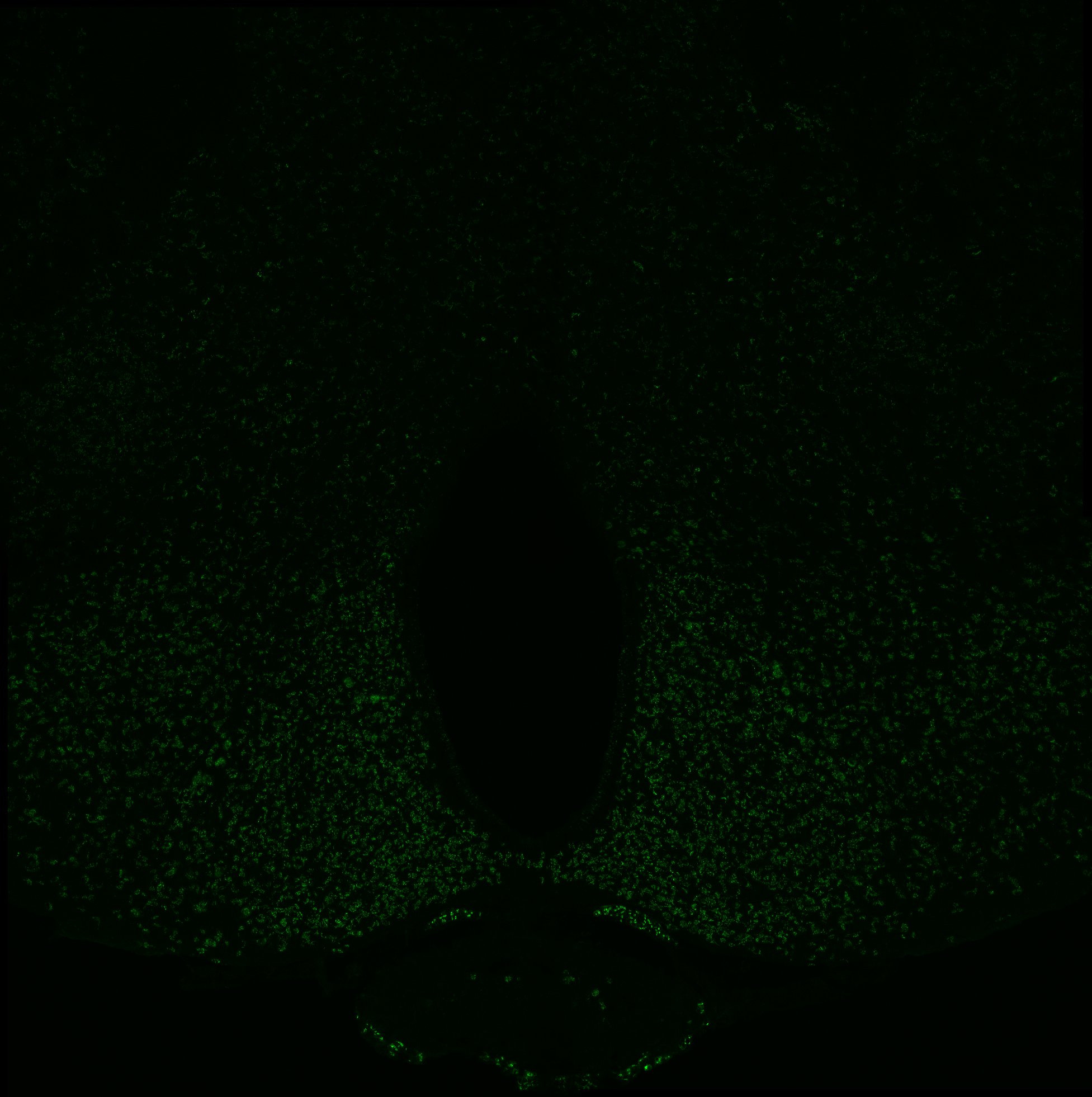

Supplement: Supplementary file 12 — Original data for Fig. 2a–d. [file 42255_2024_991_MOESM12_ESM.zip › Figure 2B/Mouse 11/1813-1 PostARH.jpg]

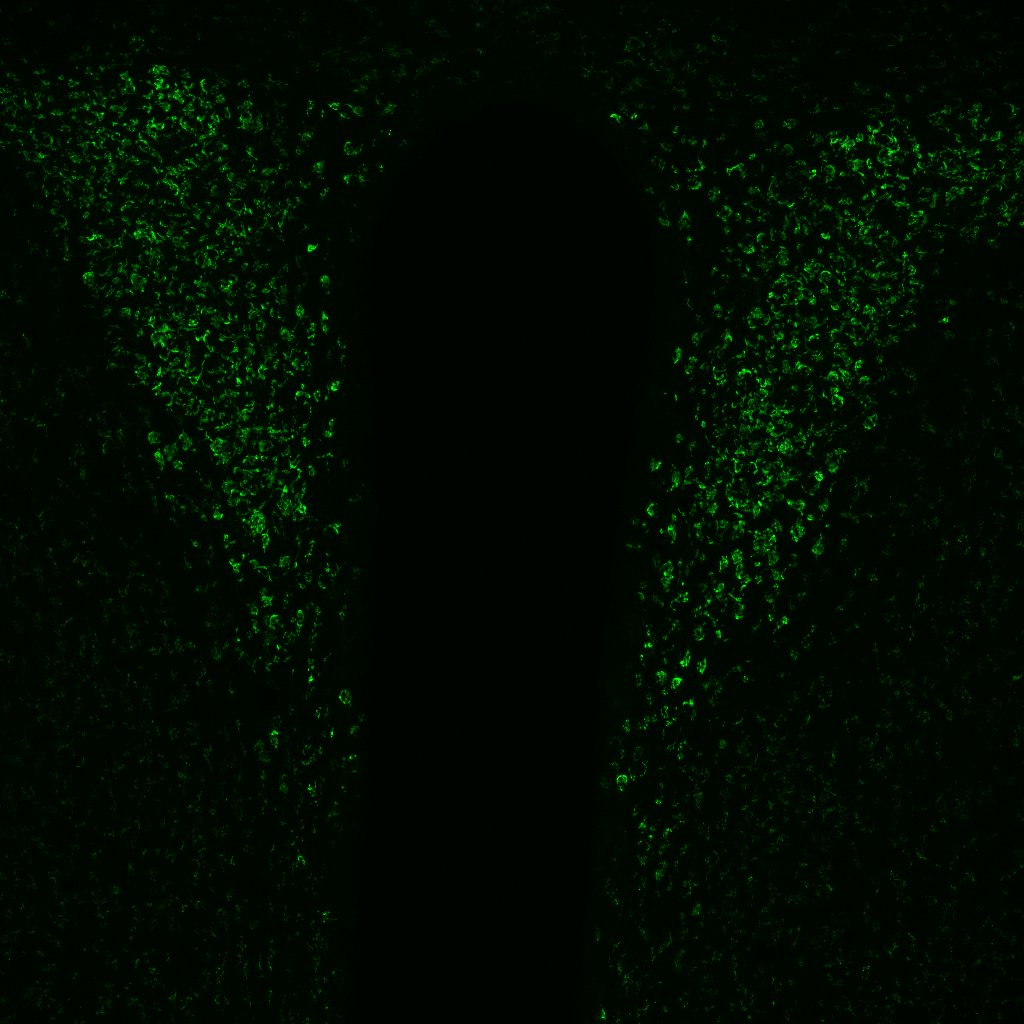

Supplement: Supplementary file 12 — Original data for Fig. 2a–d. [file 42255_2024_991_MOESM12_ESM.zip › Figure 2B/Mouse 11/1813-1 PVH1.jpg]

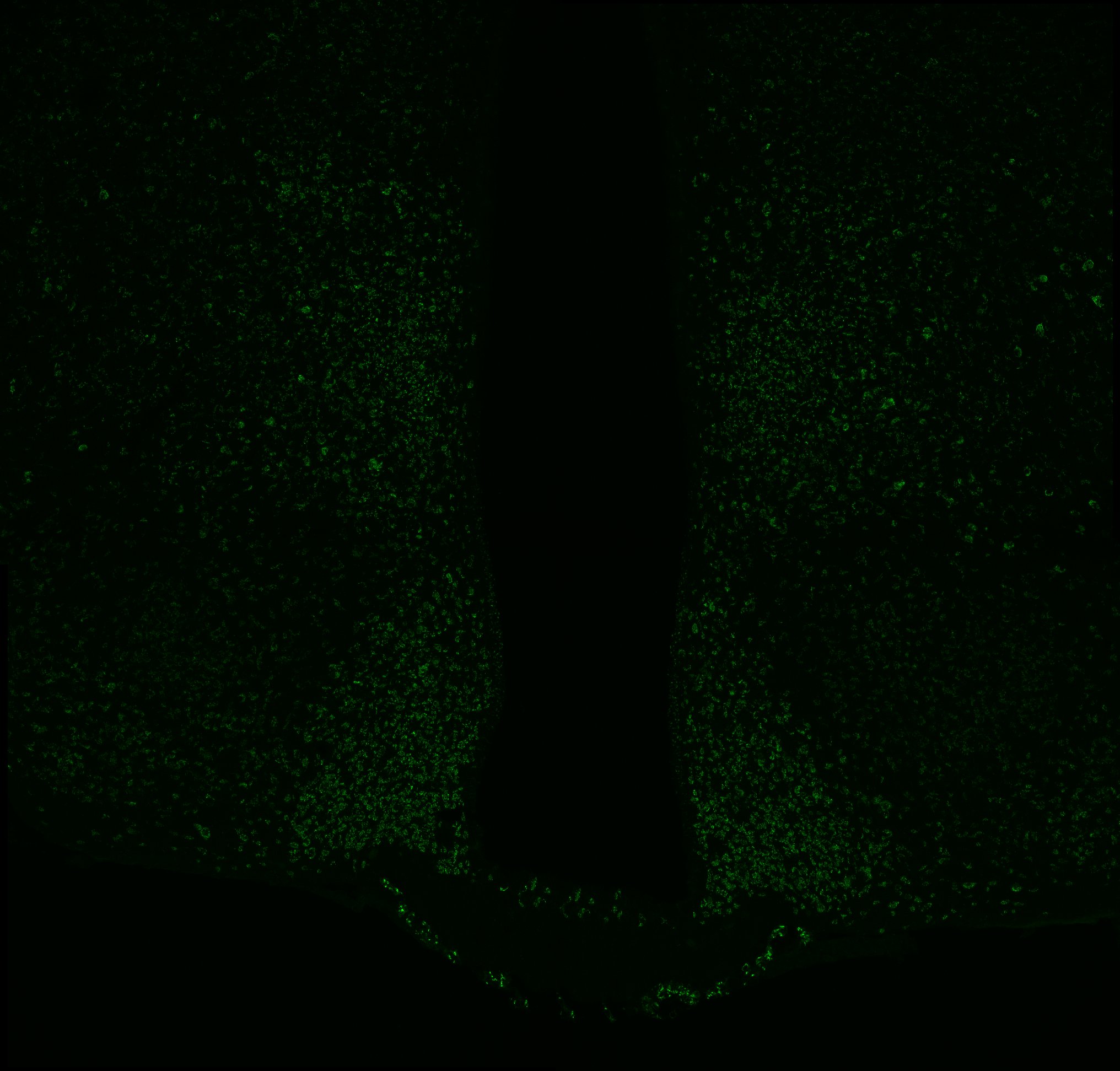

Supplement: Supplementary file 12 — Original data for Fig. 2a–d. [file 42255_2024_991_MOESM12_ESM.zip › Figure 2B/Mouse 11/1813-1 MidARH1.jpg]

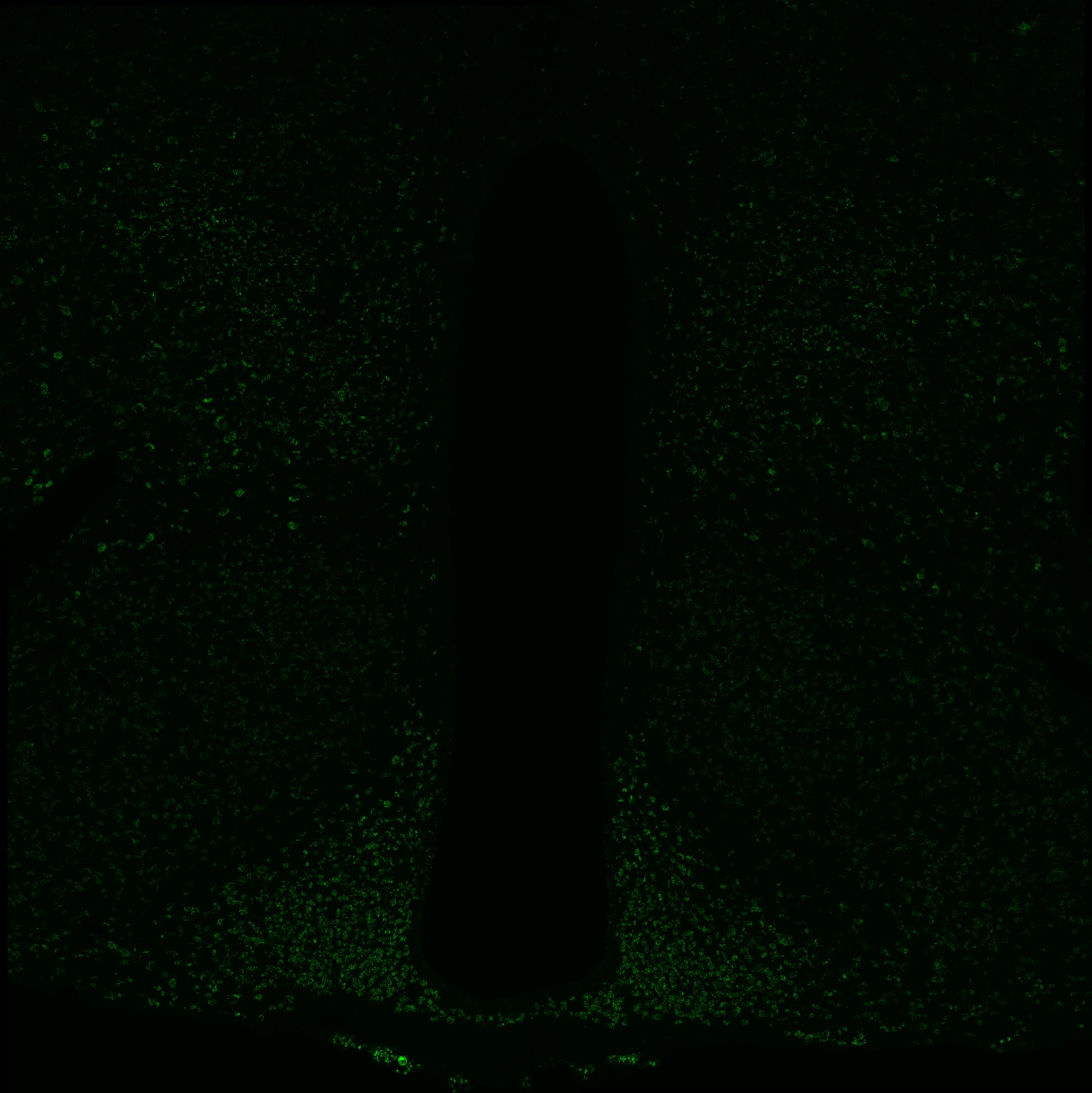

Supplement: Supplementary file 12 — Original data for Fig. 2a–d. [file 42255_2024_991_MOESM12_ESM.zip › Figure 2B/Mouse 11/1813-1 MidARH2.jpg]

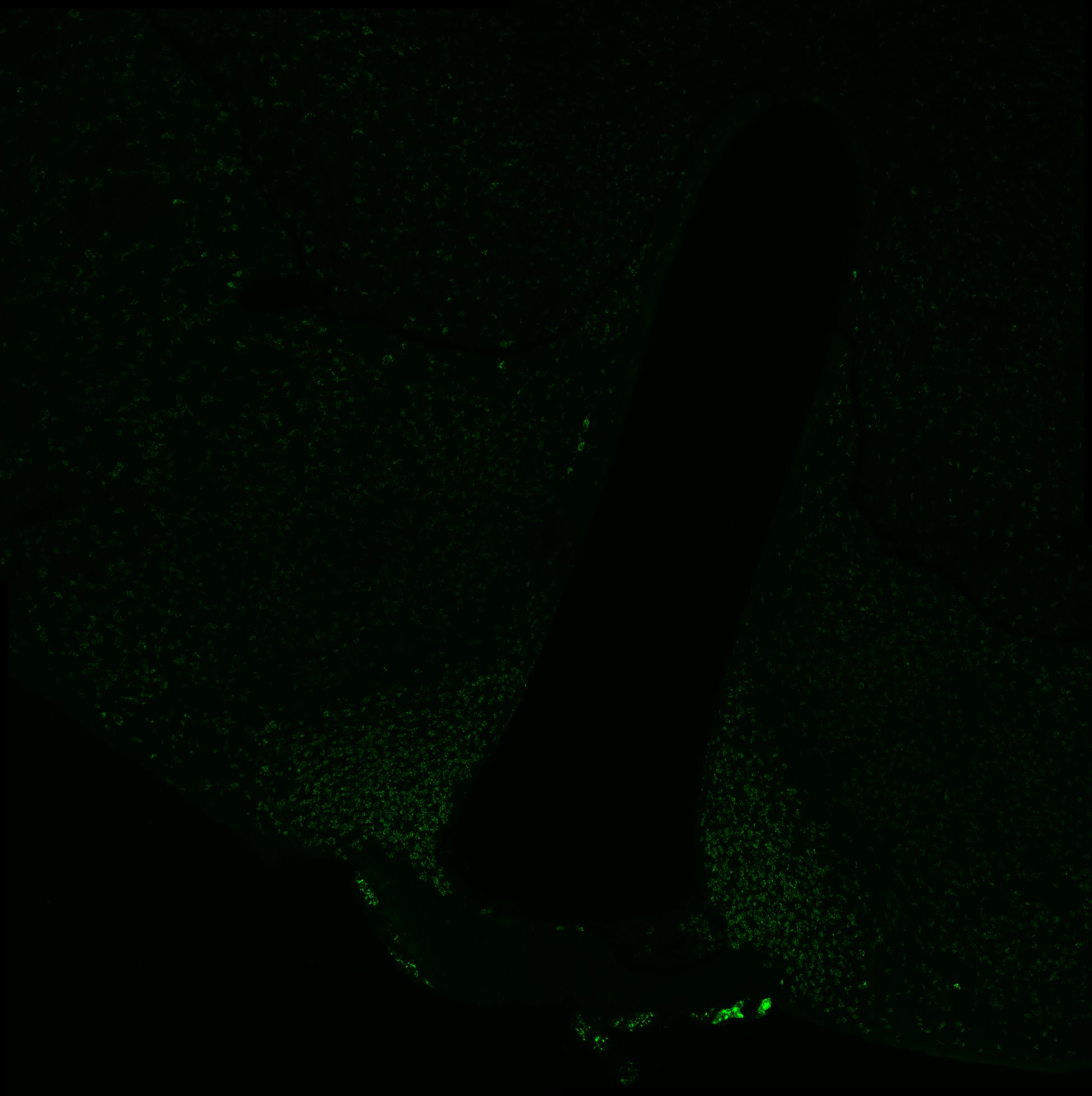

Supplement: Supplementary file 12 — Original data for Fig. 2a–d. [file 42255_2024_991_MOESM12_ESM.zip › Figure 2B/Mouse 16/1818-1 MidARH2.jpg]

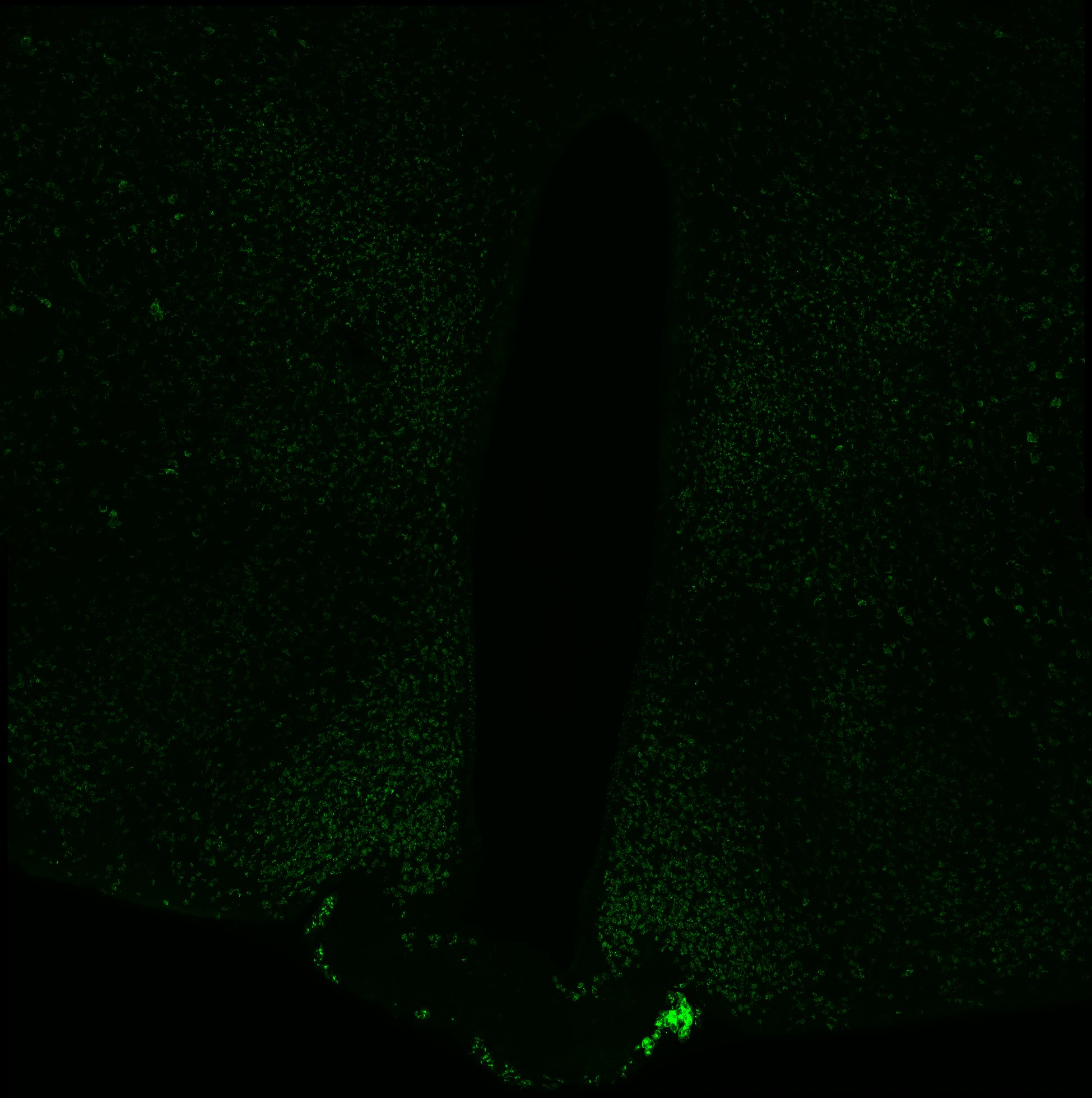

Supplement: Supplementary file 12 — Original data for Fig. 2a–d. [file 42255_2024_991_MOESM12_ESM.zip › Figure 2B/Mouse 16/1818-1 MidARH1.jpg]

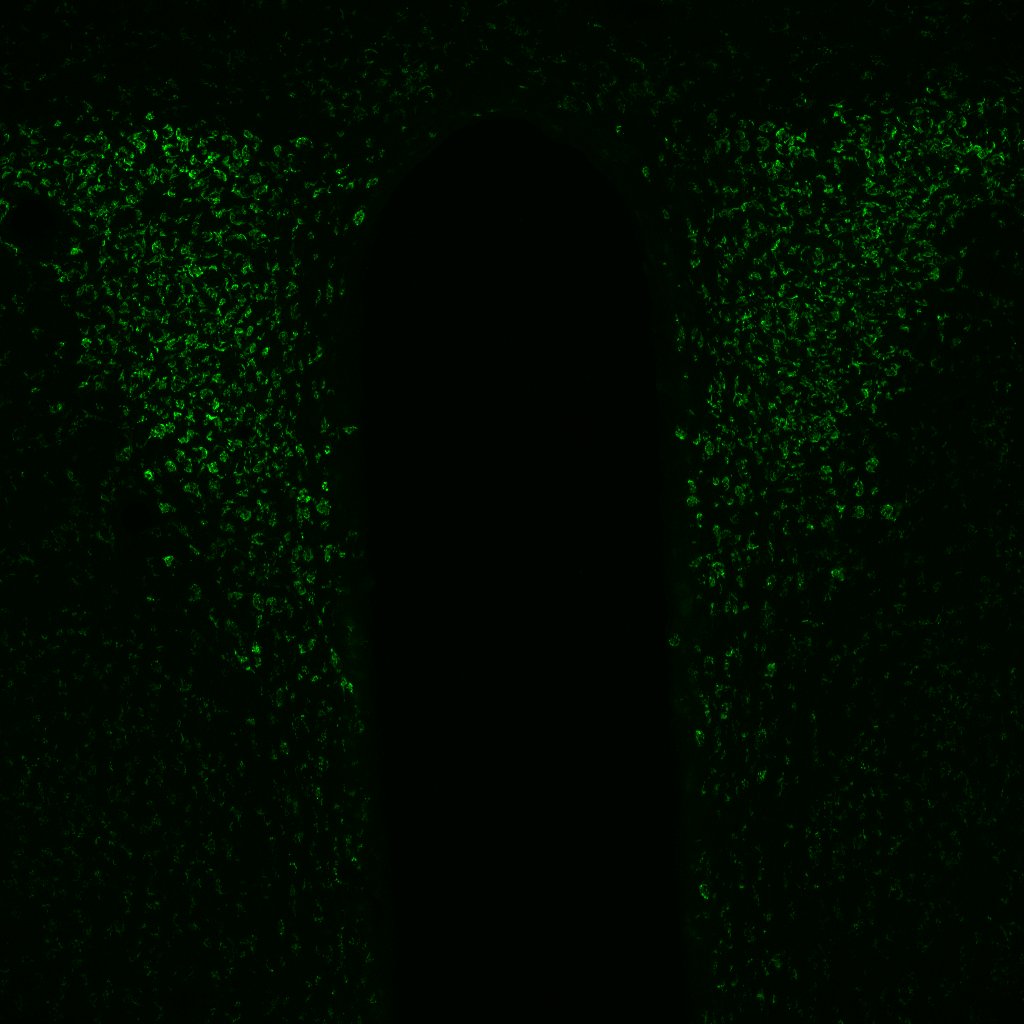

Supplement: Supplementary file 12 — Original data for Fig. 2a–d. [file 42255_2024_991_MOESM12_ESM.zip › Figure 2B/Mouse 16/1818-1 PVH1.jpg]

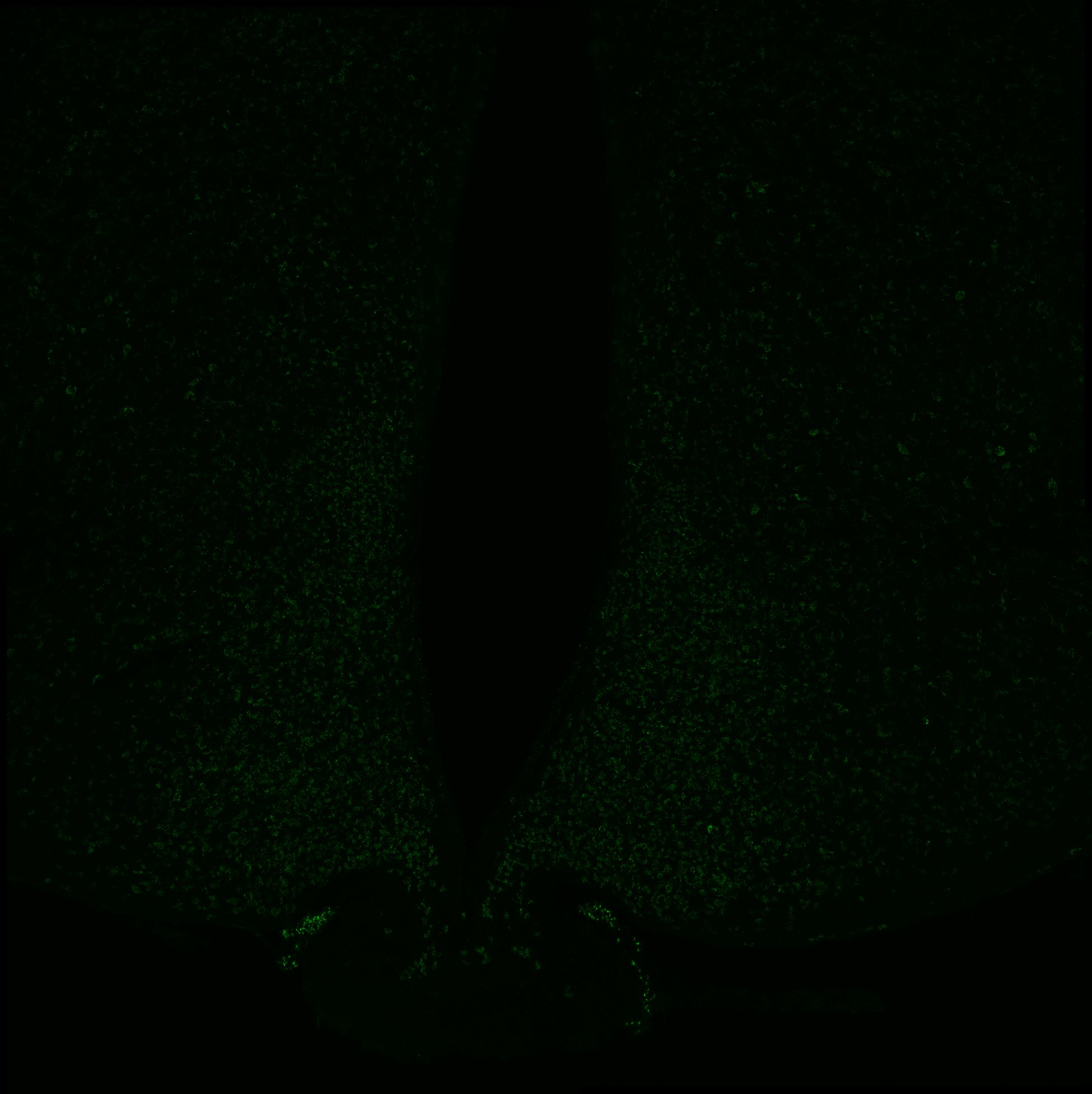

Supplement: Supplementary file 12 — Original data for Fig. 2a–d. [file 42255_2024_991_MOESM12_ESM.zip › Figure 2B/Mouse 29/1821-4 MidARH1.jpg]

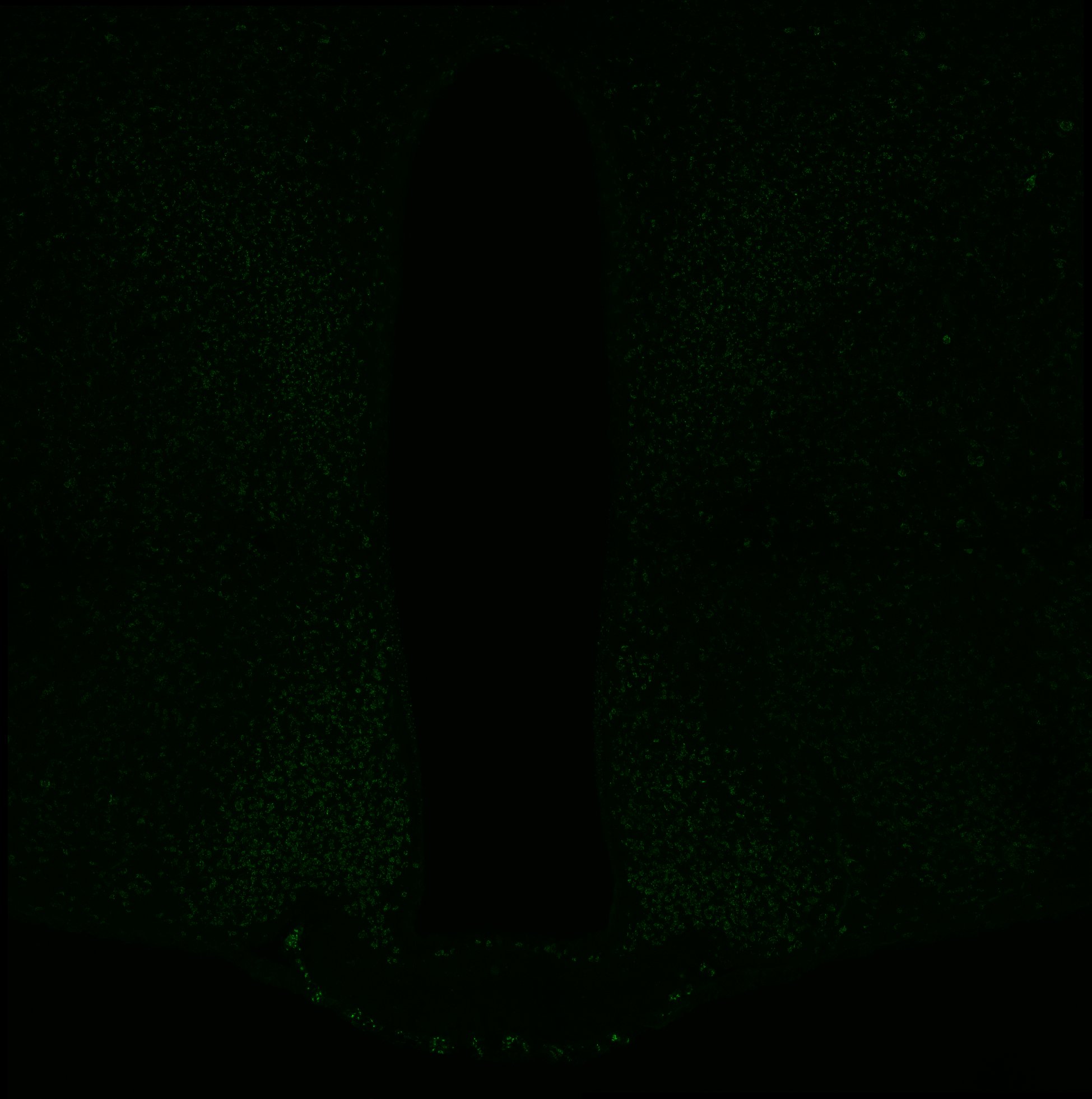

Supplement: Supplementary file 12 — Original data for Fig. 2a–d. [file 42255_2024_991_MOESM12_ESM.zip › Figure 2B/Mouse 29/1821-4 MidARH2.jpg]

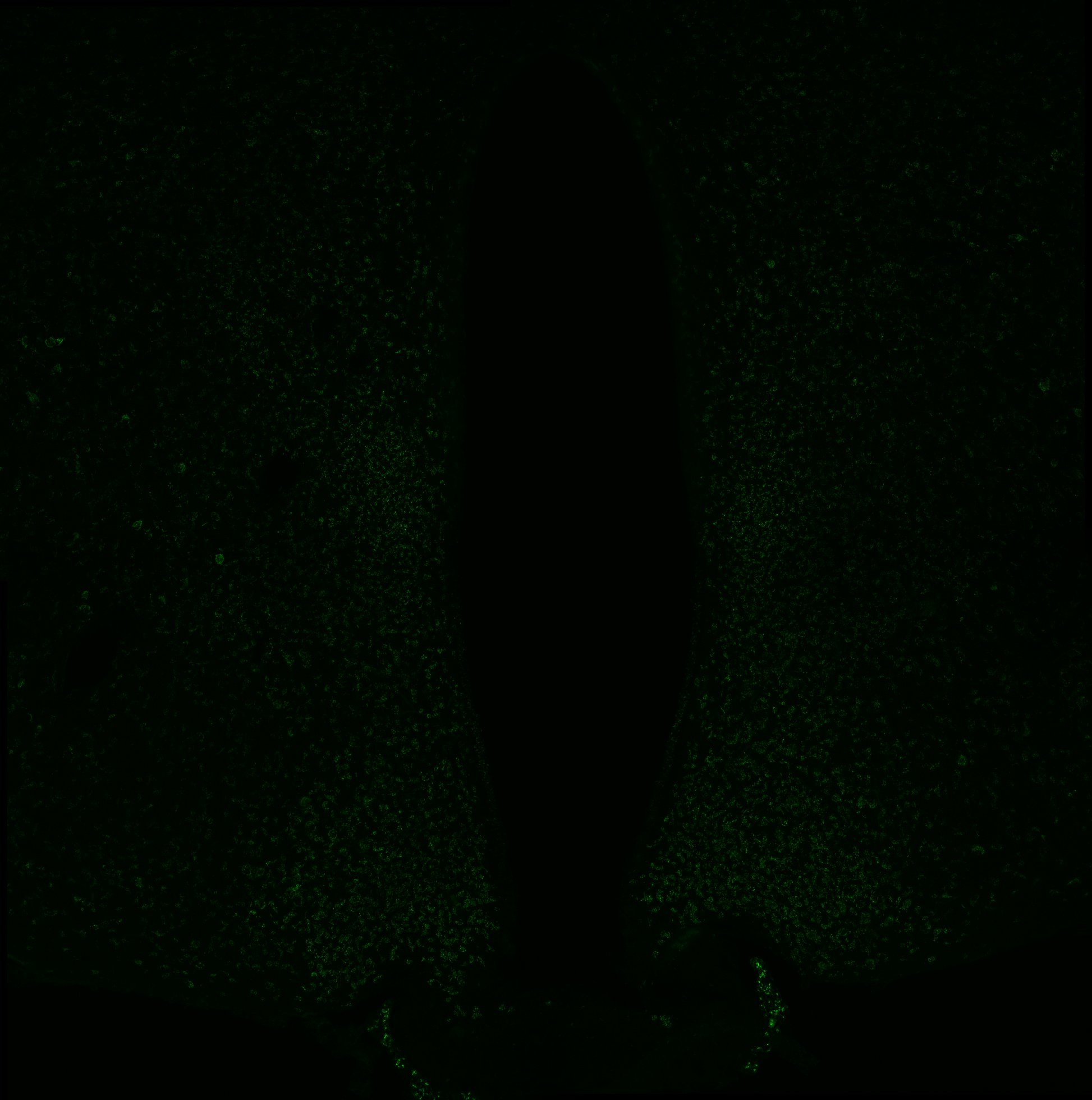

Supplement: Supplementary file 12 — Original data for Fig. 2a–d. [file 42255_2024_991_MOESM12_ESM.zip › Figure 2B/Mouse 29/1821-4 MidARH3.jpg]

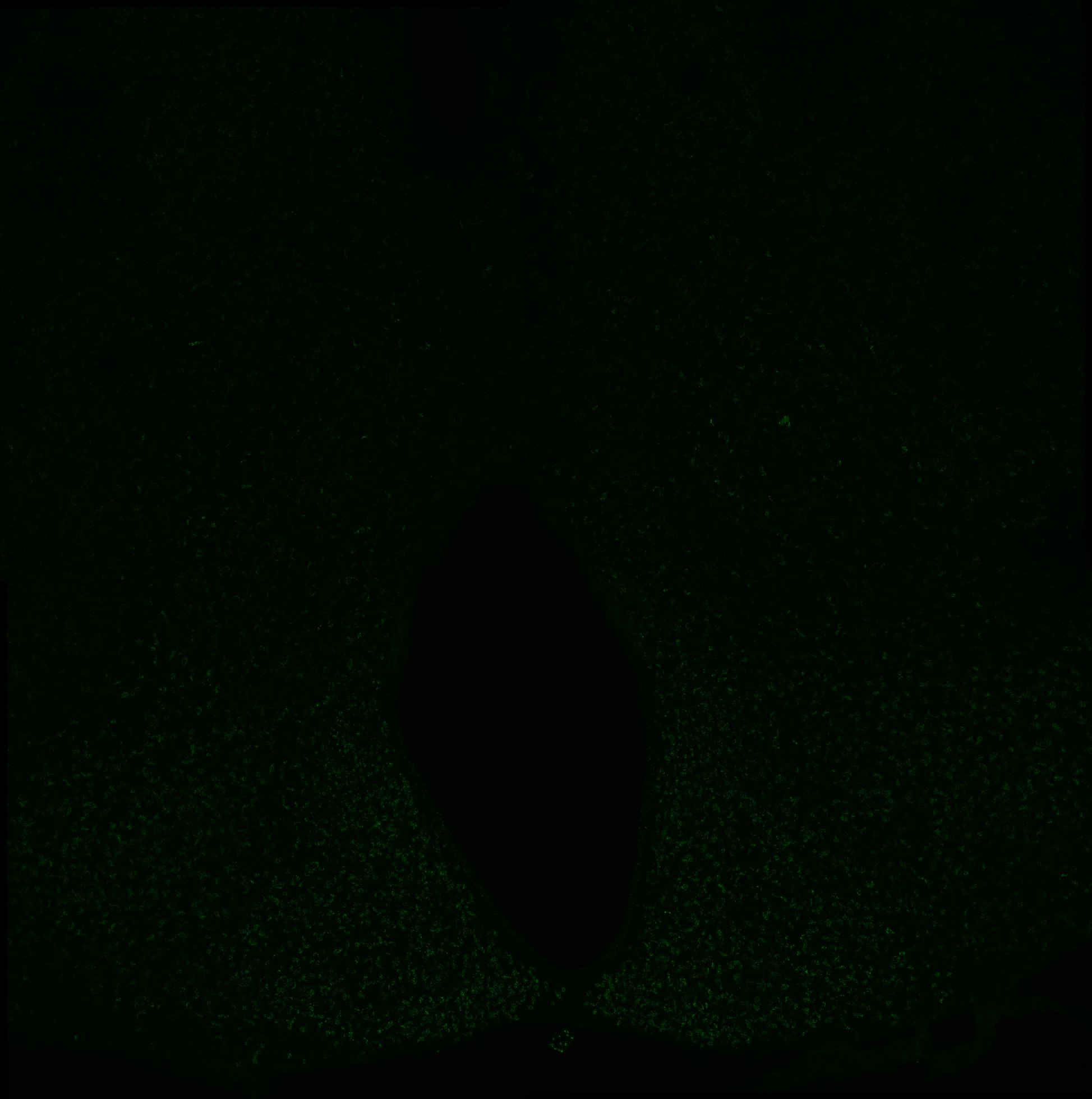

Supplement: Supplementary file 12 — Original data for Fig. 2a–d. [file 42255_2024_991_MOESM12_ESM.zip › Figure 2B/Mouse 29/1821-4 PostARH.jpg]

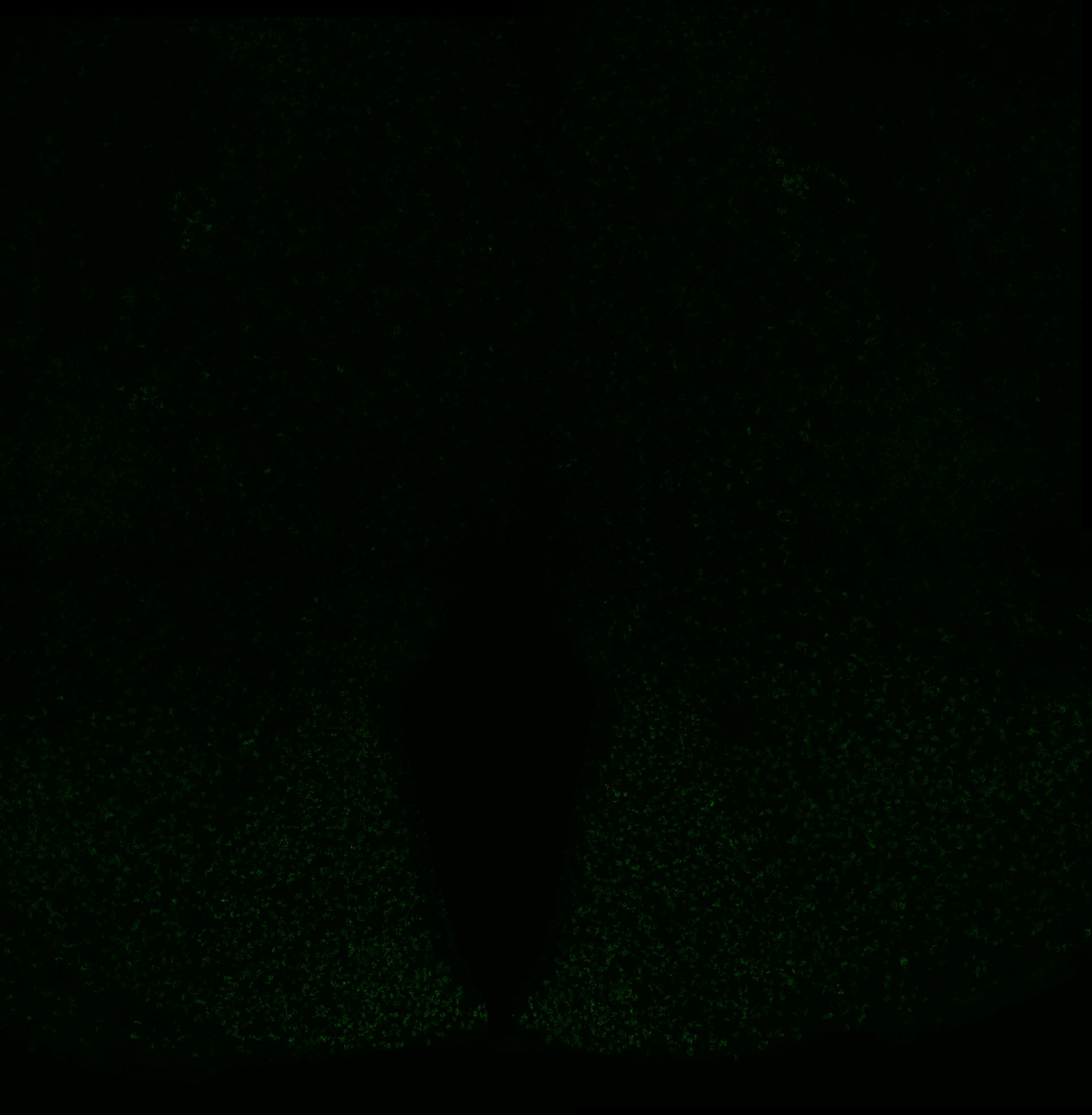

Supplement: Supplementary file 12 — Original data for Fig. 2a–d. [file 42255_2024_991_MOESM12_ESM.zip › Figure 2B/Mouse 20/1818-5 PostARH.jpg]

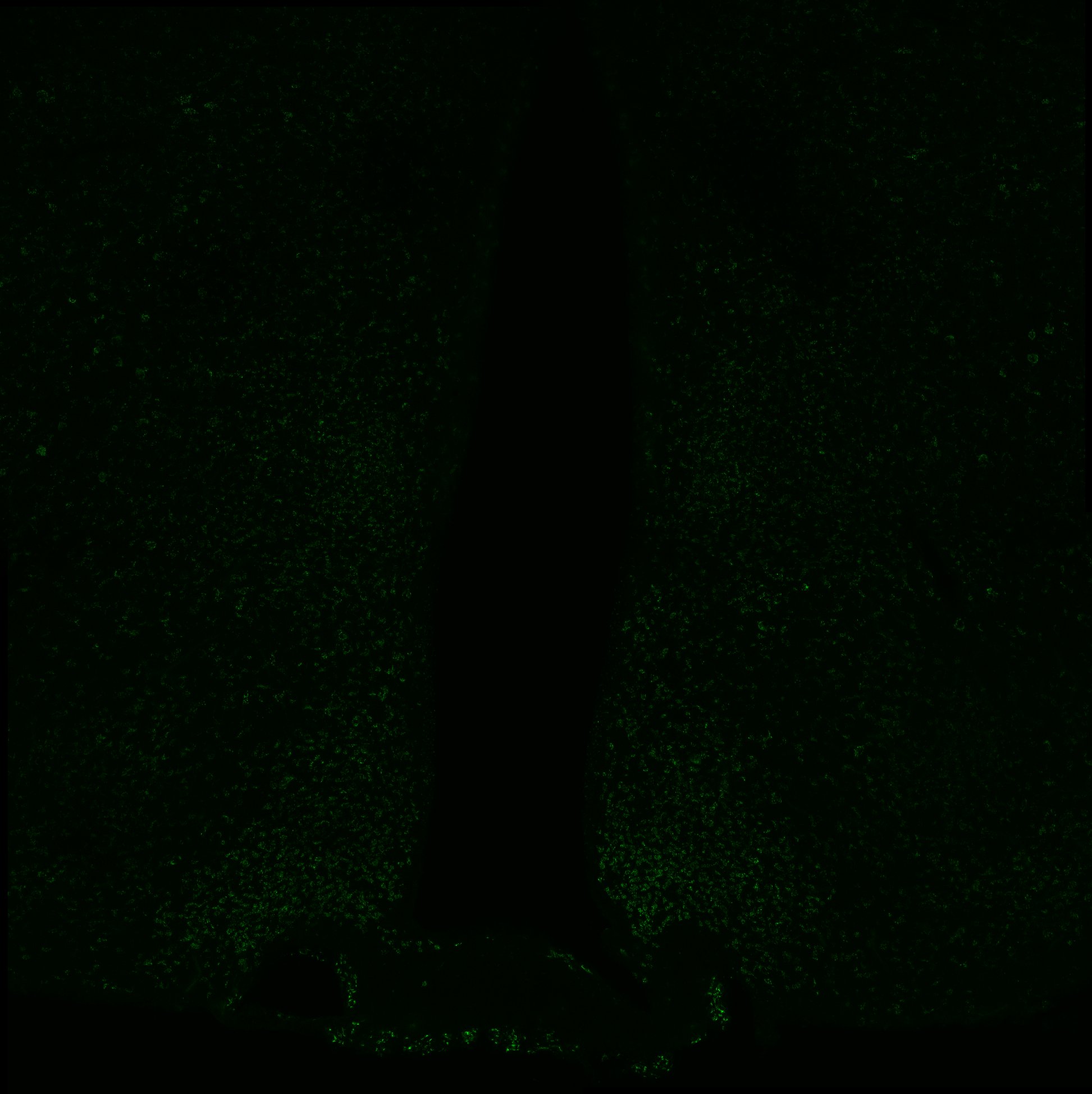

Supplement: Supplementary file 12 — Original data for Fig. 2a–d. [file 42255_2024_991_MOESM12_ESM.zip › Figure 2B/Mouse 20/1818-5 MidARH3.jpg]

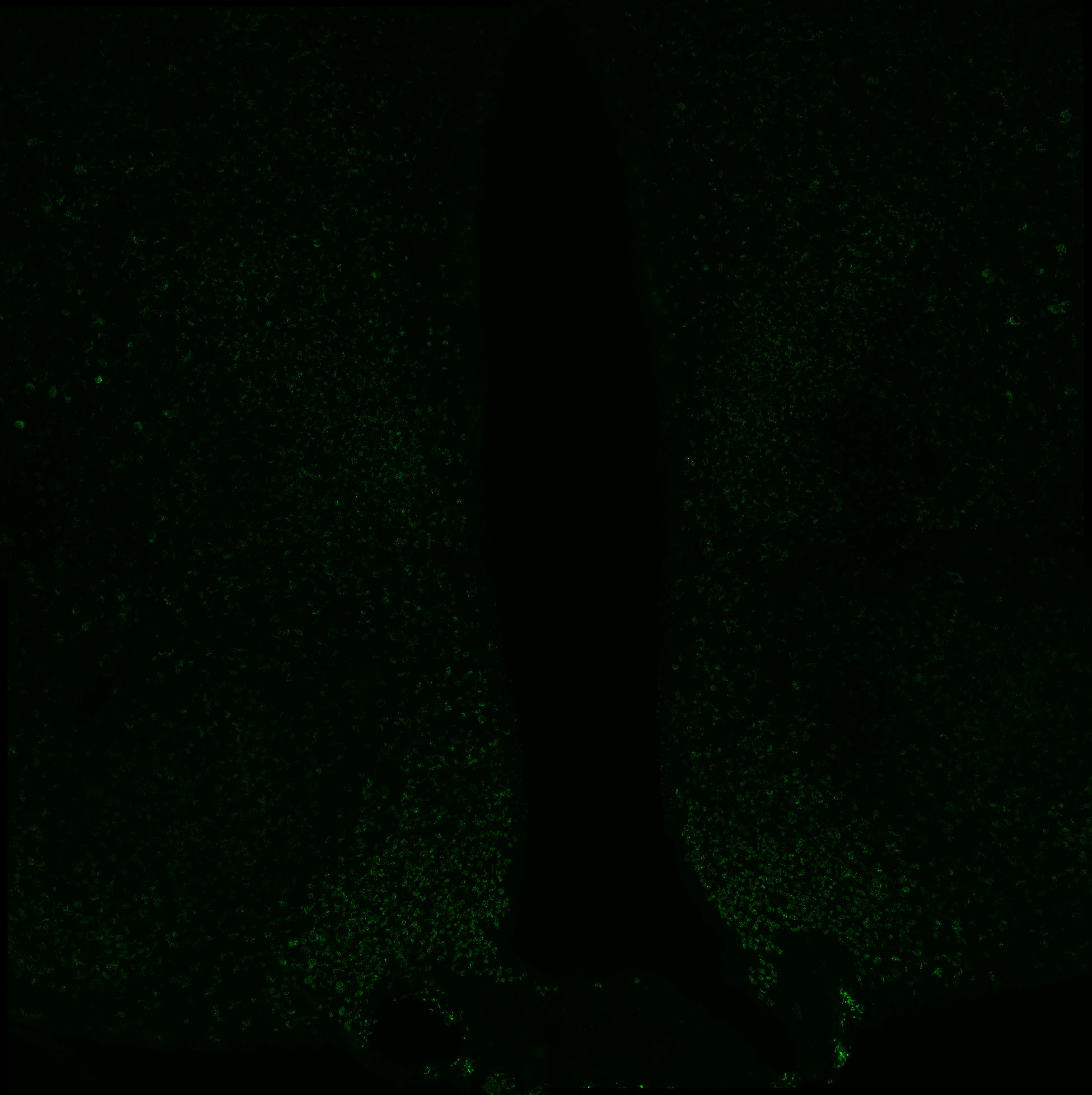

Supplement: Supplementary file 12 — Original data for Fig. 2a–d. [file 42255_2024_991_MOESM12_ESM.zip › Figure 2B/Mouse 20/1818-5 MidARH2.jpg]

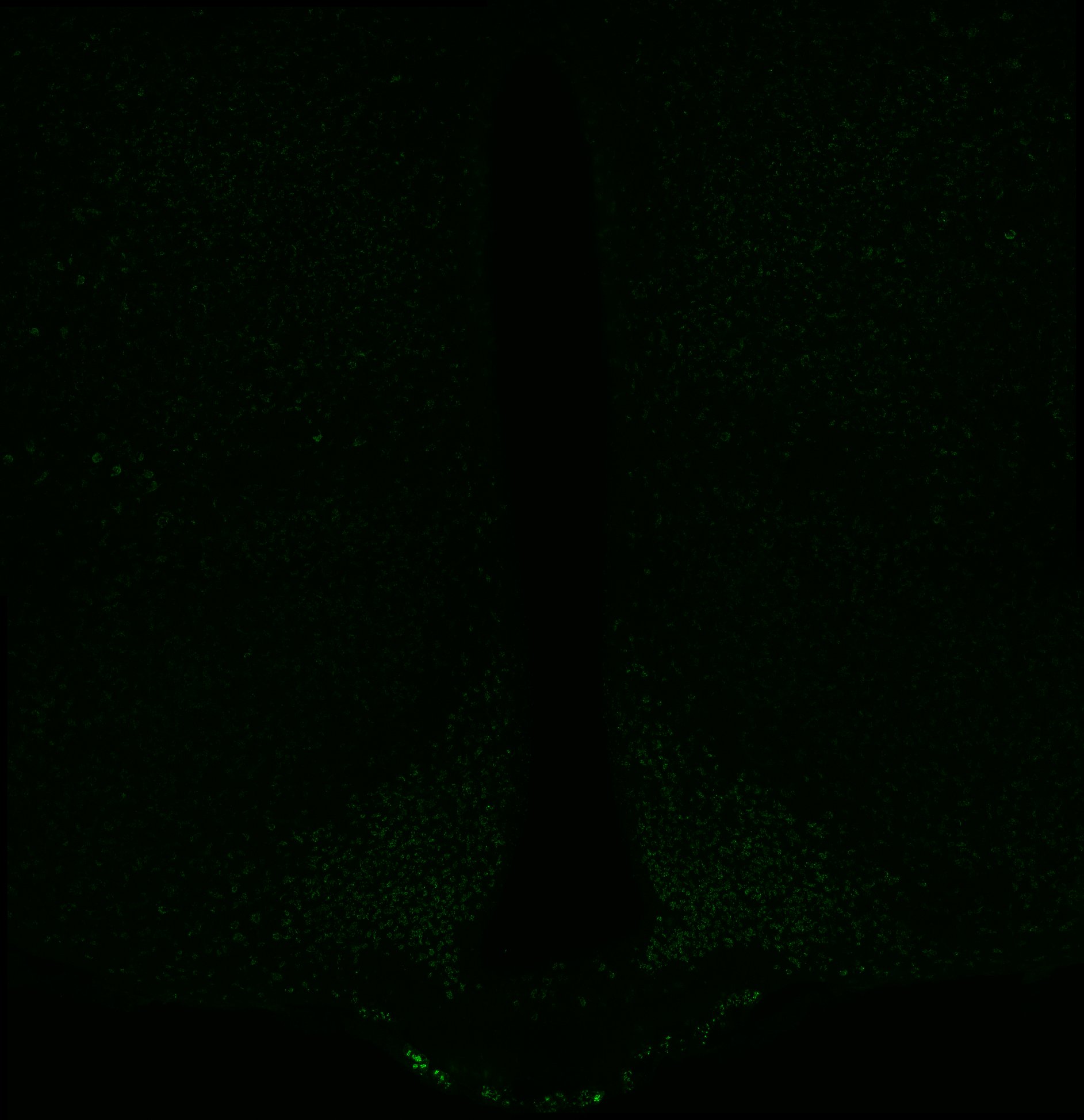

Supplement: Supplementary file 12 — Original data for Fig. 2a–d. [file 42255_2024_991_MOESM12_ESM.zip › Figure 2B/Mouse 20/1818-5 MidARH1.jpg]

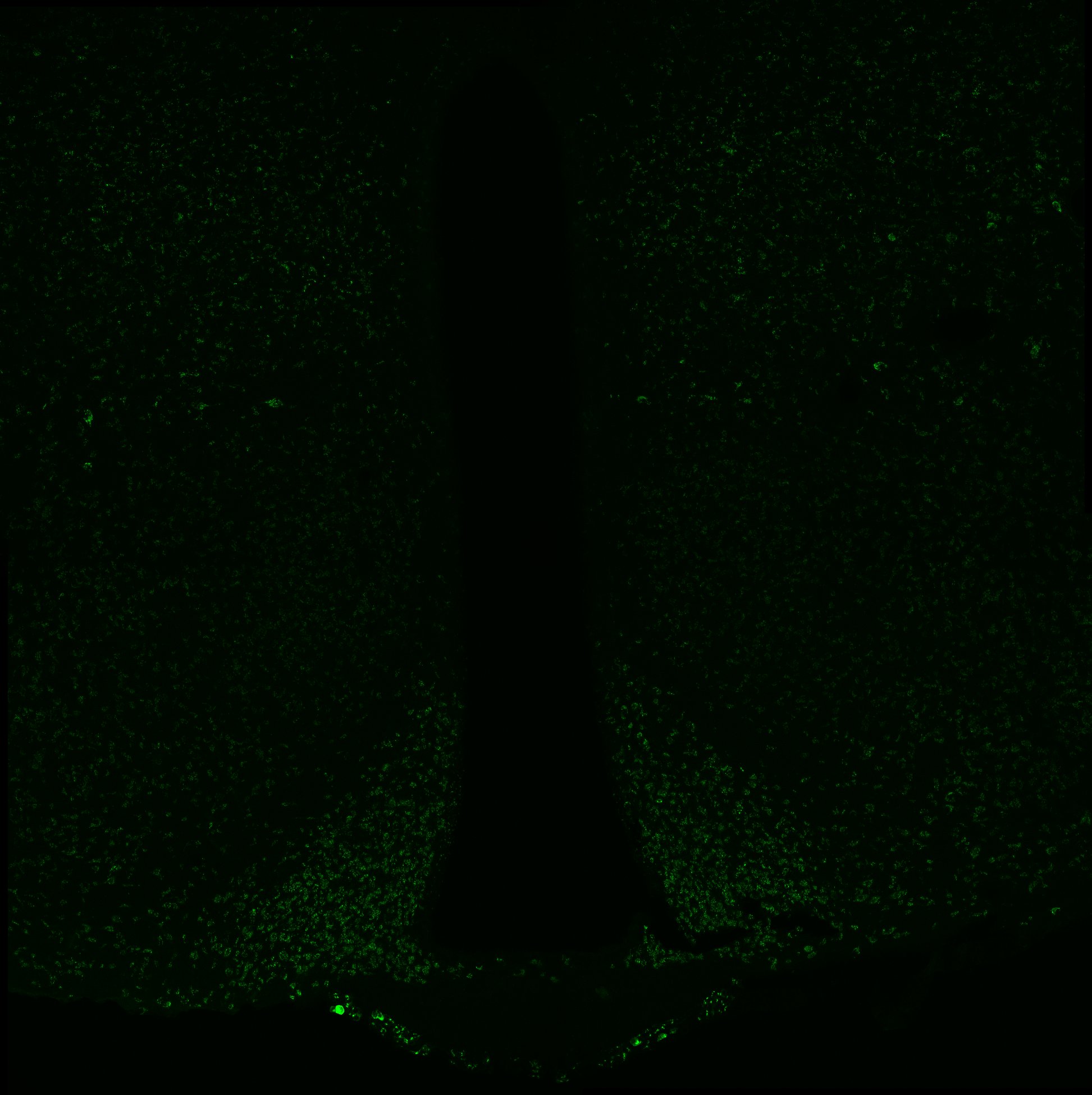

Supplement: Supplementary file 12 — Original data for Fig. 2a–d. [file 42255_2024_991_MOESM12_ESM.zip › Figure 2B/Mouse 27/1821-2 MidARH1.jpg]
