# Supplementary figures and images for: Global, neuronal or β cell-specific deletion of inceptor improves glucose homeostasis in male mice with diet-induced obesity (part 2 of 2)
Source: Nat Metab. 2024 Feb 28;6(3):448–57. doi: 10.1038/s42255-024-00991-3 (PMC10963260; doi:10.1038/s42255-024-00991-3)

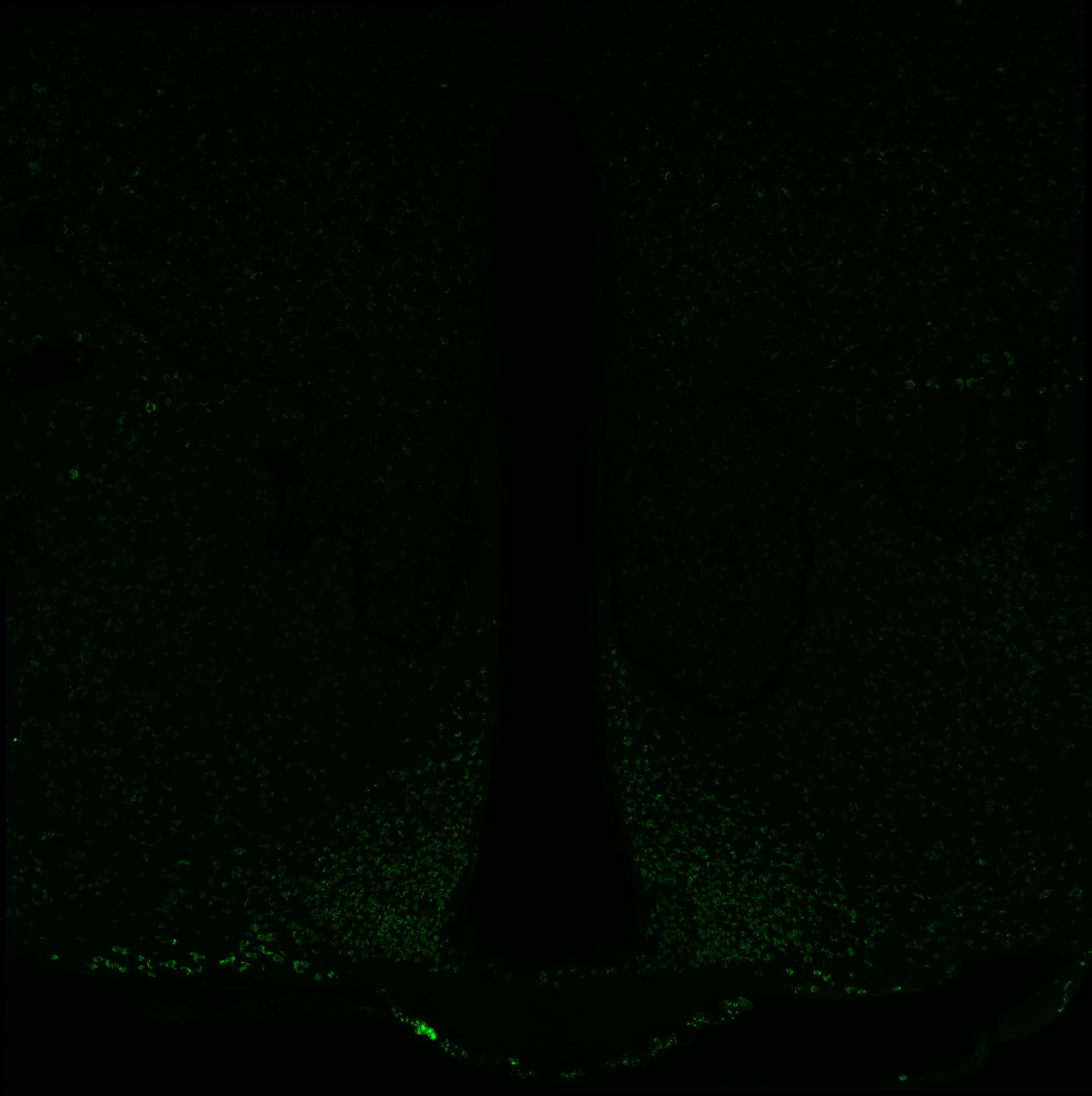

Supplement: Supplementary file 12 — Original data for Fig. 2a–d. [file 42255_2024_991_MOESM12_ESM.zip › Figure 2B/Mouse 27/1821-2 MidARH2.jpg]

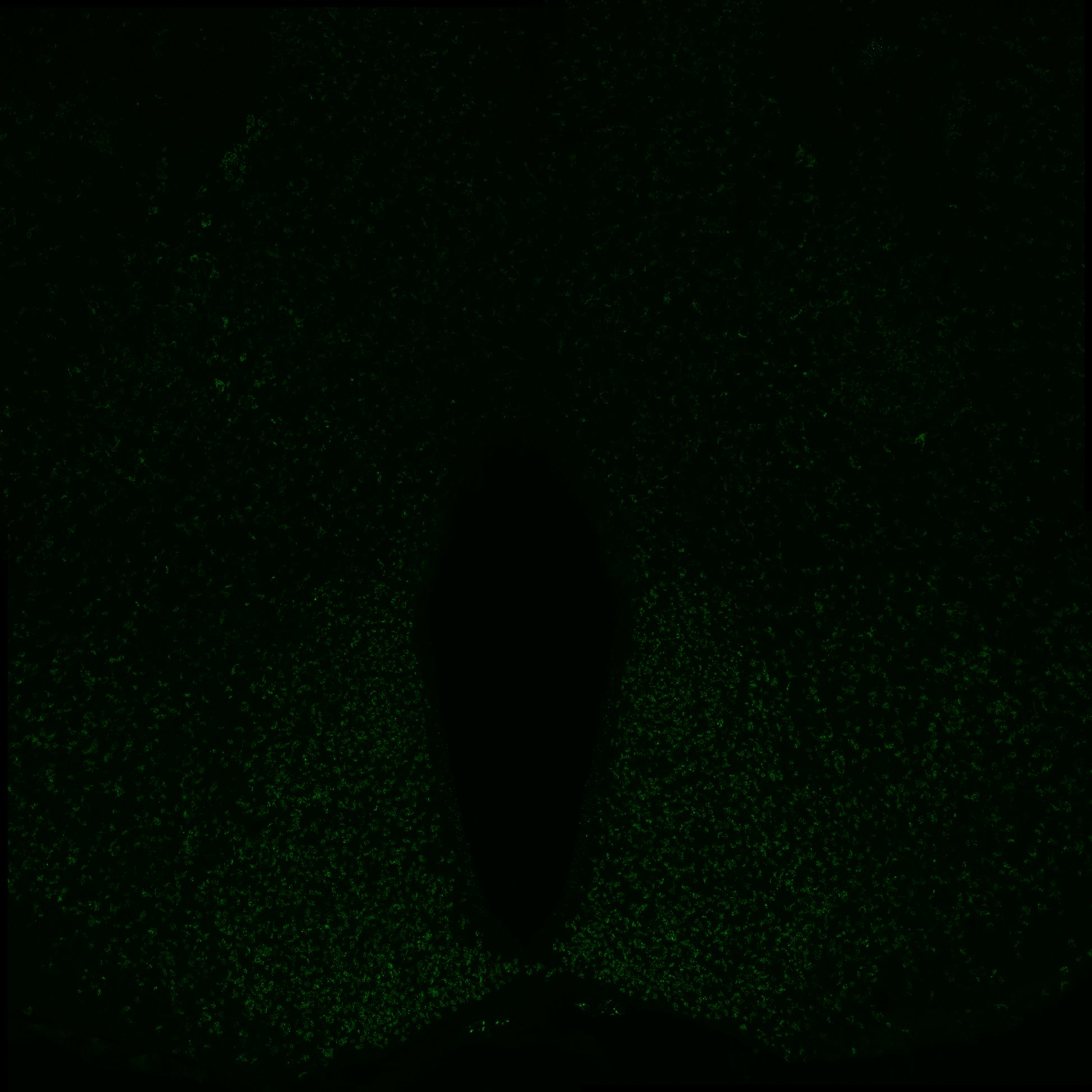

Supplement: Supplementary file 12 — Original data for Fig. 2a–d. [file 42255_2024_991_MOESM12_ESM.zip › Figure 2B/Mouse 27/1821-2 PostARH.jpg]

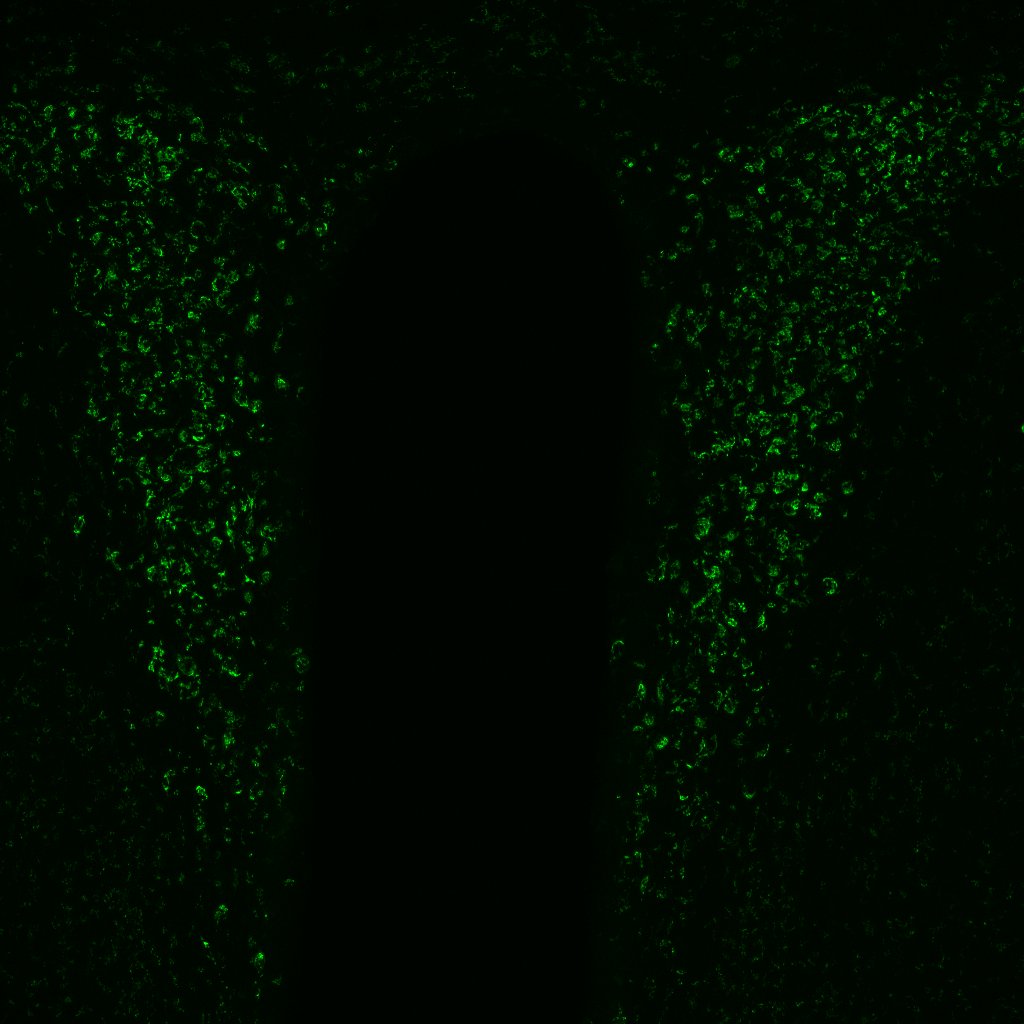

Supplement: Supplementary file 12 — Original data for Fig. 2a–d. [file 42255_2024_991_MOESM12_ESM.zip › Figure 2B/Mouse 27/1821-2 PVH1.jpg]

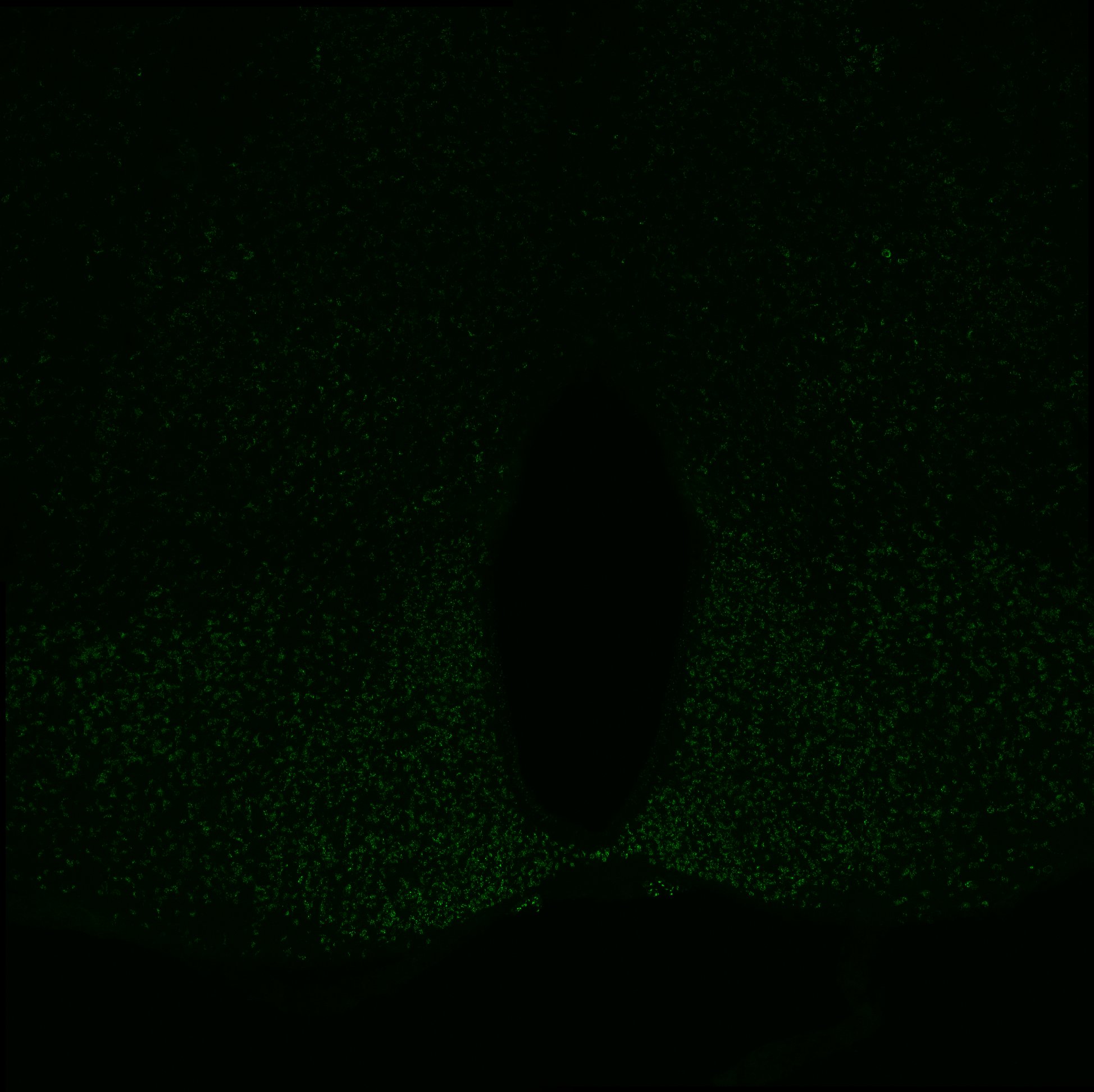

Supplement: Supplementary file 12 — Original data for Fig. 2a–d. [file 42255_2024_991_MOESM12_ESM.zip › Figure 2B/Mouse 18/1818-3 PostARH.jpg]

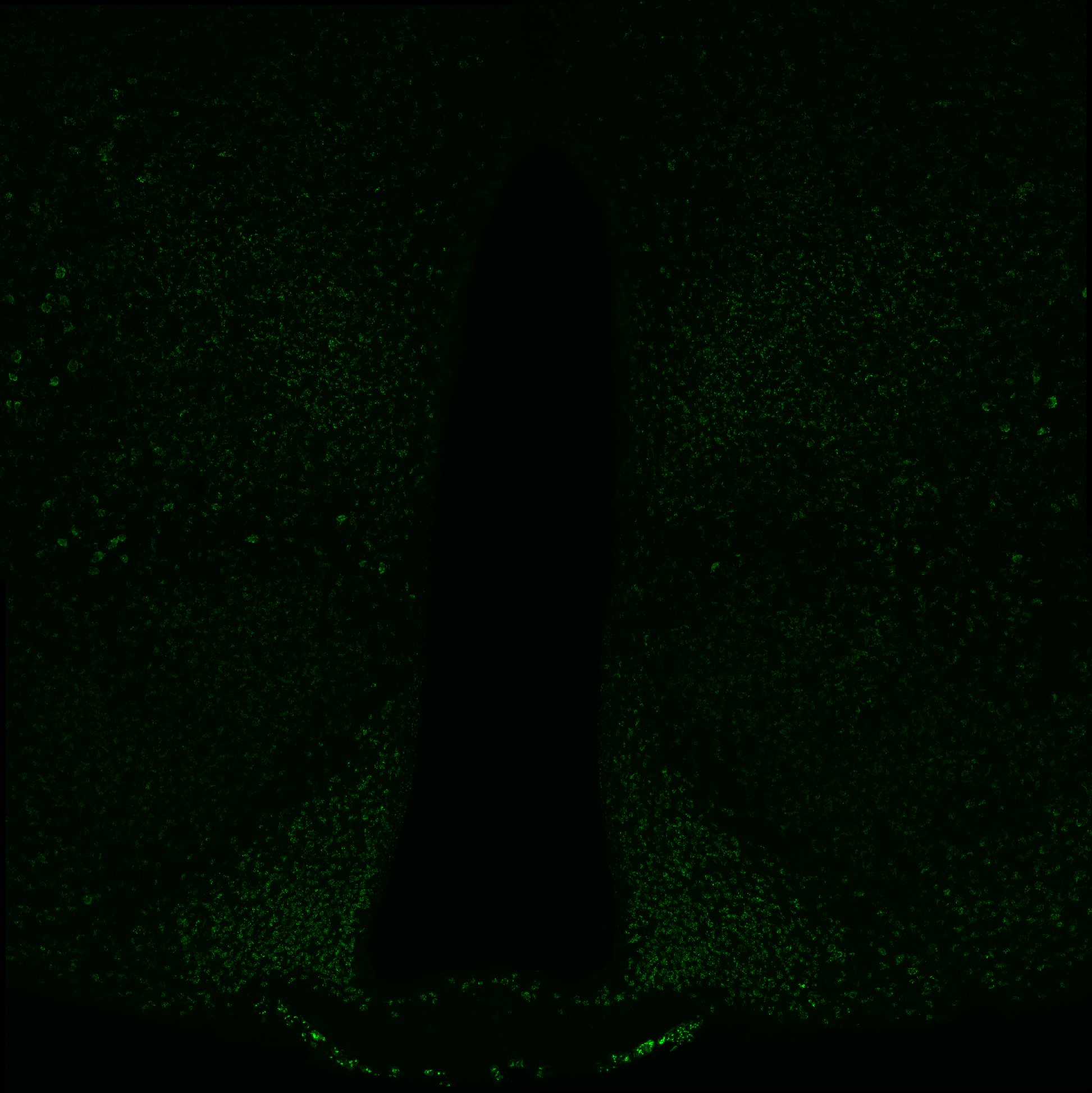

Supplement: Supplementary file 12 — Original data for Fig. 2a–d. [file 42255_2024_991_MOESM12_ESM.zip › Figure 2B/Mouse 18/1818-3 MidARH2.jpg]

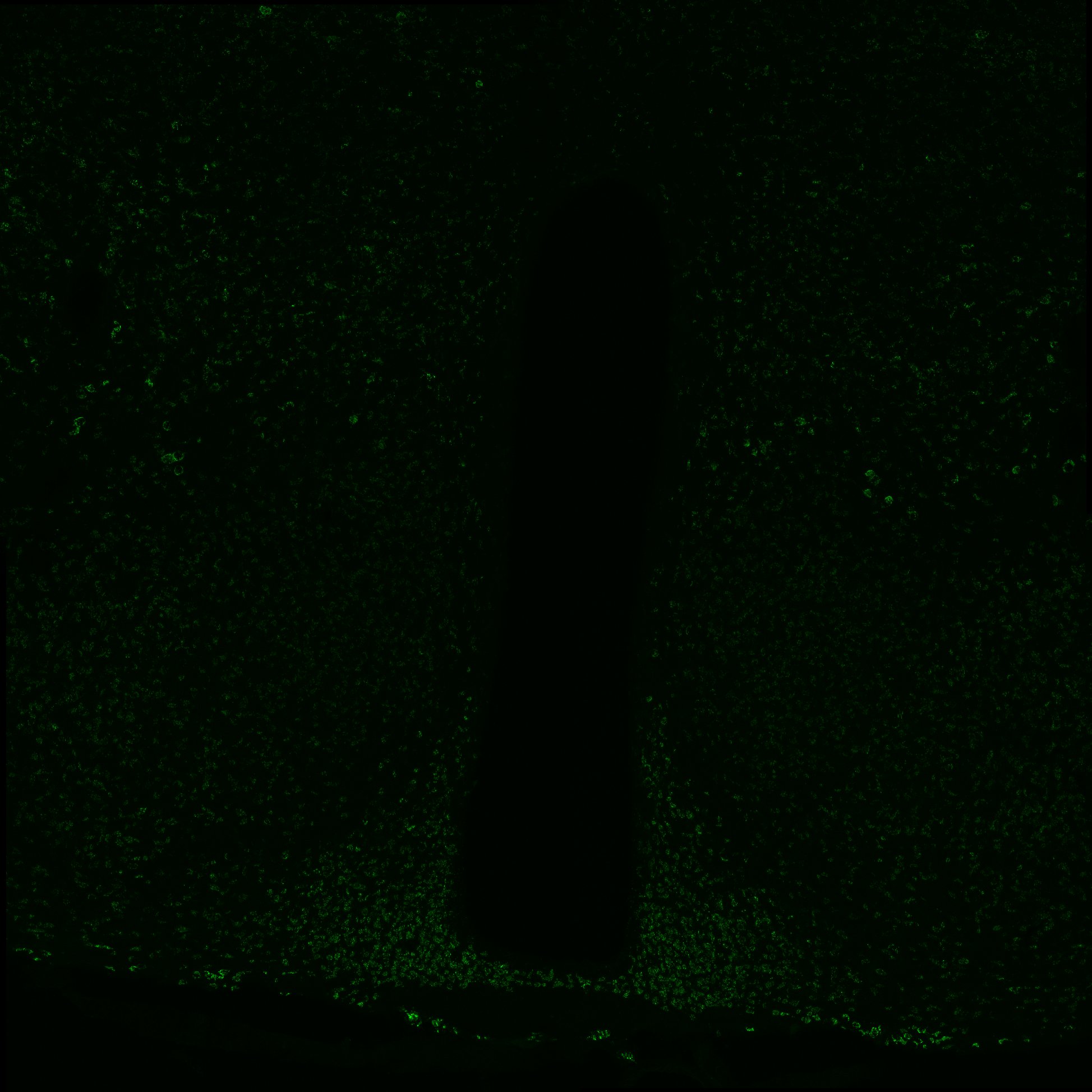

Supplement: Supplementary file 12 — Original data for Fig. 2a–d. [file 42255_2024_991_MOESM12_ESM.zip › Figure 2B/Mouse 18/1818-3 MidARH3.jpg]

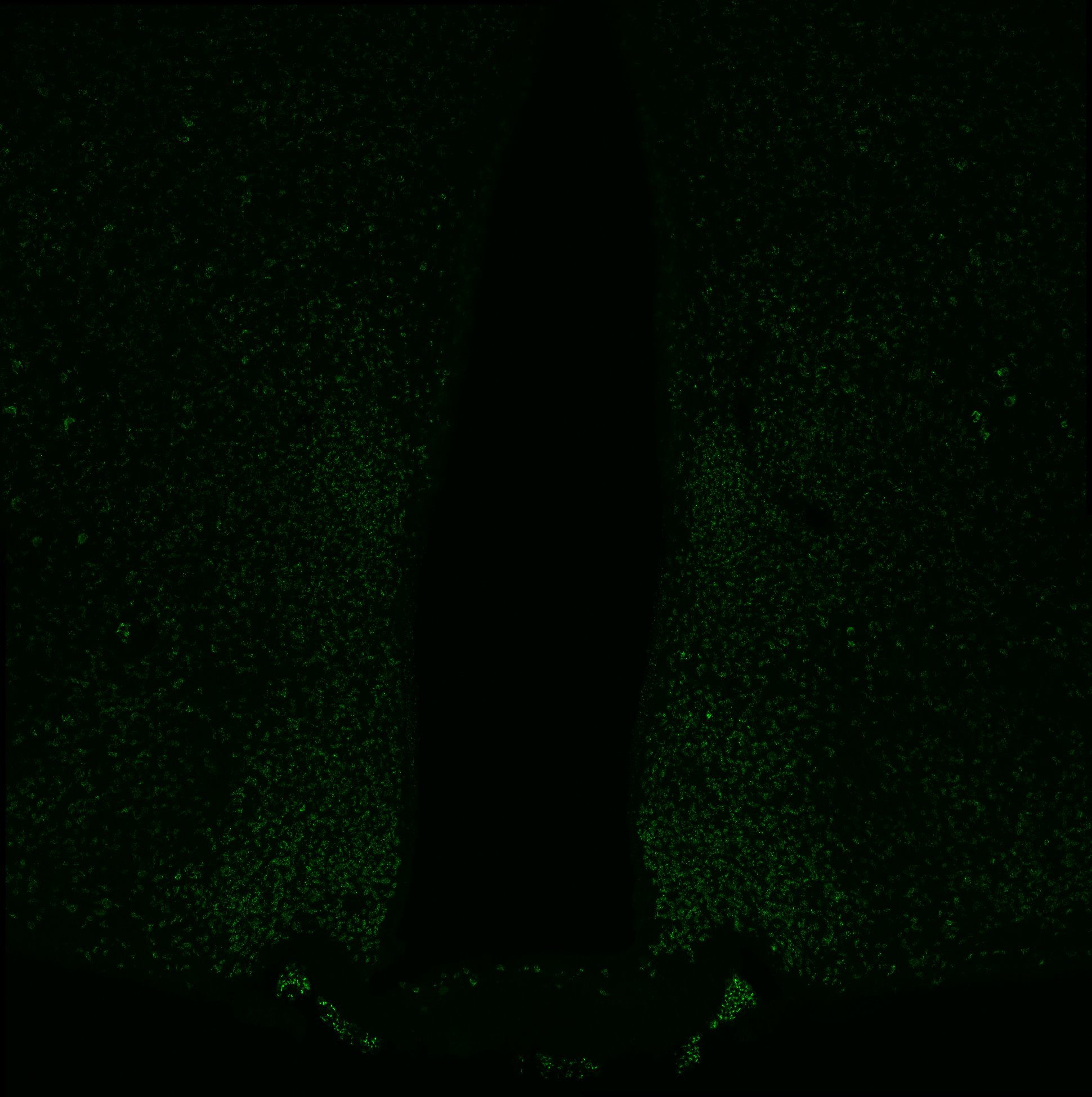

Supplement: Supplementary file 12 — Original data for Fig. 2a–d. [file 42255_2024_991_MOESM12_ESM.zip › Figure 2B/Mouse 18/1818-3 MidARH1.jpg]

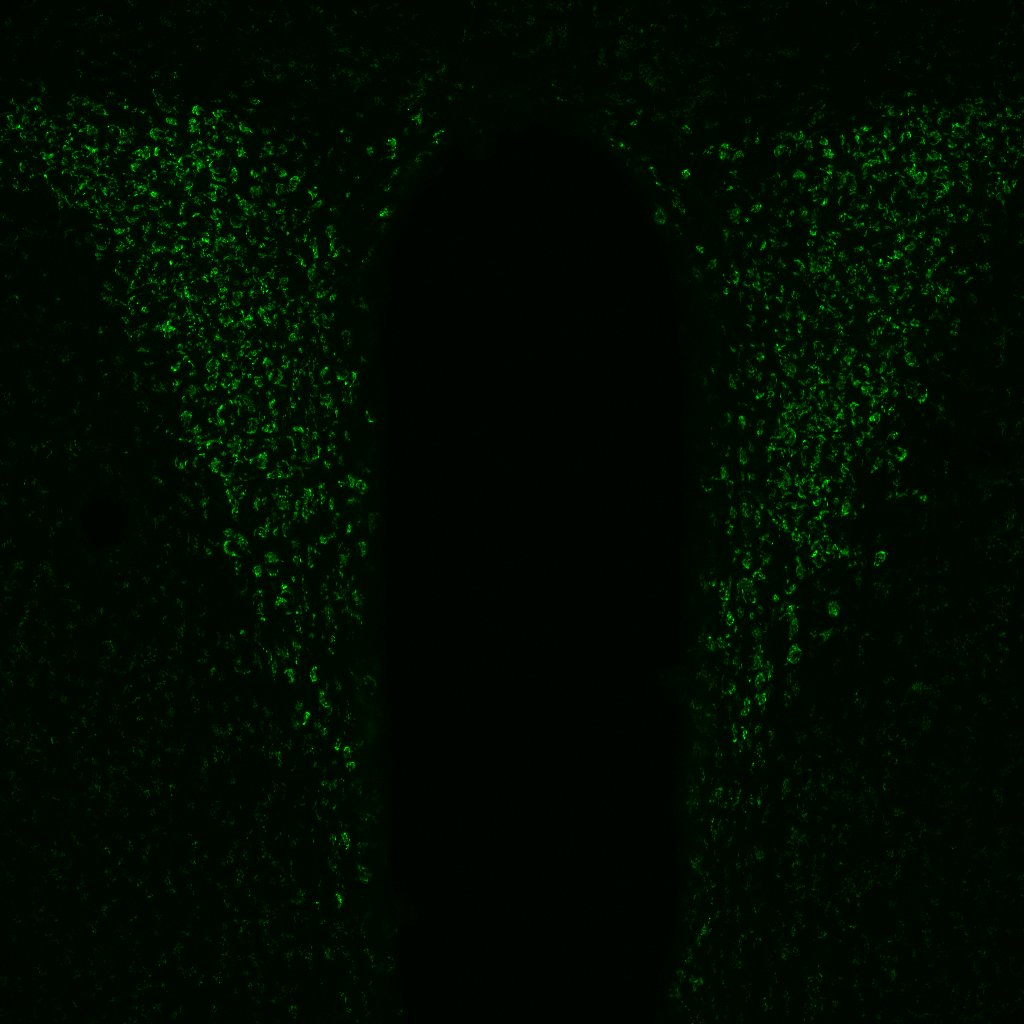

Supplement: Supplementary file 12 — Original data for Fig. 2a–d. [file 42255_2024_991_MOESM12_ESM.zip › Figure 2B/Mouse 18/1818-3 PVH.jpg]

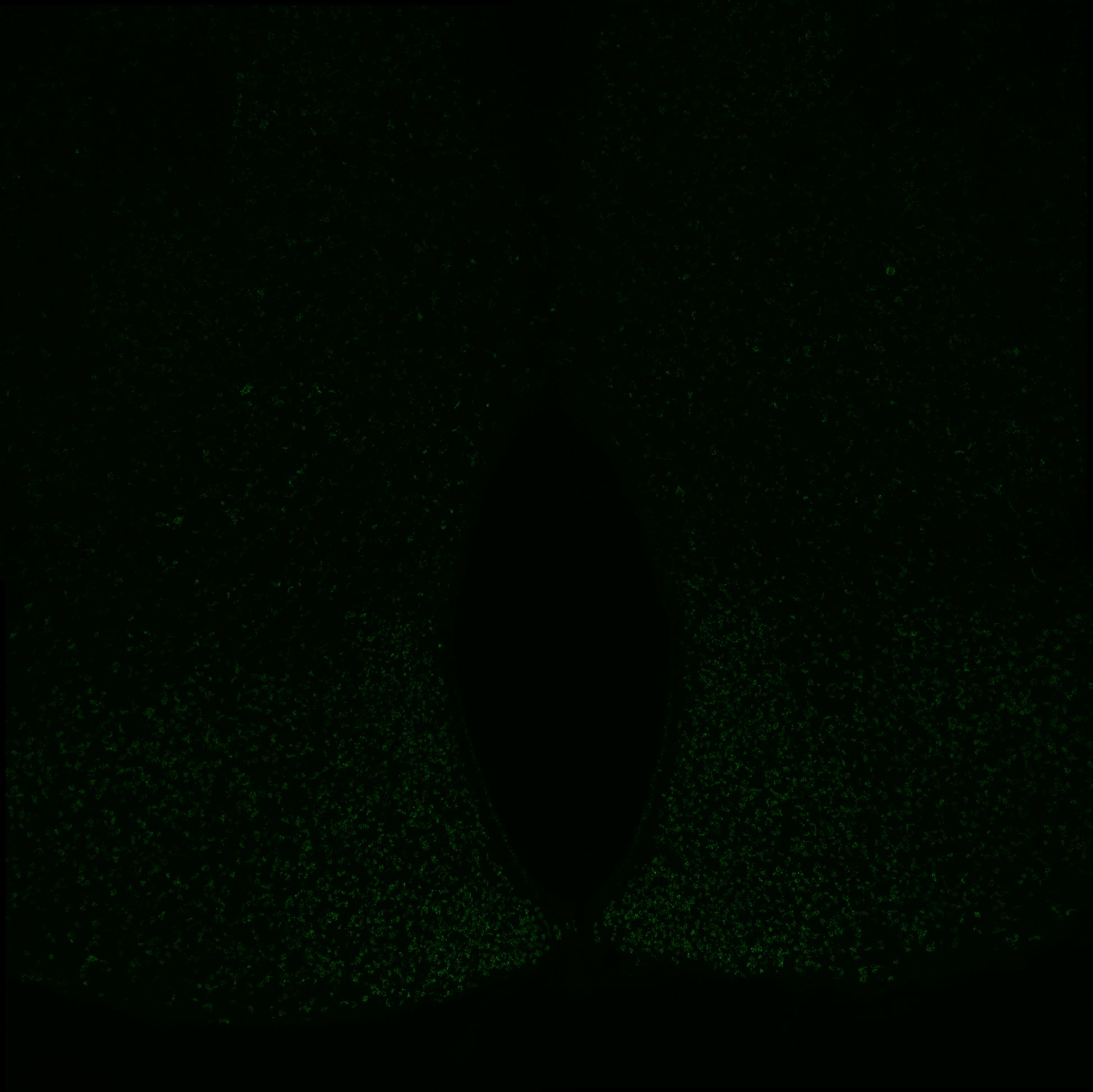

Supplement: Supplementary file 12 — Original data for Fig. 2a–d. [file 42255_2024_991_MOESM12_ESM.zip › Figure 2B/Mouse 7/1845-3 PostARH.jpg]

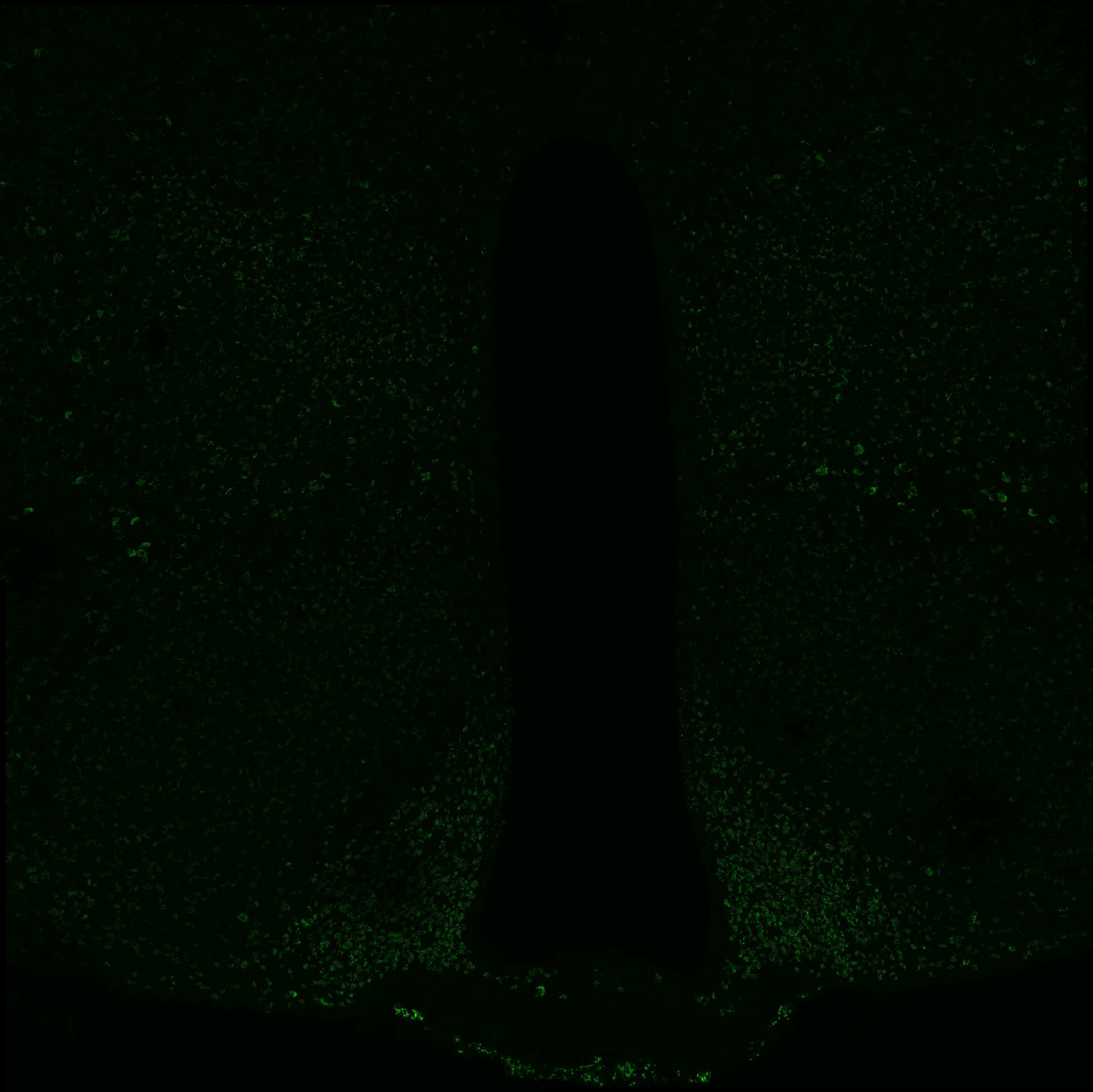

Supplement: Supplementary file 12 — Original data for Fig. 2a–d. [file 42255_2024_991_MOESM12_ESM.zip › Figure 2B/Mouse 7/1845-3 MidARH2.jpg]

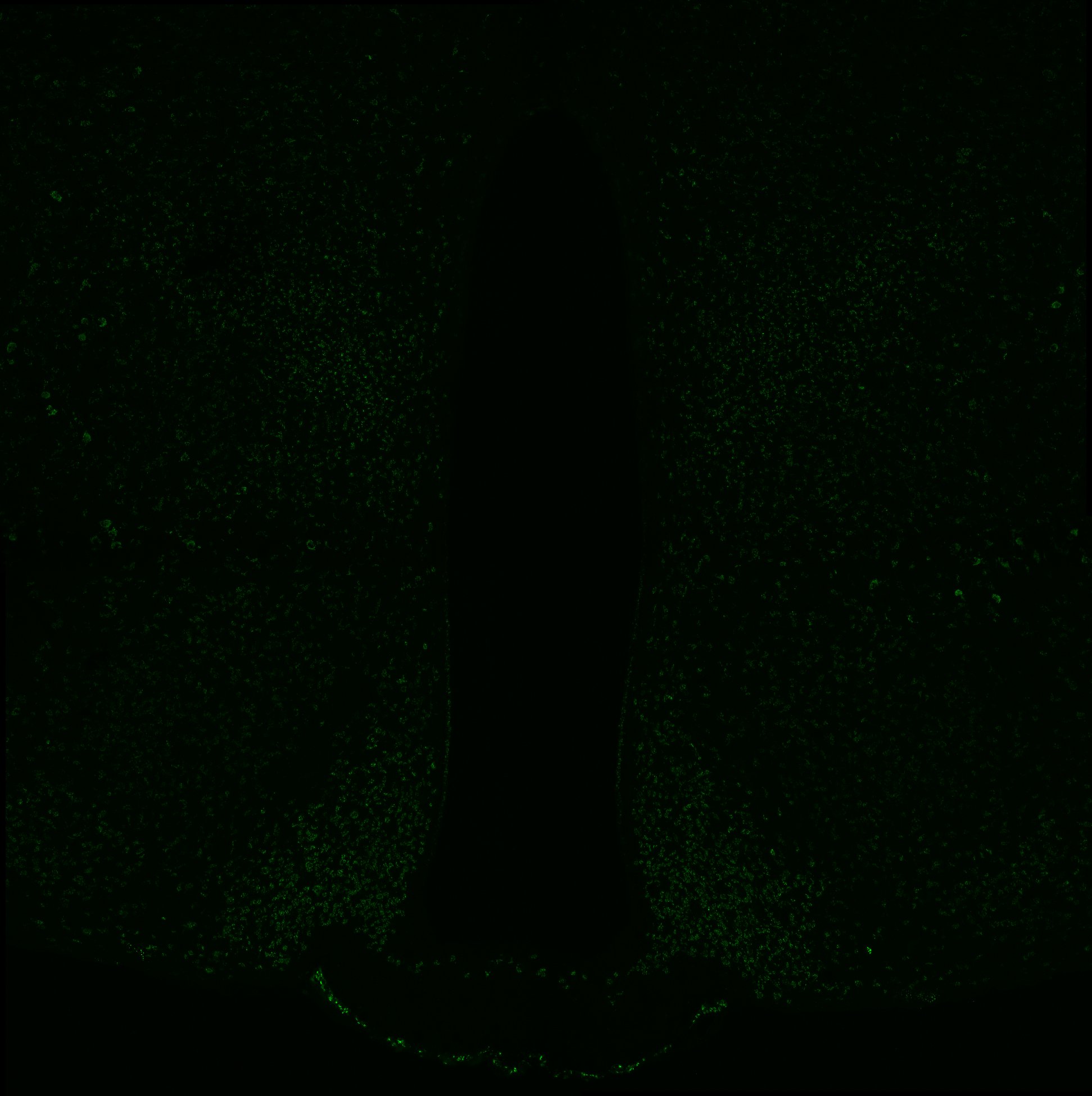

Supplement: Supplementary file 12 — Original data for Fig. 2a–d. [file 42255_2024_991_MOESM12_ESM.zip › Figure 2B/Mouse 7/1845-3 MidARH3.jpg]

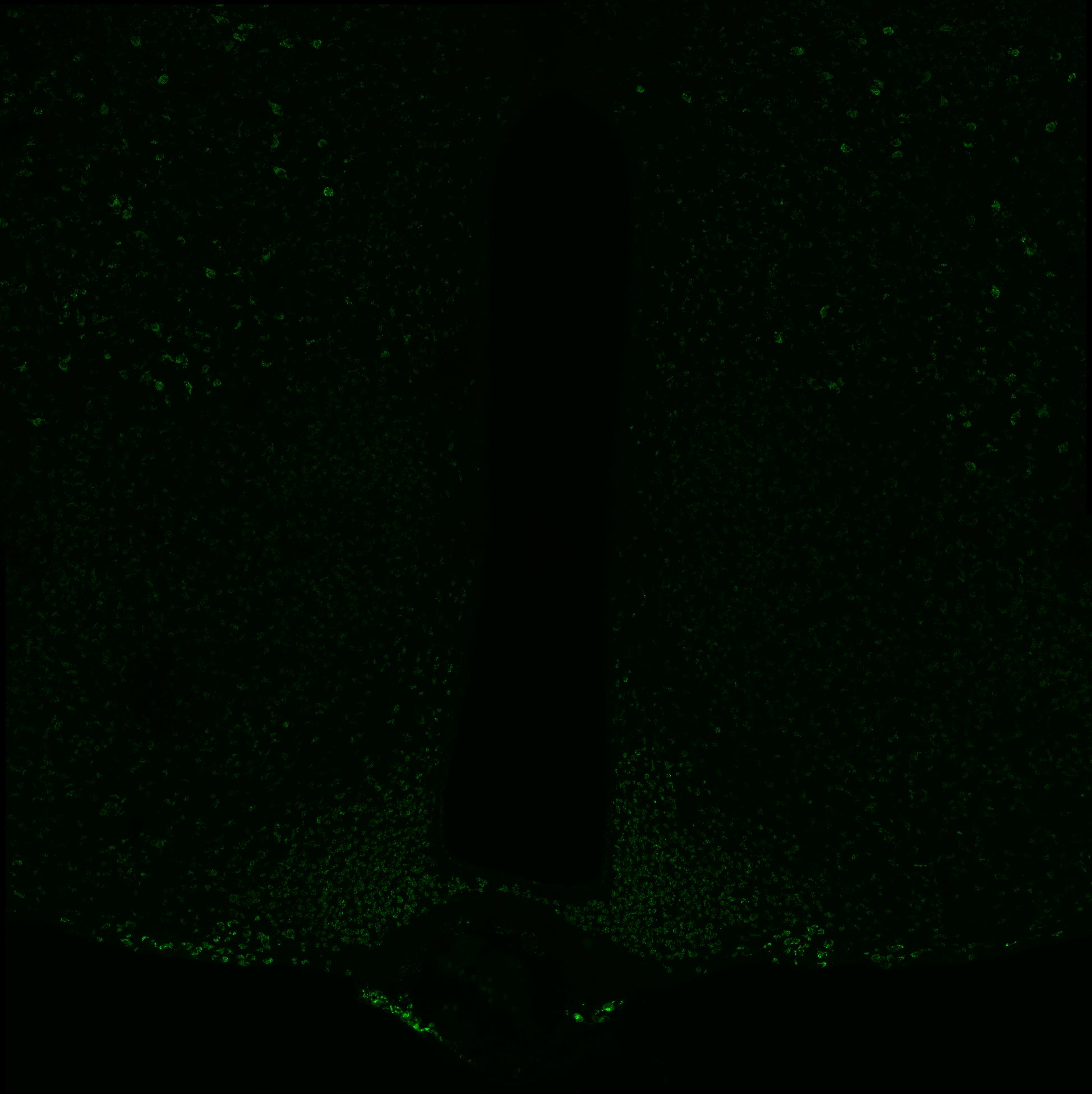

Supplement: Supplementary file 12 — Original data for Fig. 2a–d. [file 42255_2024_991_MOESM12_ESM.zip › Figure 2B/Mouse 7/1845-3 MidARH1.jpg]

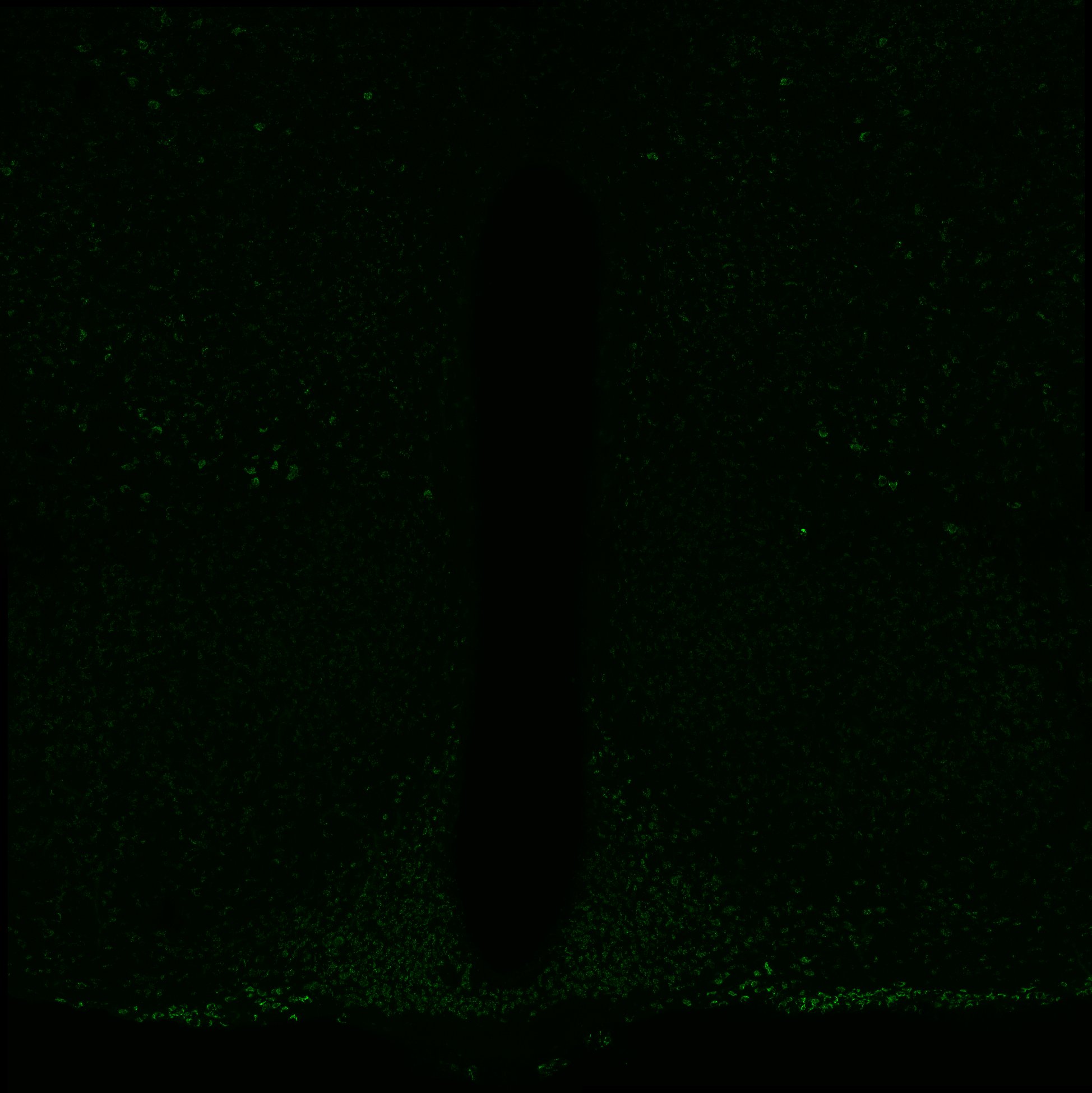

Supplement: Supplementary file 12 — Original data for Fig. 2a–d. [file 42255_2024_991_MOESM12_ESM.zip › Figure 2B/Mouse 9/1845-4 MidARH1.jpg]

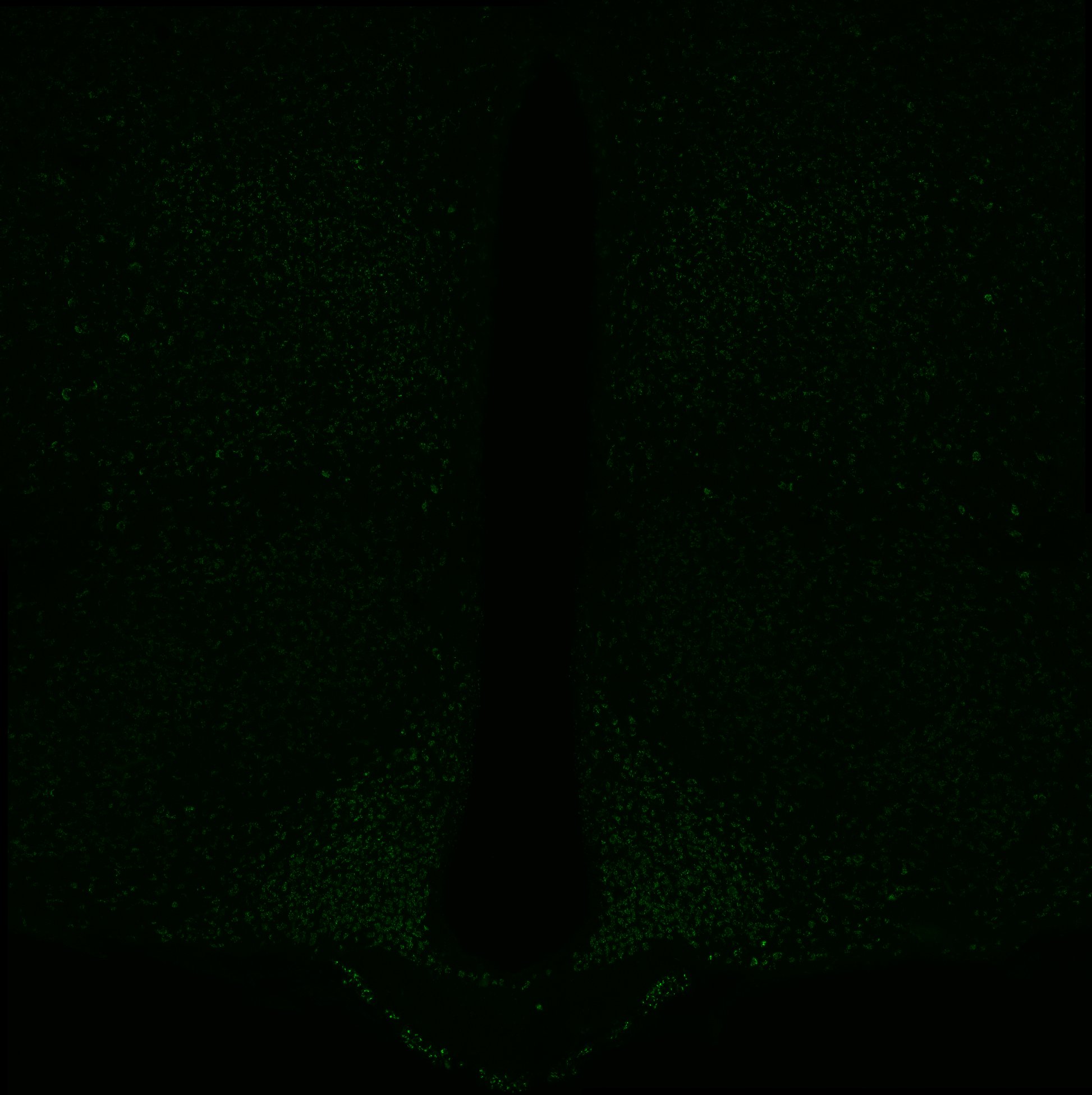

Supplement: Supplementary file 12 — Original data for Fig. 2a–d. [file 42255_2024_991_MOESM12_ESM.zip › Figure 2B/Mouse 9/1845-4 MidARH2.jpg]

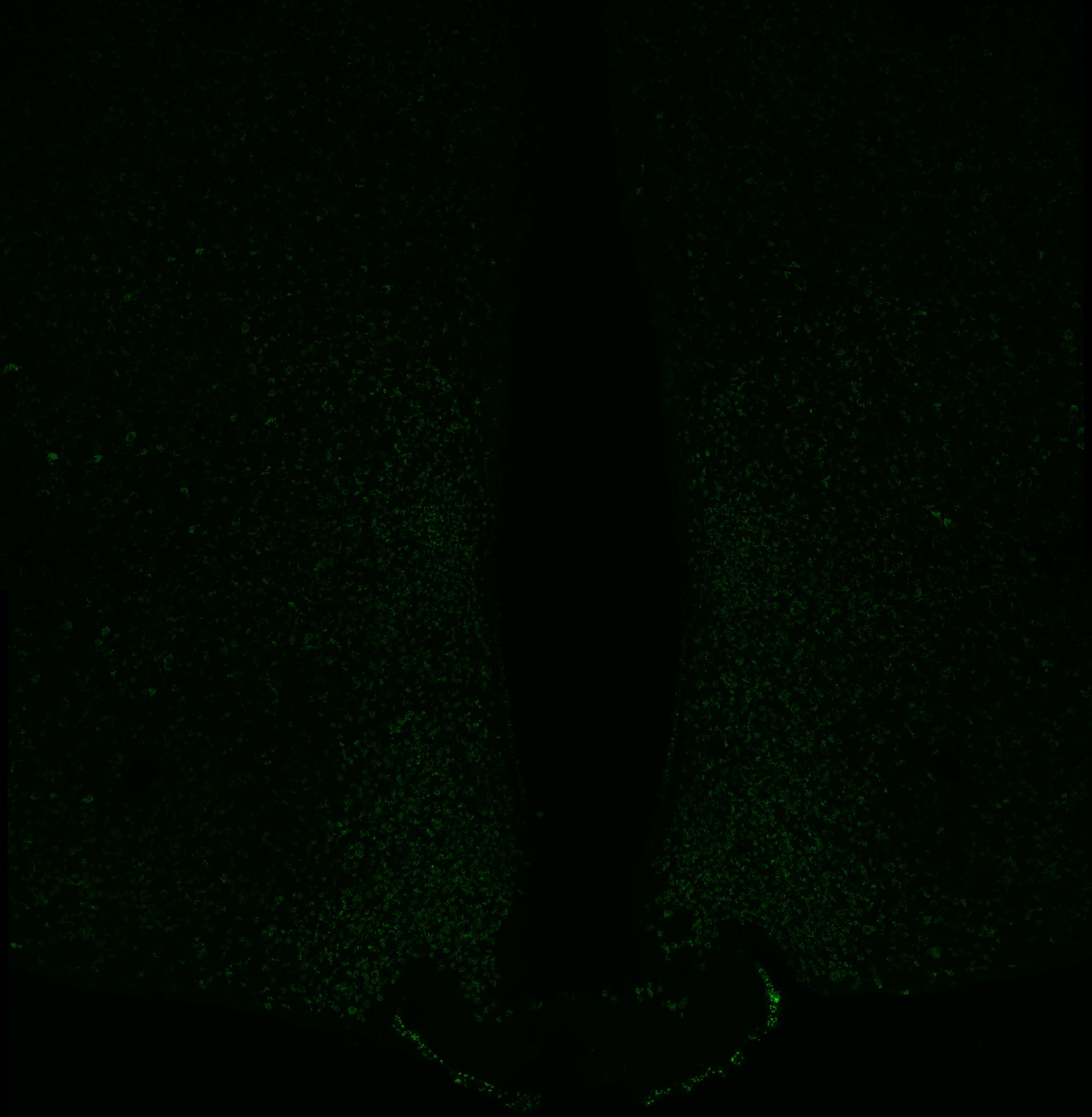

Supplement: Supplementary file 12 — Original data for Fig. 2a–d. [file 42255_2024_991_MOESM12_ESM.zip › Figure 2B/Mouse 9/1845-4 MidARH3.jpg]

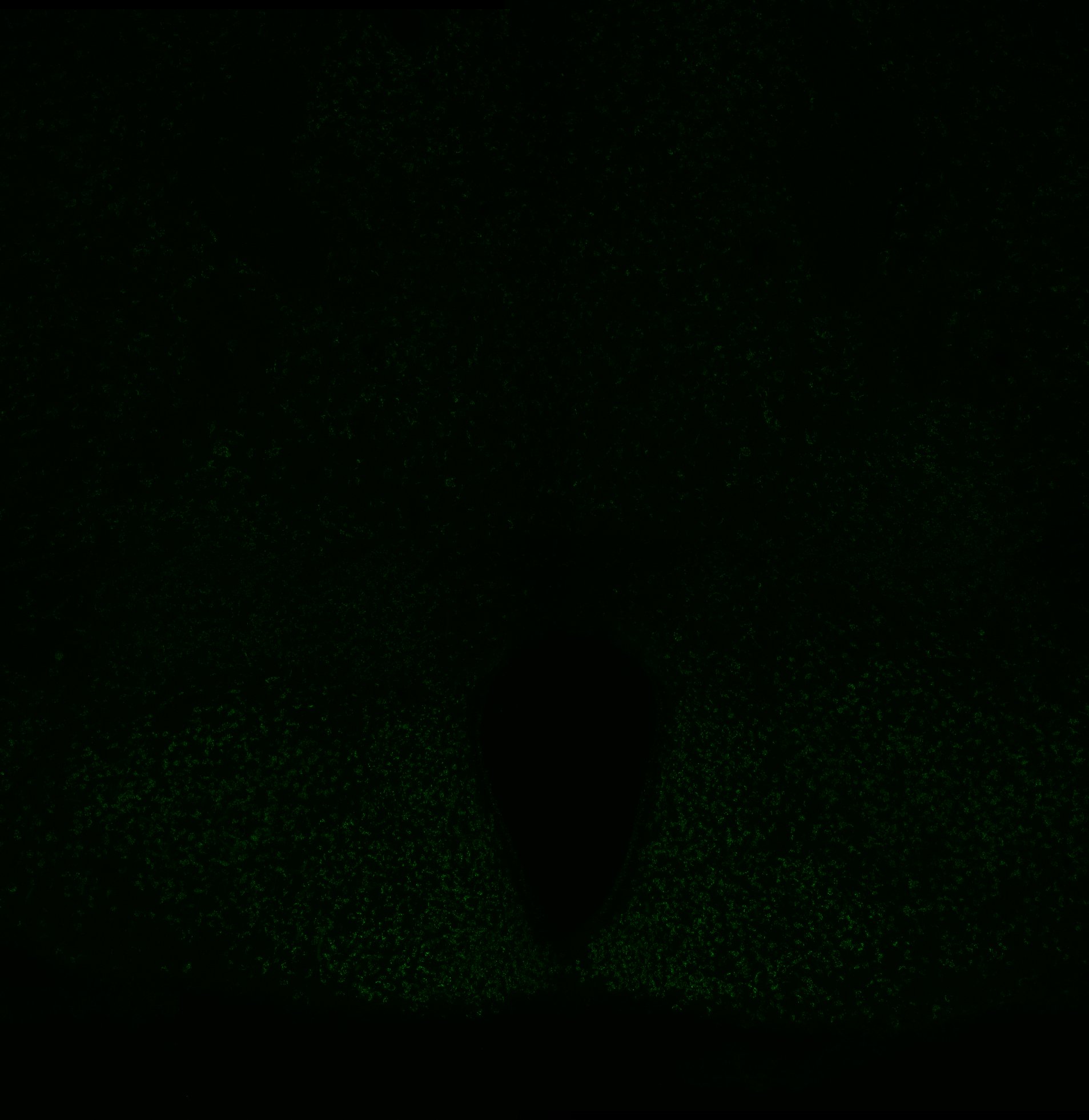

Supplement: Supplementary file 12 — Original data for Fig. 2a–d. [file 42255_2024_991_MOESM12_ESM.zip › Figure 2B/Mouse 9/1845-4 PostARH.jpg]

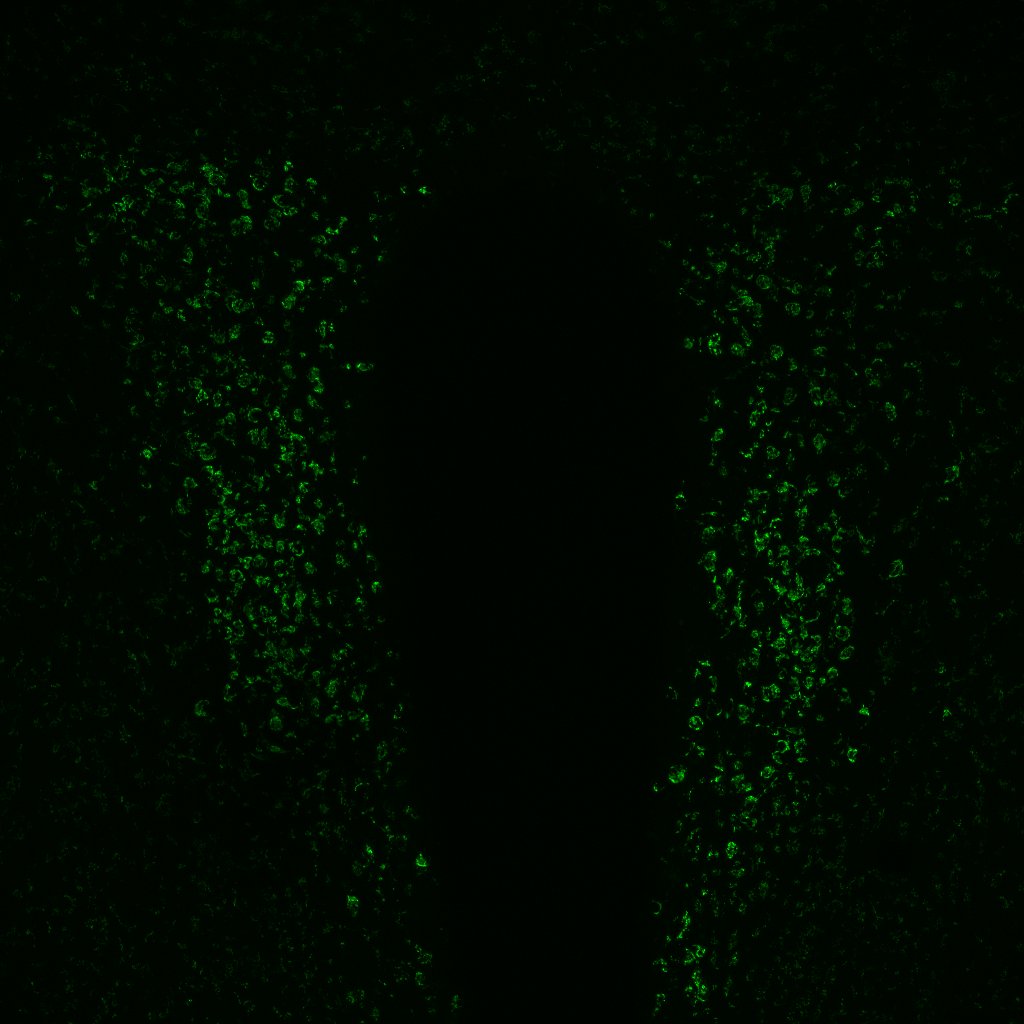

Supplement: Supplementary file 12 — Original data for Fig. 2a–d. [file 42255_2024_991_MOESM12_ESM.zip › Figure 2B/Mouse 8/1845-2 PVH1.jpg]

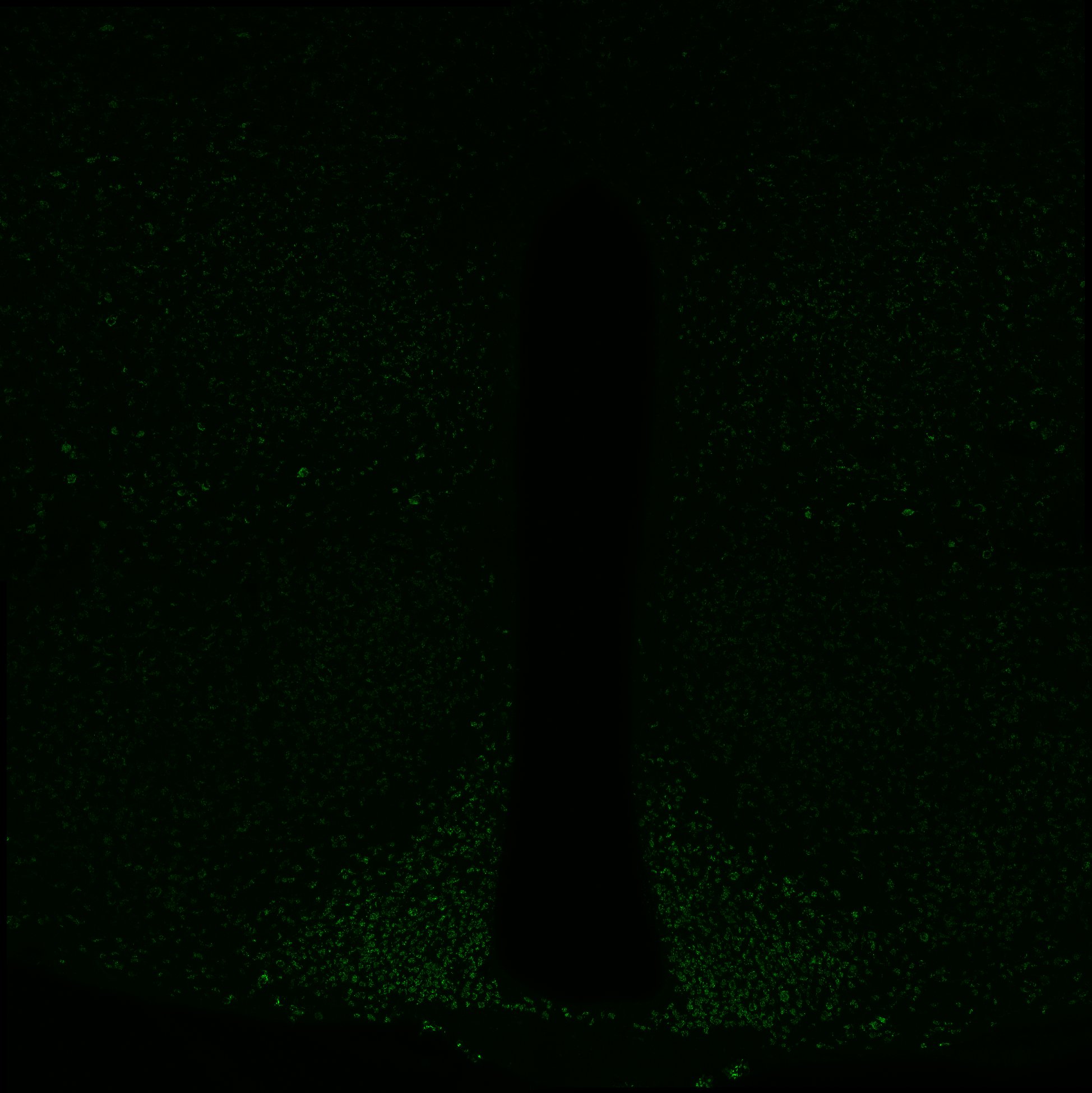

Supplement: Supplementary file 12 — Original data for Fig. 2a–d. [file 42255_2024_991_MOESM12_ESM.zip › Figure 2B/Mouse 8/1845-2 MidARH1.jpg]

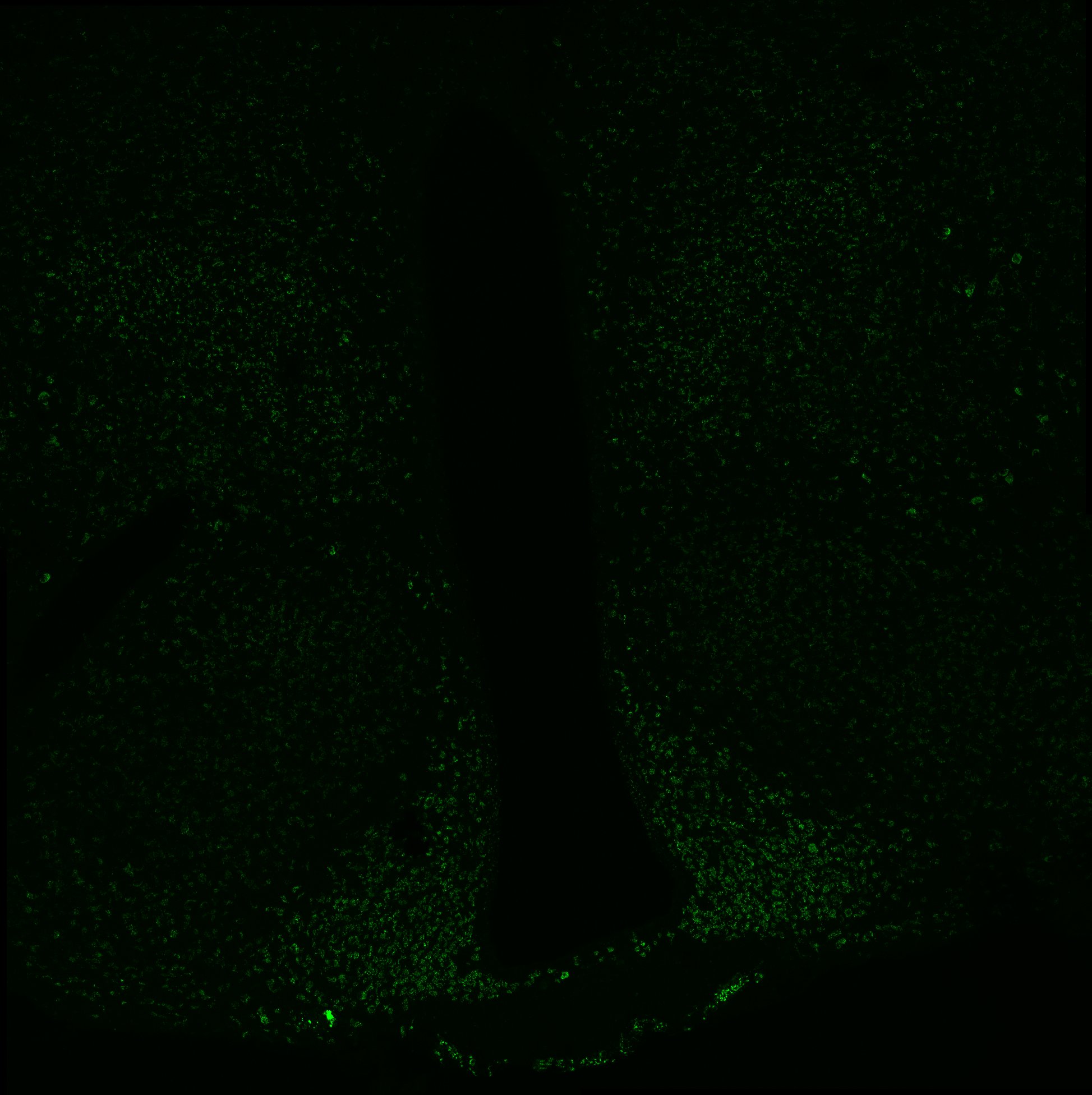

Supplement: Supplementary file 12 — Original data for Fig. 2a–d. [file 42255_2024_991_MOESM12_ESM.zip › Figure 2B/Mouse 8/1845-2 MidARH2.jpg]

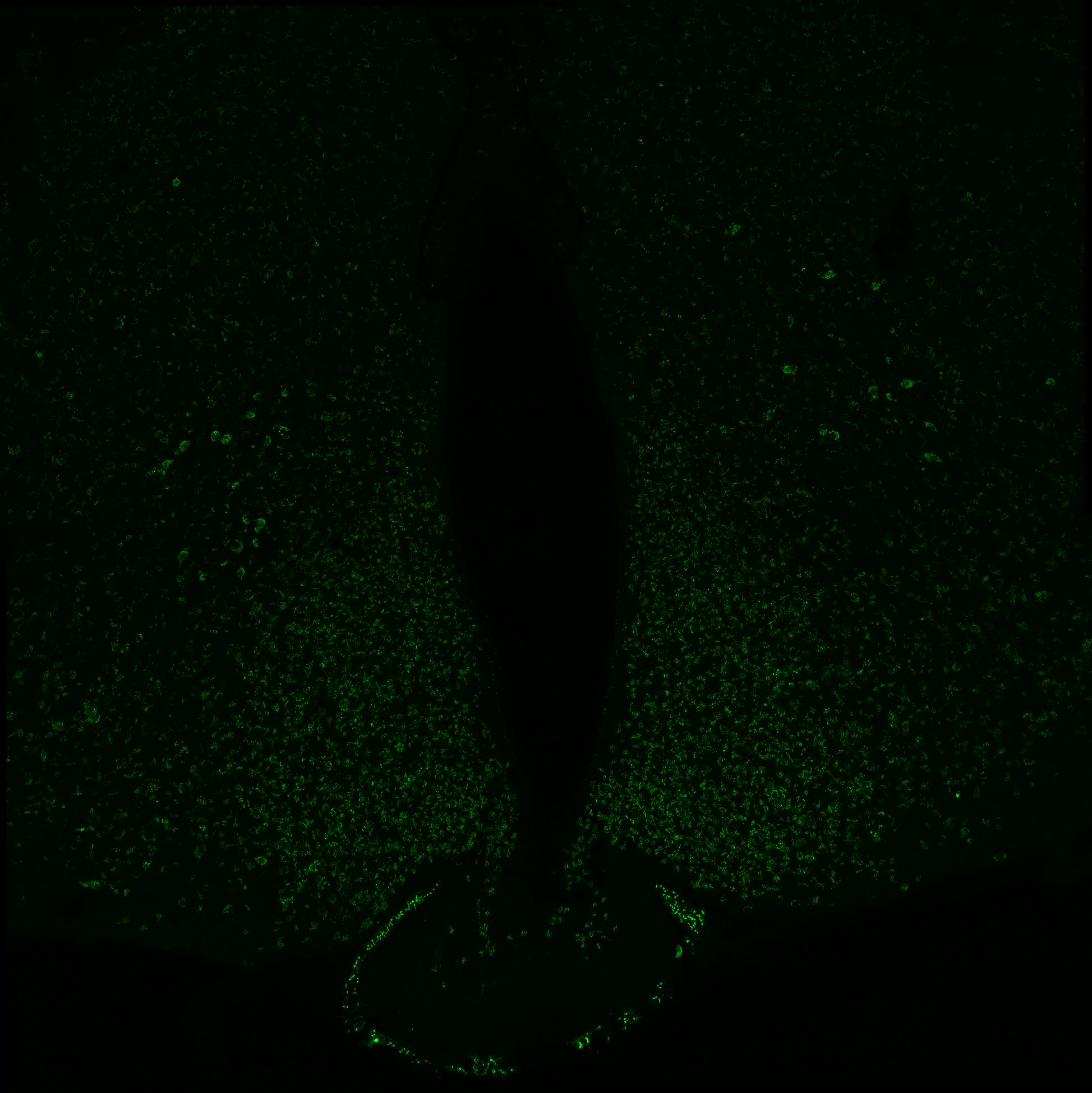

Supplement: Supplementary file 12 — Original data for Fig. 2a–d. [file 42255_2024_991_MOESM12_ESM.zip › Figure 2B/Mouse 8/1845-2 PostARH.jpg]

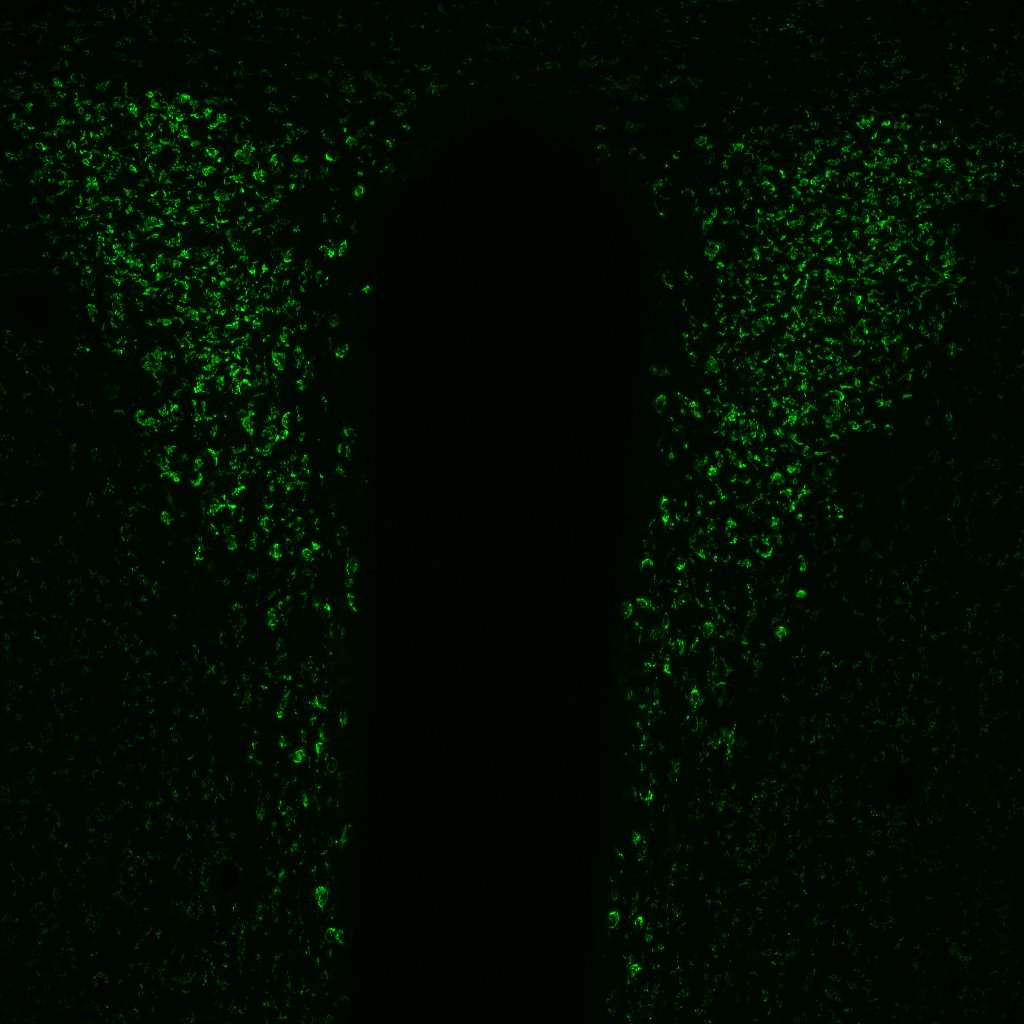

Supplement: Supplementary file 12 — Original data for Fig. 2a–d. [file 42255_2024_991_MOESM12_ESM.zip › Figure 2B/Mouse 1/1830-1 PVH1.jpg]

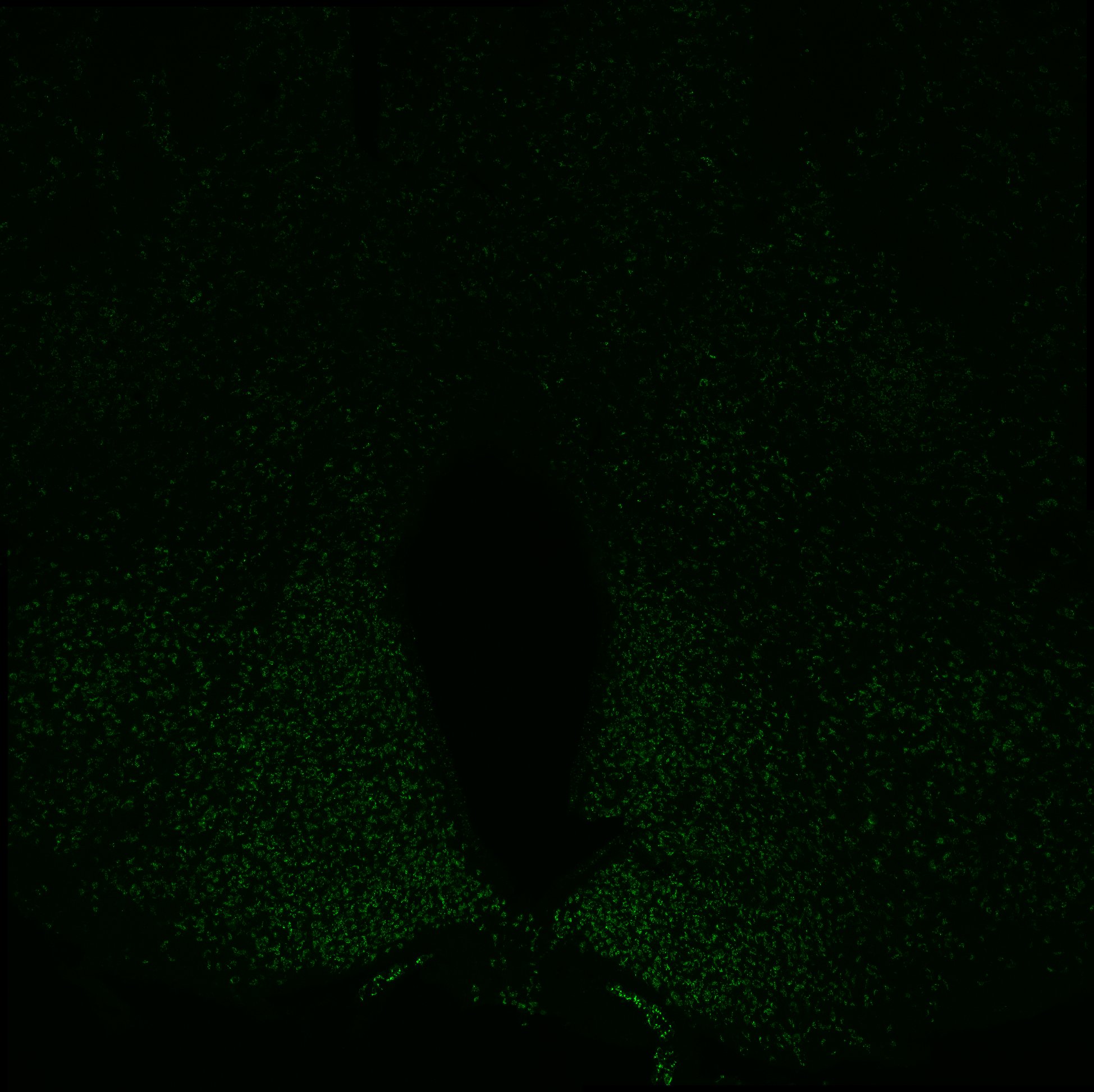

Supplement: Supplementary file 12 — Original data for Fig. 2a–d. [file 42255_2024_991_MOESM12_ESM.zip › Figure 2B/Mouse 1/1830-1 PostARH1.jpg]

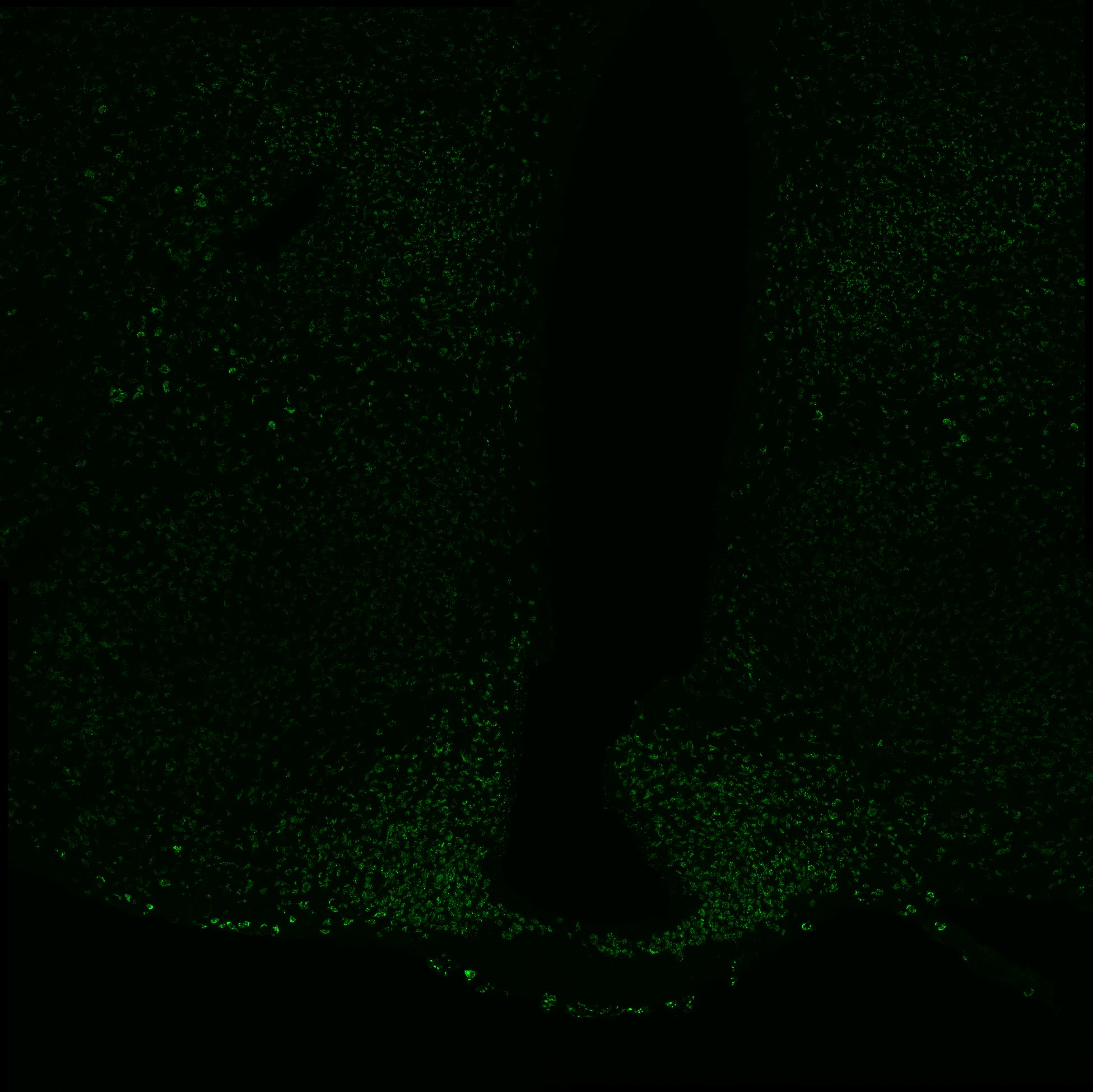

Supplement: Supplementary file 12 — Original data for Fig. 2a–d. [file 42255_2024_991_MOESM12_ESM.zip › Figure 2B/Mouse 1/1830-1 MidARH1.jpg]

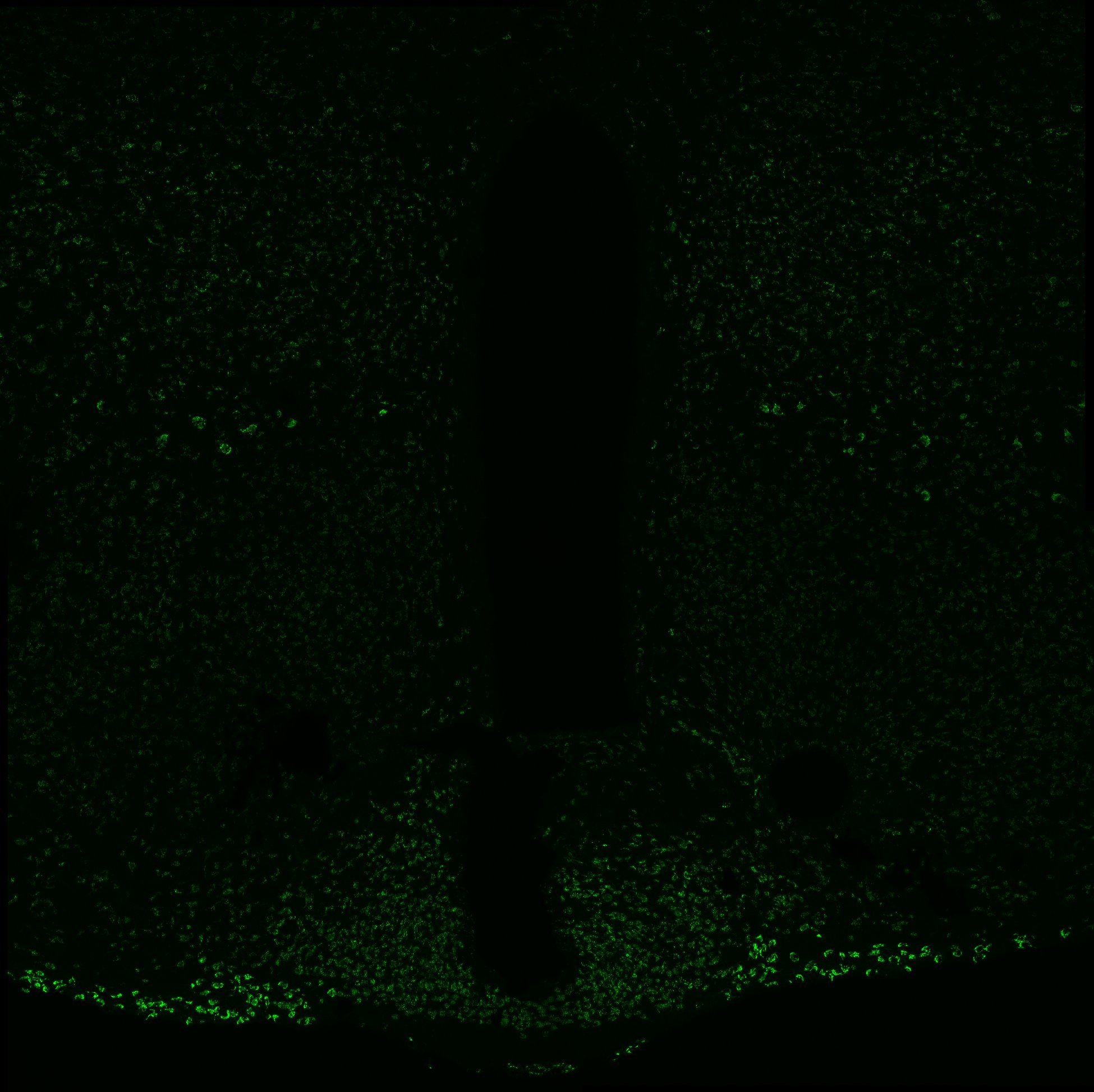

Supplement: Supplementary file 12 — Original data for Fig. 2a–d. [file 42255_2024_991_MOESM12_ESM.zip › Figure 2B/Mouse 1/1830-1 MidARH2.jpg]

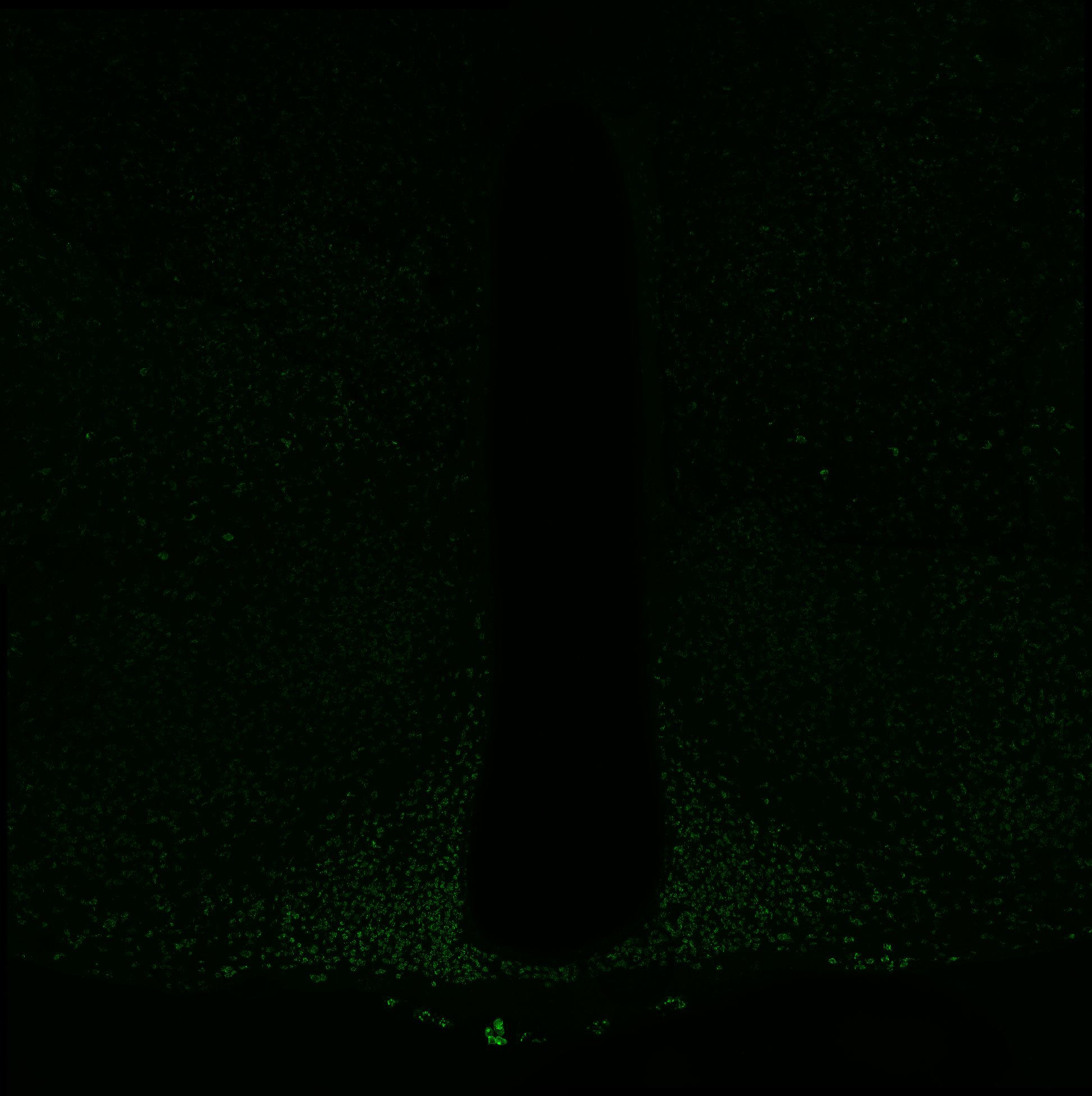

Supplement: Supplementary file 12 — Original data for Fig. 2a–d. [file 42255_2024_991_MOESM12_ESM.zip › Figure 2B/Mouse 6/1845-1 MidARH2.jpg]

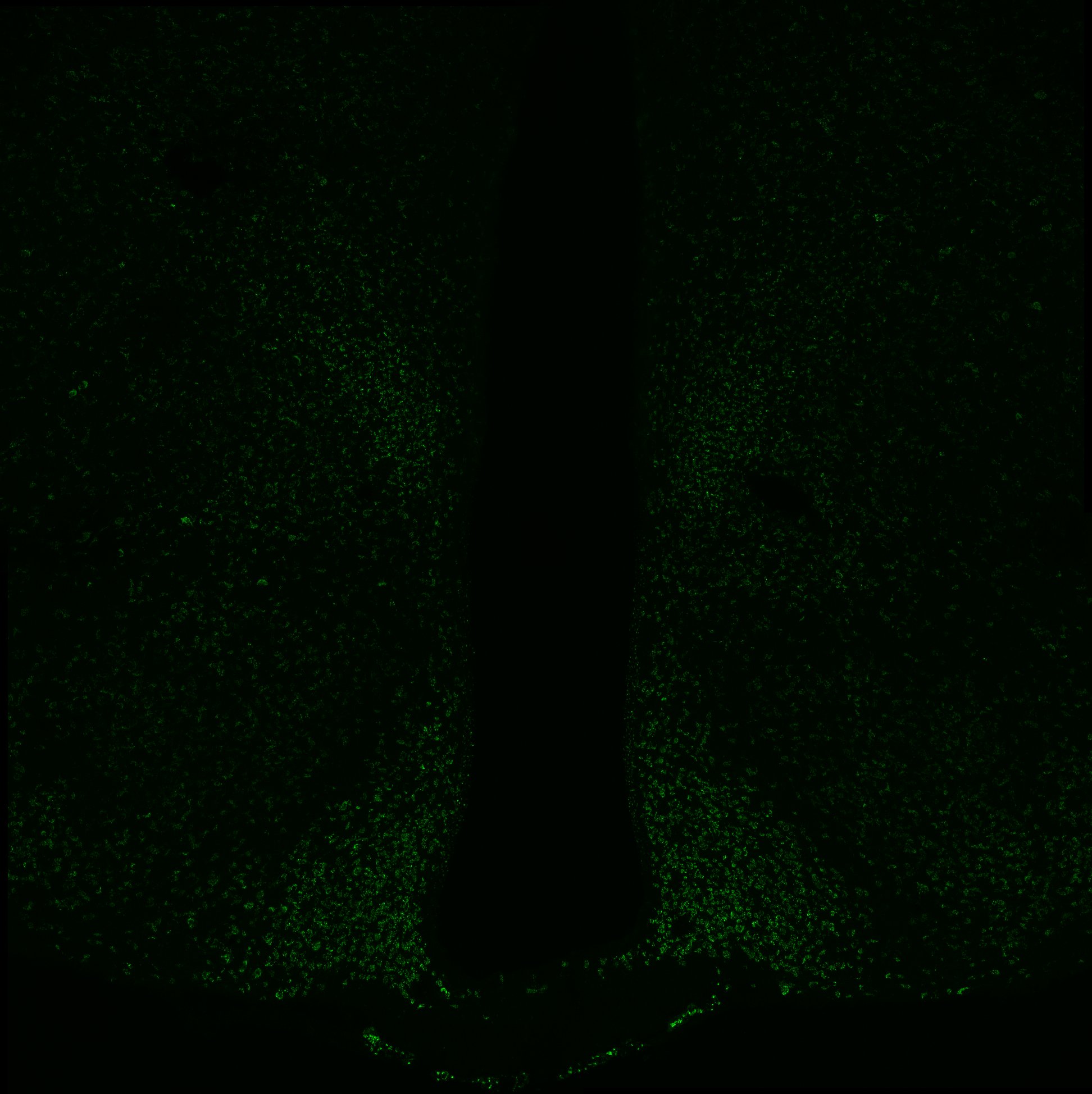

Supplement: Supplementary file 12 — Original data for Fig. 2a–d. [file 42255_2024_991_MOESM12_ESM.zip › Figure 2B/Mouse 6/1845-1 MidARH1.jpg]

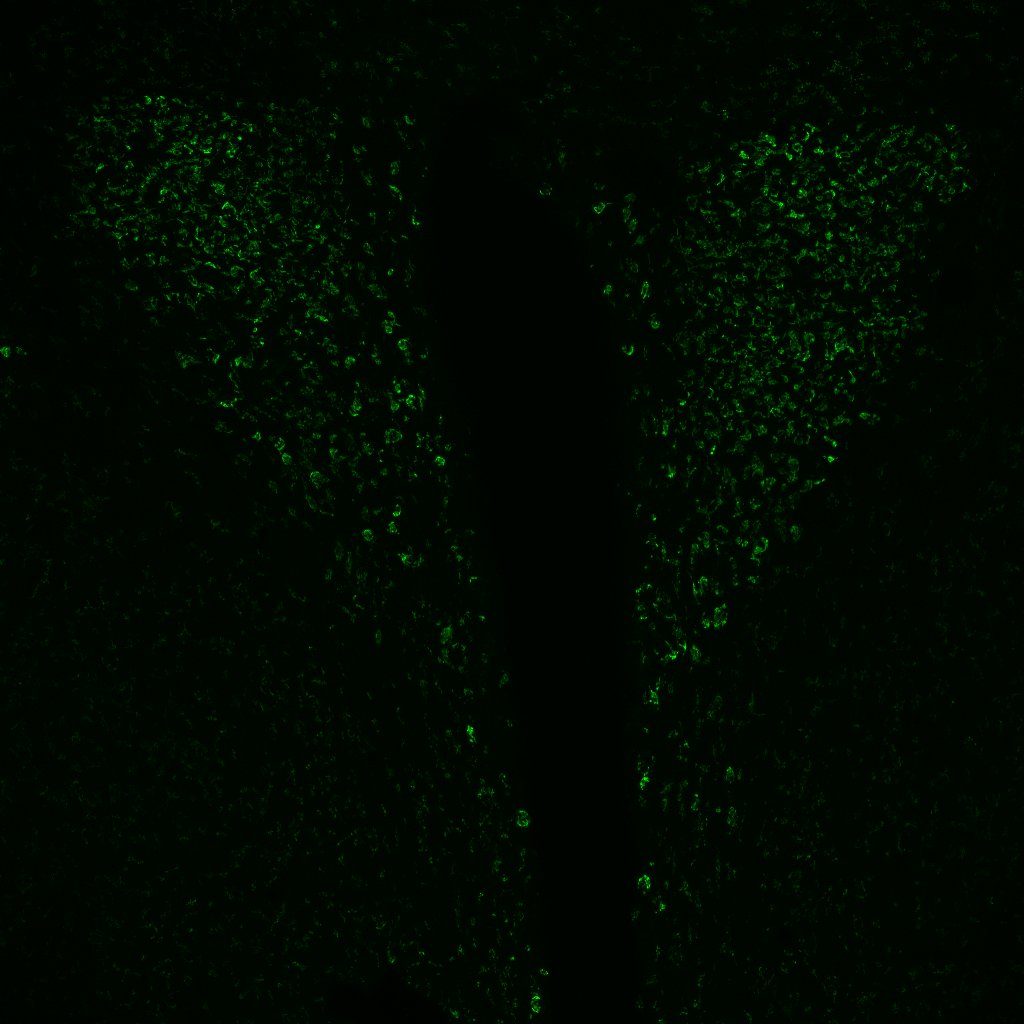

Supplement: Supplementary file 12 — Original data for Fig. 2a–d. [file 42255_2024_991_MOESM12_ESM.zip › Figure 2B/Mouse 6/1845-1 PVH2.jpg]

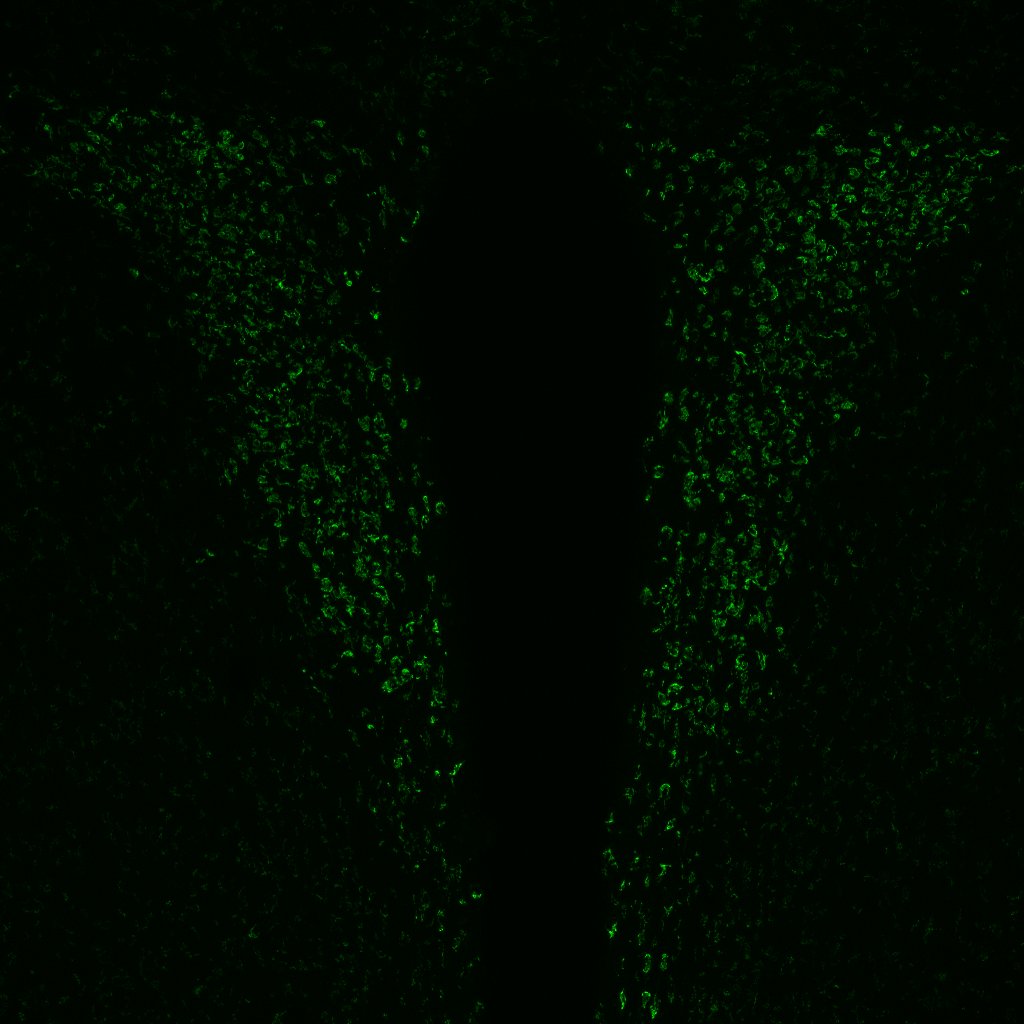

Supplement: Supplementary file 12 — Original data for Fig. 2a–d. [file 42255_2024_991_MOESM12_ESM.zip › Figure 2B/Mouse 6/1845-1 PVH1.jpg]

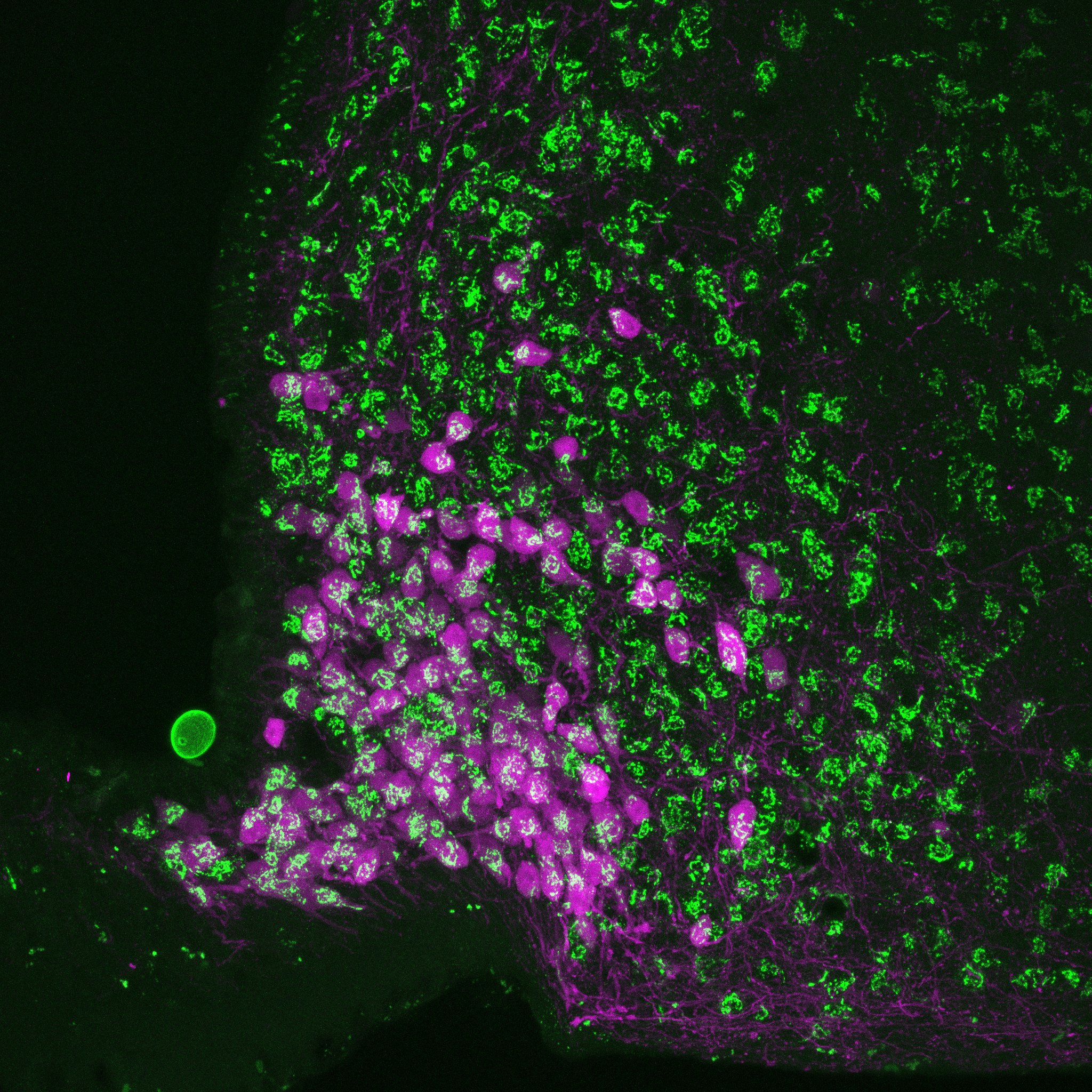

Supplement: Supplementary file 12 — Original data for Fig. 2a–d. [file 42255_2024_991_MOESM12_ESM.zip › Figure 2C/IGFRL-NPYGFP-COLOC-0-100nodapi.jpg]

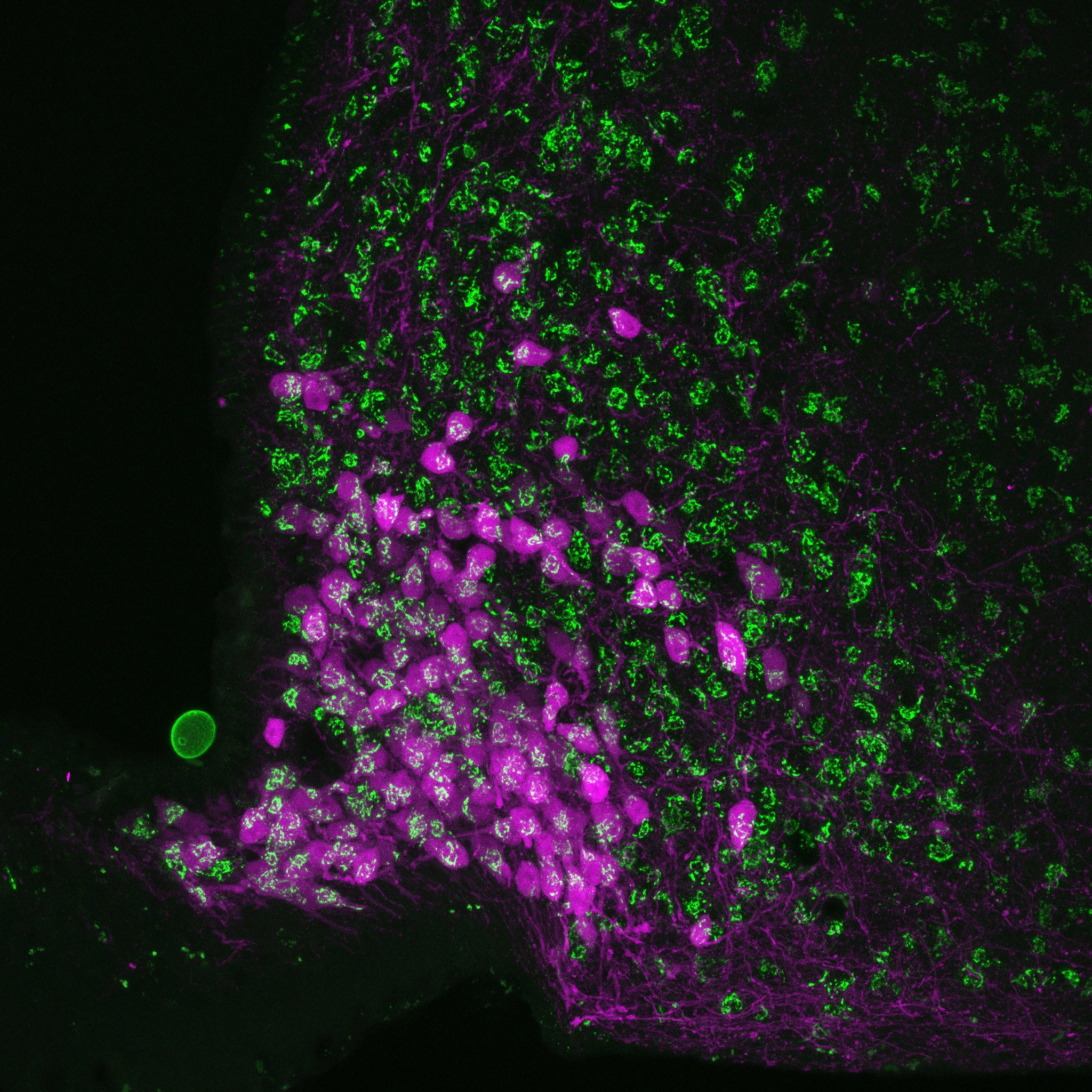

Supplement: Supplementary file 12 — Original data for Fig. 2a–d. [file 42255_2024_991_MOESM12_ESM.zip › Figure 2C/IGFRL-NPYGFP-COLOC-0-150nodapi.jpg]

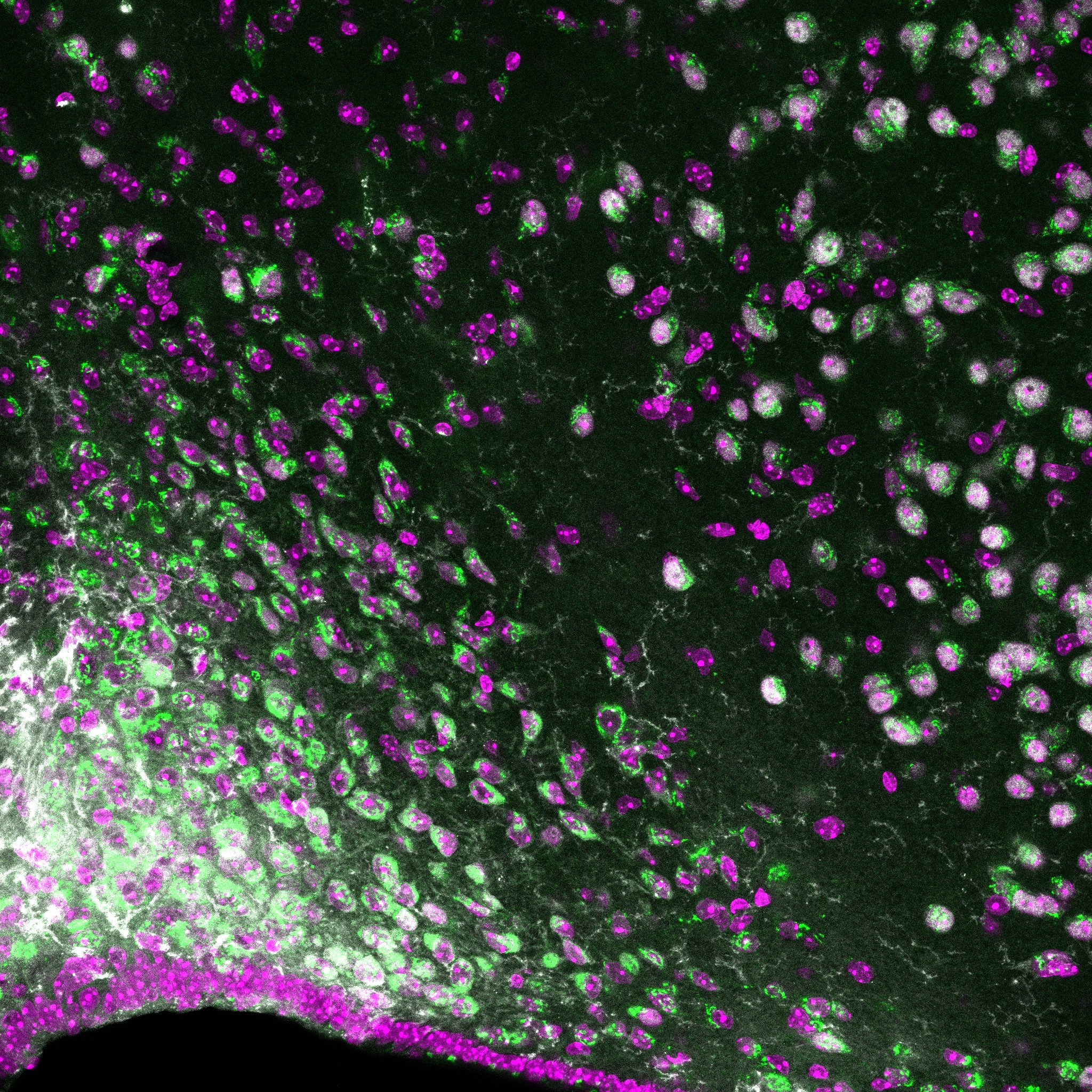

Supplement: Supplementary file 12 — Original data for Fig. 2a–d. [file 42255_2024_991_MOESM12_ESM.zip › Figure 2C/IGFRL-Neun-greenpurple-repr-contr.jpg]

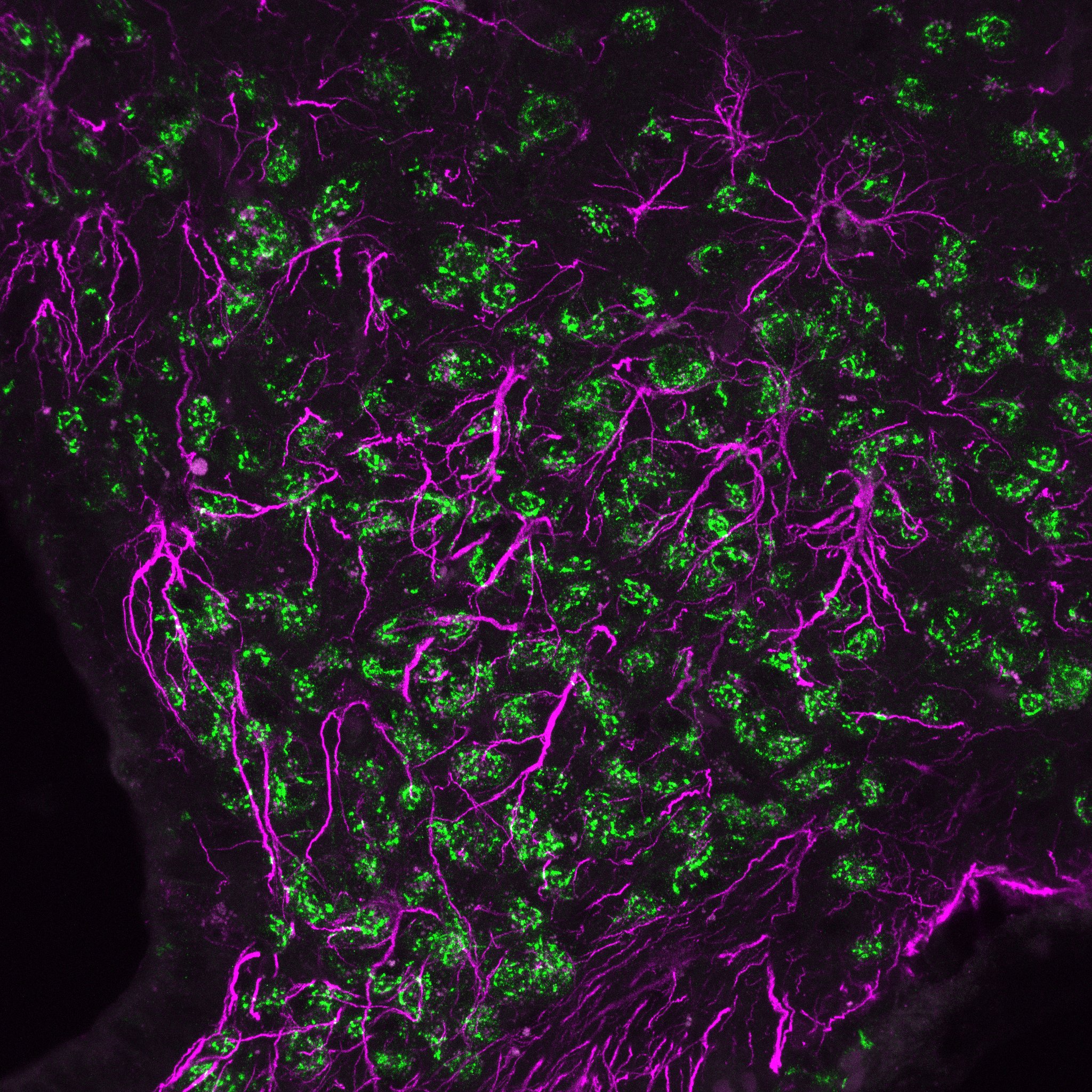

Supplement: Supplementary file 12 — Original data for Fig. 2a–d. [file 42255_2024_991_MOESM12_ESM.zip › Figure 2C/IGFRL-GFAP-COLOC-greenpurple-0-100-igfrl0-120.jpg]

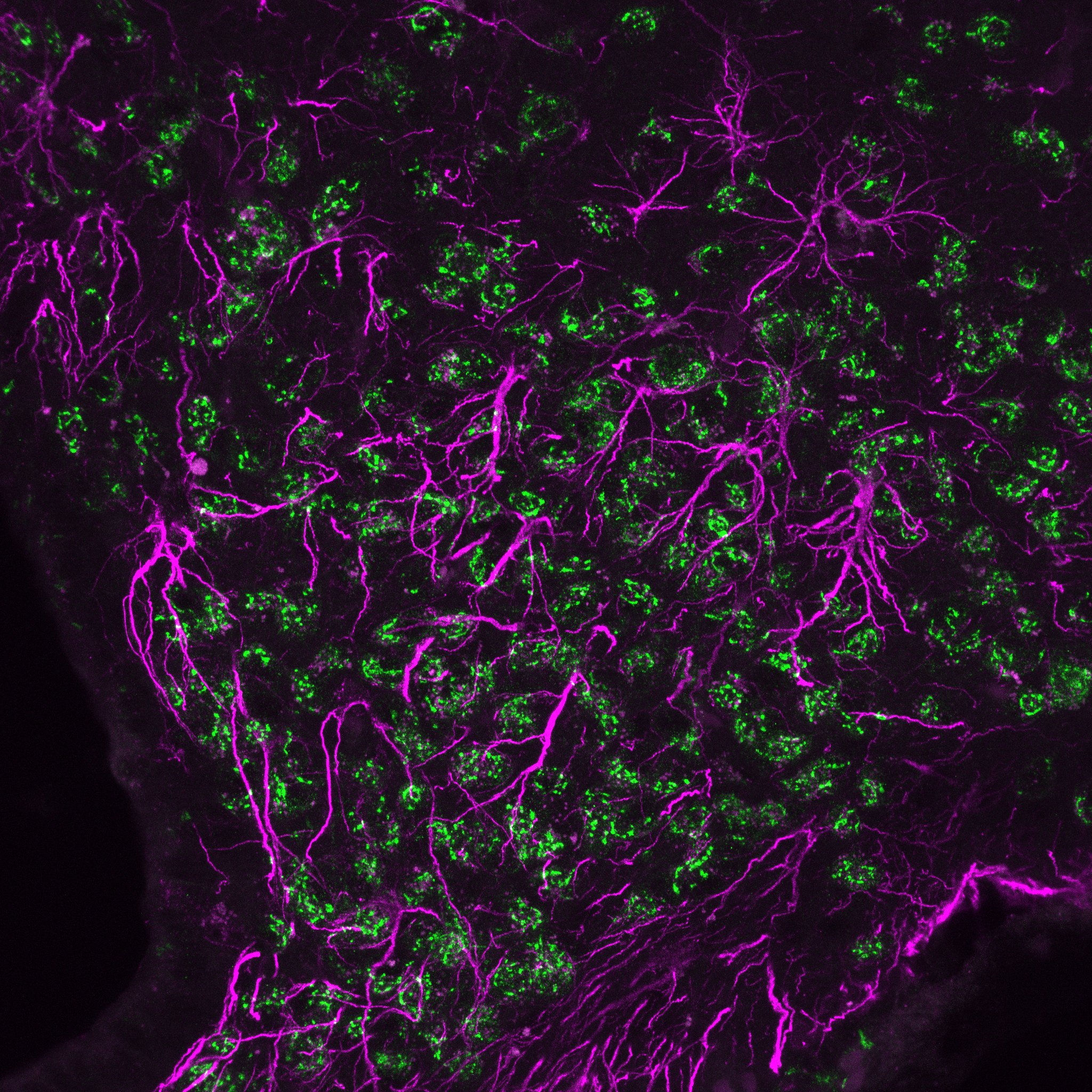

Supplement: Supplementary file 12 — Original data for Fig. 2a–d. [file 42255_2024_991_MOESM12_ESM.zip › Figure 2C/IGFRL-GFAP-COLOC-greenpurple-0-100.jpg]

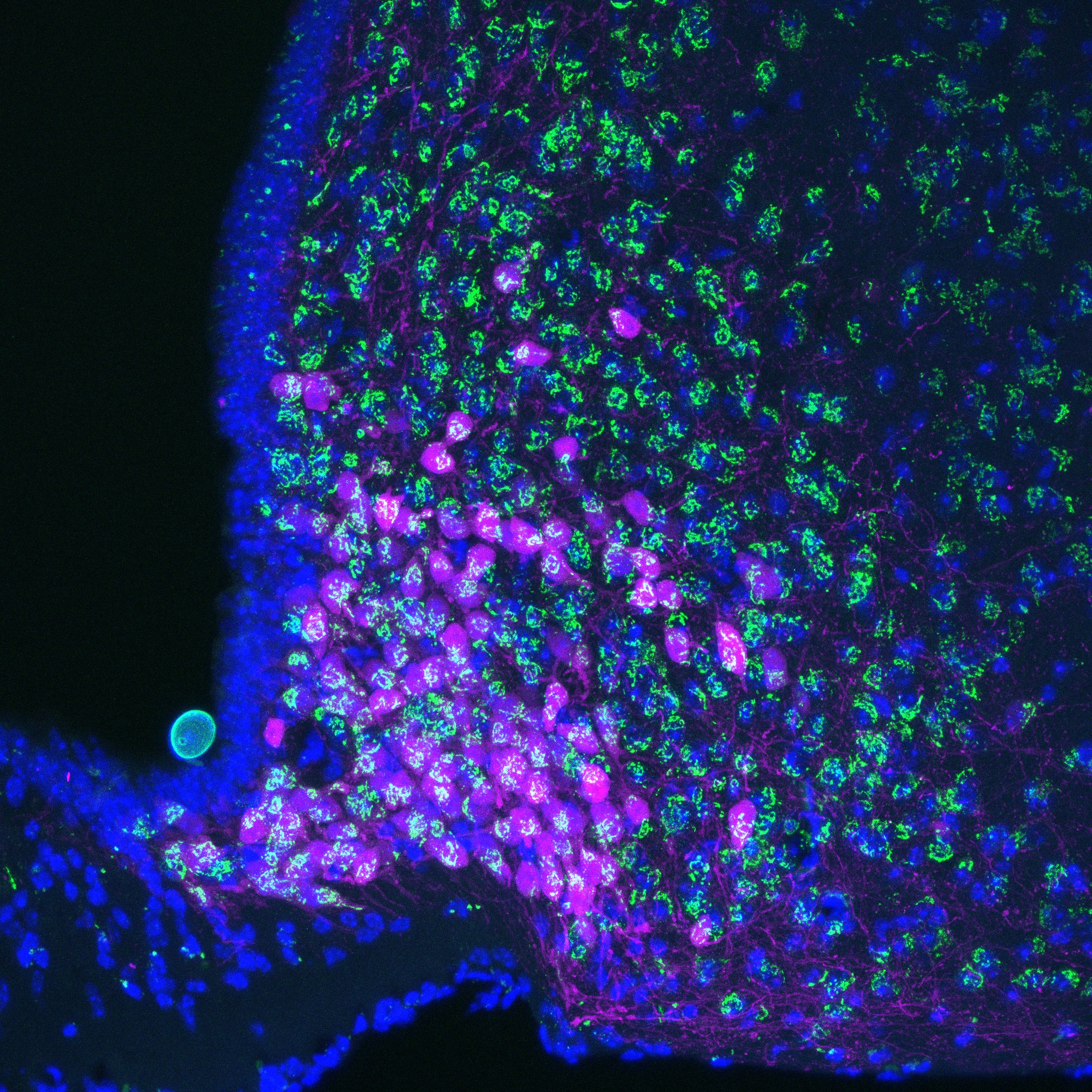

Supplement: Supplementary file 12 — Original data for Fig. 2a–d. [file 42255_2024_991_MOESM12_ESM.zip › Figure 2C/IGFRL-NPYGFP-COLOC-0-100.jpg]

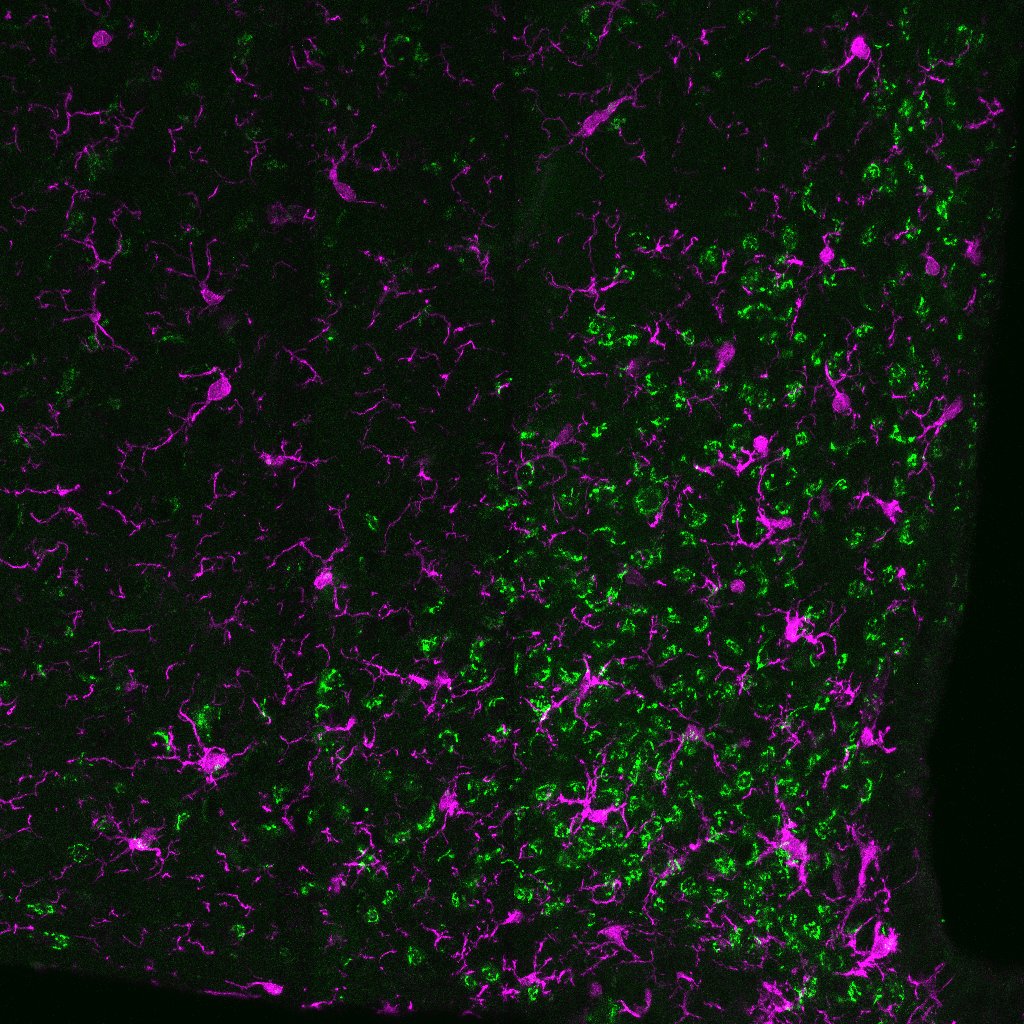

Supplement: Supplementary file 12 — Original data for Fig. 2a–d. [file 42255_2024_991_MOESM12_ESM.zip › Figure 2C/IGFRL-IBA1-COLOC-greenpurple-0-50.jpg]

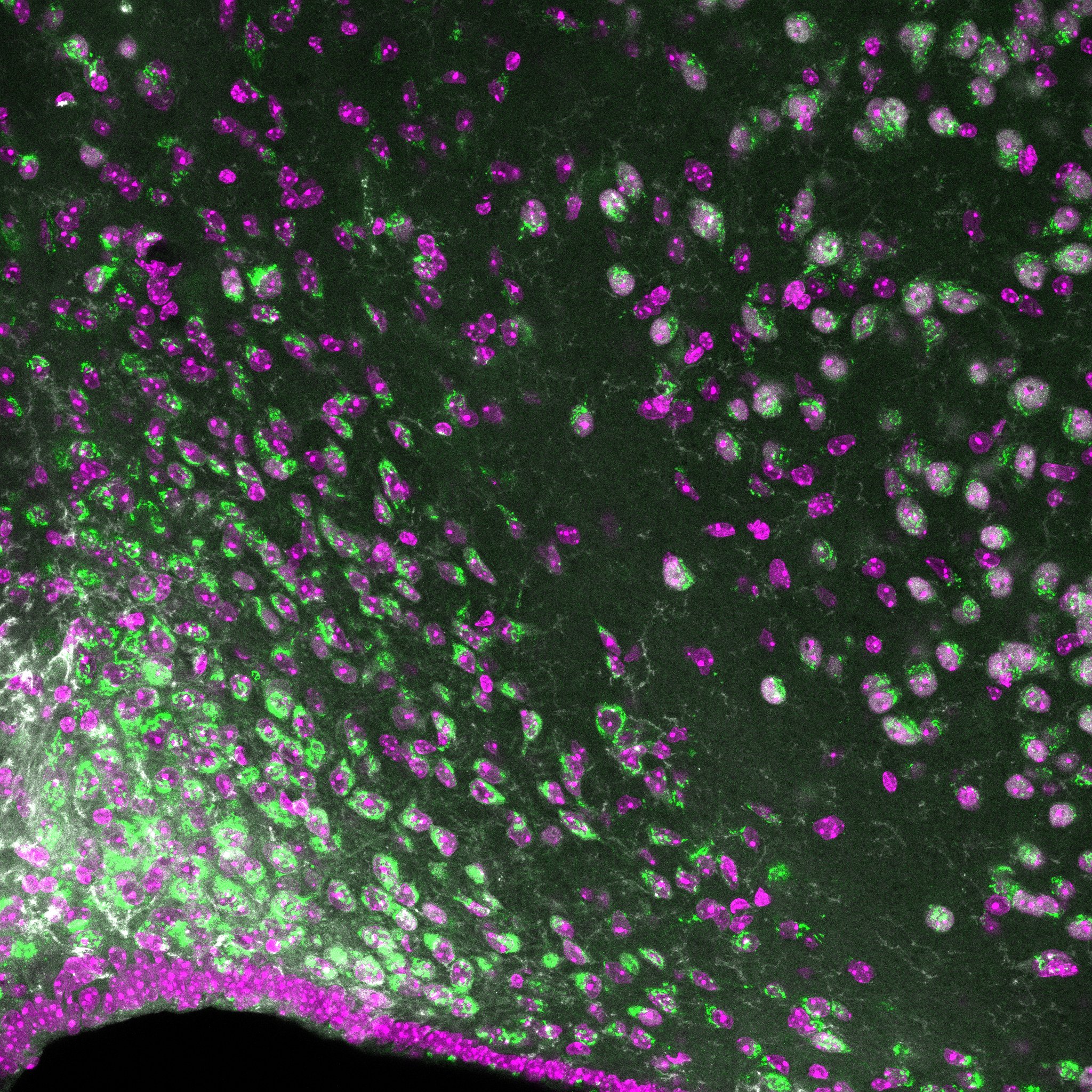

Supplement: Supplementary file 12 — Original data for Fig. 2a–d. [file 42255_2024_991_MOESM12_ESM.zip › Figure 2C/IGFRL-Neun-greenpurple-repr.jpg]

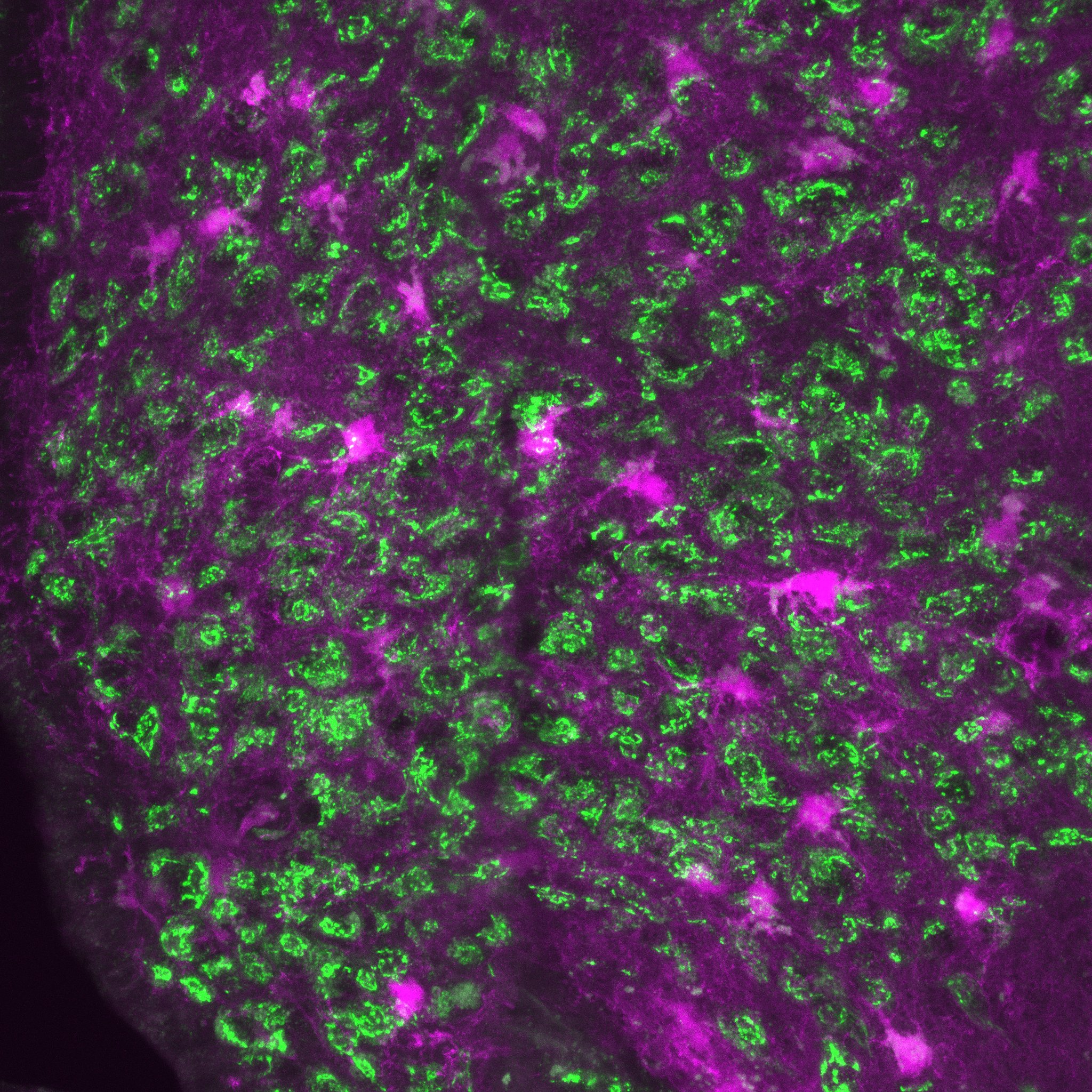

Supplement: Supplementary file 12 — Original data for Fig. 2a–d. [file 42255_2024_991_MOESM12_ESM.zip › Figure 2C/IGFRL-ALDH-COLOC-greenpurple.jpg]

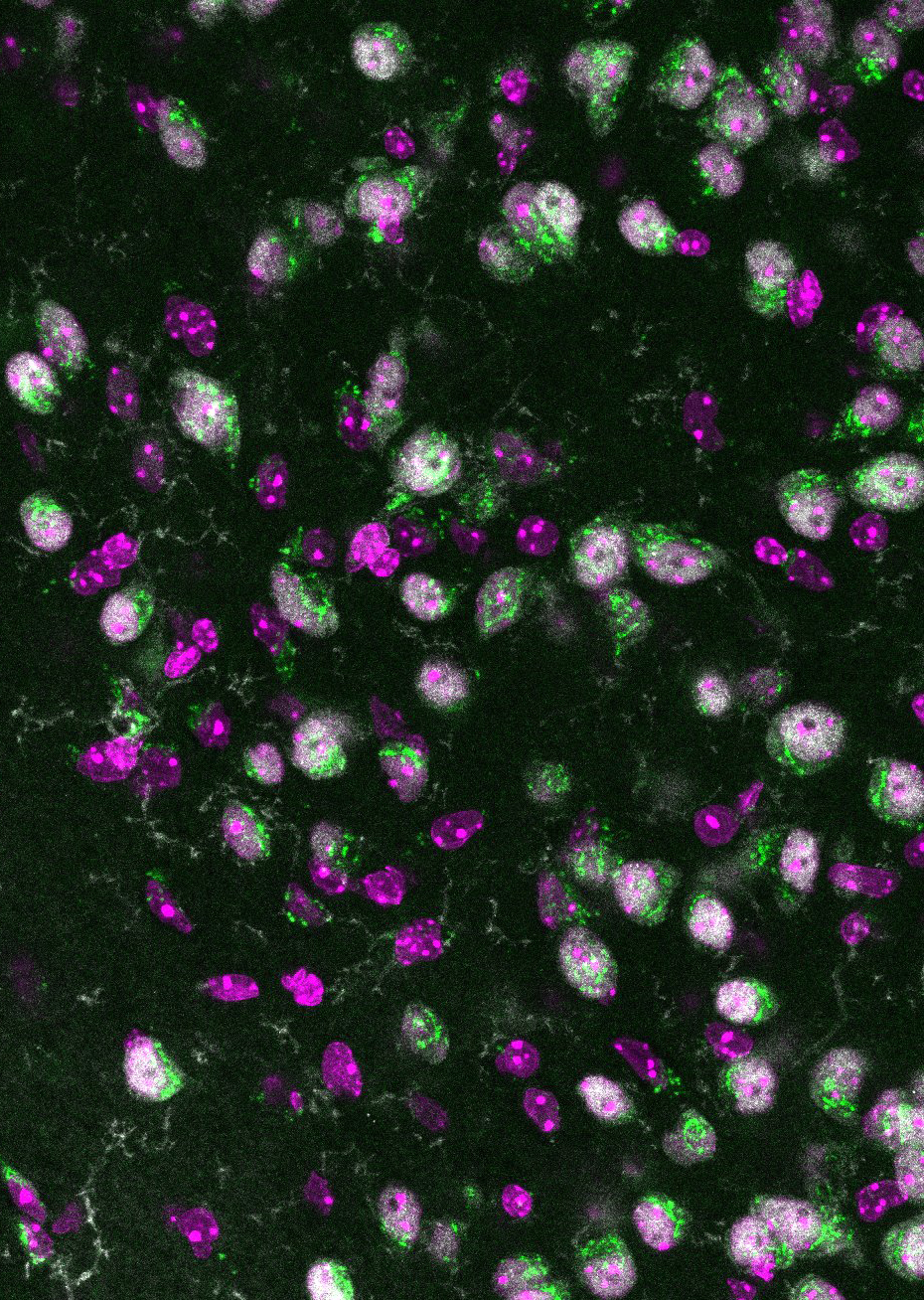

Supplement: Supplementary file 12 — Original data for Fig. 2a–d. [file 42255_2024_991_MOESM12_ESM.zip › Figure 2C/IGFRL-Neun-greenpurple-repr-vmh-contr.jpg]

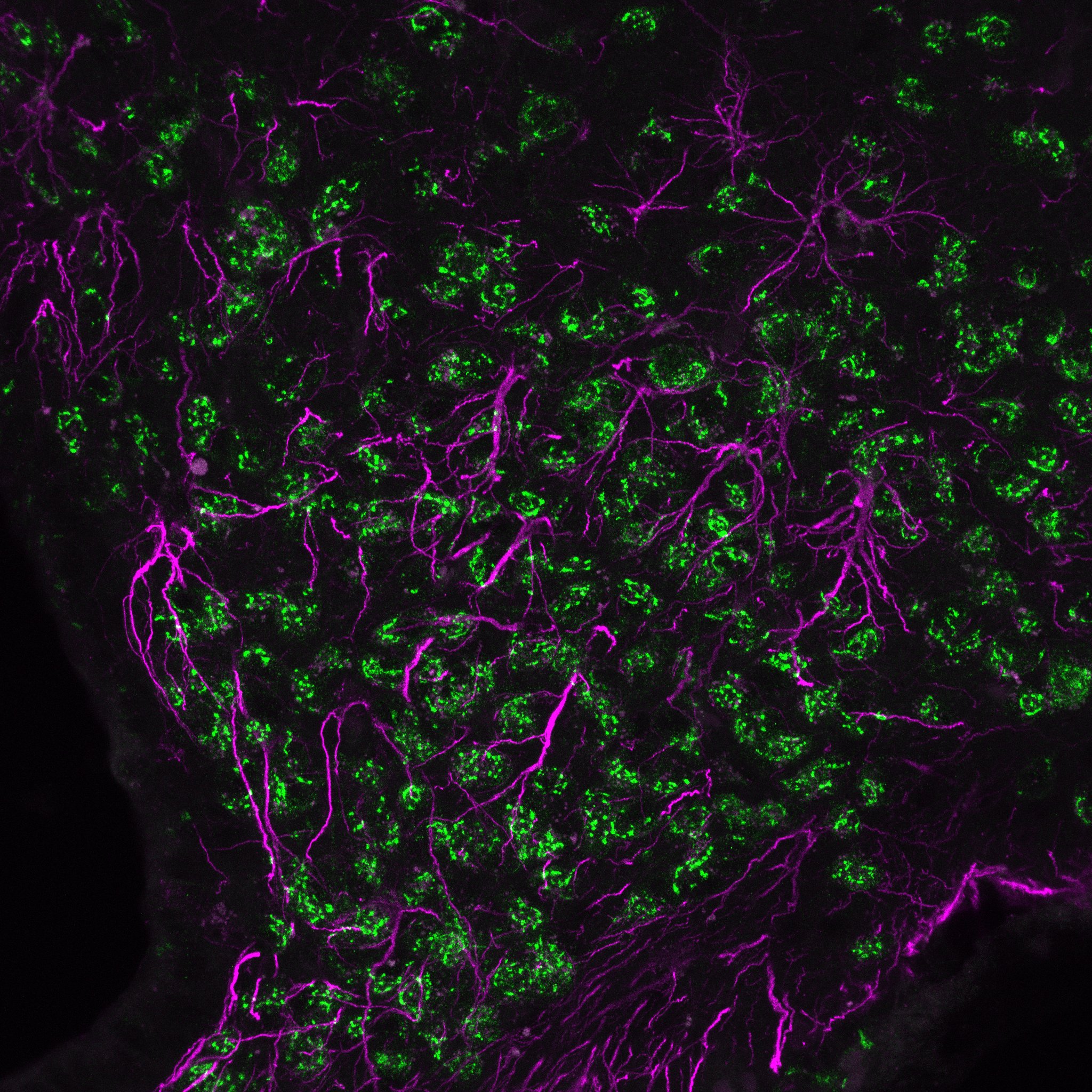

Supplement: Supplementary file 12 — Original data for Fig. 2a–d. [file 42255_2024_991_MOESM12_ESM.zip › Figure 2C/IGFRL-GFAP-COLOC-greenpurple-0-150.jpg]

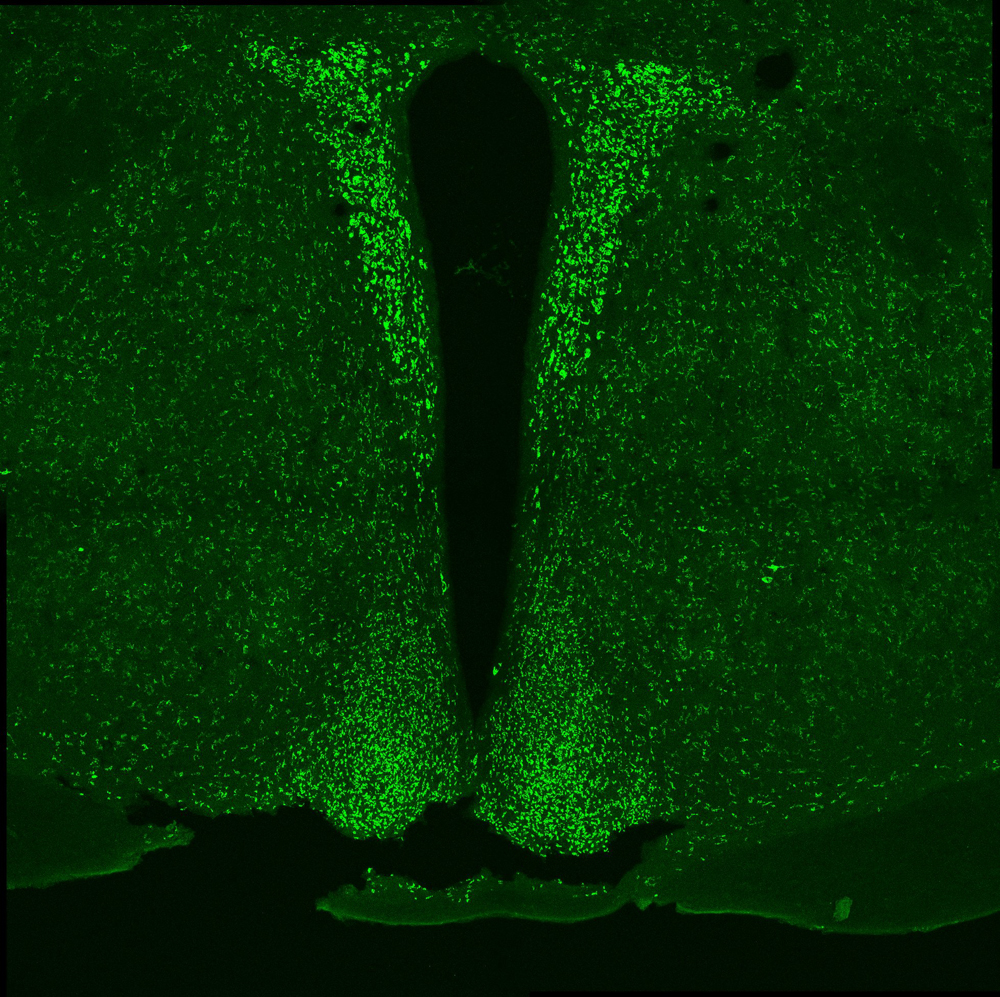

Supplement: Supplementary file 13 — Original data for Fig. 3a. [file 42255_2024_991_MOESM13_ESM.zip › Figure 3A/MAX_IGFRL-nestin-KOtest-251019.lif - wt34-pvh-20x.jpg]

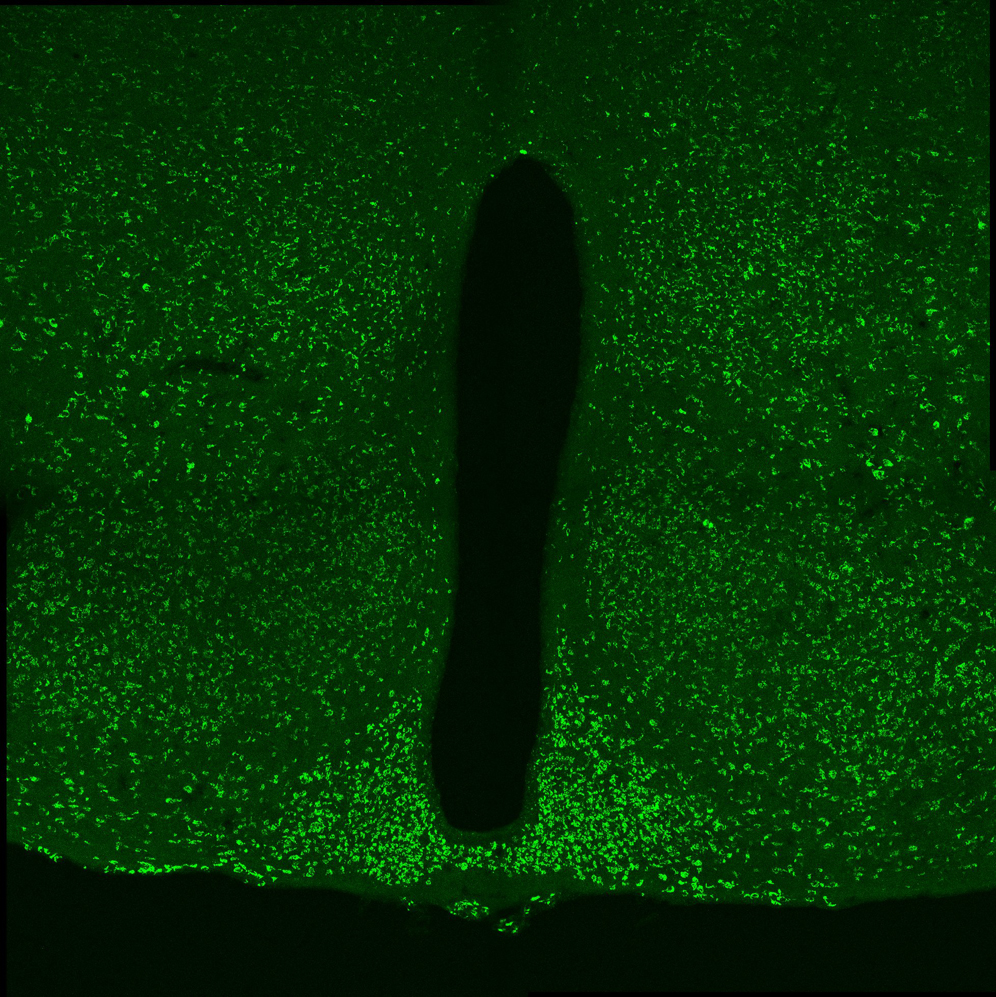

Supplement: Supplementary file 13 — Original data for Fig. 3a. [file 42255_2024_991_MOESM13_ESM.zip › Figure 3A/MAX_IGFRL-nestin-KOtest-251019.lif - wt34-arh-20x.jpg]

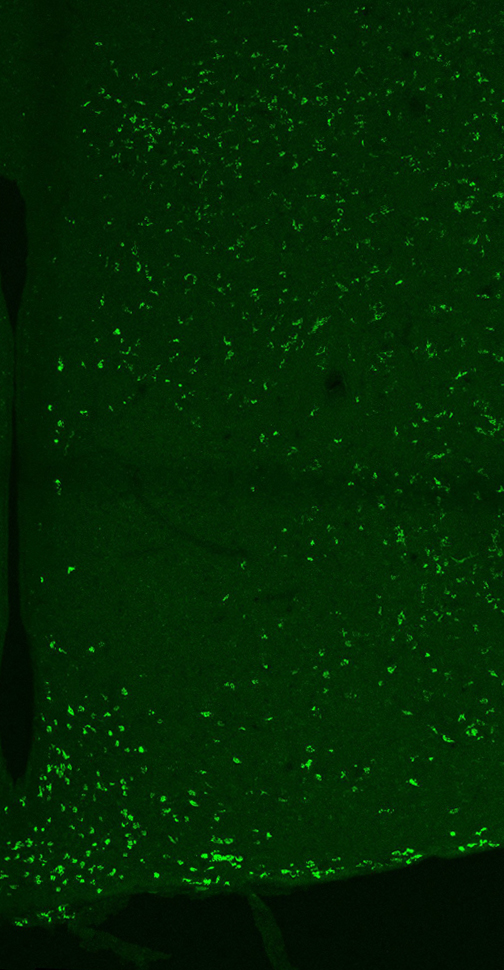

Supplement: Supplementary file 13 — Original data for Fig. 3a. [file 42255_2024_991_MOESM13_ESM.zip › Figure 3A/MAX_IGFRL-nestin-KOtest-251019.lif - ko48-arh-20x-straight.jpg]

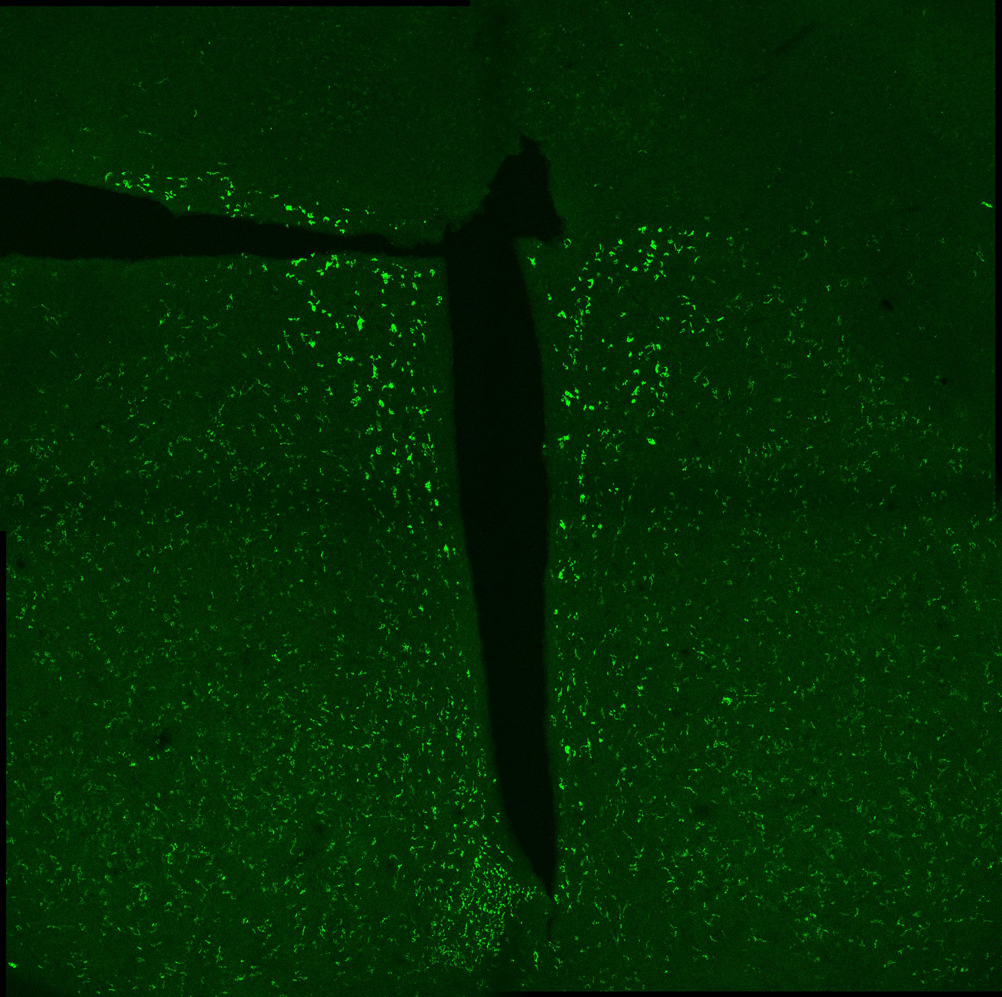

Supplement: Supplementary file 13 — Original data for Fig. 3a. [file 42255_2024_991_MOESM13_ESM.zip › Figure 3A/MAX_IGFRL-nestin-KOtest-251019.lif - ko38-pvh-20x.jpg]

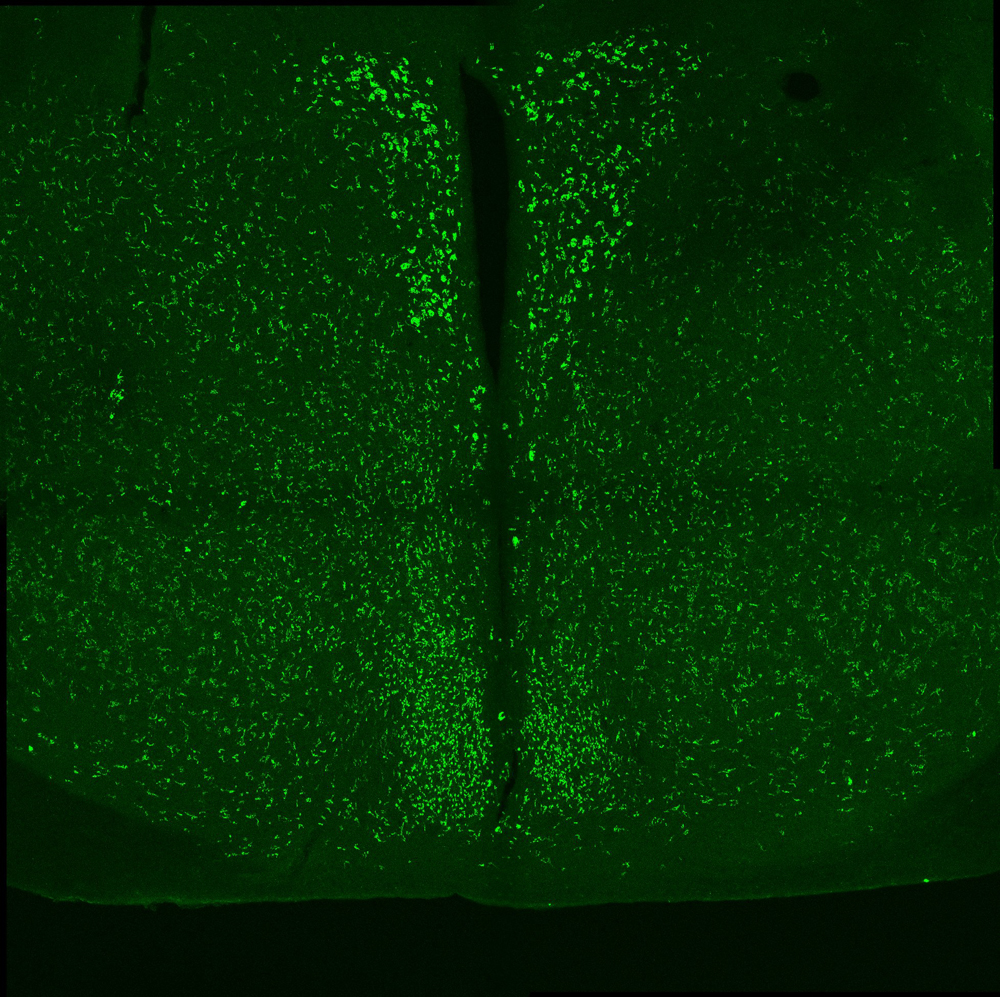

Supplement: Supplementary file 13 — Original data for Fig. 3a. [file 42255_2024_991_MOESM13_ESM.zip › Figure 3A/MAX_IGFRL-nestin-KOtest-251019.lif - ko48-pvh-20x.jpg]

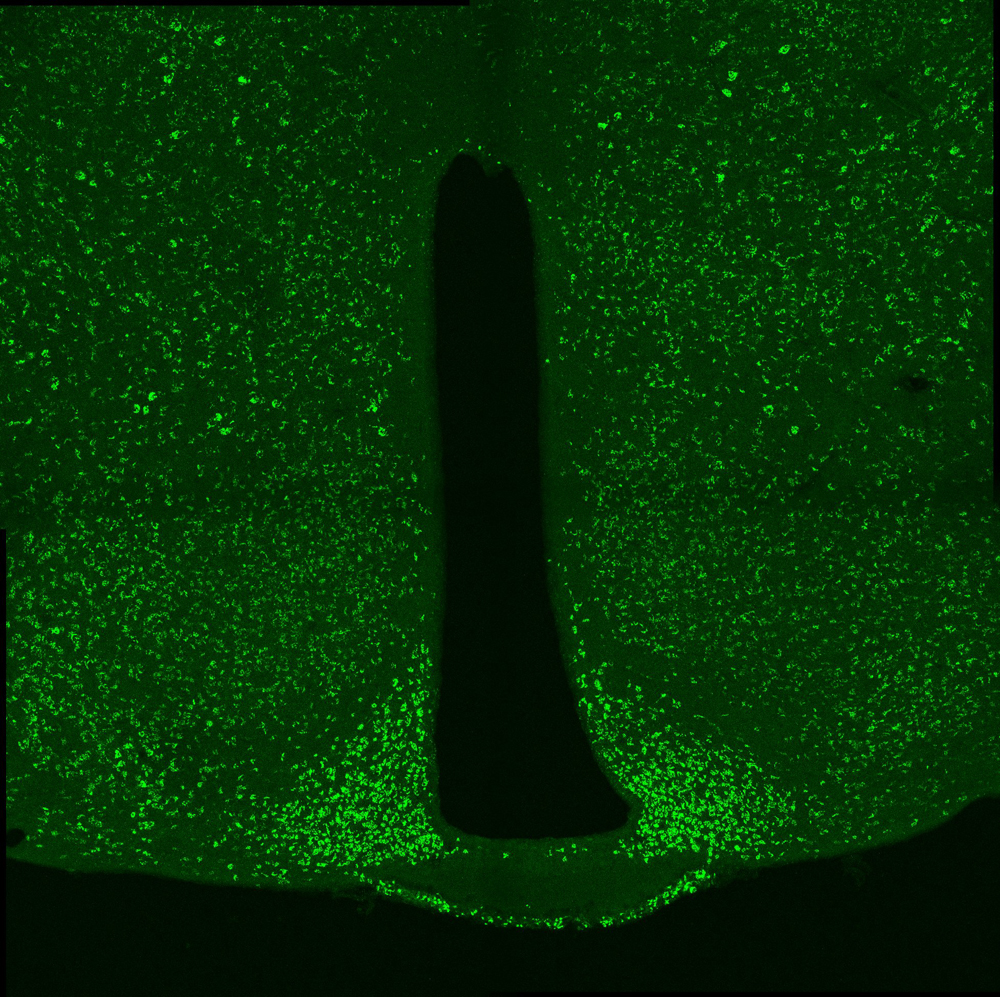

Supplement: Supplementary file 13 — Original data for Fig. 3a. [file 42255_2024_991_MOESM13_ESM.zip › Figure 3A/MAX_IGFRL-nestin-KOtest-251019.lif - wt33-arh-20x.jpg]

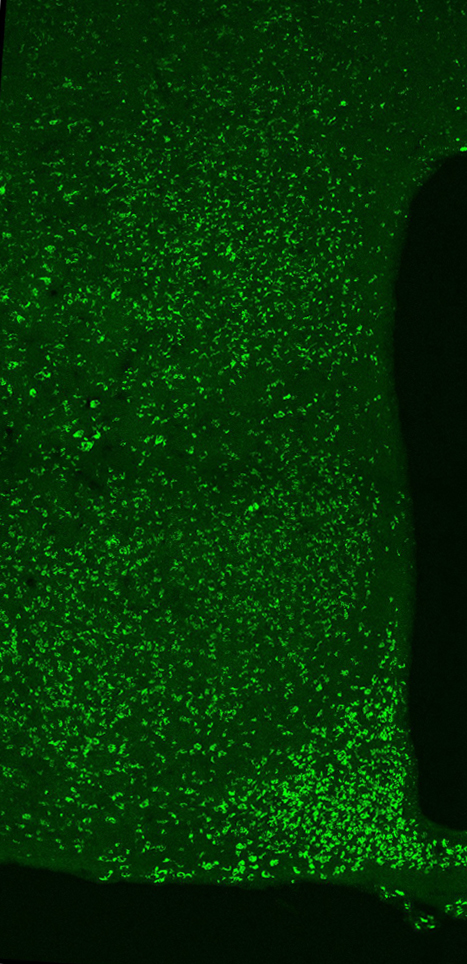

Supplement: Supplementary file 13 — Original data for Fig. 3a. [file 42255_2024_991_MOESM13_ESM.zip › Figure 3A/MAX_IGFRL-nestin-KOtest-251019.lif - wt34-arh-20x-straight.jpg]

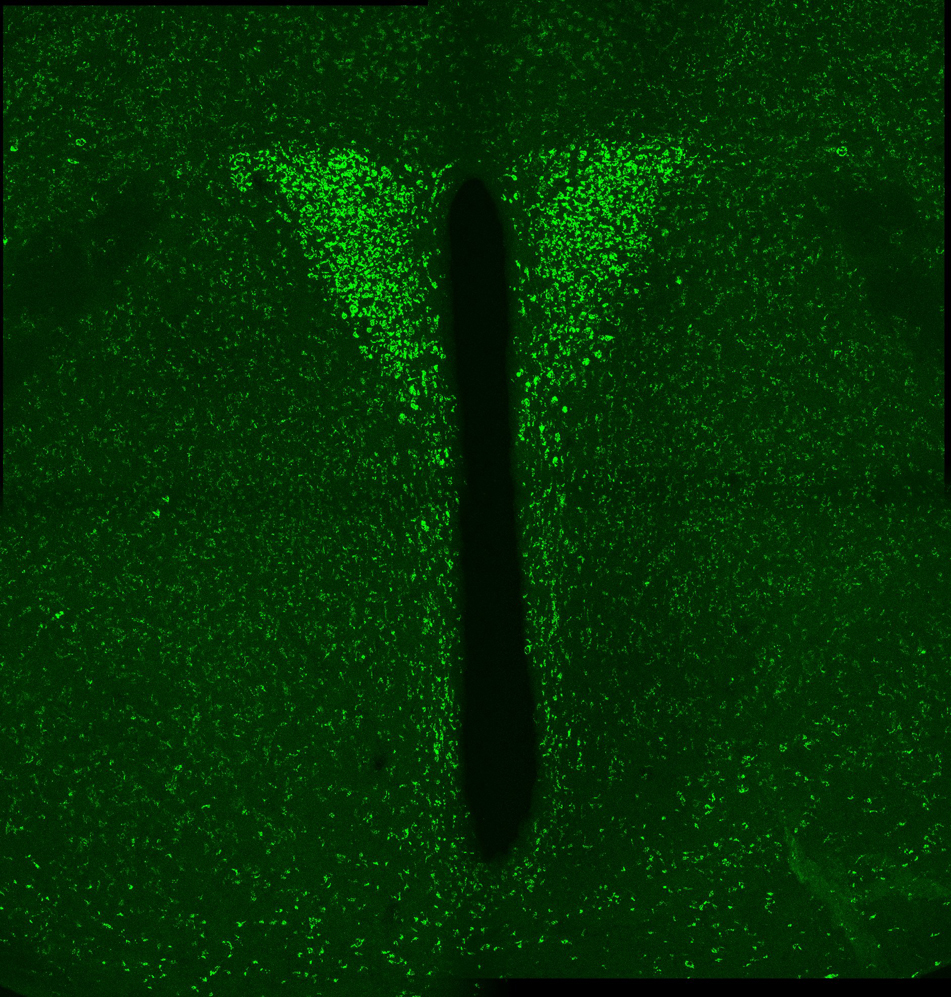

Supplement: Supplementary file 13 — Original data for Fig. 3a. [file 42255_2024_991_MOESM13_ESM.zip › Figure 3A/MAX_IGFRL-nestin-KOtest-251019.lif - wt33-pvh-20x.jpg]

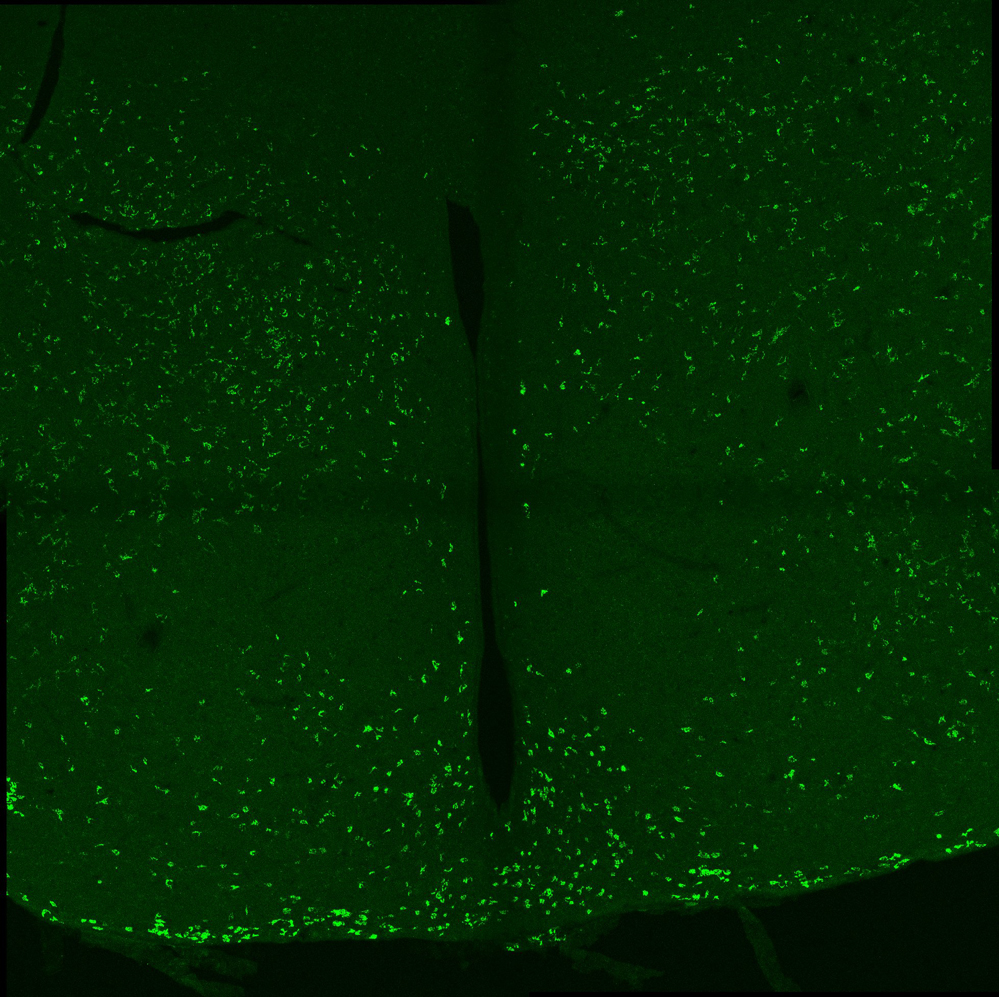

Supplement: Supplementary file 13 — Original data for Fig. 3a. [file 42255_2024_991_MOESM13_ESM.zip › Figure 3A/MAX_IGFRL-nestin-KOtest-251019.lif - ko48-arh-20x.jpg]

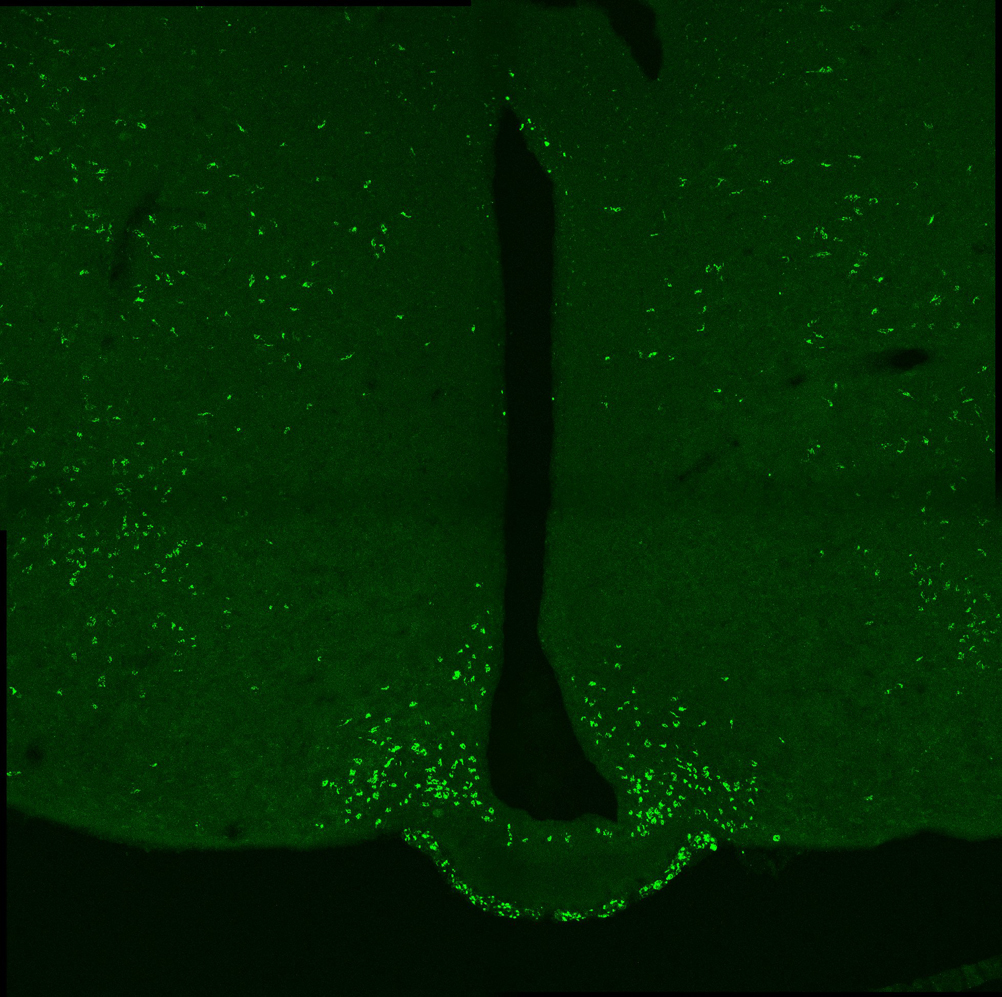

Supplement: Supplementary file 13 — Original data for Fig. 3a. [file 42255_2024_991_MOESM13_ESM.zip › Figure 3A/MAX_IGFRL-nestin-KOtest-251019.lif - ko38-arh-20x.jpg]

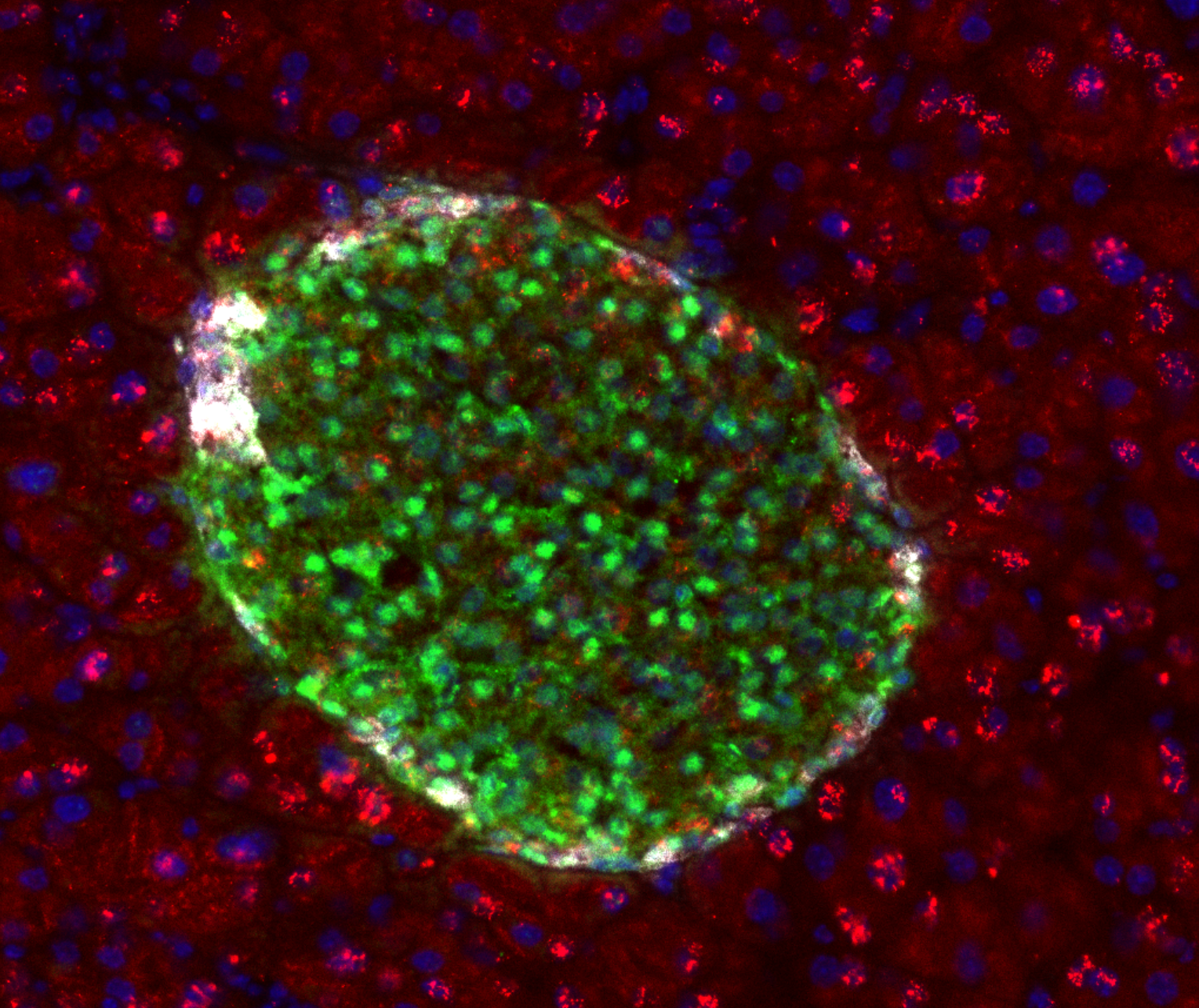

Supplement: Supplementary file 14 — Original data for Fig. 4a. [file 42255_2024_991_MOESM14_ESM.zip › Figure 4A/IDO Grandl Pancr cryo Ins750 Gcg633 Inc555 formalin rescan WT17.tiff]

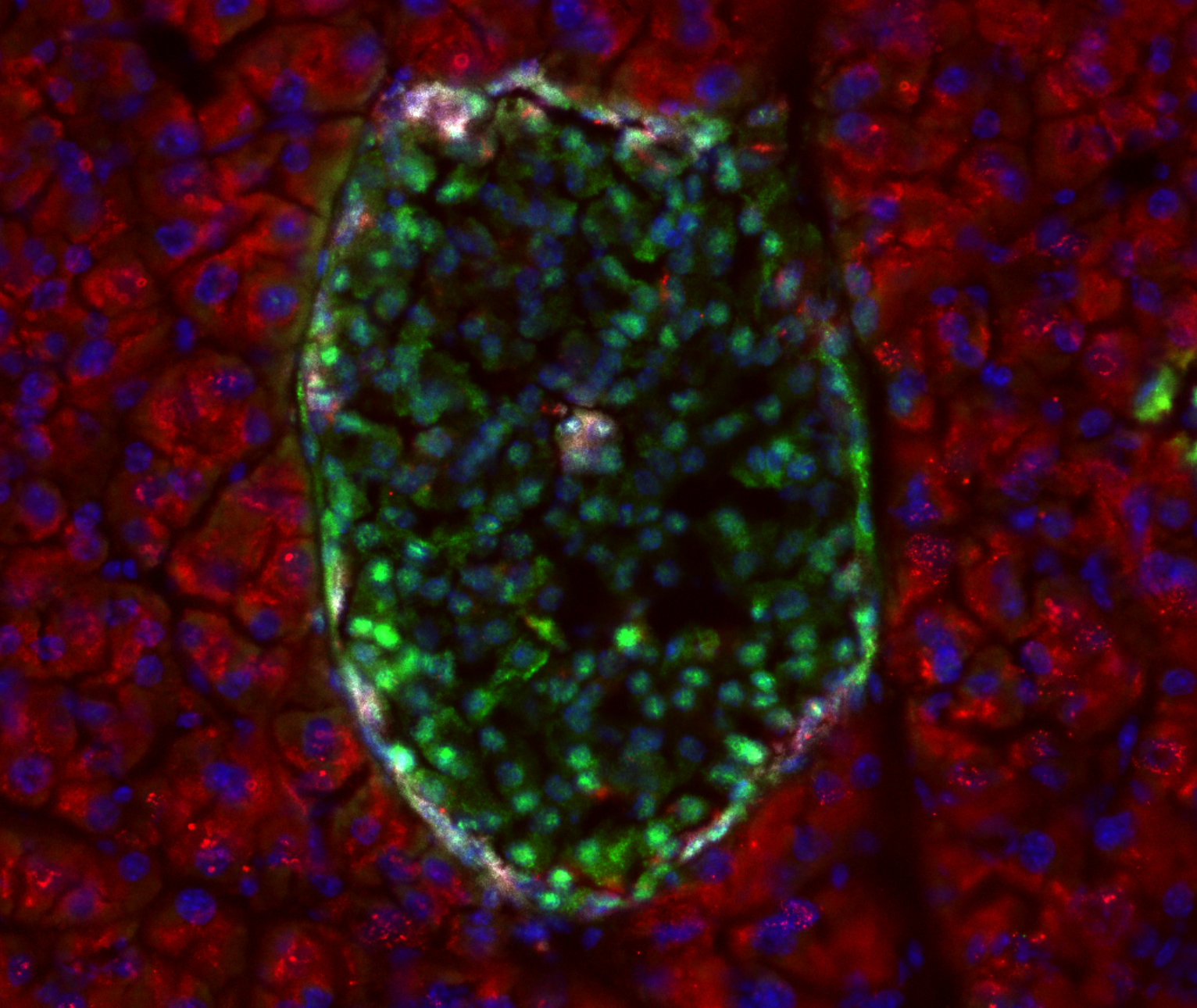

Supplement: Supplementary file 14 — Original data for Fig. 4a. [file 42255_2024_991_MOESM14_ESM.zip › Figure 4A/IDO Grandl Pancr cryo Ins750 Gcg633 Inc555 formalin rescan KO39.tiff]

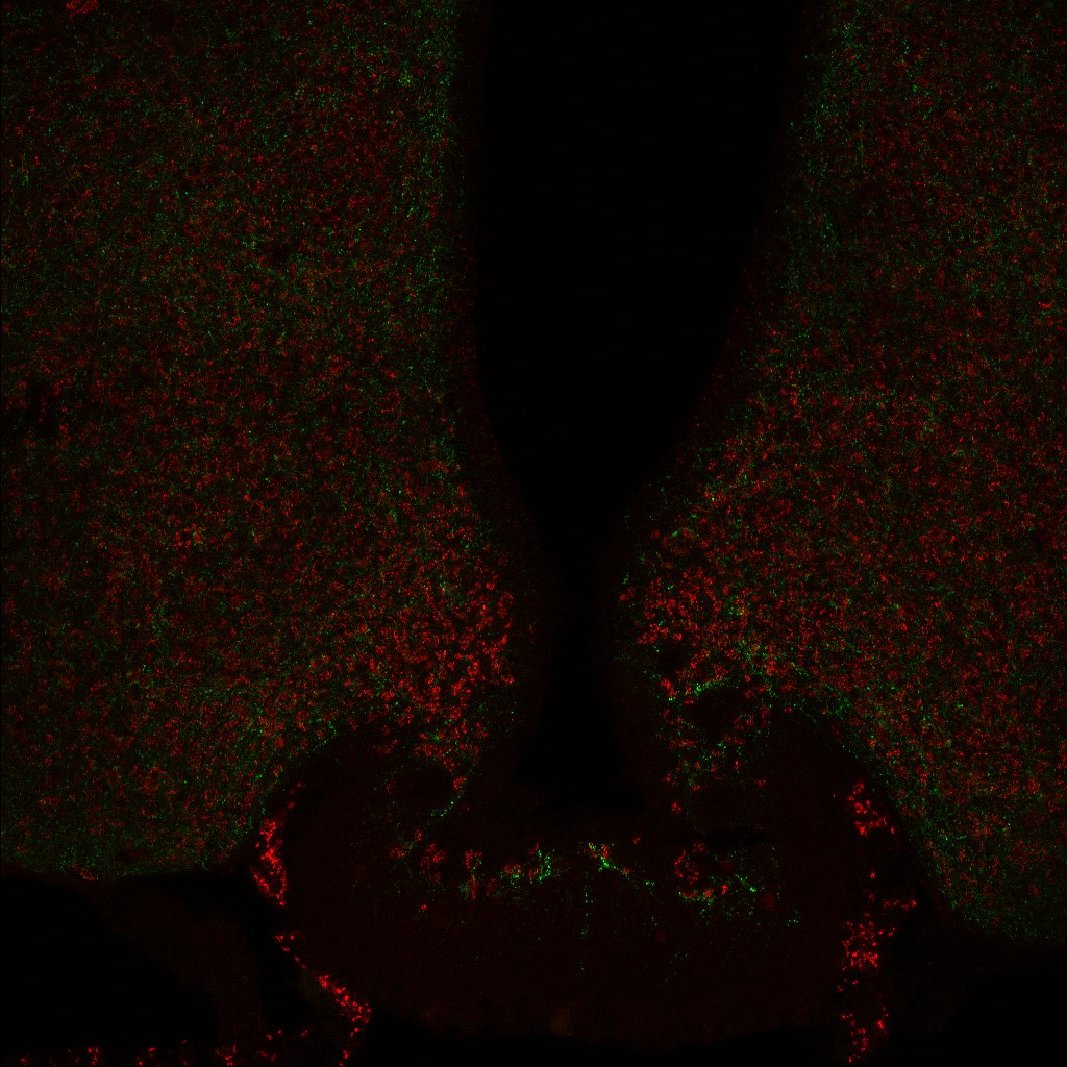

Supplement: Supplementary file 15 — Original data for Extended Data Fig. 3a,j. [file 42255_2024_991_MOESM15_ESM.zip › Extended Data Figure 3A/IGFRL-AgRP-WT-Vhcl2-ARH20x-1.jpg]

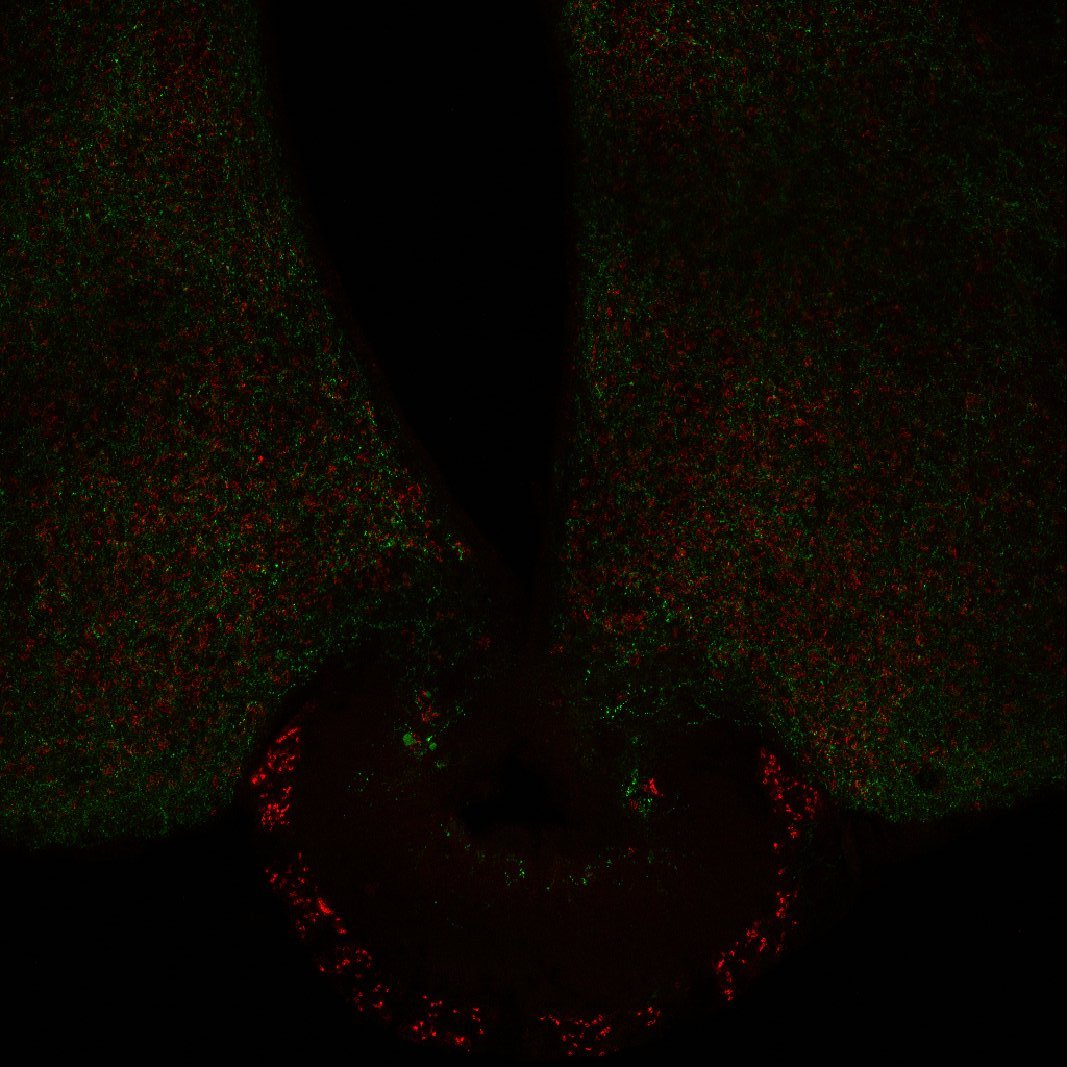

Supplement: Supplementary file 15 — Original data for Extended Data Fig. 3a,j. [file 42255_2024_991_MOESM15_ESM.zip › Extended Data Figure 3A/IGFRL-AgRP-KO-87f-ARH20x-1.jpg]

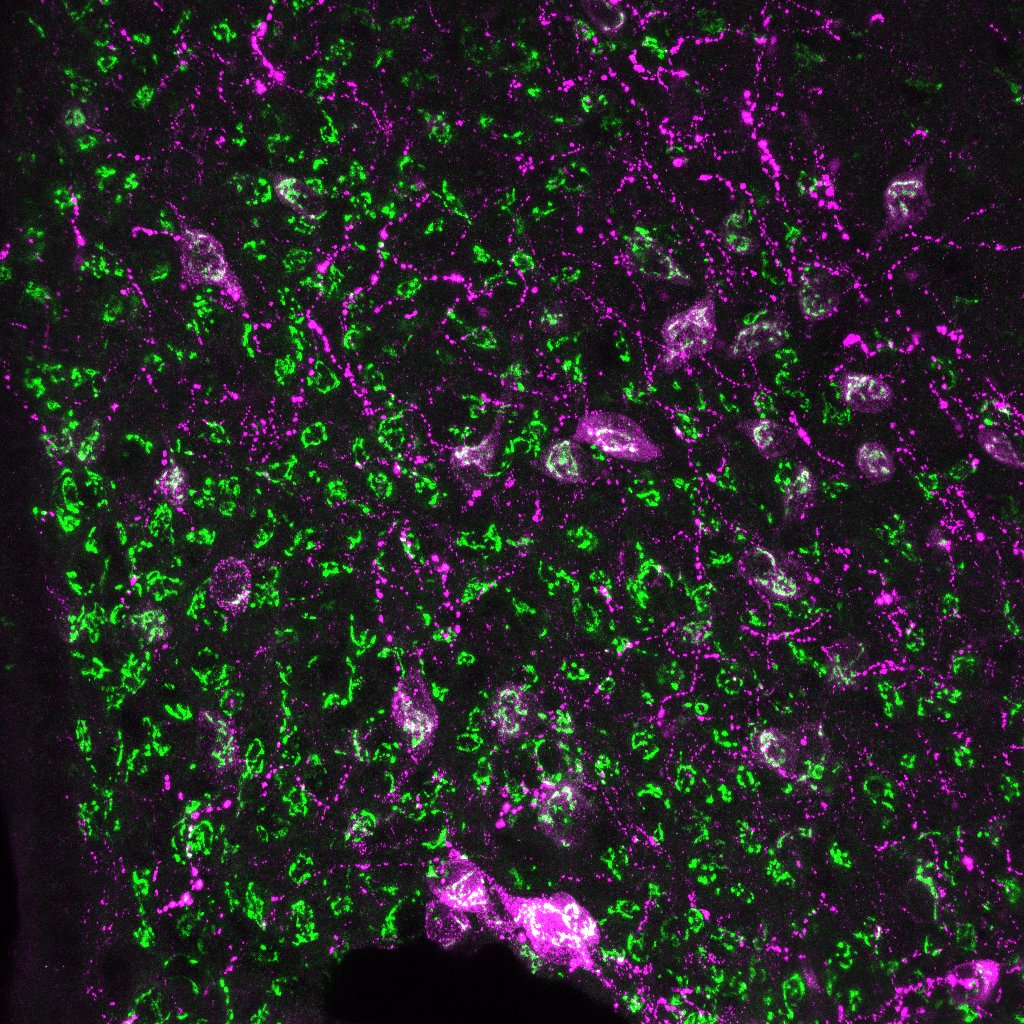

Supplement: Supplementary file 15 — Original data for Extended Data Fig. 3a,j. [file 42255_2024_991_MOESM15_ESM.zip › Extended Data Figure 3J/MAX_12m-IGFRLflox-CreNegative.lif - Series008-pomcko-wt-repr.jpg]

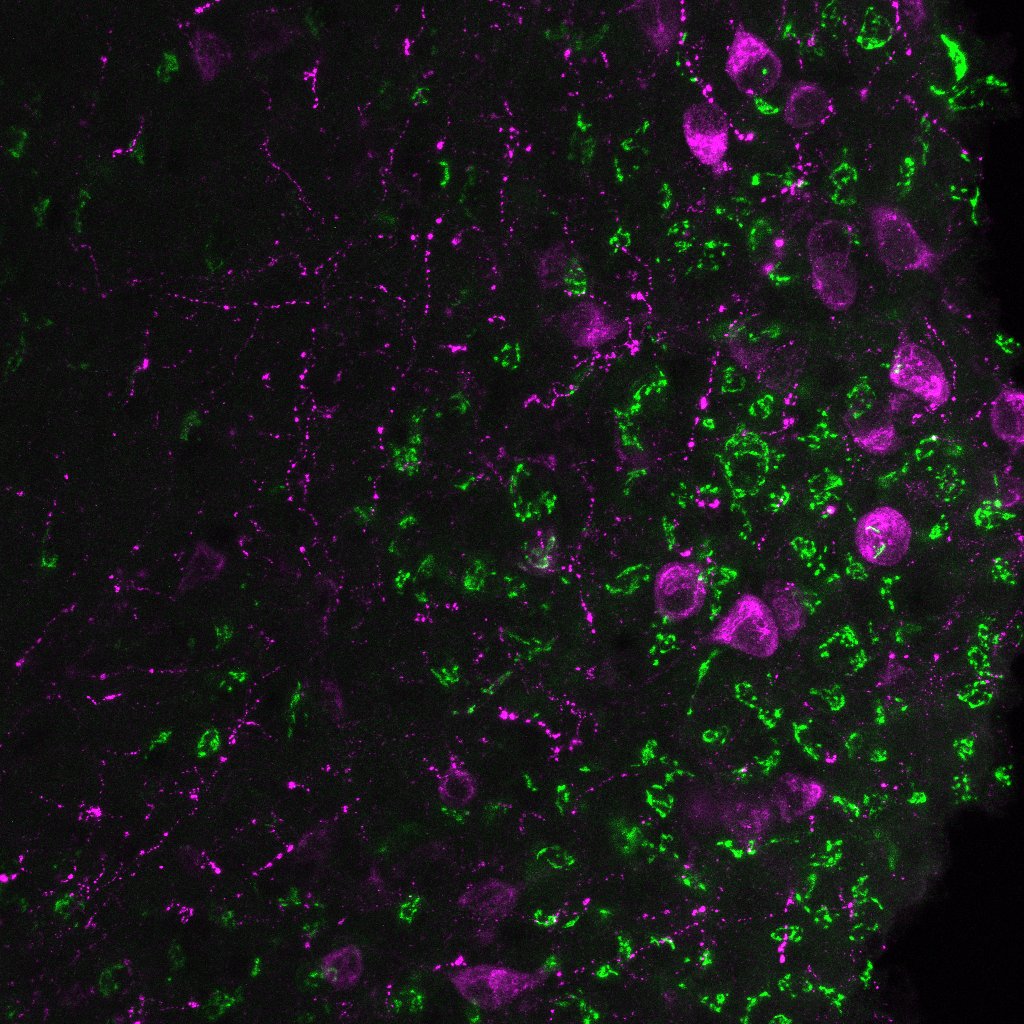

Supplement: Supplementary file 15 — Original data for Extended Data Fig. 3a,j. [file 42255_2024_991_MOESM15_ESM.zip › Extended Data Figure 3J/MAX_9m-POMC-IGFRLKO.lif - Series003-1-pomcko-ko-repr.jpg]

phospho-AKT

kD

150

100

75

50

37

25

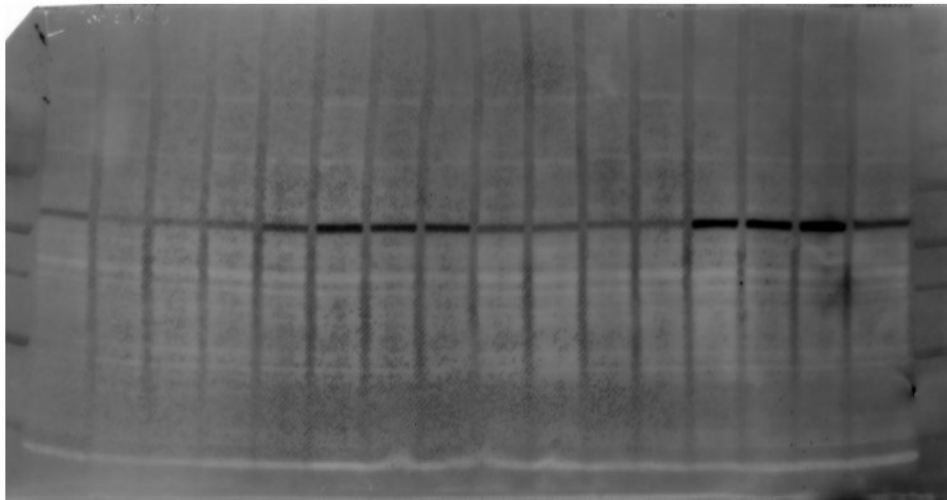

total AKT

1- 100 KC

kD  
150  
100  
75  
50  
37  
25

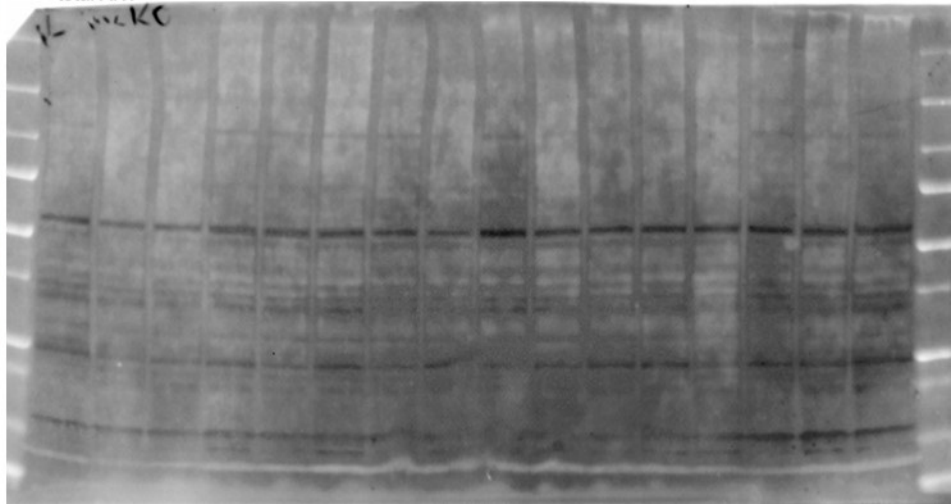

Supplement: Supplementary file 16 — Unprocessed western blots for Fig. 1k. [file 42255_2024_991_MOESM16_ESM.pdf]
